# Charge density studies of multicentre two-electron bonding of an anion radical at non-ambient temperature and pressure

**DOI:** 10.1107/S2052252521005273

**Published:** 2021-06-12

**Authors:** Valentina Milašinović, Krešimir Molčanov, Anna Krawczuk, Nikita E. Bogdanov, Boris A. Zakharov, Elena V. Boldyreva, Christian Jelsch, Biserka Kojić-Prodić

**Affiliations:** a Department of Physical Chemistry, Rudjer Bošković Institute, Bijenička 54, Zagreb 10000, Croatia; b Institute of Inorganic Chemistry, University of Göttingen, Tammannstrasse 4, Göttingen 37077, Germany; c Faculty of Chemistry, Jagiellonian University in Krakow, Gronostajowa 2, Krakow 30-387, Poland; d Boreskov Institute of Catalysis, SB RAS, Lavrentiev Avenue 5, Novosibirsk 630090, Russian Federation; e Novosibirsk State University, Pirogova Street 2, Novosibirsk 630090 Russian Federation; f CRM2, CNRS, UMR 7036, Université de Lorraine, BP 70239 Nancy, France

**Keywords:** π-stacking, non-aromatic rings, multicentre bonding, charge density, high pressure

## Abstract

The variation of charge density of two-electron multicentre bonding (pancake bonding) between semi­quinone radicals with pressure and temperature was studied on a salt of 5,6-di­chloro-2,3-di­cyano­semi­quinone radical anion (DDQ) with 4-cyano-*N*-methyl­pyridinium cation (4-CN) using the Transferable Aspheric Atom Model (TAAM) refinement. The pancake-bonded radical dimers are stacked by non-bonding π-interactions. With rising pressure, the covalent character of interactions between radicals increases, and above 2.55 GPa, the electron density indicates multicentric covalent interactions throughout the stack. The experimental charge densities were verified and corroborated by periodic DFT computations. The TAAM approach has been tested and validated for atomic resolution data measured at ambient pressure; this work shows this approach can also be applied to diffraction data obtained at pressures up to several gigapascals.

## Abstract 2

The procedure for study of charge density at high pressures and/or high temperatures by the transferrable aspherical atom model (TAAM) is described and discussed. The experimental charge densities were verified and corroborated by periodic DFT computation. This method was tested on a salt of 5,6-di­chloro-2,3-di­cyano­semi­quinone radical anion which comprises two-electron multicentre bonding between the radicals.

## 1. Introduction   

X-ray charge density analysis is considered to be the most powerful experimental method to study interatomic and intermolecular interactions (Koritsanszky & Coppens, 2001 ▸; Lecomte *et al.*, 2003 ▸; Munshi & Guru Row, 2005 ▸; Stalke, 2011 ▸, 2015 ▸; Macchi, 2013 ▸, 2021 ▸; Korlyukov & Nelyubina, 2019 ▸). Its results are directly comparable to those obtained by quantum chemical computations (Grabowsky *et al.*, 2017 ▸; Genoni *et al.*, 2017 ▸, 2018*a* ▸, ▸ *b*; Genoni & Macchi, 2020 ▸). The combination of experimental and theoretical charge densities using the atoms in molecules (AIM) approach is the basis of modern quantum crystallography (Macchi, 2013 ▸, 2021 ▸; Macchi *et al.*, 2015 ▸; Grabowsky *et al.*, 2017 ▸; Genoni *et al.*, 2018*a* ▸,*b* ▸; Korlyukov & Nelyubina, 2019 ▸; Genoni & Macchi, 2020 ▸). However, obtaining good high-resolution diffraction data still remains an experimental challenge, and is generally limited to high-quality crystals, low temperatures (typically 30–100 K) and ambient pressure. This unfortunately leaves many interesting chemical phenomena out of reach of experimental charge density studies. Up to date experimental charge densities under high pressure were recently obtained using synchrotron radiation for only two compounds, *syn*-1,6:8,13-bis­carbonyl­[14]annulene (Casati *et al.*, 2017*a* ▸,*b* ▸) and grossular Ca_3_Al_2_(SiO_4_)_3_ (Gajda *et al.*, 2020 ▸).

To circumvent the problem of obtaining charge densities from moderate-quality diffraction data, the Transferrable Aspherical Atom Model (TAAM) was proposed and first applied almost three decades ago (Brock *et al.*, 1991 ▸). It is based on the chemical reasoning that the same atoms or atom groups behave analogously regardless of the rest of the molecule (termed residue) (Brock *et al.*, 1991 ▸; Korlyukov & Nelyubina, 2019 ▸). Thus, the electron density of atoms with similar environments (*e.g.* carbonyl or hydroxyl groups) could be modelled using very similar multipolar parameters. A validated (Bąk *et al.*, 2011 ▸) approximation is to use averaged multipoles and kappa parameters stored in a database (Pichon-Pesme *et al.*, 1995 ▸) that can be applied to crystal structures for which diffraction data are not sufficient to allow a proper multipolar refinement (Zarychta *et al.*, 2007 ▸; Bąk *et al.*, 2011 ▸; Domagała *et al.*, 2012 ▸; Gajda *et al.*, 2014 ▸; Nassour *et al.*, 2017 ▸). Since the first compilation of data by Lecomte and coworkers (Pichon-Pesme *et al.*, 1995 ▸), several databases of such multipoles have been established; two are derived from quantum computations (Koritsanszky *et al.*, 2002 ▸; Dittrich *et al.*, 2004 ▸, 2013 ▸; Jarzembska & Dominiak, 2012 ▸), and one by averaging numerous experimental datasets (Zarychta *et al.*, 2007 ▸; Domagała *et al.*, 2012 ▸). These databases were at first limited to peptides, but were later expanded to include a broader variety of organic compounds; however, structures with heavy atoms and transition metals still remain out of reach of TAAM. Also, to date no structures containing organic radicals have been studied by TAAM.

Since TAAM refinement has seldom been used, it remains an open question, what amount of data can be extracted from these charge densities, and what is the minimum resolution and data quality that would justify use of TAAM, as opposed to a regular spherical atom refinement (Bąk *et al.*, 2011 ▸). In principle, TAAM refinement using multipolar parameters obtained from high-resolution diffraction experiments conducted at optimal conditions (*T* ≤ 100 K, ambient pressure) may be justified for structures under pressures up to 10 GPa. In this pressure range, molecular arrangement and intermolecular interactions are affected, whereas the molecular structure (and electron density) can only be considerably impacted at pressures exceeding 10 GPa (Tse, 2020 ▸; Yoo, 2020 ▸). Therefore, we can expect that TAAM refinement with multipolar parameters obtained at ambient pressure should be justified for a limited range of pressures below 10 GPa.

A possible solution for these problems would require a systematic study on a large number of similar diffraction datasets of different resolution and quality. Our recent variable-temperature (VT) and variable-pressure study of a salt of 5,6-di­chloro-2,3-di­cyano­semi­quinone radical anion (DDQ) with 4-cyano-*N*-methyl­pyridinium cation (4-CN) (Bogdanov *et al.*, 2020 ▸), combined with a high-resolution charge density study (Milašinović *et al.*, 2020 ▸), offers a promising material, and deals with a chemically interesting type of interaction.

**Figure 1:**
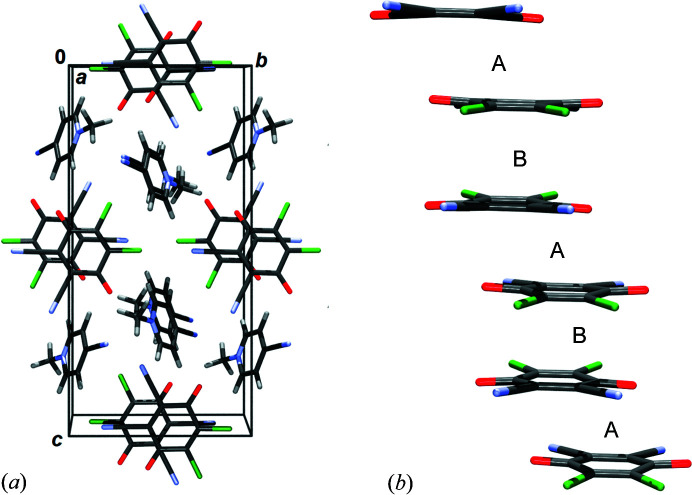
(*a*) Crystal packing of 4-CN·DDQ viewed in the direction [100]. (*b*) Stack of DDQ radical anions in 4-CN·DDQ. Short intra-dimer and long inter-dimer contacts are marked as A (2.92 Å at room temperature and ambient pressure) and B (3.49 Å at room temperature and ambient pressure), respectively. The symmetry operator for contact A is −*x*, −*y*+1, −*z* and for B it is −*x*+1, −*y*+1, −*z*.

The title compound 4-CN·DDQ (Scheme 1) comprises stacks of radicals which involve two-electron multicentre covalent bonding (2e/mc; *i.e.* pancake bonding) (Molčanov *et al.*, 2018*a* ▸; Milašinović *et al.*, 2020 ▸) in dimers of closely bound radicals. Such bonding has caught the attention of researchers over the last decade (Novoa & Miller, 2007 ▸; Preuss, 2014 ▸; Kertesz, 2019 ▸; Molčanov & Kojić-Prodić, 2019 ▸; Molčanov *et al.*, 2019*a* ▸) describing a non-localized electron pair of two closely interacting radicals occupying the same orbital (*i.e.* paired spins). Crystals comprising 2e/mc bonded radicals are therefore diamagnetic and distances between the molecular mean planes are much shorter than the sum of van der Waals radii (usually <3.1 Å). This interesting novel interaction therefore borders inter- and intramolecular and its energy typically exceeds −15 kcal mol^−1^ (Kertesz, 2019 ▸; Molčanov & Kojić-Prodić, 2019 ▸). Crystal packing of 4-CN·DDQ comprises stacks of pancake-bonded radical anions with alternating short (pancake bond; in the text referred to as contact A; symmetry operation −*x*, −*y*+1, −*z*) and long (non-bonding stacking contact; in the text referred to as contact B; symmetry operation −*x*+1, −*y*+1, –*z*) interplanar separations (Fig. 1 ▸).[Chem scheme1] 
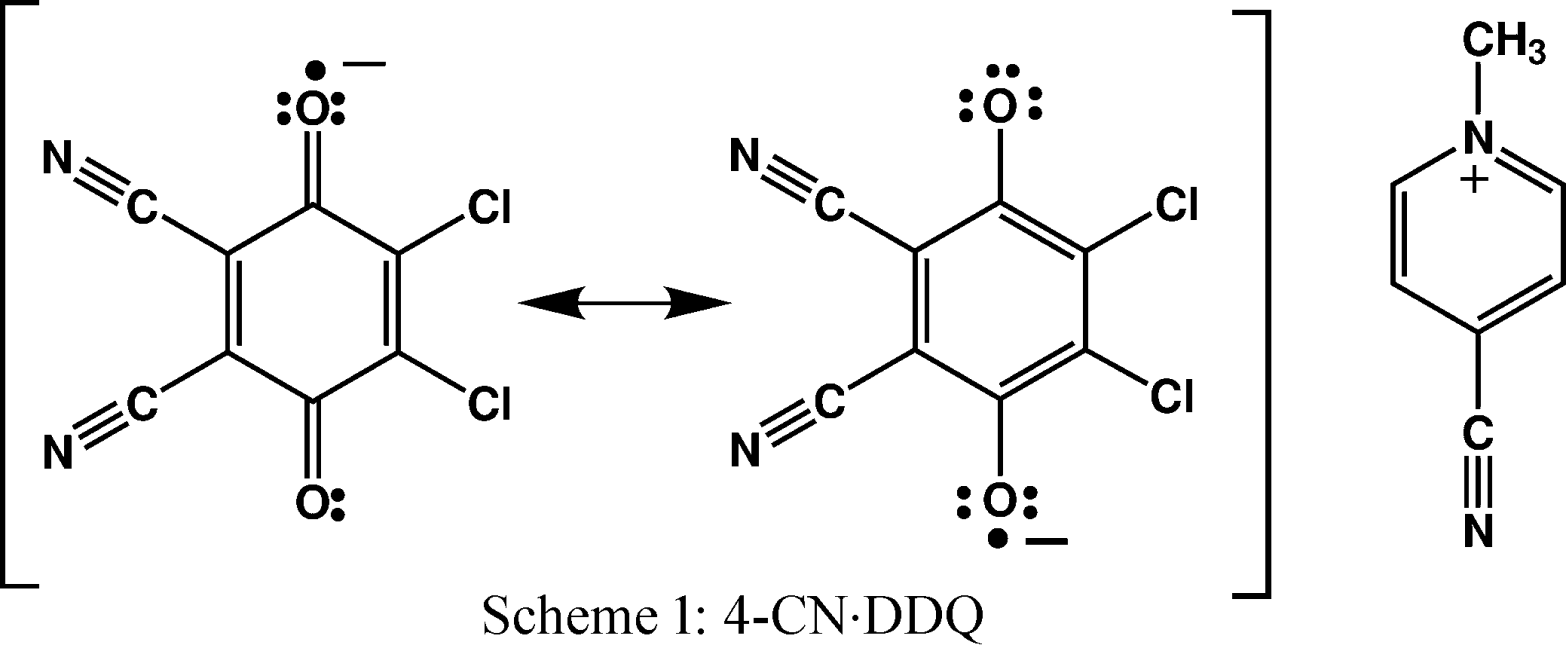


Currently only a few X-ray charge density studies of 2e/mc bonded radicals have been published, dealing with di­aza­dtihiazolyls (Domagała *et al.*, 2014 ▸; Domagała & Haynes, 2016 ▸) and semi­quinones (Molčanov *et al.*, 2018 ▸, 2019*b* ▸; Milašinović *et al.*, 2020 ▸). Due to the fact that the crystals with organic radicals are usually unstable, only the selected stable ones may be used, severely limiting the applicability of the experimental charge density.

In order to gain more information on the behaviour of novel 2e/mc interactions, crystals of pancake-bonded radicals should be studied under a broader range of conditions (temperatures and pressures), which poses additional experimental challenges. Thus data obtained are normally limited to resolutions of 0.8 Å or lower and are thus unsuitable for multipolar refinement and study of charge density. Recently, a combined VT (at ambient pressure) and high-pressure (HP; at room temperature) X-ray diffraction study of 2e/mc bonded radicals was carried out. The first work was performed using 4-CN·DDQ (Bogdanov *et al.*, 2020 ▸). In addition, this compound was studied by ultra-high resolution X-ray crystallography (Milašinović *et al.*, 2020 ▸). A large number of diffraction datasets was measured with varying quality. VT data were of high quality with resolutions of about 0.8 Å, whereas the HP data were generally poor, incomplete and lower resolution. The availability of experimentally determined multipolar charge density parameters makes this compound ideally suited to test the applicability of transferable multipoles. The study of charge density is crucial for understanding the nature of 2e/mc bonding, especially under conditions far from ideal for high-resolution data collection.

One problem here is that 2e/mc bonding involves a non-localized electron pair, meaning that its electron density is low (maximum electron density at the bonding critical points between the radicals is <0.1 e Å^−3^) (Molčanov & Kojić-Prodić, 2019 ▸; Molčanov *et al.*, 2019*a* ▸; Milašinović *et al.*, 2020 ▸). Therefore, its study is less reliable than that of intramolecular covalent bonding which is stronger. To test the reliability of our TAAM models, we propose the following criteria: (*i*) overall reduction of disagreement *R* factors and residual density of TAAM refinement compared with regular spherical refinement; (*ii*) electron densities should follow a clearly defined trend; (*iii*) experimentally obtained electron densities should be in good agreement with theoretical ones.

Concerning the crystallographic criterion (*i*), the crystallographic refinement statistics will improve the most on application of TAAM when thermal displacement parameters are moderate and the resolution limit is high (Zarychta *et al.*, 2007 ▸). However, it is known that reduction of *R* values does not necessarily mean an improvement of the model; there are cases where an incorrect model yields a lower *R* value (Molčanov *et al.*, 2011 ▸; Stilinović & Kaitner, 2010 ▸). Therefore, other tests, such as plots of *F*_calc_ *versus* *F*_obs_, *I*_calc_ *versus* *I*_obs_, fractal dimension plots (Meindl & Henn, 2008 ▸) *etc.* should also be taken into consideration. A possible pitfall is also overrefinement, addition of spurious parameters which typically yields a lower *R*, while not improving the model (Zarychta *et al.*, 2011 ▸, Krause *et al.*, 2017 ▸). However, in the case of TAAM refinement, no additional parameters are added (since transferred multipolar populations are not refined). Therefore reduction of *R* values upon a transfer of multipoles can be considered a genuine improvement of the model, rather than overrefinement.

Criterion (*ii*) determines whether the obtained charge densities make chemical sense and whether they can be interpreted. However, it may also be argued that a trend of intermolecular charge densities may be only an artefact: if the same multipoles are used and the distance between rings increases (as with increasing temperature, Bogdanov *et al.*, 2020 ▸), it is expected that the electron density between the rings should decrease. Therefore, the charge densities should be corroborated by quantum chemical computations (*iii*). Only if there is a good match between experimental and theoretical charge densities, we may claim that the transferred multipole model yielded meaningful results.

## 2. Experimental   

### 2.1. Preparation and X-ray diffraction   

Single crystals of 4-CN·DDQ were prepared as described previously (Molčanov *et al.*, 2018*b* ▸; Bogdanov *et al.*, 2020 ▸). HP diffraction data and VT diffraction data in the range 120–310 K were taken from our previous work (Bogdanov *et al.*, 2020 ▸). The measurements were performed on an Oxford Diffraction Gemini Ultra R CCD diffractometer with Mo radiation equipped with an Oxford Instruments CryoJet liquid nitro­gen cooling device. The program package *CrysAlisPRO* (Rigaku Oxford Diffraction, 2018 ▸) was used for data reduction. HP measurements were performed using an Almax Boehler diamond anvil cell (DAC) (Boehler, 2006 ▸). A stainless-steel gasket with an initial thickness of 200 µm was pre-indented to 100 µm. The ruby fluorescence method was used for pressure calibration (Forman *et al.*, 1972 ▸; Piermarini *et al.*, 1975 ▸). A pentane–iso­pentane mixture (1:1) was used as a hydro­static pressure-transmitting medium (Piermarini *et al.*, 1973 ▸; Zakharov & Achkasov, 2013 ▸). The most disagreeable reflections overlapping with diamond and gasket reflections were excluded from the hkl file manually. Absorption corrections were performed using the *ABSORB*-7 (Angel & Gonzalez-Platas, 2013 ▸) software. The multiple integrated reflections were averaged for the space group *P*2_1_/*c* using *SORTAV* (Blessing, 1987 ▸) adapted to the area detector data.

Single-crystal diffraction experiments for 90, 340 and 370 K were carried out on an Enraf-Nonius CAD4 diffractometer equipped with an Oxford Cryosystems Cryostream Series 700 liquid nitro­gen cooling device. The *WinGX* standard procedure was applied for data reduction (Farrugia, 1997 ▸, 2012 ▸). Three standard reflections were measured every 120 min as an intensity control. Since the compound contains only light atoms, no absorption correction was used.

### 2.2. Building the transferrable multipole model   

The electronic structure of the semi­quinone radical anion differs significantly from similar closed-shell molecules such as neutral quinones and hydro­quinones (Molčanov *et al.*, 2019*b* ▸). Therefore, existing databases of transferrable multipoles such as *ELMAM2* (Domagała *et al.*, 2012 ▸) could not be used; they are all based on closed-shell molecules. Instead, we used multipoles from our recent high-resolution X-ray charge density study of 4-CN·DDQ (Milašinović *et al.*, 2020 ▸). The original multipolar model, refined with a minimum of loose restraints (Milašinović *et al.*, 2020 ▸) was simplified with a reduced number of transferred parameters. Therefore, the charge density was refined using a new set of constraints considering molecular (local) symmetry and chemical equivalence. As can be seen from Scheme 1, the 4-CN cation, which is planar within experimental error, has an approximate symmetry *D*_2h_ when the non-disordered methyl group is disregarded. The DDQ anion is not perfectly planar, but slightly bent by 3.9° (Milašinović *et al.*, 2020 ▸), so its molecular symmetry should be *C*_s_. Multipolar populations of the original high-resolution study (Milašinović *et al.*, 2020 ▸) were symmetrical with respect to the molecular mean plane within one e.s.d., emphasizing the planarity of the electronic structure. Distribution of valence electrons was also planar in our charge density studies of salts of tetra­chloro­semi­quinone radical anion (Molčanov *et al.*, 2018 ▸ *b*, 2019*b* ▸).

**Figure 2:**
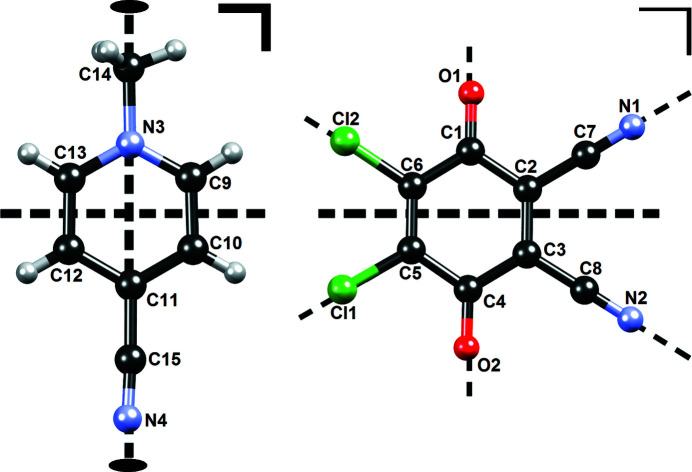
Molecular and local symmetry used to generate constraints for refinement of transferrable multipoles. Molecular symmetry (multipoles and kappas of equivalent atoms constrained to be equal) are shown as thick lines and local symmetries of atomic environments (multipoles constrained to local mirror symmetry) are shown as thin lines.

Thus, sets of atoms equivalent by local symmetry were generated (*e.g.* Cl1 and Cl2, O1 and O2, C9 and C13, *etc.* see Table S4 of the supporting information), whose multipoles and kappas were constrained to be equal. In addition, the local environment of the majority of all atoms, with the exception of the methyl carbon, is planar. Therefore, their multipoles were additionally constrained to planarity (in the case of cyano groups, they were constrained to be cylindrical, see Table S4). Molecular and local symmetries are displayed graphically in Fig. 2 ▸.

Applying these constraints in the charge density refinement using high-resolution data (Milašinović *et al.*, 2020 ▸) resulted in insignificant worsening of disagreement factors and residual densities compared with the original multipolar refinement (Milašinović *et al.*, 2020 ▸) (see Figs. S3 and S4 of the supporting information; Table S3), so the model could be considered valid. The obtained multipolar parameters (Table S5) were exported in the format of ELMAM2 (Domagała *et al.*, 2012 ▸) transferrable parameters, and were subsequently used in refinements of VT and HP structures (see below).

### 2.3. Refinement   

Previously published atomic coordinates and atomic displacement parameters (ADPs) (Bogdanov *et al.*, 2020 ▸) were used as a starting point for TAAM refinement, which was performed using the *MoPro* (Jelsch *et al.*, 2005 ▸) software package. The C—H distances were constrained to the standard bond length derived from neutron diffraction studies (Allen & Bruno, 2010 ▸). An initial Independent Atom Model (IAM) spherical refinement (scaling factors, atomic coordinates and ADPs) was carried out until convergence, and these structures were later used as references. Multipoles were then transferred on the spherical models and refinement (scaling factors, atomic coordinates and ADPs) was repeated until convergence. For refinement of HP structures, a polynomial scaling factor was used (Wenger, 2015 ▸). For VT structures, anisotropic parameters for hydrogen atoms were calculated by the *SHADE3* server (Madsen, 2006 ▸) and imported into the multipolar model; another round of refinement (scaling factors, atomic coordinates and ADPs) was then performed, keeping hydrogen ADPs constrained. The methyl group of the 4-CN cation in structures at 150 and 210 K showed disorder and could be modelled as two positions. The weighting scheme used for VT data was the same for IAM and TAAM refinements and was *W*_*hkl*_ = 1/σ(*I*_*hkl*_).

Geometry and charge-density calculations were performed with *VMoPro* (Jelsch *et al.*, 2005 ▸); molecular graphics were prepared using *MoProViewer* (Guillot, 2012 ▸) and *Mercury* (Macrae *et al.*, 2020 ▸). Crystallographic and refinement data are shown in Tables S1 and S2.

### 2.4. Quantum chemical modelling   

To probe the effects of pressure on molecular orbitals of short intra-dimer contact A (*i.e.* pancake bond) and long inter-dimer contact B, a series of calculations were performed. The *GAUSSIAN16* program package (Frisch *et al.*, 2016 ▸) was used, at the hybrid exchange–correlation functional CAM-B3LYP level (Yanai *et al.*, 2004 ▸) with aug-cc-pvtz basis set. Single-point DFT calculations were carried out to assess the energetic separation of the bonding and antibonding combinations of the two singly occupied molecular orbitals (SOMOs) of the discussed dimers A and B formed between DDQ radicals. The molecular geometries extracted from the pressure-dependent solid-state X-ray diffraction experiments (Bogdanov *et al.*, 2020 ▸) were used. Such an approach ensured that all subtle pressure-induced contraction in the covalent bonds as well as the more substantial compression of the intra- and inter-dimer contacts were taken into account. As recommended by Kertesz (2019 ▸), dispersion correction was also used, we applied the D3 correction by Grimme *et al.* (2010 ▸) in conjunction with the Becke–Johnson damping function.

The nature of intra- and intermolecular interactions by means of deformation density was studied via periodic density functional theory (DFT) calculations performed with *CRYSTAL17* software (Dovesi *et al.*, 2018 ▸). Atomic coordinates were taken either from final experimental multipolar refinement (if available) or from IAM refinement, with no further geometry optimization. The compounds at all given temperatures and under all given pressures were modelled on the PBE0-D3/POB-DZVP theory level (Vilela-Oliveria *et al.*, 2019 ▸). Obtained wavefunctions were further used to carry out the topological analysis of the periodic electron densities, adopting the QTAIM approach (Bader, 1990 ▸) using the* TOPOND14* program (Gatti & Casassa, 2017 ▸), integrated with *CRYSTAL17*.

## 3. Results and discussion   

### 3.1. Evaluation of the transferred-multipole model   

The first and the most important step in testing charge densities obtained by transfer of multipoles is to evaluate whether the models are physically meaningful. For this purpose, the three criteria outlined in the Introduction[Sec sec1] are elaborated here. Since we have two series of datasets with different data qualities, we will analyse them separately. The VT data are of high quality and spherical refinements revealed maxima of residual densities located at midpoints of chemical bonds (see S4), which can be interpreted as bonding valence electron density. However, the HP data were significantly inferior, so their residual densities after spherical refinements contained few, if any, interpretable maxima (see Section S5 of the supporting information).

#### 3.1.1. Variable-temperature data   

**Figure 3:**
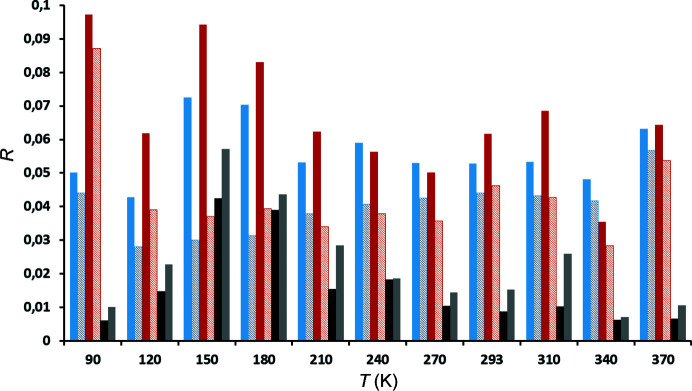
Disagreement factors of VT datasets as a function of temperature, for spherical (dark colour) and TAAM refinements (light colour). *R*(*F*) values are shown in blue (dark: spherical, light: multipolar), w*R*(*F* ^2^) in red (dark: spherical, light: multipolar). Difference *R*(*F*)_spheric_ − *R*(*F*)_TAAM_ is shown in black and *w* *R*(*F* ^2^)_spheric_ − *w* *R*(*F* ^2^)_TAAM_ in grey.

As can be seen from Table S1, TAAM refinement resulted in considerable improvement of all VT datasets (Fig. 3 ▸). On average, *R*(*F*) values improved by 0.016 and w*R*(*F* ^2^) by 0.023. In addition, the distributions of calculated and expected intensities (Figs. S16 and S17) show no significant deviations, further corroborating that the refined models are correct. Fractal dimension plots of residual density (Meindl & Henn, 2008 ▸; Fig. S18) indicate good quality of the refined models. Noticeable deviation from the parabolic shape for datasets collected at 90, 310 and 340 K can be attributed to increased noise and reflect inferior quality of measured data.

Residual electron densities were also considerably improved (Table S1 and Fig. 4 ▸); average reductions of Δρ_max_, Δρ_min_ and Δρ_r.m.s._ are 0.172, 0.081 and 0.008 e Å^−3^, respectively. Residual density maps in mean planes of radical anions and cations are shown in Figs. S5–S15; three selected examples are shown in Fig. 5 ▸. It is therefore obvious that VT data satisfy criterion (*i*).

**Figure 6:**
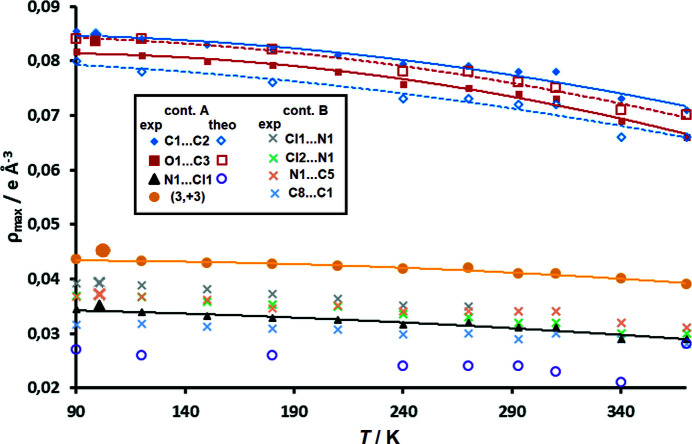
Electron density at (3,−1) CPs between DDQ radicals for VT datasets as a function of temperature. Intra-dimer (contact A) CPs are shown as full symbols (experimental data) and open symbols (theoretical data), and inter-dimer CPs (contact B) as crosses (only experimental data, for clarity). Electron density in the intra-dimer (3,+3) cage CP is displayed as yellow circles. Trend lines drawn for intra-dimer CPs are intended as guides to the eye only (full lines: experimental, dashed lines: theoretical). Data from the high-resolution study (Milašinović *et al.*, 2020 ▸) at 100 K are shown as larger symbols.

To check criterion (*ii*) an AIM analysis of critical points (CPs) had to be performed. Bond lengths are essentially temperature-invariant, so intramolecular CPs did not show any meaningful trend. Electron densities in CPs showed small variance. The standard deviations of electron density in bonding CPs ranges between 0.006 and 0.027 e Å^−3^ for the anion, and between 0.008 and 0.017 e Å^−3^ for the cation. The respective average ρ_CP_ e.s.d.s are 0.011 e Å^−3^ for the anion and 0.015 e Å^−3^ for the cation (see Section S8). The intermolecular CPs are interesting and more sensitive to crystal structure changes with temperature (see Section S9). Here, we limit the analysis only to the zone between the stacked DDQ radicals. Fig. 6 ▸ shows that the electron density between the rings decreases with the temperature increase. This is valid for both inter- and intra-dimer electron density (contacts A and B, respectively).

Experimentally determined intra- and intermolecular CPs were successfully reproduced by periodic DFT computations; all bonding (3,−1) CPs were found and their electron densities appear to match well with the experimental values (see Sections S8 and S9). This shows that criterion (*iii*) has also been satisfied; therefore, we conclude that the TAAM model is valid for crystal structures obtained from good-quality VT data. In addition, there is a good match between electron densities obtained by TAAM and those from a true multipolar refinement (Milašinović *et al.*, 2020 ▸; Fig. 6 ▸).

#### 3.1.2. High-pressure data   

TAAM refinements showed improvement over IAM refinements for datasets up to 3.95 GPa. At higher pressures (corresponding to the HP phase) the quality of data was not sufficient to see a significant lowering of the *R*(*F*) factor. However, the weighted *wR* ^2^(*F*) factor did show an improvement with TAAM refinement. The dataset at 2.55 GPa, which is near the phase transition point, could not be properly refined.

**Figure 7:**
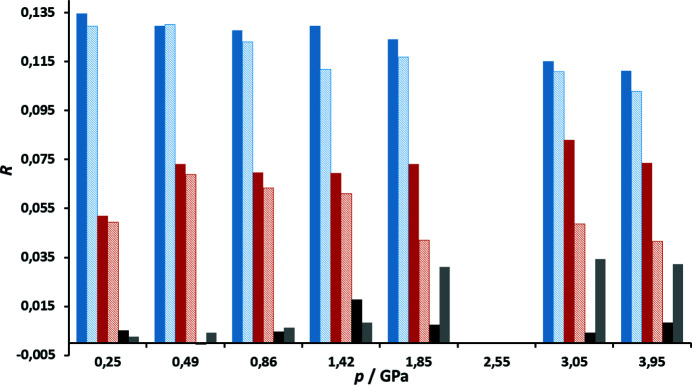
Disagreement factors of HP datasets as a function of pressure, for spherical (dark colour) and TAAM refinements (light colour), *R*(*F*) values are shown in blue (dark: spherical, light: multipolar), and w*R*(*F* ^2^) in red (dark: spherical, light: multipolar). Differences of *R*(*F*)_spheric_ − *R*(*F*)_TAAM_ are shown in black and of w*R*(*F* ^2^)_spheric_ − w*R*(*F* ^2^)_TAAM_ in grey.

**Figure 8:**
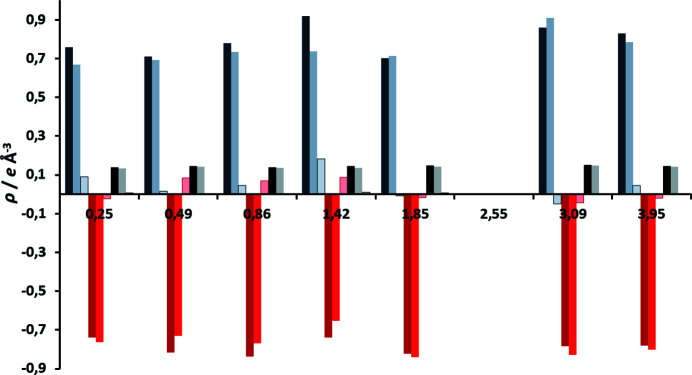
Maximum (blue), minimum (red) and root-mean-square (black/grey) residual densities of HP datasets as functions of pressure, for spherical and TAAM refinements. Dark-coloured bar represents spherical refinement, light-coloured bar represents TAAM refinement, and the lightest-coloured bar with a dark border represents the difference between spherical and multipolar refinements.

Table S2 shows that the improvement of the disagreement factors upon introduction of transferred multipoles is much more modest than for VT data (Fig. 7 ▸). Nevertheless, there is a measurable improvement: average *R*(*F*) values improved by 0.007 and w*R*(*F* ^2^) by 0.030. Distribution of calculated and expected intensities (Figs. S26 and S27) are within acceptable limits, with the exception of the highest-angle reflections. Fractal dimension plots of residual density (Meindl & Henn, 2008 ▸; Fig. S28) are much broader than for VT data due to a higher residual density, but they still retain parabolic shape. Slight ‘shoulders’ can be attributed to noise. This corroborates validity of TAAM refinement for HP data. There was also a modest improvement of residual densities (Table S2, Fig. 8 ▸); average reductions of Δρ_max_, Δρ_min_ and Δρ_r.m.s._ are 0.045, 0.020 and 0.004 e Å^−3^, respectively.

Residual density maps in mean planes of radical anions and cations are shown in Figs. S19–S25; three selected examples are shown in Fig. 9 ▸. We can therefore conclude that the HP data also satisfy criterion (*i*).

Similarly to VT data, molecular geometries are essentially unchanged, so intramolecular CPs did not show a meaningful trend. Maximum electron densities in CPs show a small variance, albeit somewhat larger than for VT data (see Section S10). Standard deviations for chemical bonds in the anion range between 0.018 and 0.053 e Å^−3^ (average 0.036 e Å^−3^) and in the cation range between 0.015 and 0.054 e Å^−3^ (average 0.026 e Å^−3^).

**Figure 10:**
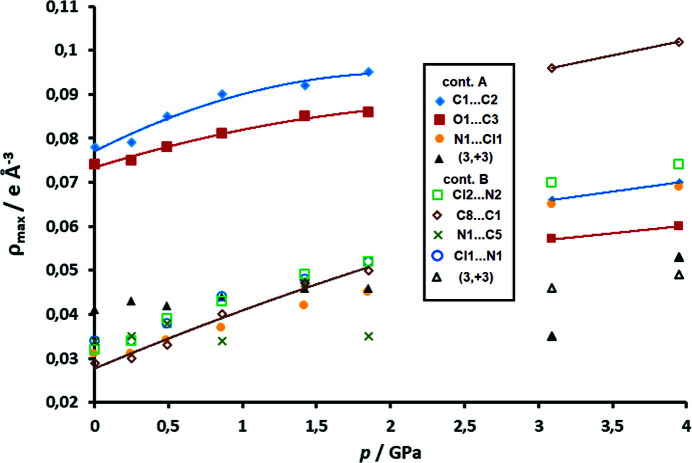
Electron density (e Å^−3^) at experimental (3,−1) CPs between DDQ radicals for HP datasets as a function of pressure (GPa). CPs of contact A are shown (symmetry operation −*x*, −*y*+1, −*z*) as full symbols and those of contact B (symmetry operation −*x*+1, −*y*+1, −*z*) as open symbols. Electron density in the intra-dimer (3,+3) cage CP is displayed as black triangles. Trend lines drawn for some CPs are intended as guides to the eye only.

Analysis of intermolecular CPs (see Section S11) was more complex due to the phase transition at about 2.5 GPa (Bogdanov *et al.*, 2020 ▸). However, note that electron density in dimers (contact A) and between them (contact B) increases monotonically with pressure up to 1.85 GPa. For pressures above the phase transition, there are only two datasets, but they also show an increase of intermolecular electron density (Fig. 10 ▸). Thus, criterion (*ii*) is satisfied.

Agreement between experimental and theoretical charge densities is good both for intra- (see Section S10) and intermolecular contacts (see Section S11): all experimental (3,−1) CPs were also found in theoretical data. In fact, despite the lower quality of diffraction data, agreement of electron densities at CPs is as good as for VT structures, indicating that criterion (*iii*) has also been satisfied.

### 3.2. Nature of 2e/mc bonding between DDQ radical anions   

#### 3.2.1. Evolution of charge density with temperature   

**Figure 11:**
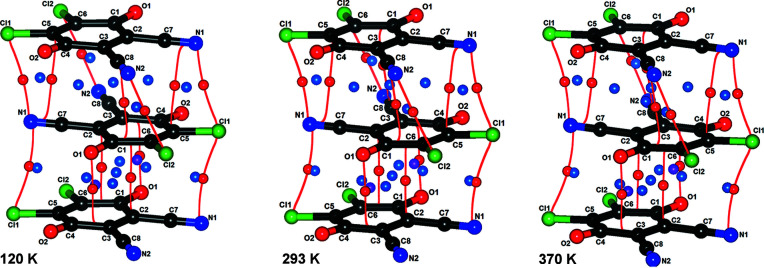
CPs in a stack of DDQ radical anions at three selected temperatures: (*a*) 120 K, (*b*) 293 K and (*c*) 370 K. Weaker inter-dimer contact B is above and intra-dimer contact A (2e/mc bond) is below. (3,−1) CPs are shown as red spheres, (3,+1) as blue spheres and (3,+3) cage CPs as purple spheres; intermolecular bond paths are shown as red lines.

The topology of electron density between the anion rings shows essentially no major geometrical difference in the range 90–370 K; all bond paths and positions of CPs are conserved (Fig. 11 ▸ and Section S9) and can also be found in our previous high-resolution study (Milašinović *et al.*, 2020 ▸). Electron density on the CPs between the rings decreases with temperature, as is commonly observed for the intermolecular interactions. The crystal unit cell usually expands and interactions become weaker with rising temperature (Chang, 2000 ▸; Bogdanov *et al.*, 2020 ▸). Fig. 6 ▸ indicates this nicely: the CP with the highest electron density in the short intra-dimer contact A (*i.e.* pancake bond) has ρ_CP_ falling from 0.085 e Å^−3^ at 90 K to 0.071 e Å^−3^ at 370 K, which is a reduction of about 15%. Data obtained from our previous high-resolution study at 100 K (Milašinović *et al.*, 2020 ▸) fit into this trend nicely (in Fig. 6 ▸ they are shown as larger symbols). Other CPs have a reduction of 5.5–16%, including also the cage minimum.

Electron density of the weak inter-dimer contact B CPs is also reduced to a similar extent (in relative value), and the reduction is within a 10–21% range.

#### 3.2.2. Evolution of charge density with pressure   

**Figure 12:**
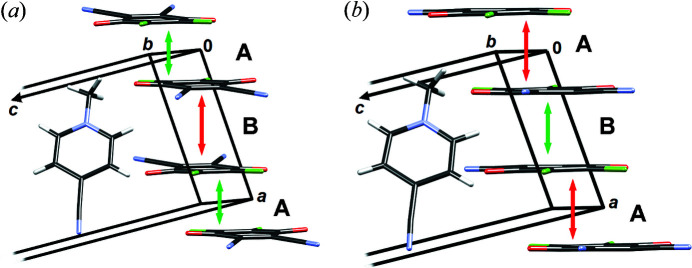
Crystal packing at (*a*) ambient pressure and (*b*) 6.00 GPa. Contacts A and B are marked, whereas shorter and longer interplanar separations are indicated by green and red arrows, respectively.

The HP data reveal two phases with different arrangements of long and short contacts between the radicals (Fig. 12 ▸). At 2.55 GPa, near the phase transition point, the radicals are almost equidistant (*i.e.* contacts A and B are almost equal).

**Figure 14:**
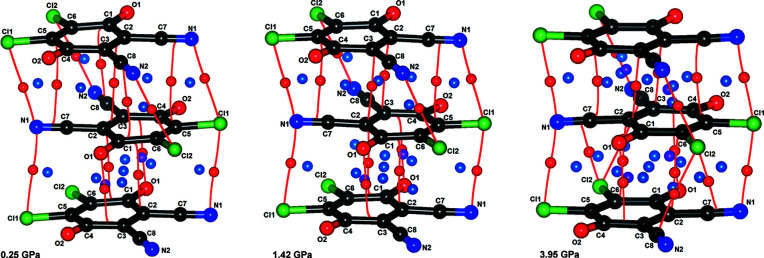
CPs in a stack of DDQ radical anions at three selected pressures: (*a*) 0.25 GPa, (*b*) 1.42 GPa and (*c*) 3.95 GPa. Contact B is above and contact A is below. (3,−1) CPs are shown as red spheres, (3,+1) as blue spheres and (3,+3) as purple spheres; intermolecular bond paths are shown as red lines.

From ambient pressure to 1.85 GPa, the stacks remain the same, with alternating short (pancake bonding) and long (non-bonding) contacts, despite compression. Therefore, monotonic increase of electron density between the rings (Figs. 10 ▸ and 13 ▸) is a result of shorter intermolecular distances, which are, in turn, a result of increasing pressure. The maximum electron density in the pancake bond (contact A) increases from 0.078 e Å^−3^ at ambient pressure to 0.095 e Å^−3^ at 1.85 GPa, which is an increase of 21%. Other CPs follow a similar trend. The positions of the CPs change very little, as can be seen from Fig. 14 ▸ (and also Section S11). The weak inter-dimer contact B, which is a non-bonding contact, does not display any cage CP which would be associated with the existence of pancake bonding (Milašinović *et al.*, 2020 ▸). The absence of a cage CP may arise from the large antiparallel displacement; the C_6_ ring is so offset that the projection of its centroid on the other ring plane falls out of the ring perimeter [Fig. 1 ▸(*b*)].

The increase with pressure of the electron density in weak contact B is, however, much more pronounced and exceeds 50% before the phase transition. This is consistent with a sharper decrease of intermolecular distances and also with increasing covalent (*i.e.* 2e/mc bonding) character of the interaction. A slight re-arrangement of the CPs can be noted (Fig. 14 ▸, Section S11). However the highest electron density at the CP is 0.050 e Å^−3^ indicating that this still remains a non-bonding contact.

Near the phase transition point (2.55 GPa), the diffraction data were rather poor and TAAM refinement yielded no improvement; however, theoretical data are available and can fill this gap. Electron densities in (3,−1) CPs at 2.55 GPa are similar for both contacts and are in the range 0.05–0.07 e Å^−3^. An exception is the contact Cl1⋯N1 which rises to 0.105 e Å^−3^.

The situation becomes more complex and interesting at pressures above the phase transition. The long and short contacts are interchanged (Fig. 12 ▸ and Section S14). Contact B (symmetry operation −*x*+1, −*y*+1, −*z*), which was previously the long contact, becomes shorter, while A (the previously short one, symmetry operation −*x*, −*y*+1, −*z*) is elongated. Upon phase transition (*p* = 1.85 to 3.95 GPa), there is a marked jump in the ρ_CP_ electron density of the B contact C8⋯C1 from 0.050 to 0.102 e Å^−3^; in contact A, the highest ρ_CP_ (C1⋯C2) falls from 0.095 to 0.070 e Å^−3^. Theoretical electron densities at 6.00 GPa rise to 0.113 e Å^−3^ and 0.094 e Å^−3^ for contacts B and A, respectively. However, electron densities in both of these contacts are consistent with existence of 2e/mc bonding, and (3,+3) CPs can be observed in both contacts (Fig. 14 ▸, Section S11). We conclude that the HP phase comprises two 2e/mc bonds, one of which is stronger. This confirms our previous tentative conclusion that the stacks of DDQ radicals may here be regarded as pancake-bonded polymers (Bogdanov *et al.*, 2020 ▸).

**Figure 15:**
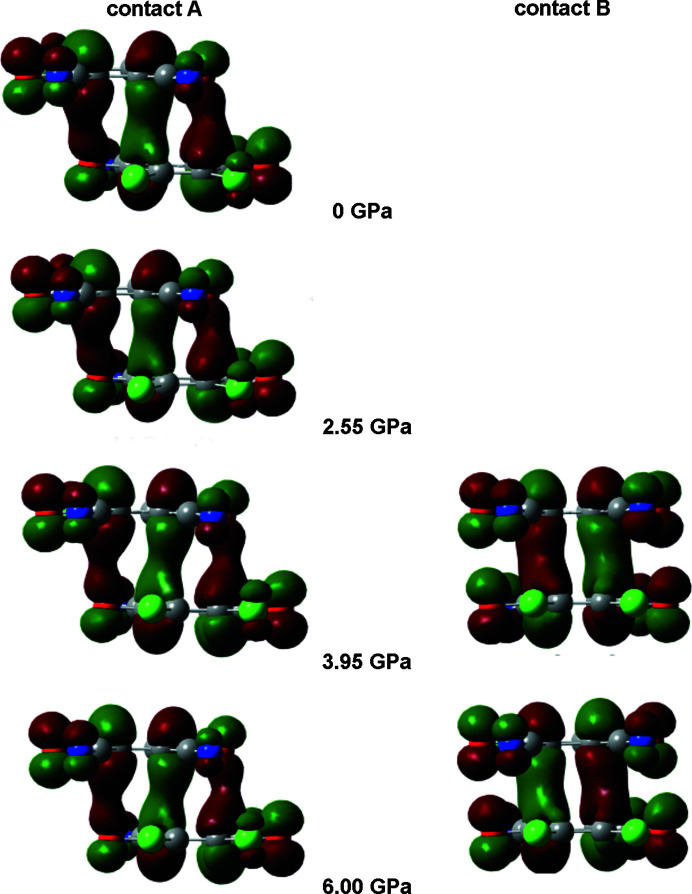
HOMO orbitals calculated for a pair of radicals at selected pressures: left is contact A and right is contact B.

**Figure 16:**
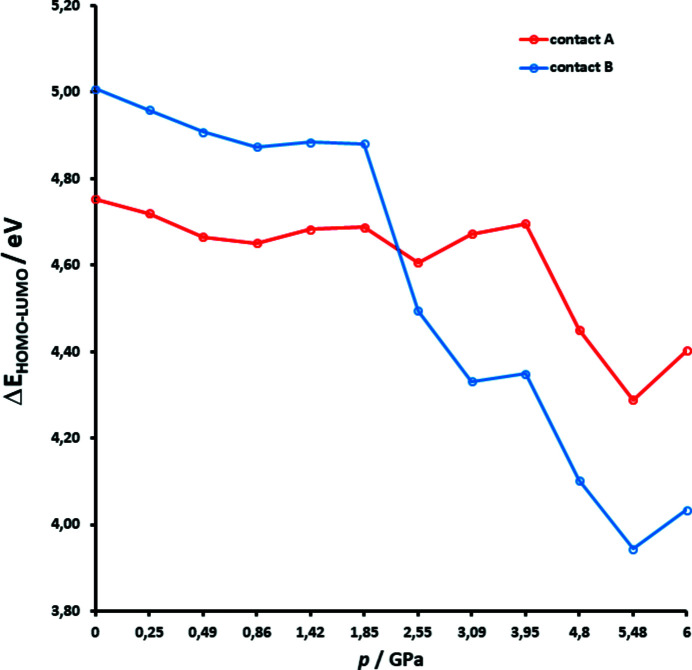
HOMO–LUMO gap between DDQ radicals presented as a function of pressure.

Calculated highest-occupied molecular orbitals (HOMOs) (Fig. 15 ▸ and Section S12; calculated for a pair of rings in singlet configuration) in contact A extend between two rings, similar to those in dimers of tetra­chloro­semi­quinone radical anions (Molčanov *et al.*, 2019*a* ▸). However, HOMOs in contact B at pressures of 2.55 GPa and higher also span between two rings (Fig. 15 ▸, Section S12), further supporting the existence of pancake-bonded polymers. The HOMO–LUMO (lowest-unoccupied molecular orbital) energy gap (Fig. 16 ▸) is reduced as pressure increases, but falls more rapidly for contact B than for contact A, especially at pressures above 2.55 GPa. This is partly due to the shortened distance between the rings in contact B (Bogdanov *et al.*, 2020 ▸), but also the increased covalent character of the interaction.

## 4. Conclusions   

For the first time, we have described charge density in 2e/mc bonds (pancake bonds) at high temperatures (up to 370 K) and high pressures (up to 3.95 GPa). At 3.09 GPa, all contacts between DDQ radicals involve electron densities exceeding 0.050 e Å^−3^ and (3,+3) cage CPs are present in both contacts A and B. We have previously defined these features as indicative of the presence of 2e/mc bonding (Molčanov & Kojić-Prodić, 2019 ▸; Molčanov *et al.*, 2019*a* ▸; Milašinović *et al.*, 2020 ▸). Therefore, we may conclude that, at pressures of 3.09 GPa and above, the topology of electron density indicates the presence of 2e/mc bonding throughout the stack of DDQ radical anions. This conclusion is corroborated with periodic DFT computations. Thus, we have proved our previous speculation (based on geometrical data, only) on the existence of pancake-bonded polymer-like structures at high pressures (Bogdanov *et al.*, 2020 ▸).

We have applied electron density transferability to model charge densities from X-ray diffraction data measured at VT and/or HP, which are not sufficient to allow multipolar refinement. Overall reduction of disagreement *R* factors and residual density were achieved compared with regular spherical IAM refinement.

Transferred multipoles from a previously determined charge density at cryogenic temperature were employed to analyse the topology (critical point analysis) of electron density and it led to clear trends with increasing temperature. The electron density values at the CPs of the two stacking interactions show congruent trends with the theoretical results. Our data show that, even weak intermolecular interactions, very sensitive to data quality, can be studied by this method.

We believe that this approach may be applied to study the nature of different types of chemical bonding and intermolecular interactions.

The present results indicate that an increased electric conductivity at pressures above 2.55 GPa should be expected, as well as a change of magnetic properties from diamagnetism to antiferromagnetism. Therefore measurements of magnetism and electric conductivity under HP are planned.

## Supplementary Material

Crystal structure: contains datablock(s) 90K, 120K, 150K, 180K, 210K, 240K, 270K, 293K, 310K, 340K, 370K, 0.25GPa, 0.49GPa, 0.86GPa, 1.42GPa, 1.85GPa, 3.09GPa, 3.95GPa. DOI: 10.1107/S2052252521005273/lq5037sup1.cif

Structure factors: contains datablock(s) 90K. DOI: 10.1107/S2052252521005273/lq5037sup2.hkl

Structure factors: contains datablock(s) 120K. DOI: 10.1107/S2052252521005273/lq5037sup3.hkl

Structure factors: contains datablock(s) 150K. DOI: 10.1107/S2052252521005273/lq5037sup4.hkl

Structure factors: contains datablock(s) 180K. DOI: 10.1107/S2052252521005273/lq5037sup5.hkl

Structure factors: contains datablock(s) 210K. DOI: 10.1107/S2052252521005273/lq5037sup6.hkl

Structure factors: contains datablock(s) 240K. DOI: 10.1107/S2052252521005273/lq5037sup7.hkl

Structure factors: contains datablock(s) 270K. DOI: 10.1107/S2052252521005273/lq5037sup8.hkl

Structure factors: contains datablock(s) 293K. DOI: 10.1107/S2052252521005273/lq5037sup9.hkl

Structure factors: contains datablock(s) 310K. DOI: 10.1107/S2052252521005273/lq5037sup10.hkl

Structure factors: contains datablock(s) 340K. DOI: 10.1107/S2052252521005273/lq5037sup11.hkl

Structure factors: contains datablock(s) 370K. DOI: 10.1107/S2052252521005273/lq5037sup12.hkl

Structure factors: contains datablock(s) 0.25GPa. DOI: 10.1107/S2052252521005273/lq5037sup13.hkl

Structure factors: contains datablock(s) 0.49GPa. DOI: 10.1107/S2052252521005273/lq5037sup14.hkl

Structure factors: contains datablock(s) 0.86GPa. DOI: 10.1107/S2052252521005273/lq5037sup15.hkl

Structure factors: contains datablock(s) 1.42GPa. DOI: 10.1107/S2052252521005273/lq5037sup16.hkl

Structure factors: contains datablock(s) 1.85GPa. DOI: 10.1107/S2052252521005273/lq5037sup17.hkl

Structure factors: contains datablock(s) 3.09GPa. DOI: 10.1107/S2052252521005273/lq5037sup18.hkl

Structure factors: contains datablock(s) 3.95GPa. DOI: 10.1107/S2052252521005273/lq5037sup19.hkl

Details on refinement and charge density. DOI: 10.1107/S2052252521005273/lq5037sup20.pdf

CCDC references: 2084435, 2084436, 2084437, 2084438, 2084439, 2084440, 2084441, 2084442, 2084443, 2084444, 2084445, 2084446, 2084447, 2084448, 2084449, 2084450, 2084451, 2084452

## Figures and Tables

**Figure 4:**
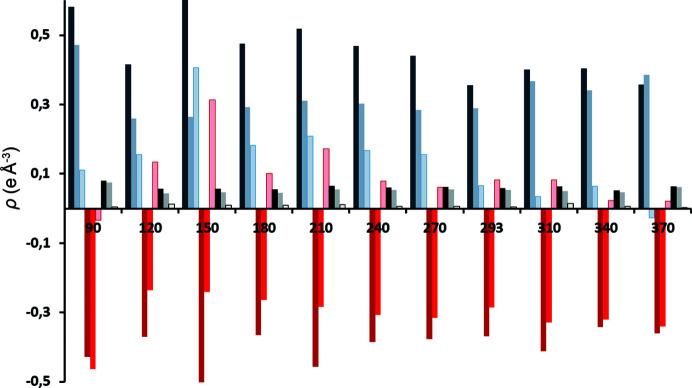
Maximum (blue), minimum (red) and root-mean-square (black/grey) residual densities of VT datasets as functions of temperature, for spherical and TAAM refinements. The dark-coloured bar represents spherical refinement, the light-coloured bar represents TAAM refinement, and the lightest-coloured bar with a dark border represents the difference between spherical and TAAM refinements.

**Figure 5:**
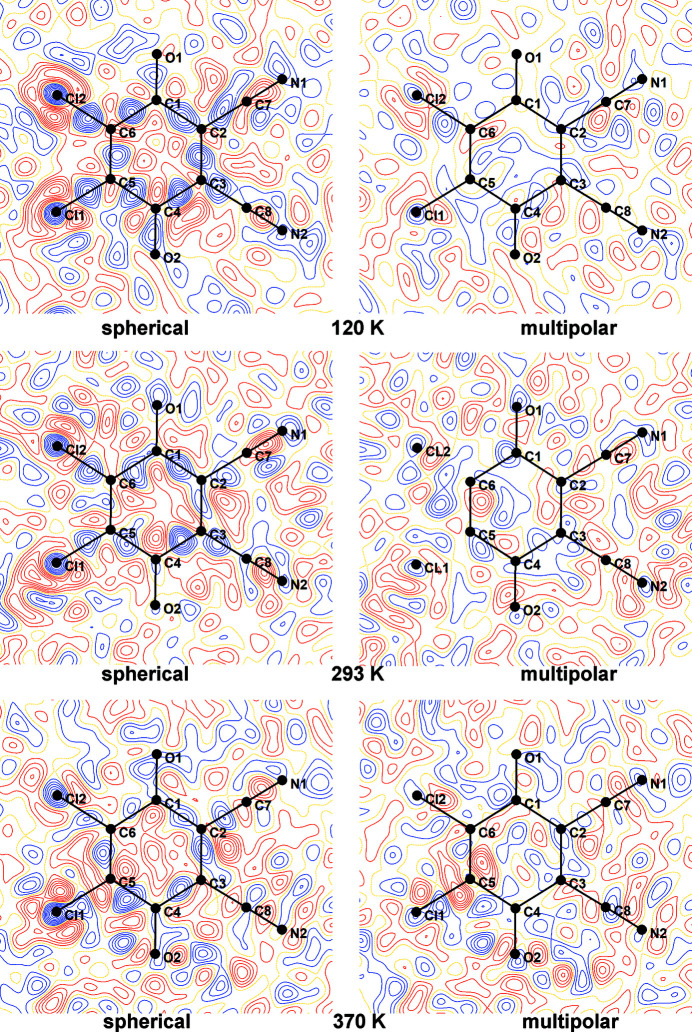
Residual densities for three selected temperatures (top: 120 K, middle: 293 K, bottom: 370 K) in mean planes of the DDQ radical anion for spherical (left) and TAAM refinements (right). Positive density is shown in blue and negative in red; yellow dotted lines represent zero density. Contours are drawn for 0.05 e Å^−1^.

**Figure 9:**
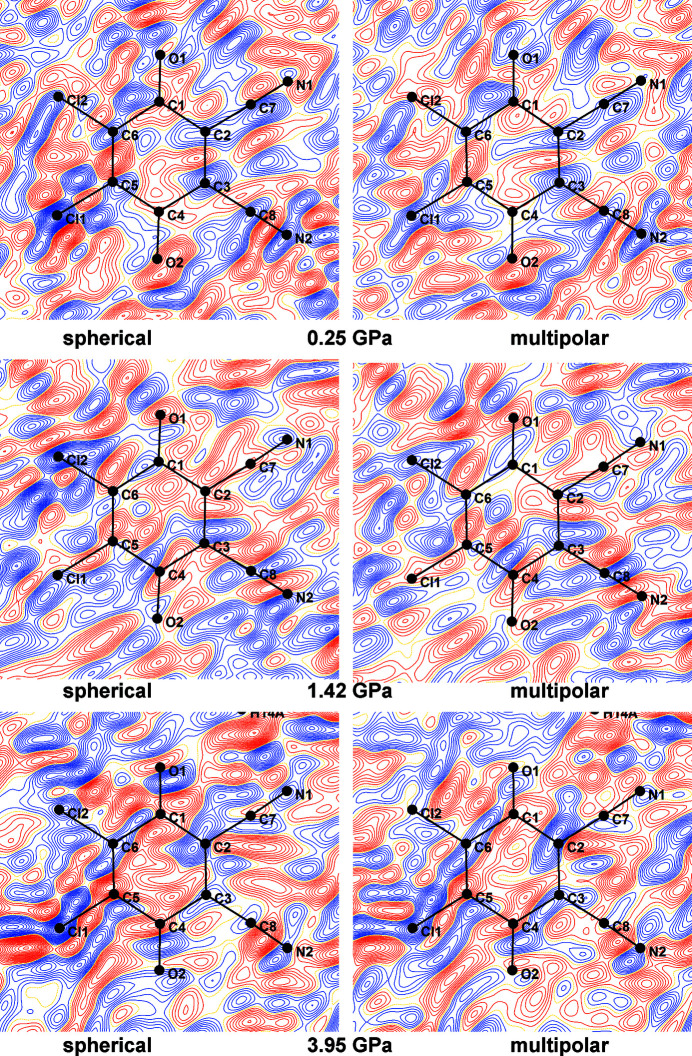
Residual densities for three selected pressures (top: 0.25 GPa, middle: 1.42 GPa, bottom: 3.95 GPa) in mean planes of the DDQ radical anion for spherical (left) and TAAM refinements (right). Positive density is shown in blue and negative in red; yellow dotted lines represent zero density. Contours are drawn for 0.05 e Å^−1^.

**Figure 13:**
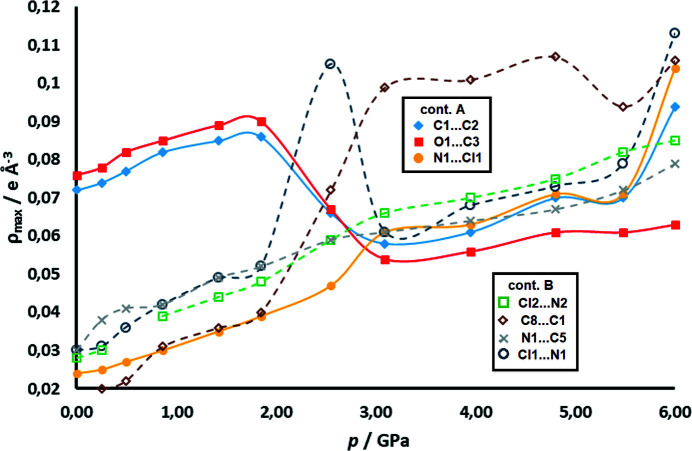
Electron density at (3,−1) CPs between DDQ radicals for theoretical HP data as a function of pressure: CPs of contact A (symmetry operation −*x*, −*y*+1, −*z*) are shown as full symbols and those of contact B (symmetry operation −*x*+1, −*y*+1, −*z*) as open symbols. Trend lines drawn for a number of CPs (full lines for contact A and dashed lines for contact B) are intended as guides to the eye only.
