# Supplementary material for: Charge density studies of multicentre two-electron bonding of an anion radical at non-ambient temperature and pressure
Source: IUCrJ. 2021 Jun 12;8(Pt 4):644–54. doi: 10.1107/S2052252521005273 (PMC8256703; doi:10.1107/S2052252521005273)
Supplement: Supplementary file 20 [file m-08-00644-sup20.pdf]

IUCrJ

Volume 8 (2021)

Supporting information for article:

Charge density studies of multicentre two-electron bonding of an anion radical at non-ambient temperature and pressure

Valentina Milašinović, Krešimir Molčanov, Anna Krawczuk, Nikita E. Bogdanov, Boris A. Zakharov, Elena V. Boldyreva, Christian Jelsch and Biserka Kojić-Prodić

CONTENTS

S1 Experimental details: tables with crystallographic, data collection and refinement details; ORTEP diagrams after transferred-multipole refinement

S2 Generation of a set of transferrable multipoles: data on refinement (including a list of constraints and restraints used) and residual densities

S3 List of transferrable multipoles

S4 Residual densities at variable temperatures (spherical and multipolar), plots I_{obs} *vs*. I_{calc}, plots Y_{obs} *vs*. Y_{calc} and fractal dimension plots

S5 Residual densities at high pressures (spherical and multipolar), plots I_{obs} *vs*. I_{calc}, plots Y_{obs} *vs*. Y_{calc} and fractal dimension plots

S6 Maps of deformation density and Laplacian at variable temperatures

S7 Maps of deformation density and Laplacian at high pressures

S8 Intramolecular critical points at variable temperatures

S9 Intermolecular critical points at variable temperatures

S10 Intramolecular critical points at high pressures

S11 Intermolecular critical points at high pressures

S12 Calculated HOMO and LUMO orbitals at high pressures

S12 Calculated HOMO and LUMO orbitals at high pressures

S13 Geometric parameters of stacking at variable temperatures

S14 Geometric parameters of stacking at high pressures

S15 References

1 Experimental details: tables with crystallographic, data collection and refinement details; ORTEP diagrams after transferred-multipole refinement

Table S1 Crystallographic, data collection and refinement data for variable-temperature X-ray diffraction.

Compound	**90K**	**120K**	**150K**	**180K**	**210K**	**240K**
Empirical formula	$C_{15}H_7Cl_2N_4O_2$	$C_{15}H_7Cl_2N_4O_2$	$C_{15}H_7Cl_2N_4O_2$	$C_{15}H_7Cl_2N_4O_2$	$C_{15}H_7Cl_2N_4O_2$	$C_{15}H_7Cl_2N_4O_2$
Formula wt. / g mol^{-1}	346.15	346.15	346.15	346.15	346.15	346.15
Colour	black	black	black	black	black	black
Crystal dimensions / mm	0.32 x 0.26 x 0.20	0.40 x 0.15 x 0.07	0.40 x 0.15 x 0.07	0.40 x 0.15 x 0.07	0.40 x 0.15 x 0.07	0.40 x 0.15 x 0.07
Space group	$P\,2_1/n$	$P\,2_1/n$	$P\,2_1/n$	$P\,2_1/n$	$P\,2_1/n$	$P\,2_1/n$
a / Å	6.7493(11)	6.75559(15)	6.7832(1)	6.78877(15)	6.8159(1)	6.83004(15)
b / Å	10.0720(14)	10.0647(2)	10.0779(1)	10.07003(19)	10.0882(1)	10.08887(19)
c / Å	20.462(6)	20.4519(4)	20.4867(2)	20.4706(4)	20.5143(2)	20.5119(4)
α / °	90	90	90	90	90	90
β / °	94.781(19)	94.8065 19)	94.754(1)	94.7503(18)	94.740(1)	94.7341(18)
γ / °	90	90	90	90	90	90
Z	4	4	4	4	4	4
V / Å^3	1386.2(5)	1385.70(5)	1395.66(3)	1394.62(5)	1405.74(3)	1408.60(5)
D_{calc} / g cm^{-3}	1.659	1.655	1.648	1.643	1.636	1.625
λ / Å	0.71073 (Mo$K\alpha$)	0.71073 (Mo$K\alpha$)	0.71073 (Mo$K\alpha$)	0.71073 (Mo$K\alpha$)	0.71073 (Mo$K\alpha$)	0.71073 (Mo$K\alpha$)
μ / mm^{-1}	0.484	0.484	0.482	0.480	0.480	0.480
Θ range / °	2.00 – 24.97	2.0 – 28.1	2.0 – 28.0	2.0 – 27.9	2.0 – 28.2	2.0 – 27.8
T / K	90(2)	120.0(1)	149.9(2)	180.0(2)	210(1)	240.0(2)
Diffractometer type	CAD4	Gemini Ultra	Gemini Ultra	Gemini Ultra	Gemini Ultra	Gemini Ultra
Range of h, k, l	$0 < h < 8$; $0 < k < 11$; $-24 < l < 24$	$-8 < h < 7$; $-13 < k < 12$; $-26 < l < 27$	$-7 < h < 8$; $-10 < k < 12$; $-25 < l < 25$	$-8 < h < 7$; $-13 < k < 12$; $-26 < l < 27$	$-5 < h < 8$; $-12 < k < 12$; $-25 < l < 25$	$-7 < h < 9$; $-13 < k < 12$; $-26 < l < 26$
Reflections collected	2642	9214	12481	9309	12507	9373
Independent reflections	2420	2815	2828	2887	3147	3218
Observed reflections ($I \geq 2\sigma$)	2297	2682	2828	2822	3147	3176
Absorption correction	None	Multi-scan	Multi-scan	Multi-scan	Multi-scan	Multi-scan
T_{min}, T_{max}	-	0.928; 1.000	0.928; 1.000	0.927; 1.000	0.930; 1.000	0.953; 1.000
R_{int}	0.0576	0.028	0.0213	0.034	0.0212	0.028
Spherical refinement						
$R\ (F)$	0.0501	0.0403	0.0726	0.0431	0.0532	0.0509
$R_w\ (F^2)$	0.0960	0.0603	0.0932	0.0537	0.0624	0.0416
Goodness of fit	1.272	1.083	2.644	1.766	1.860	1.519
H atom treatment	Restrained isotropic	Restrained isotropic	Restrained isotropic	Restrained isotropic	Restrained isotropic	Restrained isotropic
No. of parameters	229	229	229	229	229	229
$\Delta\rho_{max}$, $\Delta\rho_{min}$, $\Delta\rho_{rms}$, (eÅ^{-3})	0.582; -0.429; 0.080	0.416; -0.370; 0.056	0.670; -0.534; 0.060	0.475; -0.364; 0.055	0.519; -0.456; 0.065	-0.469; -0.385; 0.060
Multipolar refinement						
$R\ (F)$	0.0441	0.0280	0.0301	0.0314	0.0377	0.0411
$R_w\ (F^2)$	0.0863	0.0374	0.0371	0.0381	0.0340	0.0357
Goodness of fit	1.143	1.083	0.982	1.271	0.940	1.112
H atom treatment	Restrained	Restrained	Restrained	Restrained	Restrained	Restrained

	anisotropic	anisotropic	anisotropic	anisotropic	anisotropic	anisotropic
No. of parameters	238	238	238	238	238	238
$\Delta\rho_{max}$, $\Delta\rho_{min}$, $\Delta\rho_{rms}$, (eÅ^{-3})	0.472; -0.463; 0.0075	0.259; -0.236; 0.044	0.264; -0.240; 0.046	0.292; -0.263; 0.045	0.310; -0.283; 0.054	0.302; -0.307; 0.054

Table S1 Cont'd.

Compound	**270K**	**293K**	**310K**	**340K**	**370K**
Empirical formula	$C_{15}H_7Cl_2N_4O_2$	$C_{15}H_7Cl_2N_4O_2$	$C_{15}H_7Cl_2N_4O_2$	$C_{15}H_7Cl_2N_4O_2$	$C_{15}H_7Cl_2N_4O_2$
Formula wt. / g mol^{-1}	346.15	346.15	346.15	346.15	346.15
Colour	black	black	black	black	black
Crystal dimensions / mm	0.40 x 0.15 x 0.07	0.40 x 0.15 x 0.07	0.40 x 0.15 x 0.07	0.30 x 0.08 x 0.07	0.30 x 0.08 x 0.07
Space group	$P\,2_1/n$	$P\,2_1/n$	$P\,2_1/n$	$P\,2_1/n$	$P\,2_1/n$
a / Å	6.84899(17)	6.86364(18)	6.87524(18)	6.9337(8)	6.9626(13)
b / Å	10.0928(2)	10.0969(2)	10.1004(2)	6.9337(8)	10.1312(9)
c / Å	20.5226(4)	20.5284(4)	20.5391(4)	20.599(2)	20.609(2)
α / °	90	90	90	90	90
β / °	94.715(2)	94.703(2)	94.709(2)	94.713(9)	94.7000
γ / °	90	90	90	90	90
Z	4	4	4	4	4
V / Å^3	1413.83(5)	1417.86(5)	1421.47 (6)	1440.8(2)	1448.9(3)
D_{calc} / g cm^{-3}	1.623	1.617	1.604	1.596	1.587
λ / Å	0.71073 (Mo$K\alpha$)	0.71073 (Mo$K\alpha$)	0.71073 (Mo$K\alpha$)	0.71073 (Mo$K\alpha$)	0.71073 (Mo$K\alpha$)
μ / mm^{-1}	0.47	0.47	0.47	0.465	0.463
Θ range / °	2.0 – 27.8	2.0 – 27.3	2.0 – 27.3	1.98 – 24.98	1.98 – 25.24
T / K	270.0(5)	293.0(5)	310.0(5)	340.0(2)	370.0(2)
Diffractometer type	Gemini Ultra	Gemini Ultra	Gemini Ultra	CAD4	CAD4
Range of h, k, l	$-7 < h < 9$; $-13 < k < 12$; $-26 < l < 26$	$–9 < h < 7$; $–13 < k < 12$; $–26 < l < 27$	$–9 < h < 7$; $–13 < k < 12$; $–26 < l < 27$	$0 < h < 8$; $-12 < k < 0$; $-24 < l < 24$	$-8 < h < 0$; $-12 < k < 0$; $-24 < l < 24$
Reflections collected	9414	9438	9454	3078	3031
Independent reflections	3225	3235	3240	2530	2790
Observed reflections ($I \geq 2\sigma$)	3152	3131	2478	2325	2389
Absorption correction	Multi-scan	Multi-scan	Multi-scan	None	None
T_{min}, T_{max}	0.926; 1.000	0.956, 1.000	0.935, 1.000	-	-
R_{int}	0.028	0.029	0.028	0.141	0.026
Spherical refinement					
R (F)	0.0530	0.0529	0.0534	0.0481	0.0632
R_w (F^2)	0.0479	0.0578	0.0638	0.0339	0.0626
Goodness of fit	1.479	1.109	1.185	0.511	1.594
H atom treatment	Restrained isotropic	Restrained isotropic	Restrained isotropic	Restrained isotropic	Restrained isotropic
No. of parameters	229	229	229	229	229
$\Delta\rho_{max}$, $\Delta\rho_{min}$, $\Delta\rho_{rms}$, (eÅ^{-3})	0.440; -0.376; 0.061	0.356; -0.369; 0.059	0.400; -0.412; 0.064	0.404; -0.345; 0.052	0.357; -0.361; 0.064
Multipolar refinement					
R (F)	0.0428	0.0441	0.0432	0.0419	0.0569
R_w (F^2)	0.0338	0.0414	0.0384	0.0267	0.0518
Goodness of fit	1.064	0.832	0.840	0.408	1.328
H atom treatment	Restrained anisotropic	Restrained anisotropic	Restrained anisotropic	Restrained anisotropic	Restrained anisotropic
No. of parameters	238	238	238	238	238
$\Delta\rho_{max}$, $\Delta\rho_{min}$, $\Delta\rho_{rms}$, (eÅ^{-3})	0.283; -0.315; 0.055	0.289; -0.286; 0.053	0.367; -0.329; 0.050	0.340; -0.320; 0.047	0.385; -0.349; 0.061

Table S2 Crystallographic, data collection and refinement data for high-pressure X-ray diffraction.

Compound	**0.25 GPa**	**0.49 GPa**	**0.86 GPa**	**1.42 GPa**	**1.85 GPa**
Empirical formula	$C_{15}H_7Cl_2N_4O_2$	$C_{15}H_7Cl_2N_4O_2$	$C_{15}H_7Cl_2N_4O_2$	$C_{15}H_7Cl_2N_4O_2$	$C_{15}H_7Cl_2N_4O_2$
Formula wt. / g mol^{-1}	346.15	346.15	346.15	346.15	346.15
Colour	black	black	black	black	black
Crystal dimensions / mm	0.25 x 0.10 x 0.05	0.25 x 0.10 x 0.05	0.25 x 0.10 x 0.05	0.25 x 0.10 x 0.05	0.25 x 0.10 x 0.05
Space group	$P\,2_1/n$	$P\,2_1/n$	$P\,2_1/n$	$P\,2_1/n$	$P\,2_1/n$
a / Å	6.8312(4)	6.7445(3)	6.6277(4)	6.5155(3)	6.4387(2)
b / Å	10.0839(16)	10.0486(12)	10.0050(17)	9.9444(12)	9.9135(11)
c / Å	20.5232(15)	20.4539(11)	20.3418(16)	20.2293(11)	20.1349(10)
α / °	90	90	90	90	90
β / °	94.635(6)	94.589(4)	94.609(5)	94.662(4)	94.729(3)
γ / °	90	90	90	90	90
Z	4	4	4	4	4
V / Å^3	1409.1(3)	1381.77(19)	1344.5(3)	1306.37(18)	1280.83(16)
D_{calc} / g cm^{-3}	1.632	1.664	1.710	1.760	1.795
λ / Å	0.71073 (Mo$K\alpha$)	0.71073 (Mo$K\alpha$)	0.71073 (Mo$K\alpha$)	0.71073 (Mo$K\alpha$)	0.71073 (Mo$K\alpha$)
μ / mm^{-1}	0.476	0.485	0.499	0.513	0.523
Θ range / °	2.0 – 22.5	1.998 – 25.4	2.009 – 26.4	2.020 – 26.6	2.030 – 26.7
T / K	293(2)	293(2)	293(2)	293(2)	293(2)
Diffractometer type	Gemini Ultra	Gemini Ultra	Gemini Ultra	Gemini Ultra	Gemini Ultra
Range of h, k, l	$-8 < h < 8$; $-8 < k < 8$; $-23 < l < 23$	$-8 < h < 8$; $-8 < k < 8$; $-23 < l < 23$	$-7 < h < 7$; $-8 < k < 8$; $-23 < l < 23$	$-7 < h < 7$; $-8 < k < 8$; $-23 < l < 23$	$-7 < h < 7$; $-8 < k < 8$; $-23 < l < 23$
Reflections collected	7278	7093	6881	6645	6531
Independent reflections	1702	1716	1673	1632	1618
Observed reflections ($I \geq 2\sigma$)	1702	1716	1673	1632	1618
Absorption correction	Multi-scan	Multi-scan	Multi-scan	Multi-scan	Multi-scan
T_{min}, T_{max}	0.361, 0.450	0.364, 0.450	0.364, 0.450	-	-
R_{int}	0.0875	0.073	0.074	0.074	0.074
Spherical refinement					
R (F)	0.1346	0.1425	0.1396	0.1297	0.1242
R_w (F^2)	0.0644	0.0740	0.0705	0.0695	0.0731
Goodness of fit	0.778	1.888	1.368	1.492	1.703
H atom treatment	Restrained isotropic	Restrained isotropic	Restrained isotropic	Restrained isotropic	Restrained isotropic
No. of parameters	229	229	229	229	229
$\Delta\rho_{max}$, $\Delta\rho_{min}$, $\Delta\rho_{rms}$, (eÅ^{-3})	0.757; -0.673; 0.139	0.709; -0.816; 0.144	0.773; -0.839; 0.139	0.920; -0.740; 0.144	0.703; -0.823; 0.147
Multipolar refinement					
R (F)	0.1295	0.1280	0.1233	0.1120	0.1167
R_w (F^2)	0.0576	0.0680	0.0633	0.0612	0.0683
Goodness of fit	0.733	1.917	1.230	1.328	1.565
H atom treatment	Restrained isotropic	Restrained isotropic	Restrained isotropic	Restrained isotropic	Restrained isotropic
No. of parameters	238	238	238	238	238
$\Delta\rho_{max}$, $\Delta\rho_{min}$, $\Delta\rho_{rms}$, (eÅ^{-3})	0.668; -0.673; 0.133	0.693; -0.730; 0.141	0.733; -0.769; 0.137	0.737; -0.652; 0.136	0.712; -0.839; 0.142

Table S2 Cont'd.

Compound	**3.09 GPa**	**3.95 GPa**
Empirical formula	$C_{15}H_7Cl_2N_4O_2$	$C_{15}H_7Cl_2N_4O_2$
Formula wt. / g mol^{-1}	346.15	346.15
Colour	black	black
Crystal dimensions / mm	0.25 x 0.10 x 0.05	0.25 x 0.10 x 0.05
Space group	$P\,2_1/n$	$P\,2_1/n$

a / Å	6.2590(2)	6.2058(3)
b / Å	9.8519(12)	9.8299(15)
c / Å	19.8571(11)	19.7739(13)
α / °	90	90
β / °	95.624(4)	95.781(5)
γ / °	90	90
Z	4	4
V / Å^3	1218.56(17)	1200.1(2)
D_{calc} / g cm^{-3}	1.887	1.916
λ / Å	0.71073 (Mo$K\alpha$)	0.71073 (Mo$K\alpha$)
μ / mm^{-1}	0.550	0.559
Θ range / °	2.061 – 27.2	2.316 – 27.3
T / K	293(2)	293(2)
Diffractometer type	Gemini Ultra	Gemini Ultra
Range of h, k, l	$-7 < h < 7$; $-8 < k < 8$; $-23 < l < 22$	$-7 < h < 7$; $-8 < k < 8$; $-22 < l < 22$
Reflections collected	6137	6073
Independent reflections	1528	1502
Observed reflections ($I \geq 2\sigma$)	1528	1502
Absorption correction	Multi-scan	Multi-scan
T_{min}, T_{max}	0.368, 0.449	0.366, 0.449
R_{int}	0.076	0.070
Spherical refinement		
R (F)	0.1157	0.1113
R_w (F^2)	0.0831	0.0737
Goodness of fit	1.682	1.741
H atom treatment	Restrained isotropic	Restrained isotropic
No. of parameters	229	229
$\Delta\rho_{max}$, $\Delta\rho_{min}$, $\Delta\rho_{rms}$, (eÅ^{-3})	0.860; -0.784; 0.151	0.893; -0.780; 0.144
Multipolar refinement		
R (F)	0.1108	0.1030
R_w (F^2)	0.0766	0.0663
Goodness of fit	1.588	1.623
H atom treatment	Restrained isotropic	Restrained isotropic
No. of parameters	238	238
$\Delta\rho_{max}$, $\Delta\rho_{min}$, $\Delta\rho_{rms}$, (eÅ^{-3})	0.910; -0.827; 0.148	0.785; -0.800; 0.141

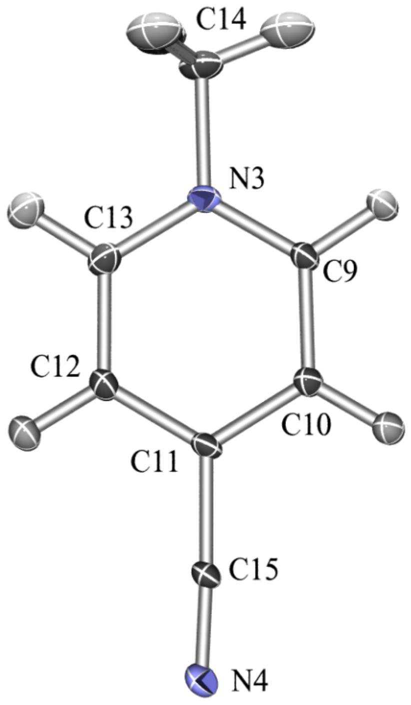

90 K

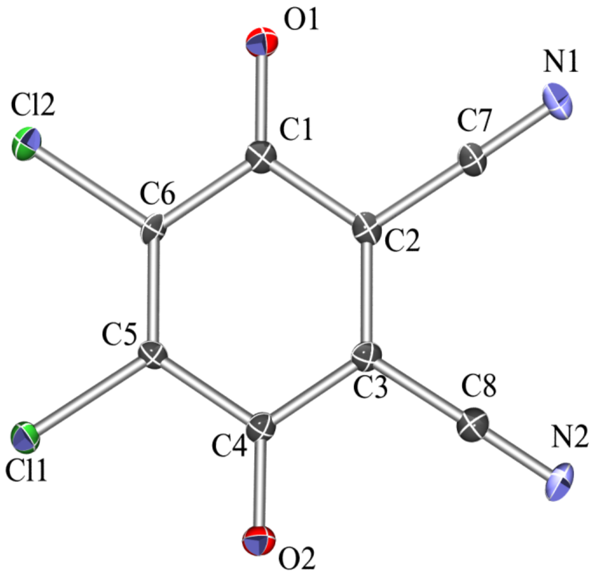

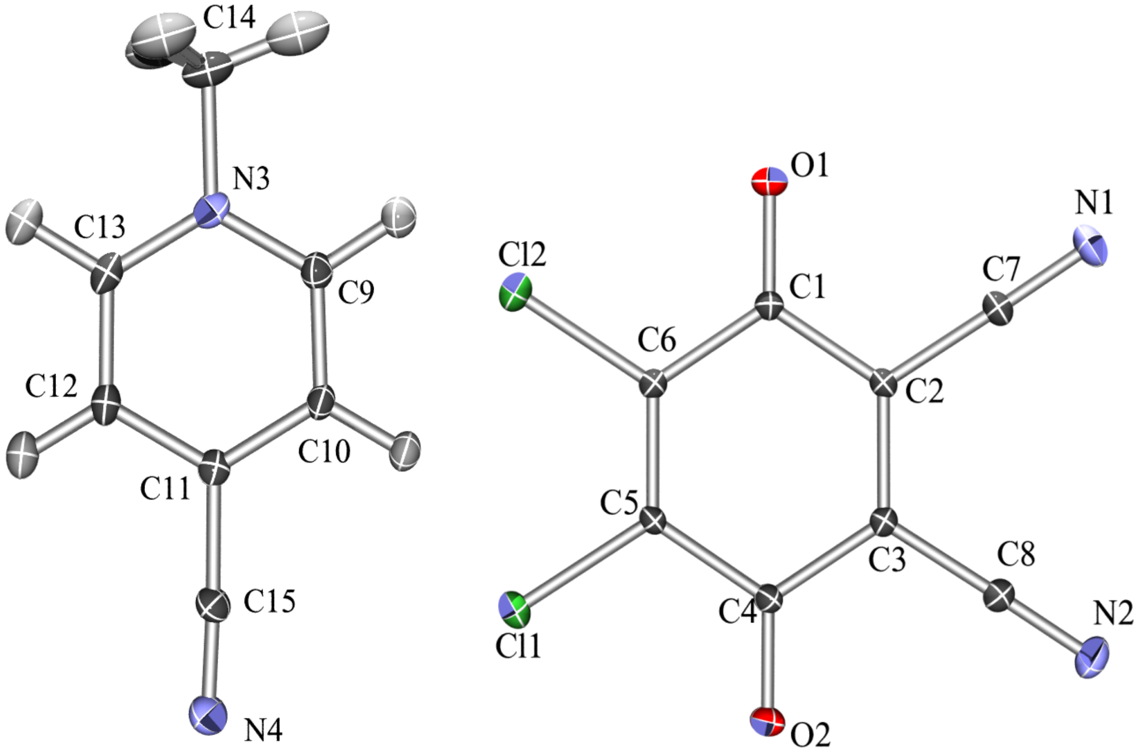
C14
N3
C13
C9
C12
C10
C11
C15
N4
O1
N1
Cl2
C7
C1
C6
C2
C5
C3
C8
C4
N2
Cl1
O2

120 K

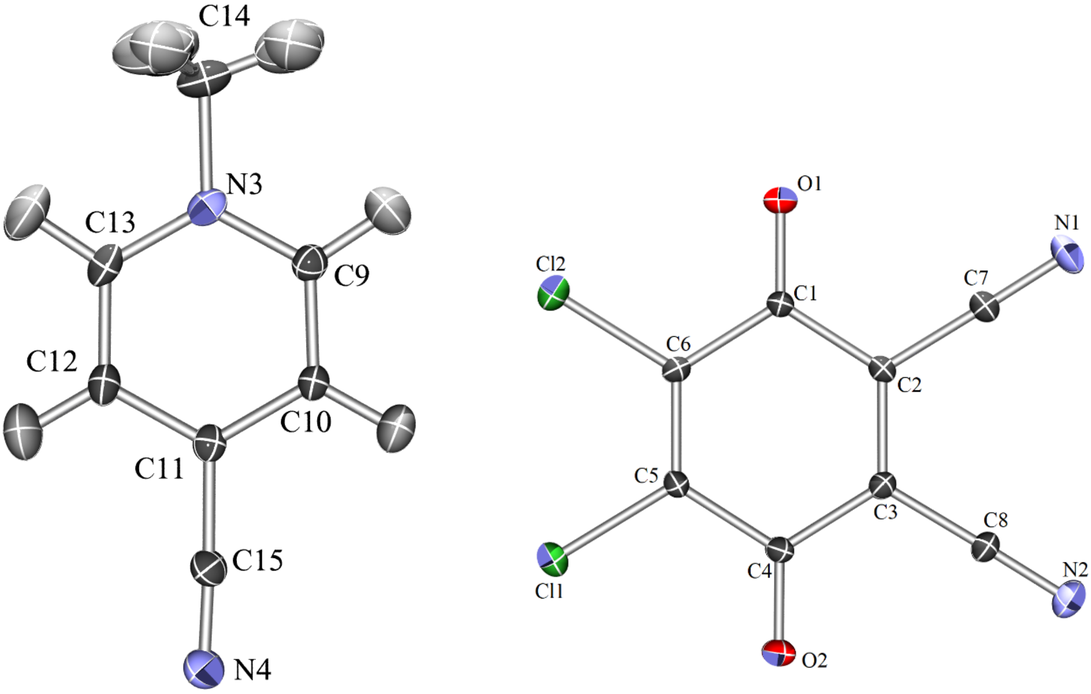
C14
N3
C13
C9
C12
C10
C11
C15
N4
O1
N1
Cl2
C7
C1
C6
C2
C5
C3
C8
C4
N2
Cl1
O2

150 K

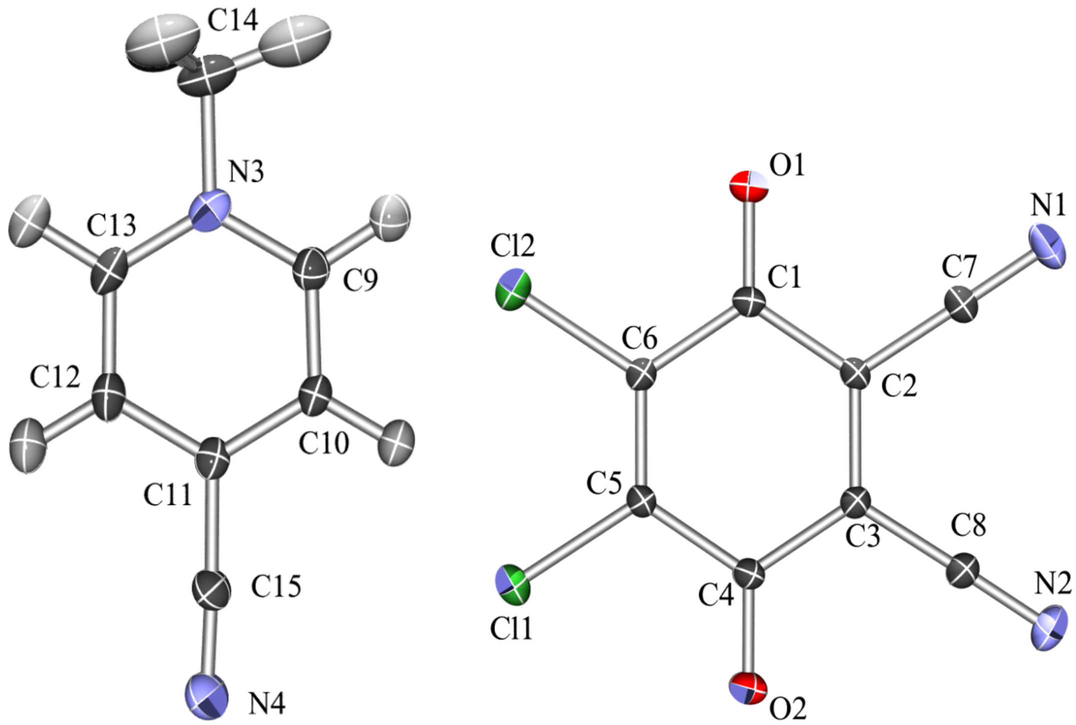
C14
N3
C13
C9
C12
C10
C11
C15
N4
O1
N1
Cl2
C1
C7
C6
C2
C5
C3
C8
C4
N2
Cl1
O2

180 K

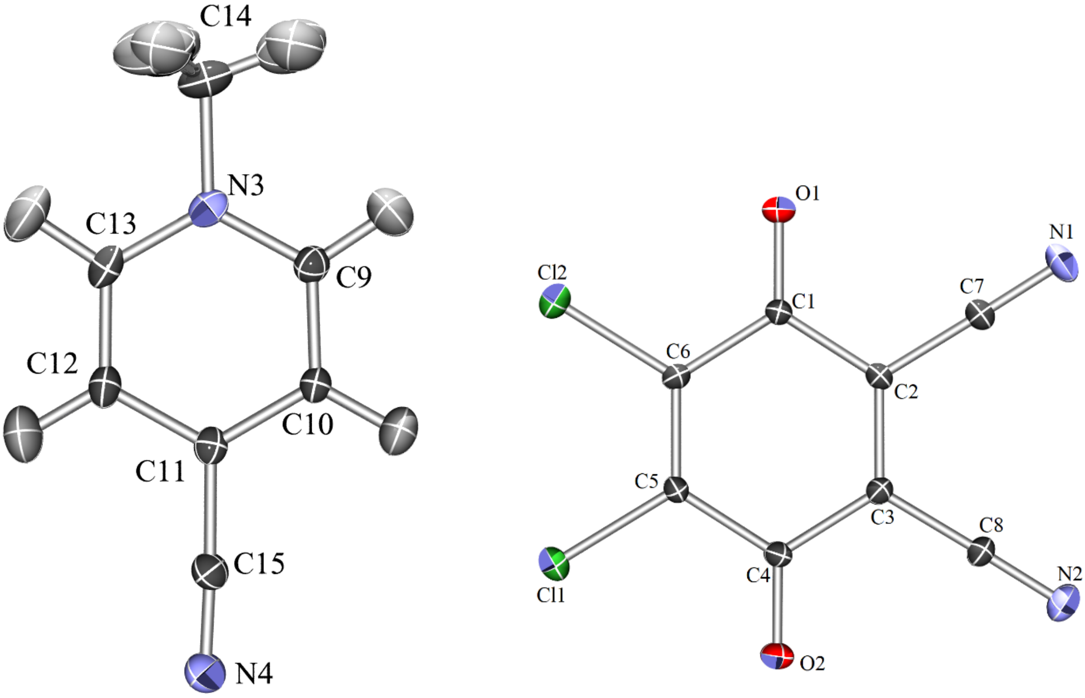
C14
N3
C13
C9
C12
C10
C11
C15
N4
O1
N1
Cl2
C7
C1
C6
C2
C5
C3
C8
C4
N2
Cl1
O2

210 K

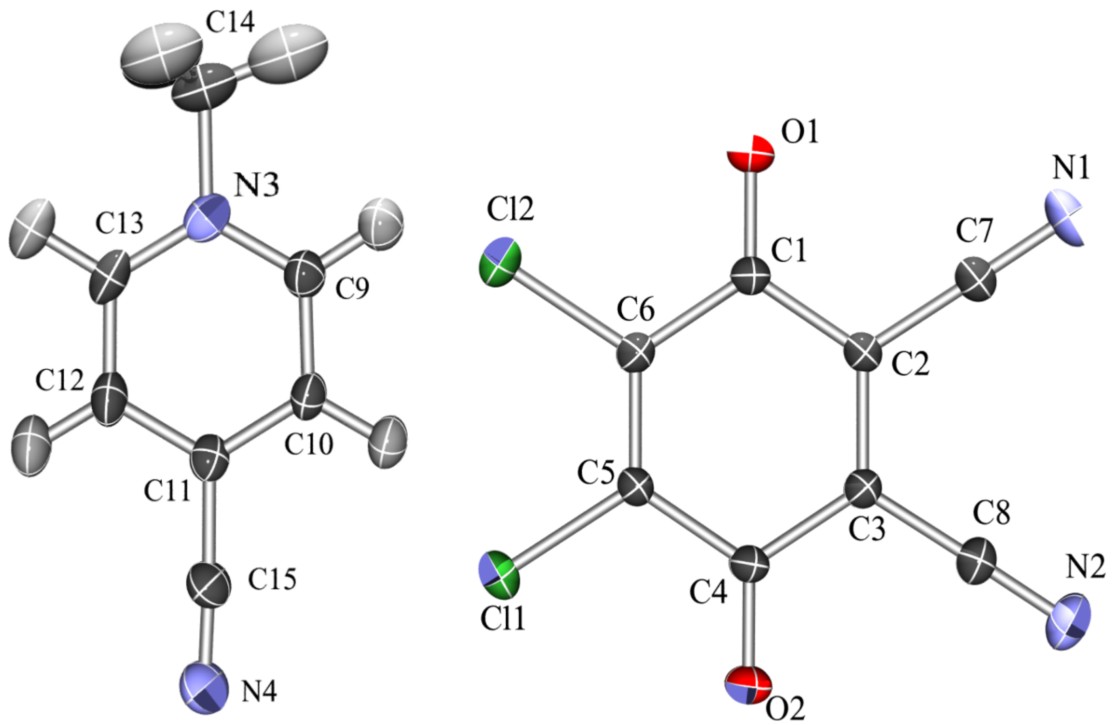
C14
N3
C13
C9
C12
C10
C11
C15
N4
O1
N1
Cl2
C7
C1
C6
C2
C5
C3
C8
Cl1
C4
N2
O2

240 K

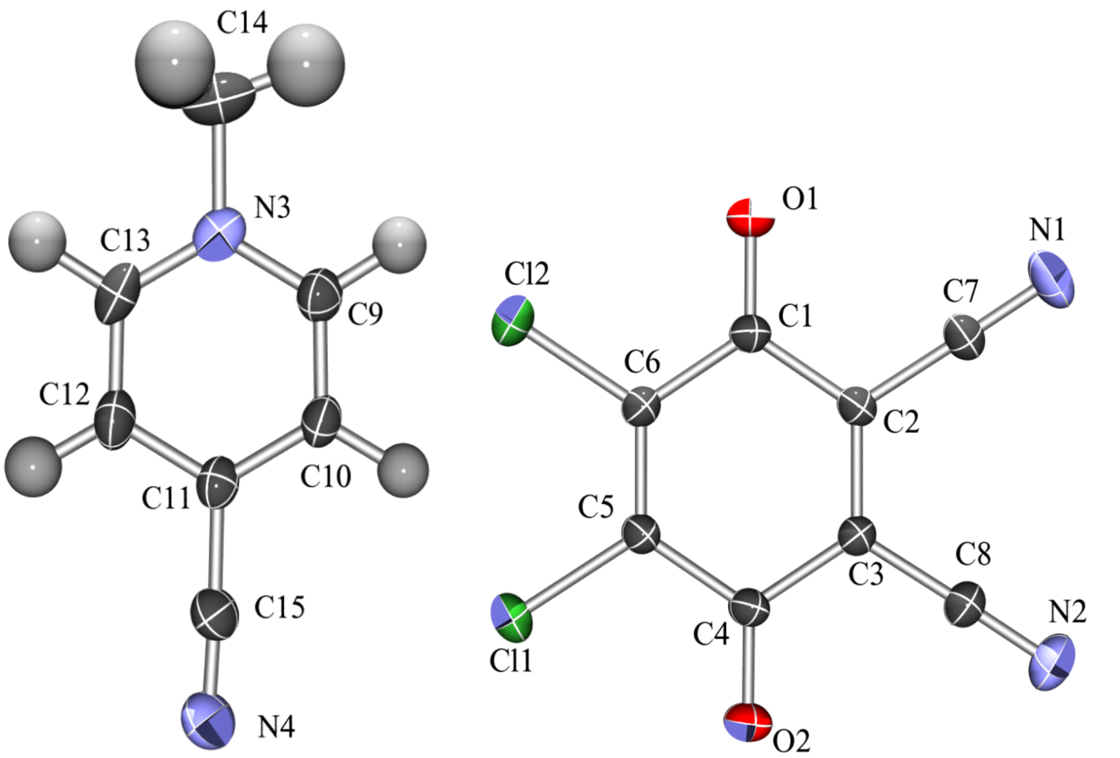
C14
N3
C13
C9
C12
C10
C11
C15
N4
O1
N1
Cl2
C7
C1
C6
C2
C5
C3
C8
C4
Cl1
N2
O2

270K

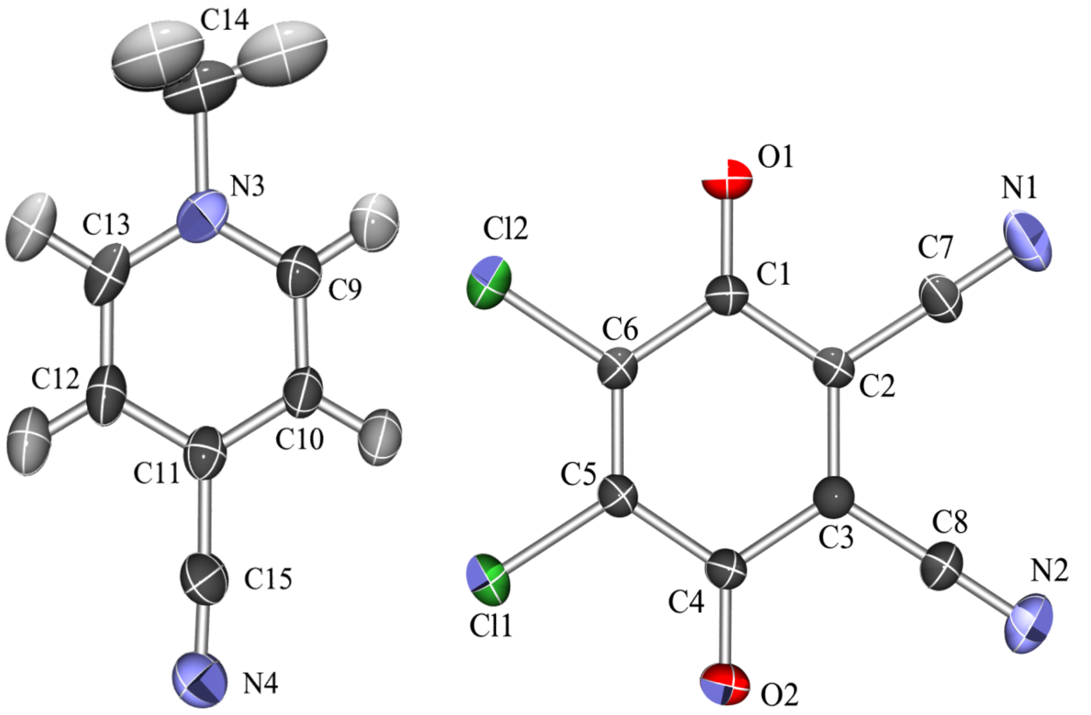
C14
N3
C13
C9
C12
C10
C11
C15
N4
O1
Cl2
N1
C7
C1
C6
C2
C5
C8
C3
N2
C4
Cl1
O2

293 K

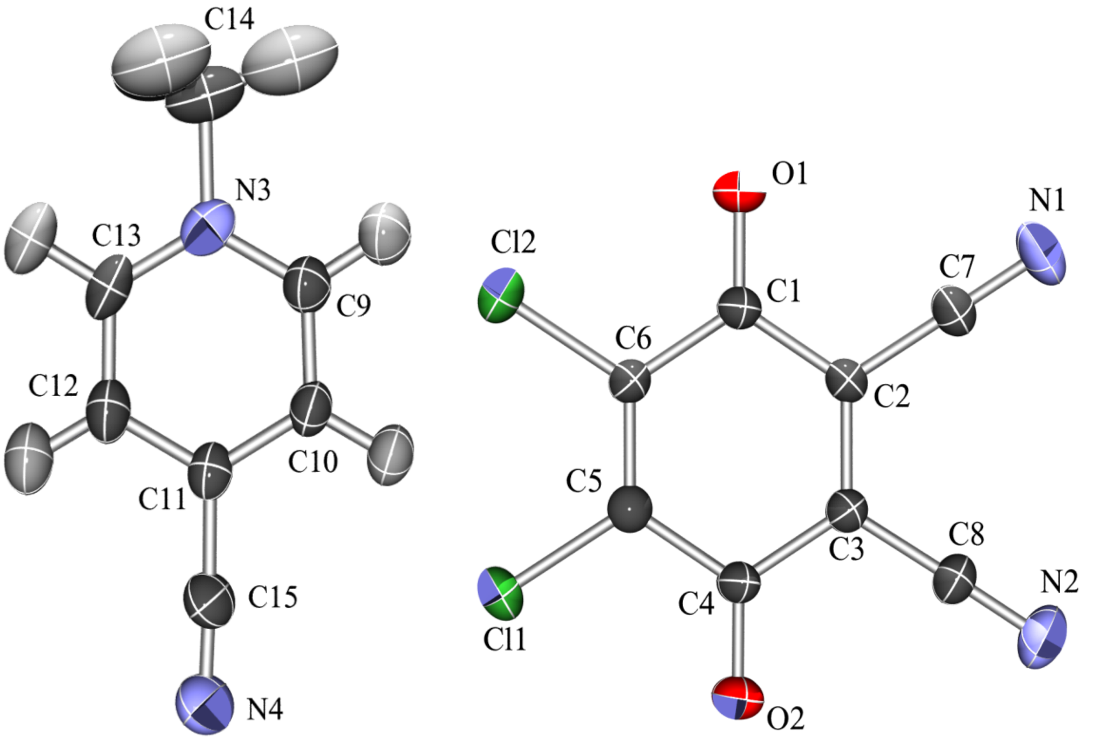
C14
N3
C13
C9
C12
C10
C11
C15
N4
O1
Cl2
N1
C7
C1
C6
C2
C5
C8
C3
N2
C4
Cl1
O2

310 K

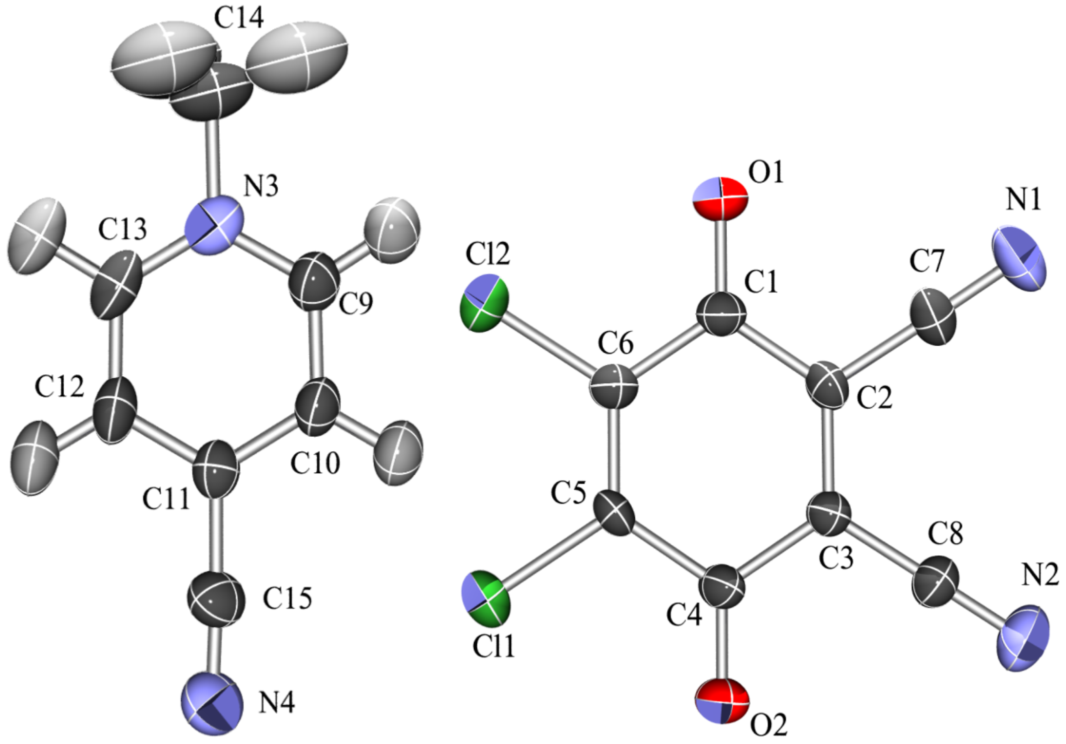

340 K

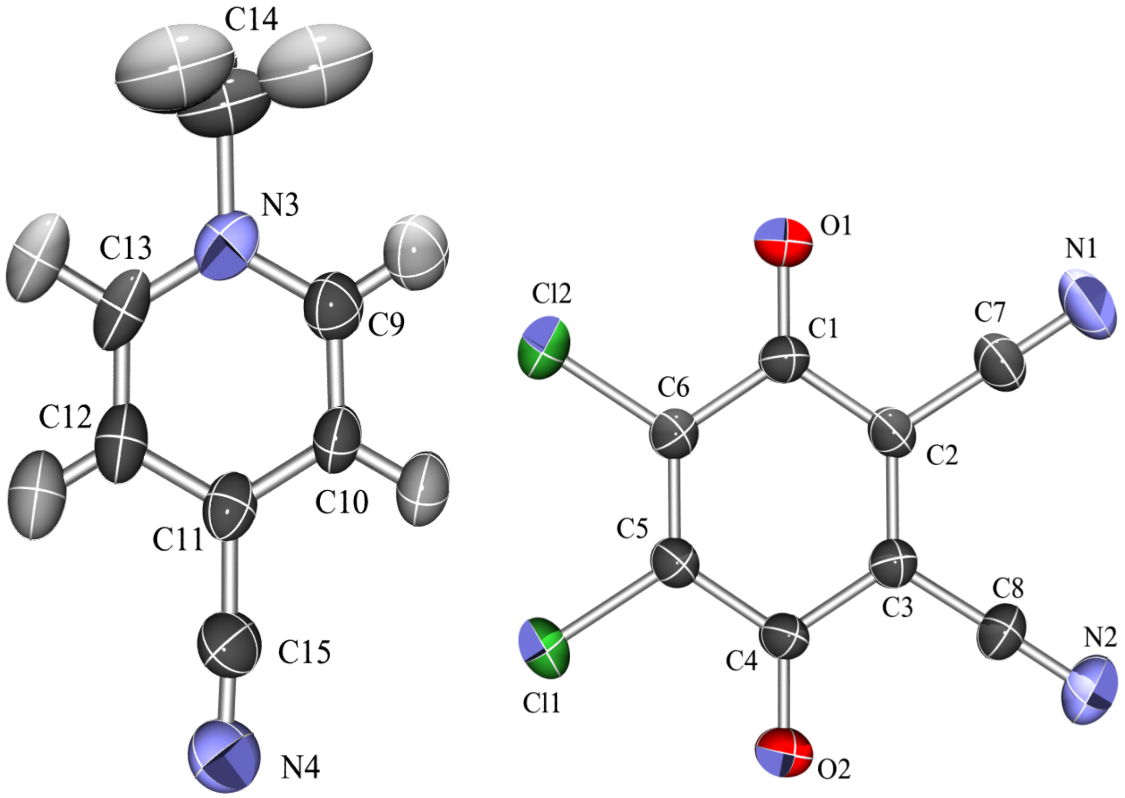

370 K

Figure S1 ORTEP-3 [1] diagram of 4-cyano-*N*-methylpyridinium cation (left) and DDQ radical anion (right) after transferred-multipolar refinement at various temperatures. Displacement ellipsoids are shown for the probability of 50 %.

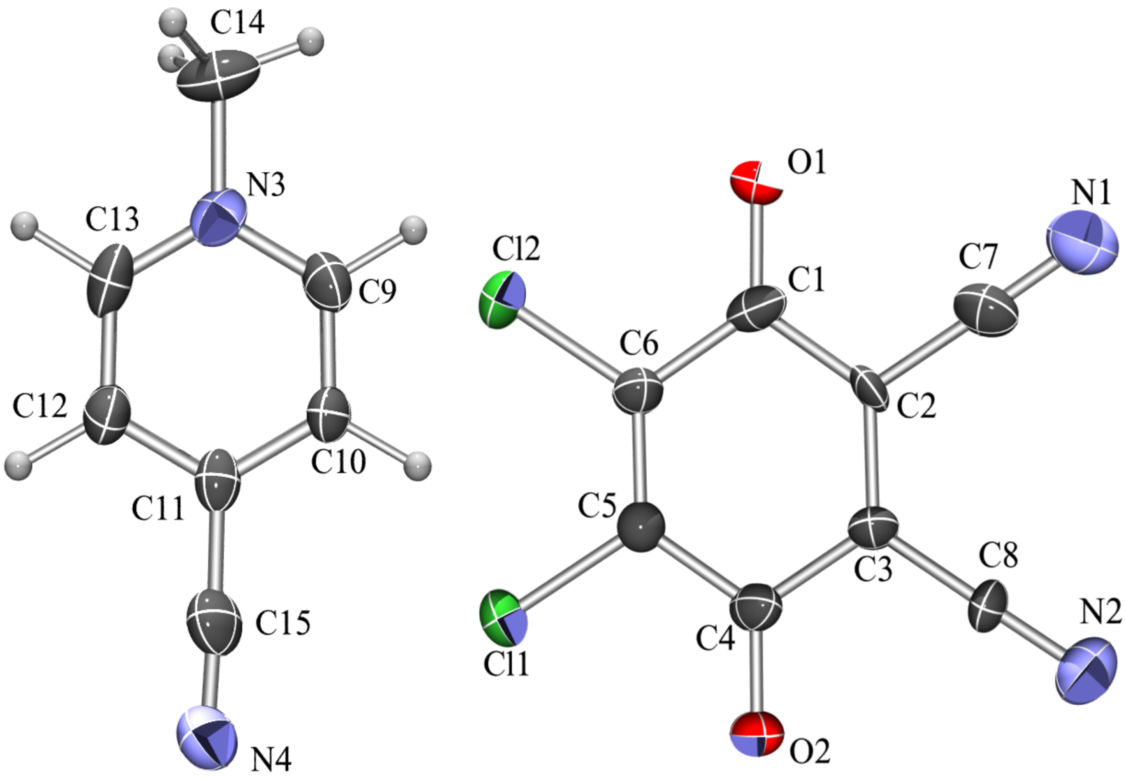

0.25 GPa

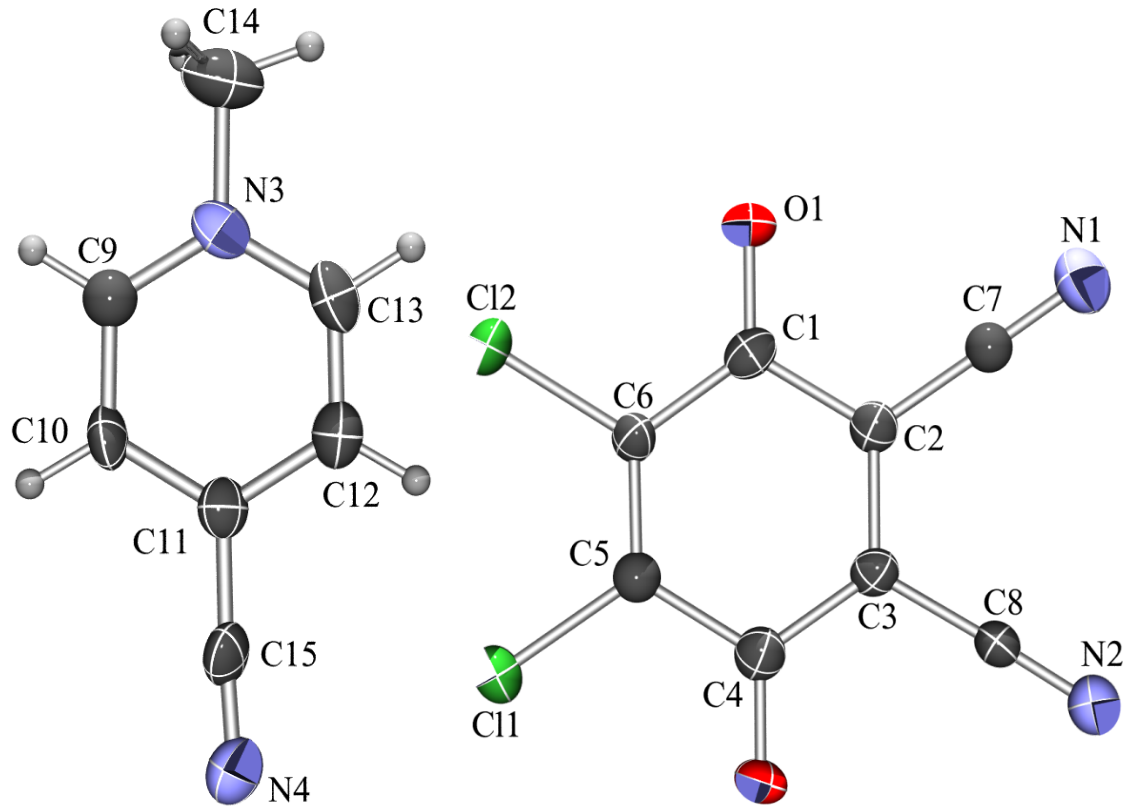

0.49 GPa

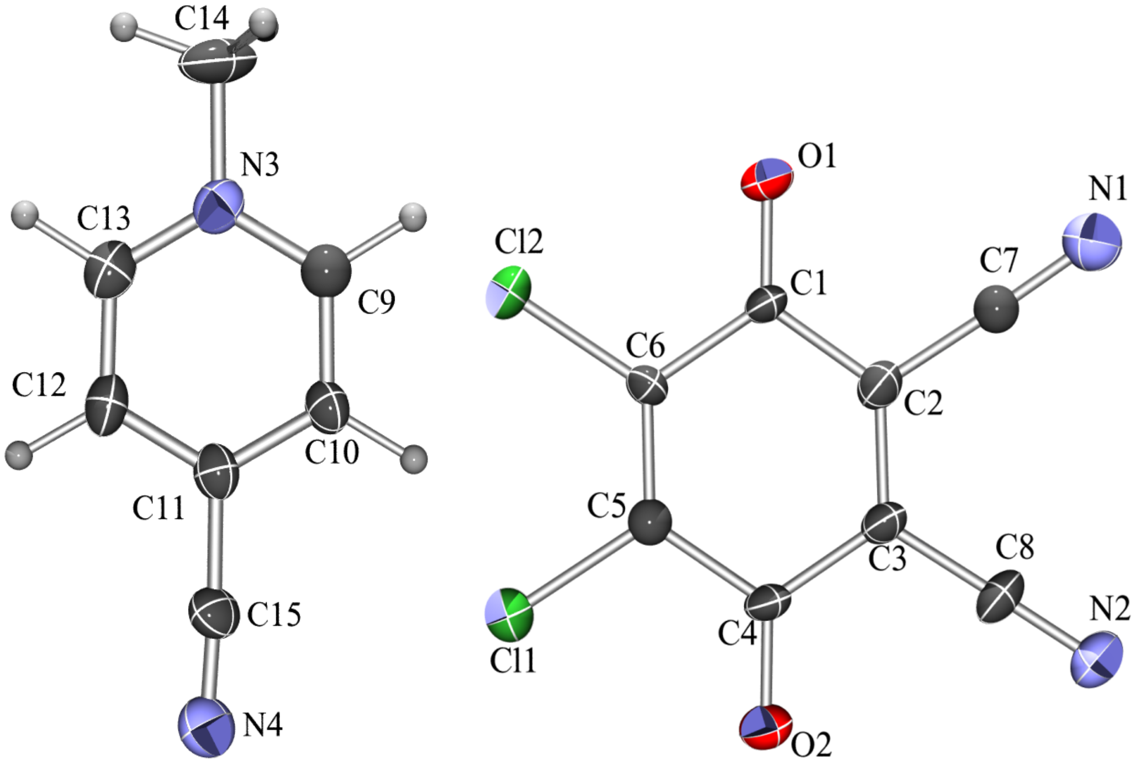

0.86 GPa

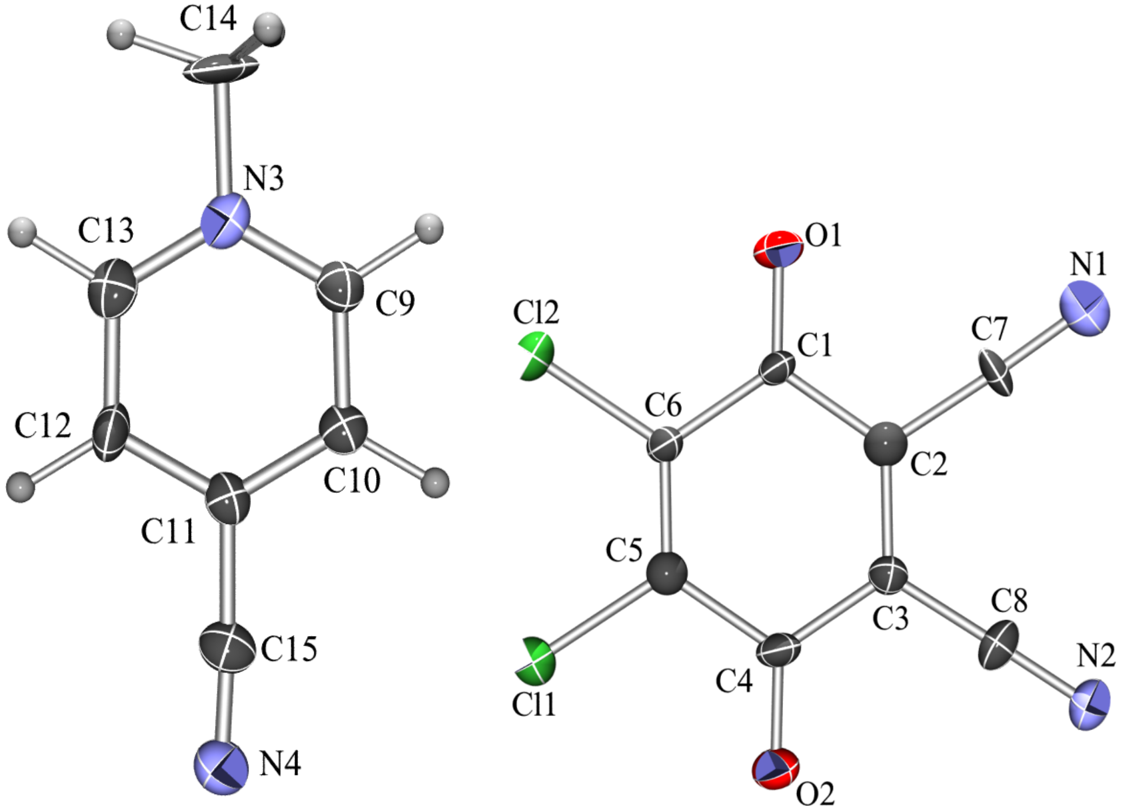

1.42 GPa

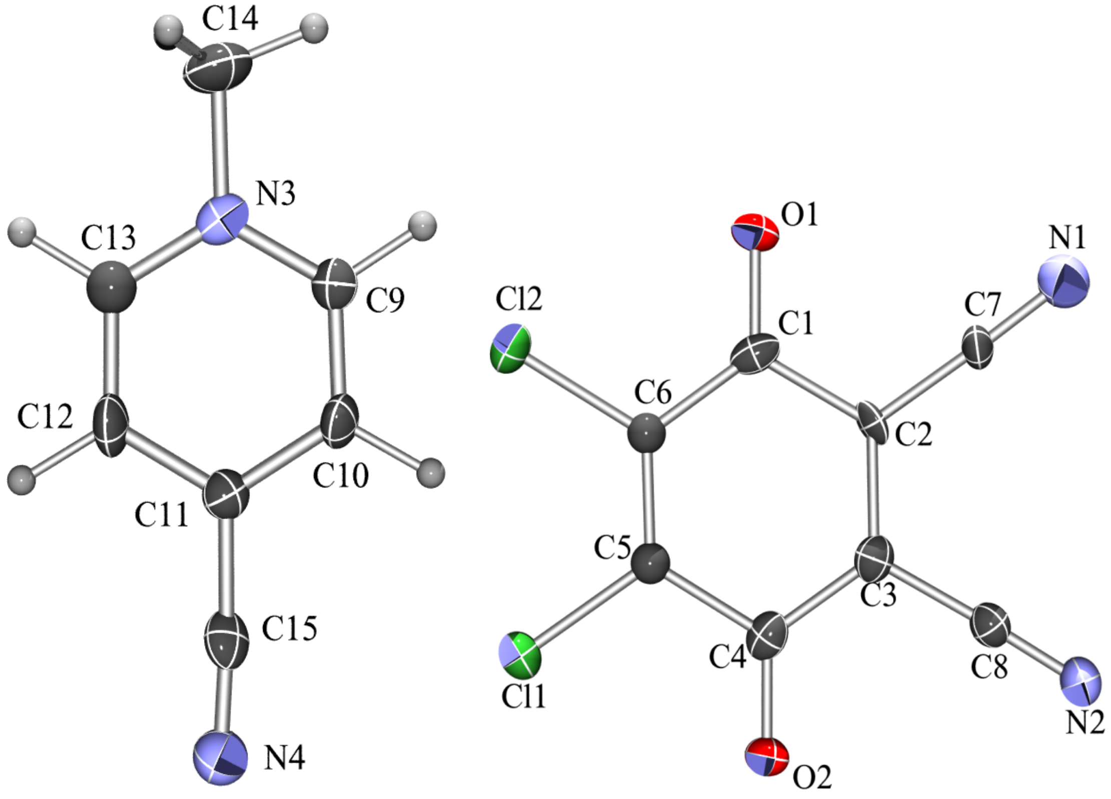

1.85 GPa

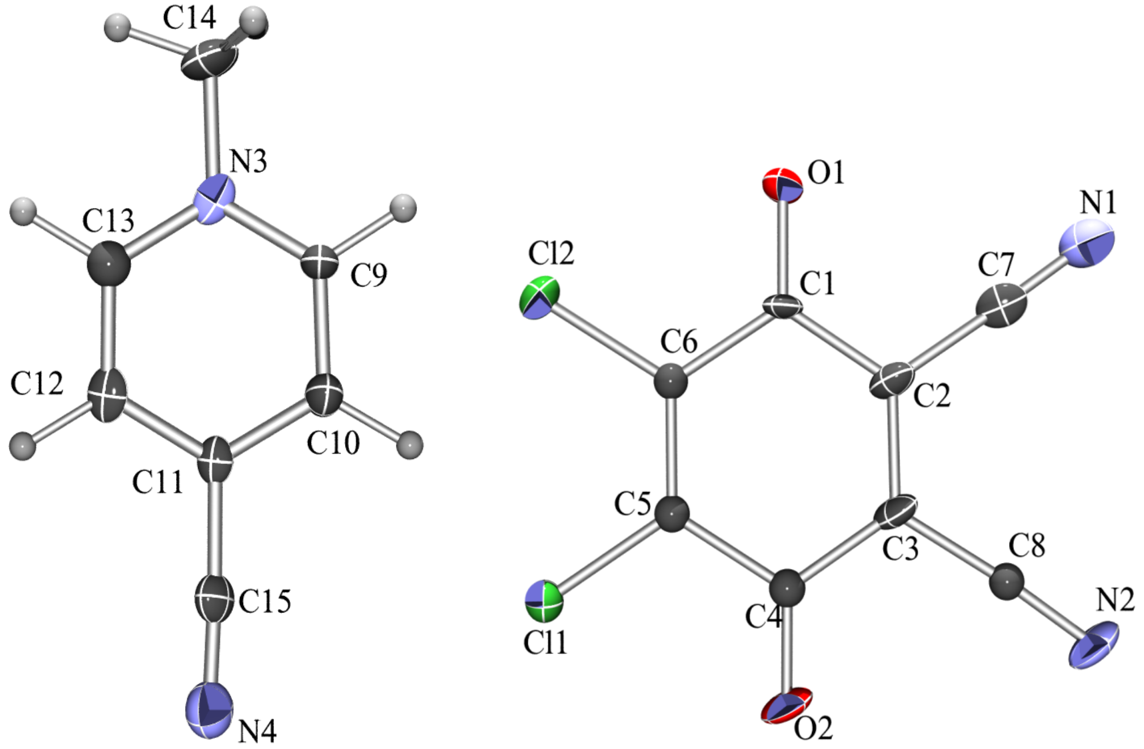

3.09 GPa

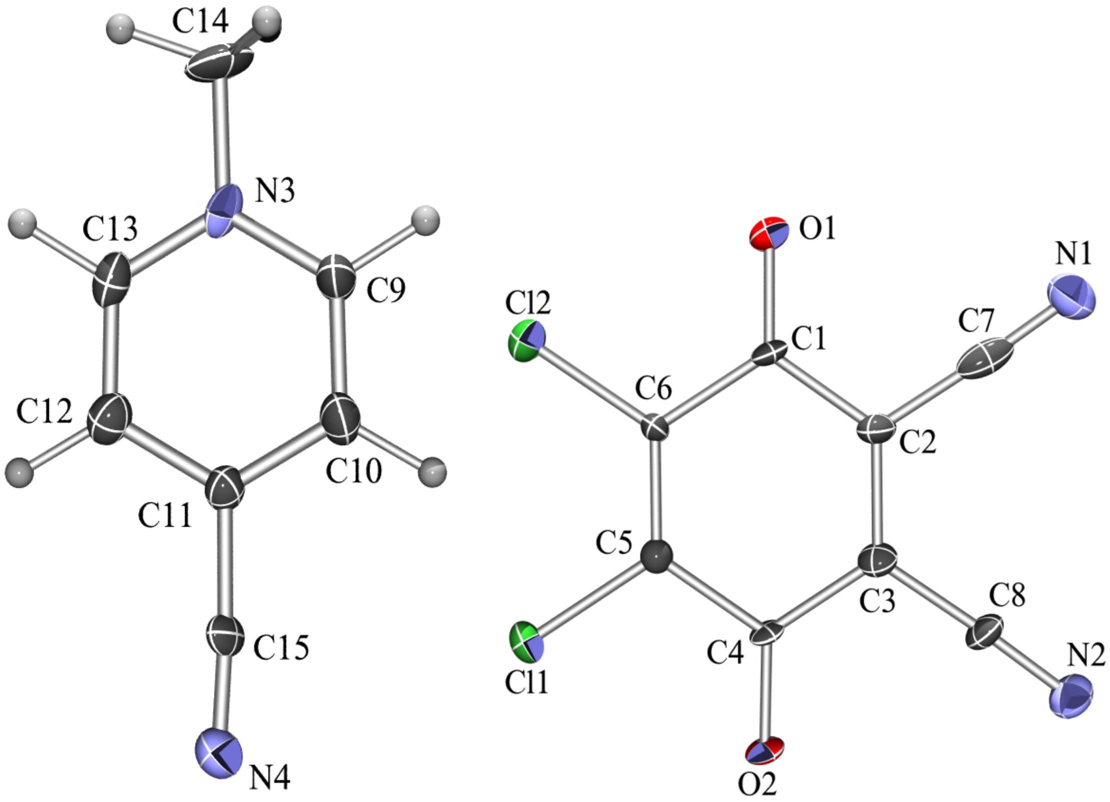

3.95 GPa

Figure S2 ORTEP-3 [1] diagram of 4-cyano-*N*-methylpyridinium cation (left) and DDQ radical anion (right) after transferred-multipolar refinement at various pressures. Displacement ellipsoids are shown for the probability of 50 % and hydrogen atoms are shown as spheres of arbitrary radii.

S2 Generation of a set of transferrable multipoles: data on refinement (including a list of constraints and restraints used) and residual densities

Table S3 Details on refinement of charge density for generation of transferrable multipoles. Original multipolar refinement with minimal restraints taken from ref. [2] and a simplified model with constraints, to obtain a set of transferrable multipoles.

Unrestrained multipolar refinement [2]	
Weighting scheme	$w = 1/[6\sigma^2(F_o^2)]$
R (F)	0.0298
R_w (F^2)	0.0285
Goodness of fit	1.518
H atom treatment	Restrained anisotropic
No. of parameters	709
No. of restraints	477
$\Delta\rho_{max}$, $\Delta\rho_{min}$, $\Delta\rho_{rms}$ (eÅ^{-3})	0.366; -0.369; 0.064
Constrained multipolar refinement for obtaining transferrable multipoles	
Weighting scheme	$w = 1/[6\sigma^2(F_o^2)]$
R (F)	0.0309
R_w (F^2)	0.0312
Goodness of fit	1.461
H atom treatment	Restrained anisotropic
No. of parameters	709
No. of restraints	477
$\Delta\rho_{max}$, $\Delta\rho_{min}$, $\Delta\rho_{rms}$ (eÅ^{-3})	0.400; -0.364; 0.065

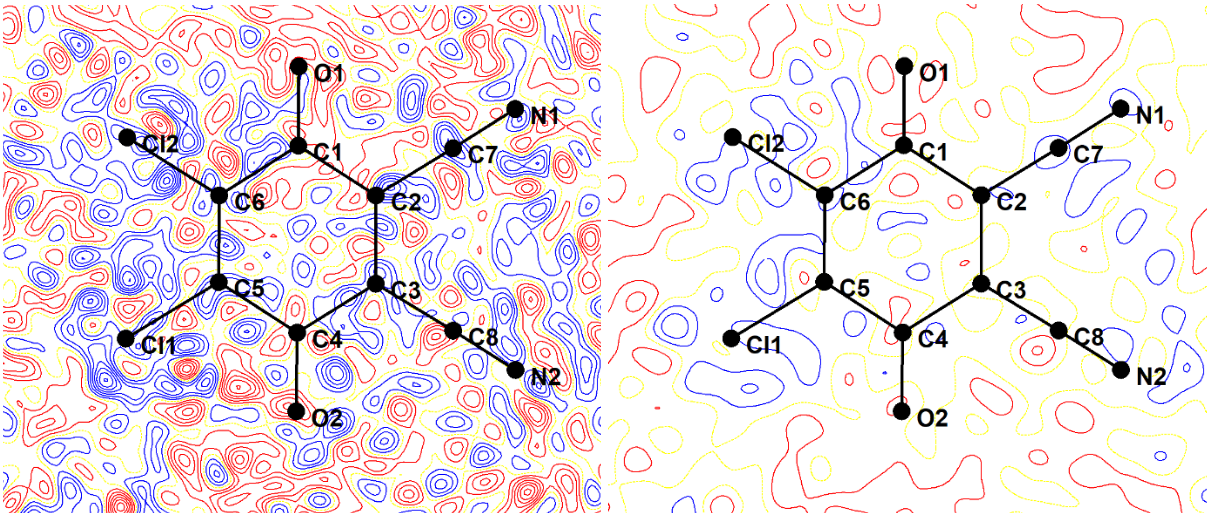

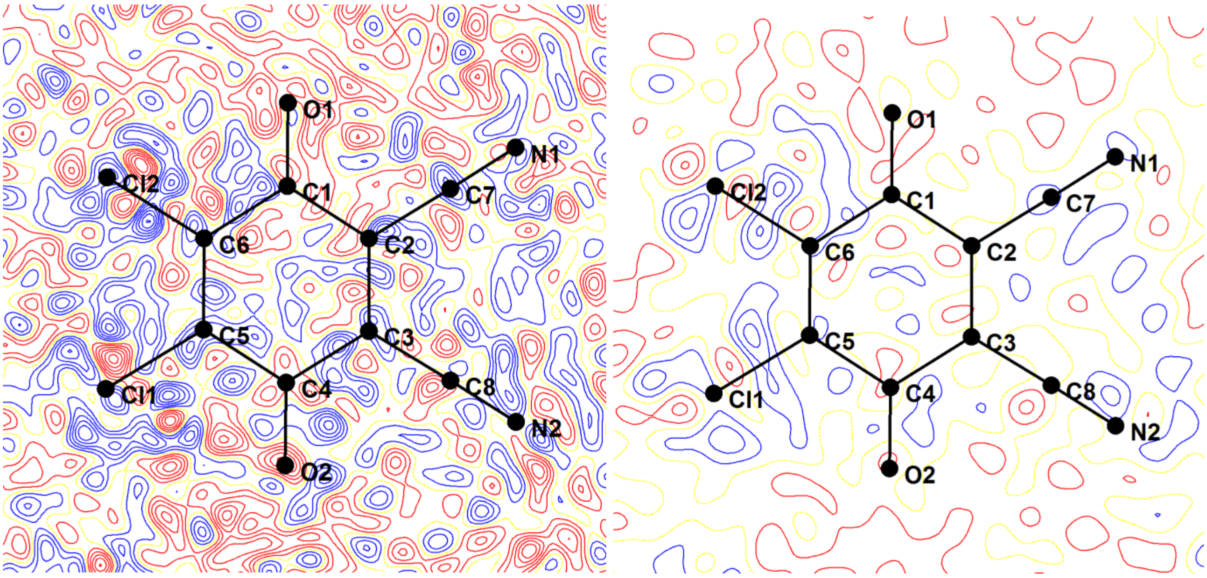

Figure S3 Residual density in the plane of the DDQ radical anion with all reflections used (left) and only low-angle reflections (rignt; $s < 0.7$ Å^{-1}) used. Data from the original minimal-restraint multipolar refinement [2] is shown in the top row and data from constrained multipolar refinement for generation of transferrable multipoles is shown in the bottom row. Positive density is shown in blue and negative in red; yellow dotted lines represent zero density. Contours are drawn for 0.05 eÅ^{-1}.

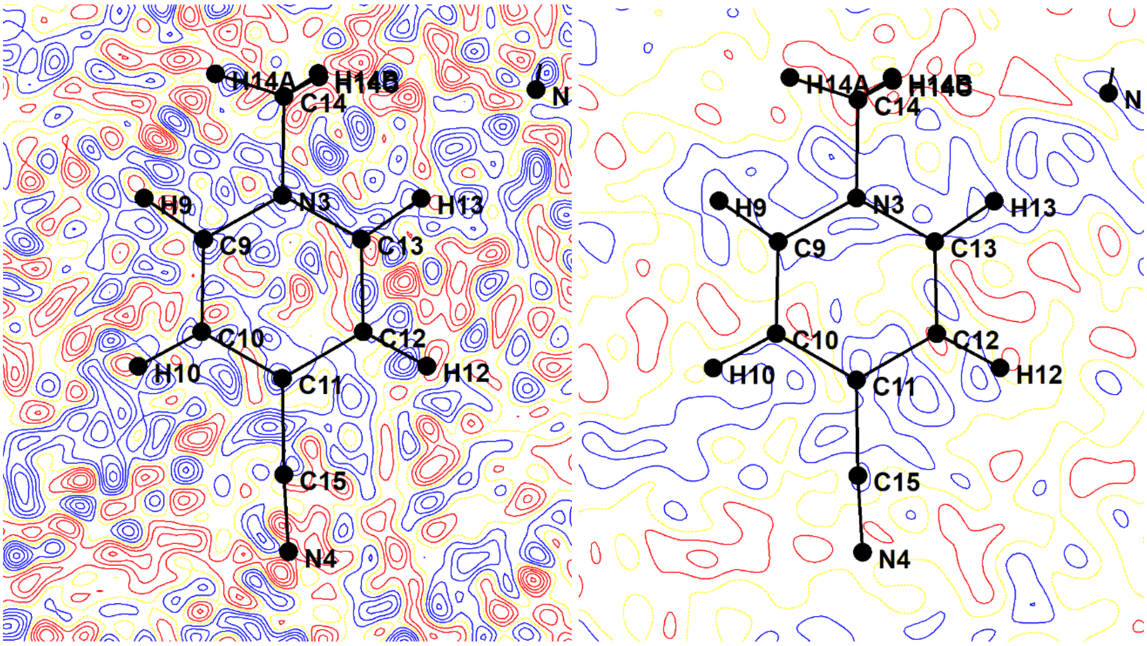

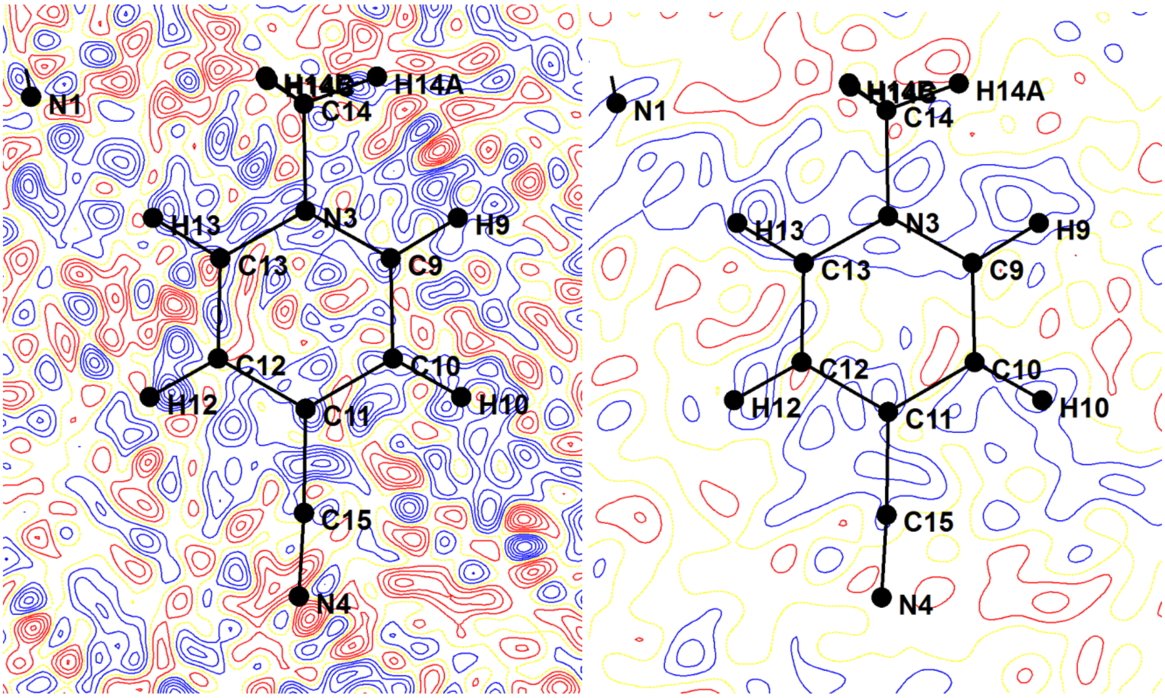

Figure S4 Residual density in the plane of the 4-CN-*N*-MePy cation with all reflections used (left) and only low-angle reflections (right; $s < 0.7$ Å^{-1}) used. Data from the original minimal-restraint multipolar refinement [2] is shown in the top row and data from constrained multipolar refinement for generation of transferrable multipoles is shown in the bottom row. Positive density is shown in blue and negative in red; yellow dotted lines represent zero density. Contours are drawn for 0.05 eÅ^{-1}.

Table S4 List of constraints and restraints used to generate a set of transferrable multipoles.

CONSTRAINTS

```
FIXUIJ    H9  1  0.023881   0.038016   0.025286  -0.005153   0.007173  -0.007213
FIXUIJ   H10  1  0.029901   0.036509   0.019252  -0.005644   0.001479  -0.009212
FIXUIJ   H12  1  0.032035   0.033231   0.021116  -0.008370   0.006930  -0.000709
FIXUIJ   H13  1  0.032587   0.029130   0.018226  -0.004551  -0.000214  -0.006100
FIXUIJ  H14A  1  0.031365   0.047547   0.025164  -0.007117   0.009576  -0.010196
FIXUIJ  H14B  1  0.034845   0.032385   0.033437   0.000906  -0.011153   0.005875
FIXUIJ  H14C  1  0.029463   0.021814   0.044473   0.004242   0.002950  -0.011162

CONKAP Cl1    1  Cl2    1
CONKAP O1     1  O2     1
CONKAP N1     1  N2     1
CONKAP C1     1  C4     1
CONKAP C2     1  C3     1
CONKAP C5     1  C6     1
CONKAP C7     1  C8     1
CONKAP C9     1  C13    1
CONKAP C10    1  C12    1
CONKAP H9     1  H13    1
CONKAP H10    1  H12    1
CONKAP H14A   1  H14B   1  H14C   1

AVEPVM Cl1    1  Cl2    1
AVEPVM O1     1  O2     1
```

```
AVEPVM N1    1  N2    1
AVEPVM C1    1  C4    1
AVEPVM C2    1  C3    1
AVEPVM C5    1  C6    1
AVEPVM C7    1  C8    1
AVEPVM C9    1  C13   1
AVEPVM C10   1  C12   1

AVEPLM C7    1  C15   1
AVEPVM H9    1  H13   1
AVEPVM H10   1  H12   1
AVEPVM H14A  1  H14B  1  H14C  1
AVEPLM H9    1  H14A  1

SYMPLM mxmy   Cl1   1
SYMPLM mxmy   Cl2   1
SYMPLM mymz   O1    1
SYMPLM mymz   O2    1
SYMPLM cy     N1    1
SYMPLM cy     N2    1
SYMPLM mymz   C1    1
SYMPLM mymz   C2    1
SYMPLM mymz   C3    1
SYMPLM mymz   C4    1
SYMPLM mz     C5    1
SYMPLM mz     C6    1
SYMPLM cy     C7    1
SYMPLM cy     C8    1
SYMPLM mymz   N3    1
SYMPLM cy     N4    1
SYMPLM mz     C9    1
SYMPLM mymz   C10   1
SYMPLM mymz   C11   1
SYMPLM mymz   C12   1
SYMPLM mz     C13   1
SYMPLM 3m     C14   1
SYMPLM cy     C15   1

CONURA  C9    1   H9     1   1.2
CONURA  C10   1   H10    1   1.2
CONURA  C12   1   H12    1   1.2
CONURA  C13   1   H13    1   1.2
CONURA  C14   1   H14C   1   1.5
CONURA  C14   1   H14B   1   1.5
CONURA  C14   1   H14A   1   1.5

CONDIS  C9    1   H9     1   1.083
CONDIS  C10   1   H10    1   1.083
CONDIS  C12   1   H12    1   1.083
CONDIS  C13   1   H13    1   1.083
CONDIS  C14   1   H14C   1   1.077
CONDIS  C14   1   H14B   1   1.077
CONDIS  C14   1   H14A   1   1.077

RESTRAINTS
RESVAL Cl1 1 7.15 0.01
```

RESVAL Cl2 1 7.15 0.01

RESVAL O1 1 6.20 0.01

RESVAL O2 1 6.20 0.01

RESVAL N1 1 5.00 0.01

RESVAL N2 1 5.00 0.01

RESVAL N3 1 4.95 0.01

RESVAL N4 1 5.00 0.01

RESVAL C1 1 3.9 0.01

RESVAL C2 1 4.0 0.01

RESVAL C3 1 4.0 0.01

RESVAL C4 1 3.9 0.01

RESVAL C5 1 4.0 0.01

RESVAL C5 1 4.0 0.01

RESVAL C7 1 4.15 0.01

RESVAL C8 1 4.15 0.01

RESVAL C9 1 4.10 0.01

RESVAL C10 1 3.90 0.01

RESVAL C12 1 3.90 0.01

RESVAL C11 1 4.00 0.01

RESVAL C13 1 4.10 0.01

RESVAL C14 1 4.00 0.01

RESVAL C15 1 4.10 0.01

KAPPA2 CHEM H 1.15 0.04

KAPPA1 CHEM H 1.15 0.04

S3 List of transferrable multipoles

Table S5 List of transferrable multipoles (ELMAM2 [3] output).

```
  CHEMAT   AXES   AX   AY   AZ   NEIGH   COMPOUND

==========================================================================
ATOM    Cl1_0    Cl     ZX    C    C    -    C        Clc
NBOND   1  CYCL  0       CHIV  -       CHIR  0
SYMPLM  mxmy     CONVAL  -  CONPLM  -  CONKAP  -
DIST    Cl-C  1.710
ANGL
KMD      1.00082 0.71662  7.194  0.    0.    0.   -0.043
KMD_ESD  0.00165 0.02314  0.016  0.    0.    0.    0.008
QUA                  -0.167  0.    0.   -0.010  0.
QUA_ESD                0.009  0.    0.    0.007  0.
OCT        0.133  0.    0.   -0.008  0.    0.    0.
OCT_ESD    0.009  0.    0.    0.007  0.    0.    0.
HEX        0.028  0.    0.   -0.011  0.    0.    0.   -0.020  0.
HEX_ESD    0.010  0.    0.    0.009  0.    0.    0.    0.009  0.
TEXT Cl_C_CC_ClO   Cl1c$[1.5c%(1cl1c)1c(1.5o1c)]
==========================================================================ATOM
O1_1      O      XY    C    C    -    C        Oc
NBOND   1  CYCL  0       CHIV  -       CHIR  0
SYMPLM  mymz     CONVAL  -  CONPLM  -  CONKAP  -
DIST    O-C  1.247
ANGL
KMD      0.98506 0.83185  6.192  0.   -0.051  0.    0.
KMD_ESD  0.00145 0.04217  0.015  0.    0.009  0.    0.
QUA                  -0.086  0.    0.   -0.080  0.
QUA_ESD                0.008  0.    0.    0.008  0.
OCT        0.   -0.029  0.    0.    0.    0.028  0.
OCT_ESD    0.    0.007  0.    0.    0.    0.006  0.
TEXT O_C_CC_Cl   O1.5c$[1c%(1cl1.5c)1c(1.5c1.5c)]
==========================================================================
ATOM    N1_1      N      ZX    C    C    -    C        Nc
NBOND   1  CYCL  0       CHIV  -       CHIR  0
SYMPLM  cy       CONVAL  -  CONPLM  -  CONKAP  -
DIST    N-C  1.158
ANGL
KMD      0.99260 0.98960  5.000  0.    0.    0.   -0.038
KMD_ESD  0.00187 0.03890  0.016  0.    0.    0.    0.009
QUA                   0.192  0.    0.    0.    0.
QUA_ESD                0.010  0.    0.    0.    0.
OCT        0.056  0.    0.    0.    0.    0.    0.
OCT_ESD    0.009  0.    0.    0.    0.    0.    0.
TEXT N_C_C   N2c$[1.5c(1c1.5c)]
==========================================================================
ATOM    C1_1      C     bXY    C    C    -    OCC      Cocc
NBOND   3  CYCL  0       CHIV  0.016    CHIR  0
SYMPLM  mymz     CONVAL  -  CONPLM  -  CONKAP  -
DIST    C-O  1.247   C-C  1.469   C-C  1.450
ANGL    O-C-C  122.7   O-C-C  122.4   C-C-C  114.9
KMD      1.00042 0.90646  3.895  0.   -0.092  0.    0.
```

```
KMD_ESD  0.00246 0.01722  0.017  0.     0.012  0.     0.
QUA                -0.252  0.     0.     0.081  0.
QUA_ESD                    0.009  0.     0.     0.009  0.
OCT        0.   -0.011  0.     0.     0.    -0.347  0.
OCT_ESD    0.    0.009  0.     0.     0.     0.014  0.
TEXT C_OCC_ClCCC_ClN   C1.5o1c$[1cl1.5c(1cl1c)]1c%[1.5c(2n)1.5c(1c1.5c)]
===========================================================================
ATOM    C2_1     C     bXY   C   C   -    CCC      Cccc
NBOND   3  CYCL  0        CHIV  0.030   CHIR  0
SYMPLM  mymz      CONVAL  -  CONPLM  -  CONKAP  -
DIST    C-C  1.450   C-C  1.425   C-C  1.389
ANGL    C-C-C  115.9   C-C-C  122.6   C-C-C  121.5
KMD      0.99561 0.88083  3.988  0.    -0.013  0.     0.
KMD_ESD  0.00254 0.01838  0.016  0.     0.007  0.     0.
QUA                -0.179  0.     0.     0.018  0.
QUA_ESD                    0.010  0.     0.     0.006  0.
OCT        0.   -0.015  0.     0.     0.    -0.336  0.
OCT_ESD    0.    0.007  0.     0.     0.     0.015  0.
TEXT C_CCC_ONCCC_ClON   C1c$[1.5o1c(1cl1.5c)]1.5c%[2n]1.5c[1c(1.5o1c)1.5c(2n)]
===========================================================================
ATOM    C5_1     C     bXY   C   C   -    ClCC     Cclcc
NBOND   3  CYCL  0        CHIV  0.002   CHIR  0
SYMPLM  mz        CONVAL  -  CONPLM  -  CONKAP  -
DIST    C-Cl  1.710   C-C  1.363   C-C  1.468
ANGL    Cl-C-C  121.6   Cl-C-C  115.8   C-C-C  122.6
KMD      0.99365 0.93029  4.007  0.     0.066 -0.006  0.
KMD_ESD  0.00252 0.02138  0.012  0.     0.008  0.007  0.
QUA                -0.164  0.     0.    -0.060 -0.037
QUA_ESD                    0.009  0.     0.     0.007  0.006
OCT        0.    0.037 -0.012  0.     0.    -0.280 -0.030
OCT_ESD    0.    0.007  0.007  0.     0.     0.014  0.007
TEXT C_ClCC_ClOCC_O   C1cl1.5c$[1cl1c(1.5o1c)]1c%[1.5o1c(1.5c1.5c)]
===========================================================================
ATOM    C7_1     C      ZX   N   C   -    NC       Cnc
NBOND   2  CYCL  0        CHIV  -       CHIR  0
SYMPLM  cy        CONVAL  -  CONPLM  -  CONKAP  -
DIST    C-N  1.158   C-C  1.425
ANGL    N-C-C  179.1
KMD      1.00625 0.86503  4.138  0.     0.     0.     0.258
KMD_ESD  0.00267 0.01916  0.016  0.     0.     0.     0.013
QUA                 0.409  0.     0.     0.     0.
QUA_ESD                    0.014  0.     0.     0.     0.
OCT       -0.012  0.     0.     0.     0.     0.     0.
OCT_ESD    0.010  0.     0.     0.     0.     0.     0.
TEXT C_NC_CC_O   C2n$1.5c[1c%(1.5o1c)1.5c(1c1.5c)]
===========================================================================
ATOM    N3_1     N     bXY   C   C   -    CCC      Nccc
NBOND   3  CYCL  0        CHIV  0.023   CHIR  0
SYMPLM  mymz      CONVAL  -  CONPLM  -  CONKAP  -
DIST    N-C  1.347   N-C  1.348   N-C  1.476
ANGL    C-N-C  121.8   C-N-C  120.0   C-N-C  118.2
KMD      0.99882 0.88540  4.979  0.     0.007  0.     0.
KMD_ESD  0.00236 0.04027  0.017  0.     0.014  0.     0.
QUA                -0.140  0.     0.    -0.012  0.
QUA_ESD                    0.012  0.     0.     0.014  0.
```

```
OCT       0.    0.035  0.    0.    0.    -0.220  0.
OCT_ESD    0.    0.010  0.    0.    0.    0.019  0.
TEXT N_CCC_CCHHHHH   N1c%[1.5c(1.5c1h)1h]1c$[1.5c(1.5c1h)1h]1c[1h1h1h]
==========================================================================ATOM
N4_1      N      ZX    C    C    -    C        Nc
NBOND   1  CYCL  0        CHIV  -       CHIR  0
SYMPLM   cy        CONVAL  -  CONPLM  -  CONKAP  -
DIST    N-C  1.156
ANGL
KMD     0.99185 0.98401  5.000  0.    0.    0.    -0.028
KMD_ESD  0.00237 0.05539  0.017  0.    0.    0.    0.013
QUA                  0.208  0.    0.    0.    0.
QUA_ESD                 0.014  0.    0.    0.    0.
OCT       0.019  0.    0.    0.    0.    0.    0.
OCT_ESD    0.012  0.    0.    0.    0.    0.    0.
TEXT N_C_C   N2c$[1.5c(1.5c1.5c%)]
==========================================================================
ATOM    C9_1      C     bXY    N    C    -    NCH       Cnch
NBOND   3  CYCL  0        CHIV  0.000    CHIR  0
SYMPLM   mz        CONVAL  -  CONPLM  -  CONKAP  -
DIST    C-N  1.347   C-C  1.381   C-H  1.083
ANGL    N-C-C  120.4   N-C-H  119.8   C-C-H  119.8
KMD     0.99438 0.81763  4.111  0.    0.    0.066  0.
KMD_ESD  0.00270 0.01767  0.017  0.    0.014  0.013  0.
QUA                 -0.291  0.    0.    0.025  0.086
QUA_ESD                 0.013  0.    0.    0.013  0.011
OCT       0.    -0.005 -0.011  0.    0.    -0.369  0.001
OCT_ESD    0.    0.010  0.011  0.    0.    0.018  0.014
TEXT C_NCH_CCCH   C1n$[1c(1.5c1h)1c(1h1h1h)]1.5c%[1.5c(1.5c1.5c)1h]1h
==========================================================================
ATOM    H9_1      H      ZX    C    N    -    C        Hc
NBOND   1  CYCL  0        CHIV  -       CHIR  0
SYMPLM   1         CONVAL  -  CONPLM  -  CONKAP  -
DIST    H-C  1.083
ANGL
KMD     1.09239 1.15731  0.803  0.    0.    0.    0.173
KMD_ESD  0.02235 0.03555  0.037  0.    0.    0.    0.009
TEXT H_C_NC   H1c$[1n%(1c1c)1.5c(1.5c1h)]
==========================================================================
ATOM    C10_1     C     bXY    C    C    -    CCH       Ccch
NBOND   3  CYCL  0        CHIV  0.000    CHIR  0
SYMPLM   mymz      CONVAL  -  CONPLM  -  CONKAP  -
DIST    C-C  1.381   C-C  1.393   C-H  1.083
ANGL    C-C-C  118.6   C-C-H  120.7   C-C-H  120.7
KMD     0.99960 0.92169  3.909  0.    -0.048  0.    0.
KMD_ESD  0.00289 0.02176  0.017  0.    0.014  0.    0.
QUA                 -0.241  0.    0.    0.026  0.
QUA_ESD                 0.011  0.    0.    0.012  0.
OCT       0.    0.034  0.    0.    0.    -0.303  0.
OCT_ESD    0.    0.009  0.    0.    0.    0.017  0.
TEXT C_CCH_NCCH_N   C1.5c$[1n(1c1c)1h]1.5c%[1.5c(2n)1.5c(1.5c1h)]1h
==========================================================================
ATOM    H10_1     H      ZX    C    C    -    C        Hc
NBOND   1  CYCL  0        CHIV  -       CHIR  0
SYMPLM   1         CONVAL  -  CONPLM  -  CONKAP  -
```

```
DIST    H-C  1.083
ANGL
KMD     1.14627 1.17910  0.728  0.    0.    0.    0.125
KMD_ESD  0.02575 0.05438  0.036  0.    0.    0.    0.013
TEXT H_C_CC_N   H1c$[1.5c%(1n1h)1.5c(1.5c1.5c)]
==============================================================
ATOM    C11_1     C     bXY   C   C   -    CCC      Cccc
NBOND   3  CYCL  0       CHIV  0.029   CHIR  0
SYMPLM  mymz      CONVAL  -  CONPLM  -  CONKAP  -
DIST    C-C  1.436   C-C  1.393   C-C  1.393
ANGL    C-C-C  120.7   C-C-C  119.1   C-C-C  120.1
KMD     0.99992 0.85301  4.011  0.    0.057  0.    0.
KMD_ESD  0.00337 0.02703  0.017  0.    0.014  0.    0.
QUA               -0.274  0.    0.    -0.026  0.
QUA_ESD              0.015  0.    0.    0.013  0.
OCT       0.    0.020  0.    0.    0.    -0.324  0.
OCT_ESD    0.    0.013  0.    0.    0.    0.023  0.
TEXT C_CCC_NCCHH_NN   C1.5c[2n]1.5c$[1.5c(1n1h)1h]1.5c%[1.5c(1n1h)1h]
==============================================================
ATOM    C14_1     C      ZX   N   H   -    NHHH      Cnhhh
NBOND   4  CYCL  0       CHIV  0.758   CHIR  0
SYMPLM  3m       CONVAL  -  CONPLM  -  CONKAP  -
DIST    C-N  1.476   C-H  1.077   C-H  1.077   C-H  1.077
ANGL    N-C-H  109.4   N-C-H  109.5   N-C-H  109.5   H-C-H  109.5   H-C-H  109.5   H-C-H  109.5
KMD     0.99696 0.90778  4.016  0.    0.    0.    -0.090
KMD_ESD  0.00348 0.03812  0.018  0.    0.    0.    0.017
QUA               -0.094  0.    0.    0.    0.
QUA_ESD              0.018  0.    0.    0.    0.
OCT       0.257  0.    0.    0.    0.    0.106  0.
OCT_ESD    0.022  0.    0.    0.    0.    0.019  0.
TEXT C_NHHH_CC   C1n$[1c(1.5c1h)1c(1.5c1h)]1h1h%1h
==============================================================
ATOM    H14A_1    H      ZX   C   N   -    C       Hc
NBOND   1  CYCL  0       CHIV  -      CHIR  0
SYMPLM  1       CONVAL  -  CONPLM  -  CONKAP  -
DIST    H-C  1.077
ANGL
KMD     1.07472 1.14788  0.972  0.    0.    0.    0.173
KMD_ESD  0.01609 0.03622  0.028  0.    0.    0.    0.
TEXT H_C_NHH   H1c$[1n%(1c1c)1h1h]
==============================================================
ATOM    H14B_1    H      ZX   C   N   -    C       Hc
NBOND   1  CYCL  0       CHIV  -      CHIR  0
SYMPLM  1       CONVAL  -  CONPLM  -  CONKAP  -
DIST    H-C  1.077
ANGL
KMD     1.07472 1.14788  0.972  0.    0.    0.    0.173
KMD_ESD  0.    0.    0.    0.    0.    0.    0.
TEXT H_C_NHH   H1c$[1n%(1c1c)1h1h]
==============================================================
ATOM    H14C_1    H      ZX   C   N   -    C       Hc
NBOND   1  CYCL  0       CHIV  -      CHIR  0
SYMPLM  1       CONVAL  -  CONPLM  -  CONKAP  -
DIST    H-C  1.077
ANGL
```

```
KMD      1.07472 1.14788  0.972  0.     0.     0.     0.173
KMD_ESD  0.      0.       0.     0.     0.     0.     0.
TEXT H_C_NHH   H1c$[1n%(1c1c)1h1h]
============================================================
ATOM     C15_1      C       ZX    N    C    -     NC        Cnc
NBOND    2   CYCL  0        CHIV  -       CHIR  0
SYMPLM   cy        CONVAL  -  CONPLM  -  CONKAP  -
DIST     C-N  1.156   C-C  1.436
ANGL     N-C-C  176.5
KMD      1.01807 0.83637  4.109  0.     0.     0.     0.258
KMD_ESD  0.00359 0.02535  0.017  0.     0.     0.     0.
QUA                       0.409  0.     0.     0.     0.
QUA_ESD                          0.     0.     0.     0.     0.
OCT       -0.012  0.     0.     0.     0.     0.     0.
OCT_ESD    0.     0.     0.     0.     0.     0.     0.
TEXT C_NC_CC   C2n$1.5c[1.5c%(1.5c1h)1.5c(1.5c1h)]
============================================================
```

S4 Residual densities at variable temperatures (spherical and multipolar)

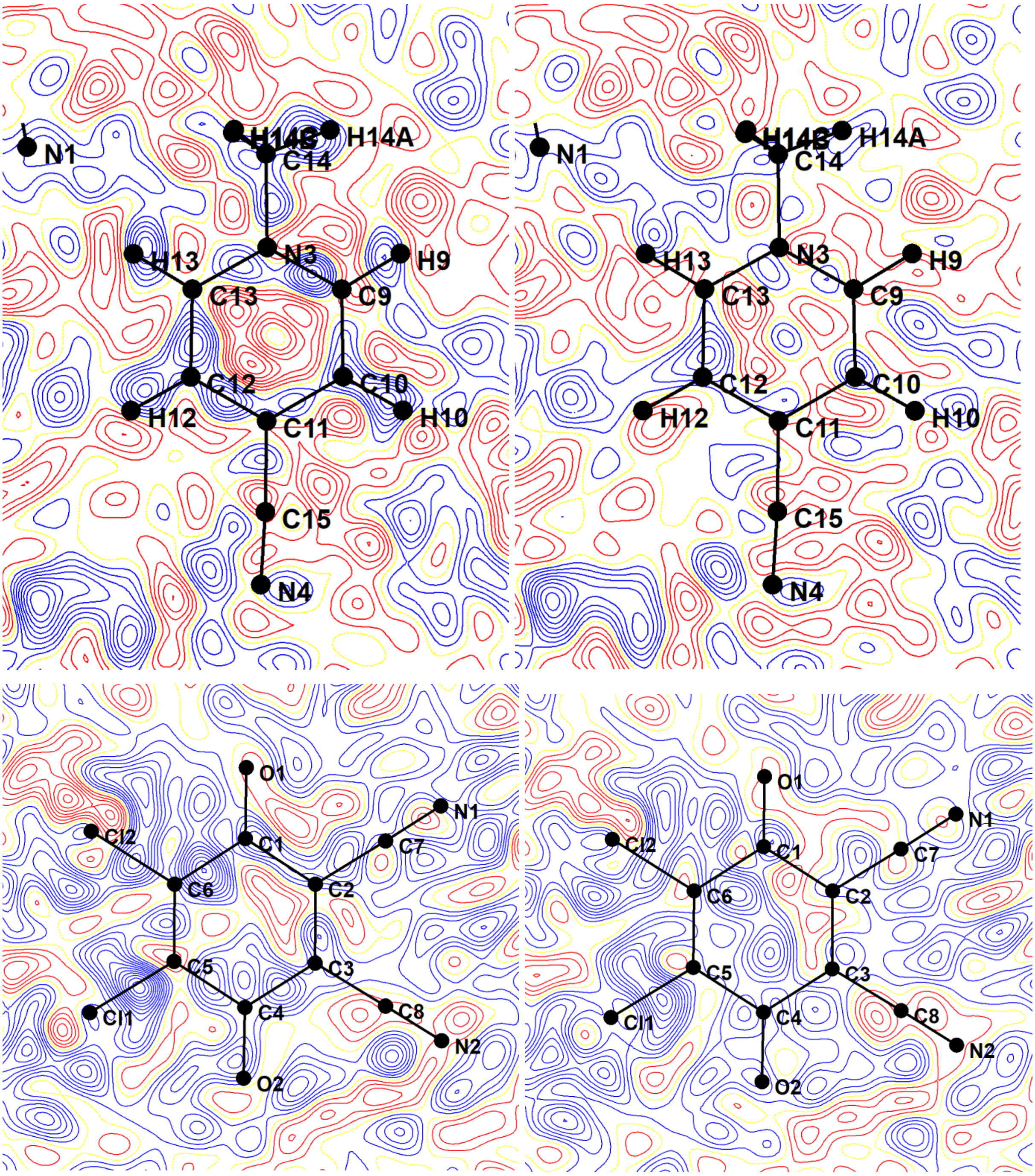

Figure S5 Residual density in the mean plane of 4-cyano-*N*-methylpyridinium cation (top row) and DDQ radical anion (bottom row) at 90 K. Left: spherical refinement, right: transferred-multipole refinement. Positive density is shown in blue and negative in red; yellow dotted lines represent zero density. Contours are drawn for 0.05 eÅ^{-1}.

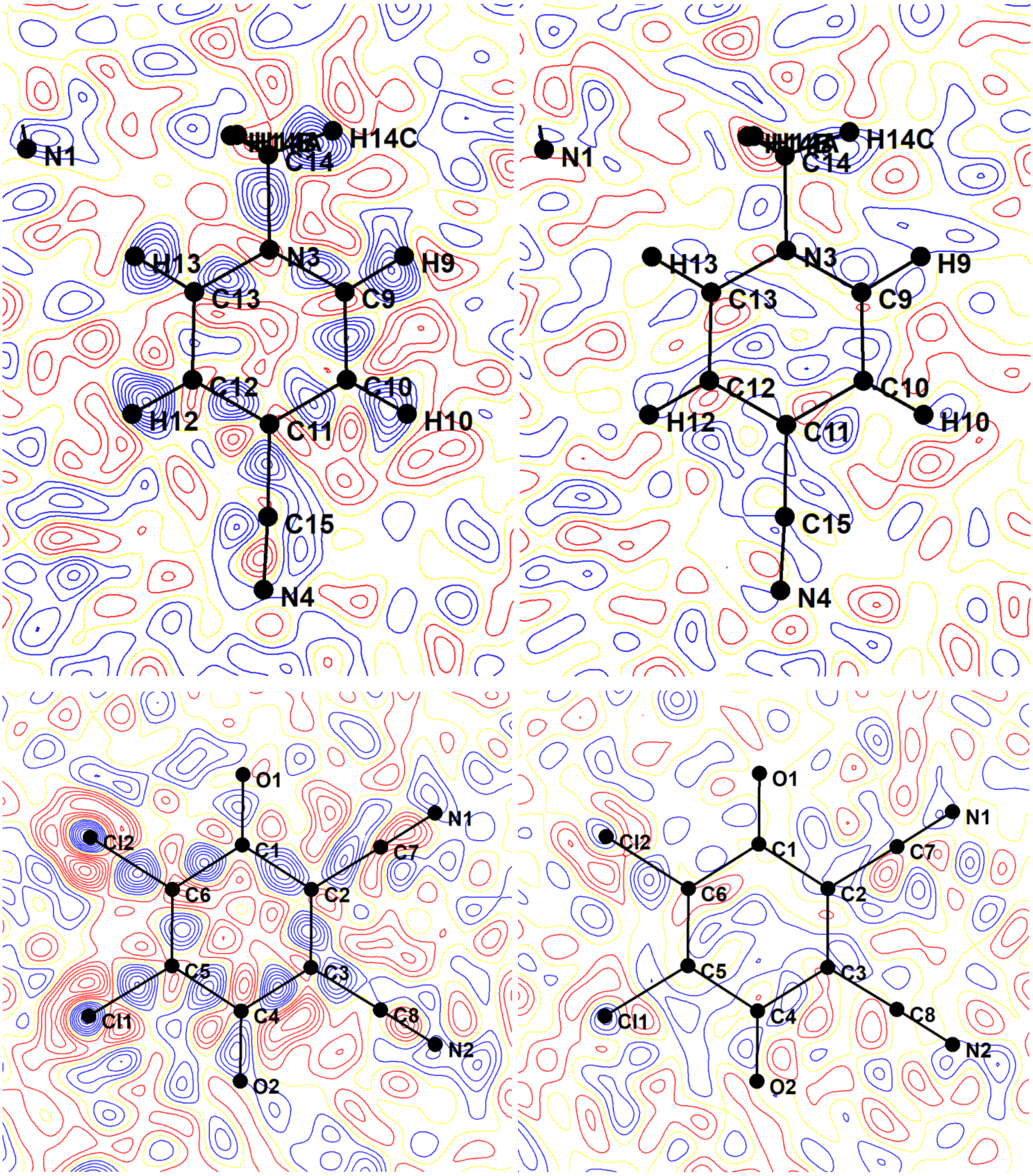

Figure S6 Residual density in the mean plane of 4-cyano-*N*-methylpyridinium cation (top row) and DDQ radical anion (bottom row) at 120 K. Left: spherical refinement, right: transferred-multipole refinement. Positive density is shown in blue and negative in red; yellow dotted lines represent zero density. Contours are drawn for 0.05 eÅ^{-1}.

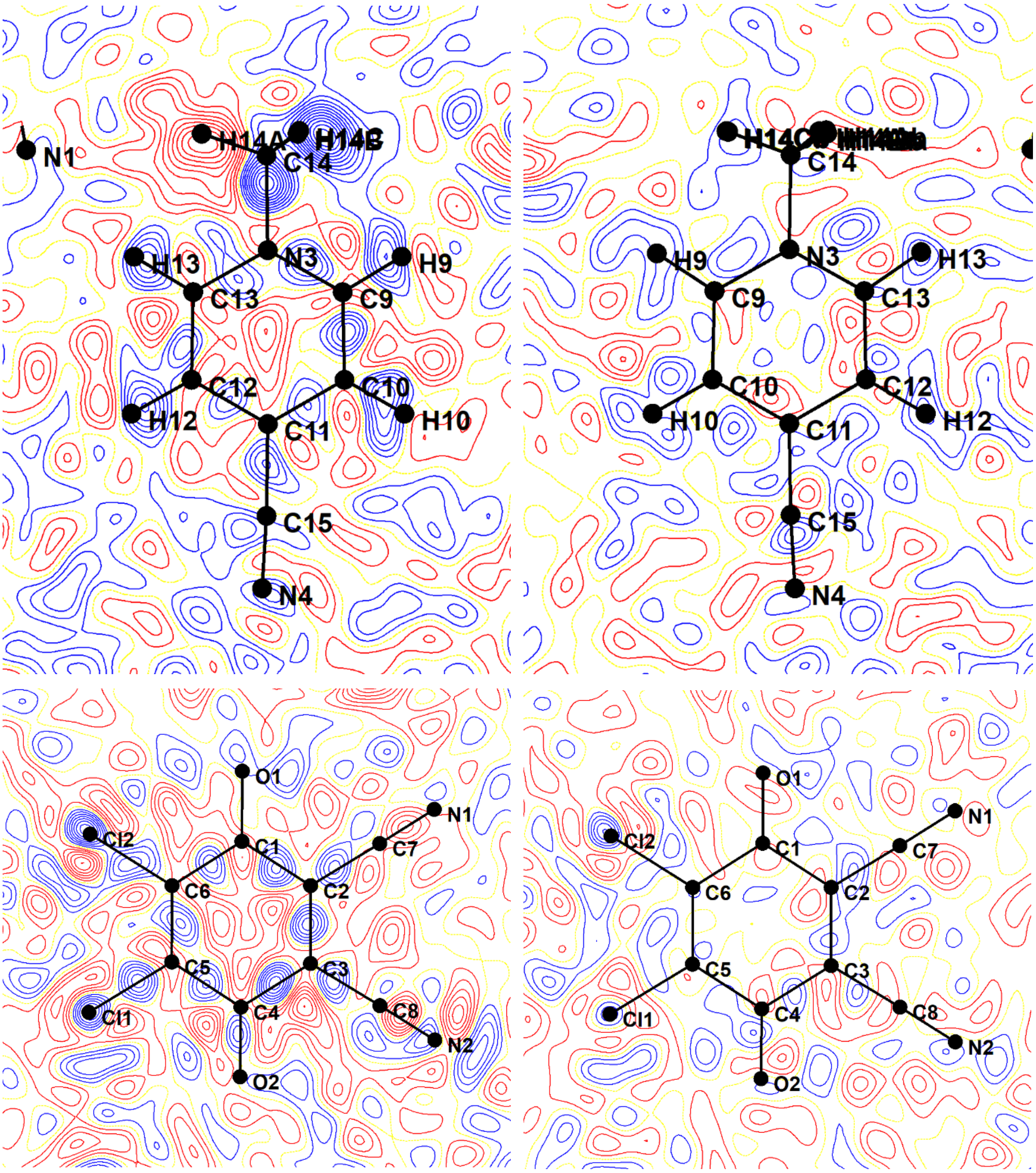

Figure S7 Residual density in the mean plane of 4-cyano-*N*-methylpyridinium cation (top row) and DDQ radical anion (bottom row) at 150 K. Left: spherical refinement, right: transferred-multipole refinement. Positive density is shown in blue and negative in red; yellow dotted lines represent zero density. Contours are drawn for 0.05 eÅ^{-1}.

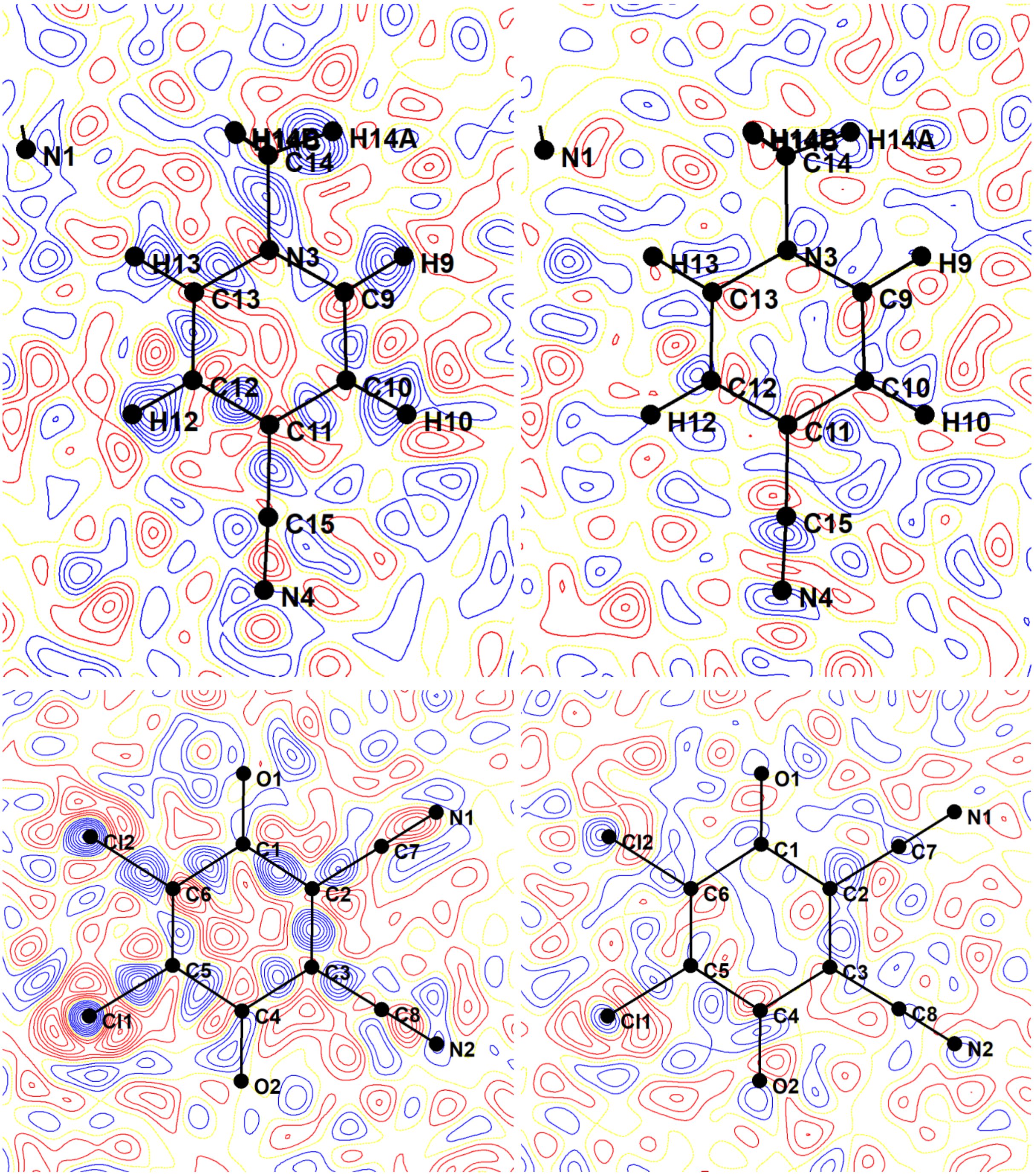

Figure S8 Residual density in the mean plane of 4-cyano-*N*-methylpyridinium cation (top row) and DDQ radical anion (bottom row) at 180 K. Left: spherical refinement, right: transferred-multipole refinement. Positive density is shown in blue and negative in red; yellow dotted lines represent zero density. Contours are drawn for 0.05 eÅ^{-1}.

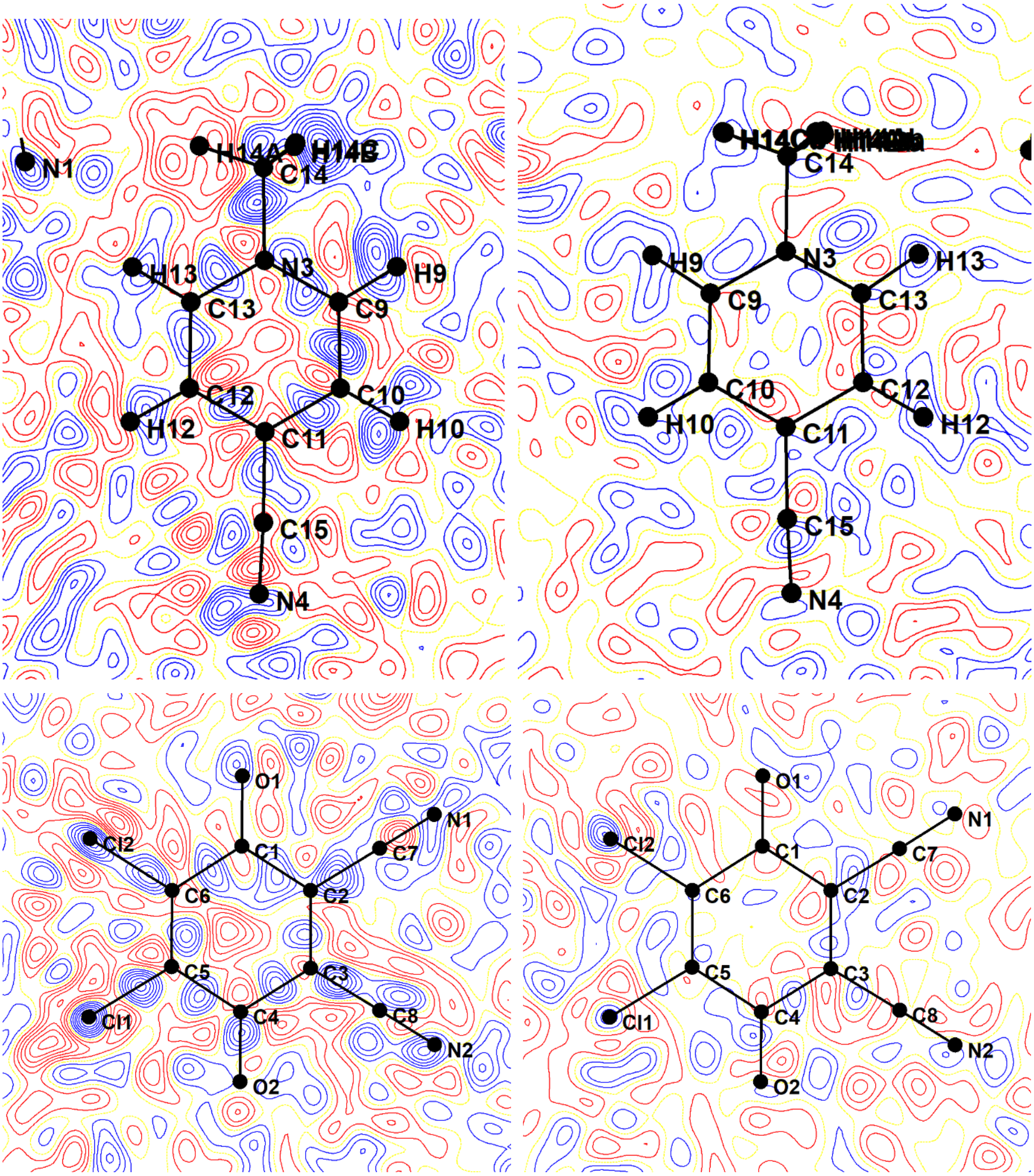

Figure S9 Residual density in the mean plane of 4-cyano-*N*-methylpyridinium cation (top row) and DDQ radical anion (bottom row) at 210 K. Left: spherical refinement, right: transferred-multipole refinement. Positive density is shown in blue and negative in red; yellow dotted lines represent zero density. Contours are drawn for 0.05 eÅ^{-1}.

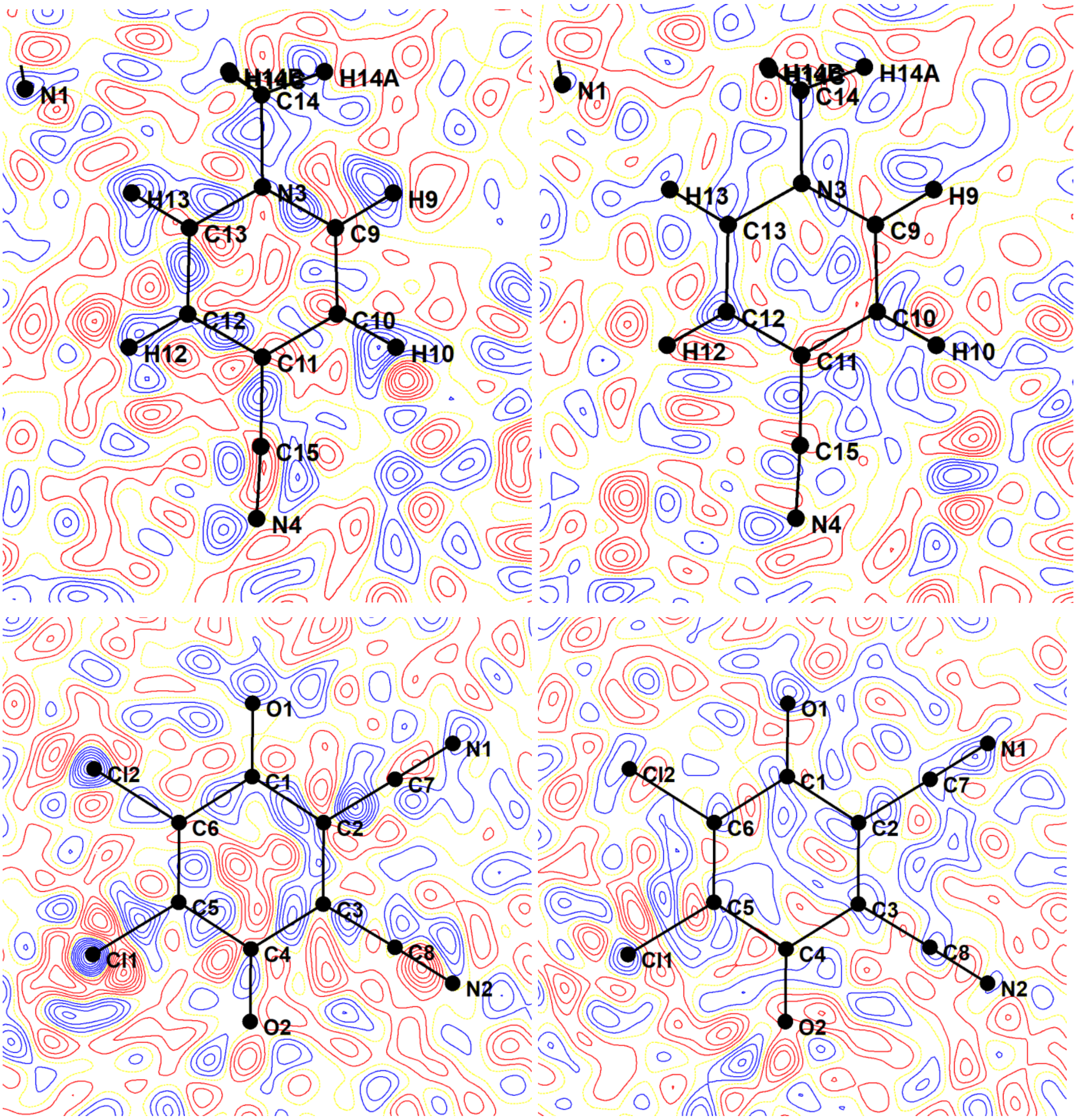

Figure S10 Residual density in the mean plane of 4-cyano-*N*-methylpyridinium cation (top row) and DDQ radical anion (bottom row) at 240 K. Left: spherical refinement, right: transferred-multipole refinement. Positive density is shown in blue and negative in red; yellow dotted lines represent zero density. Contours are drawn for 0.05 eÅ^{-1}.

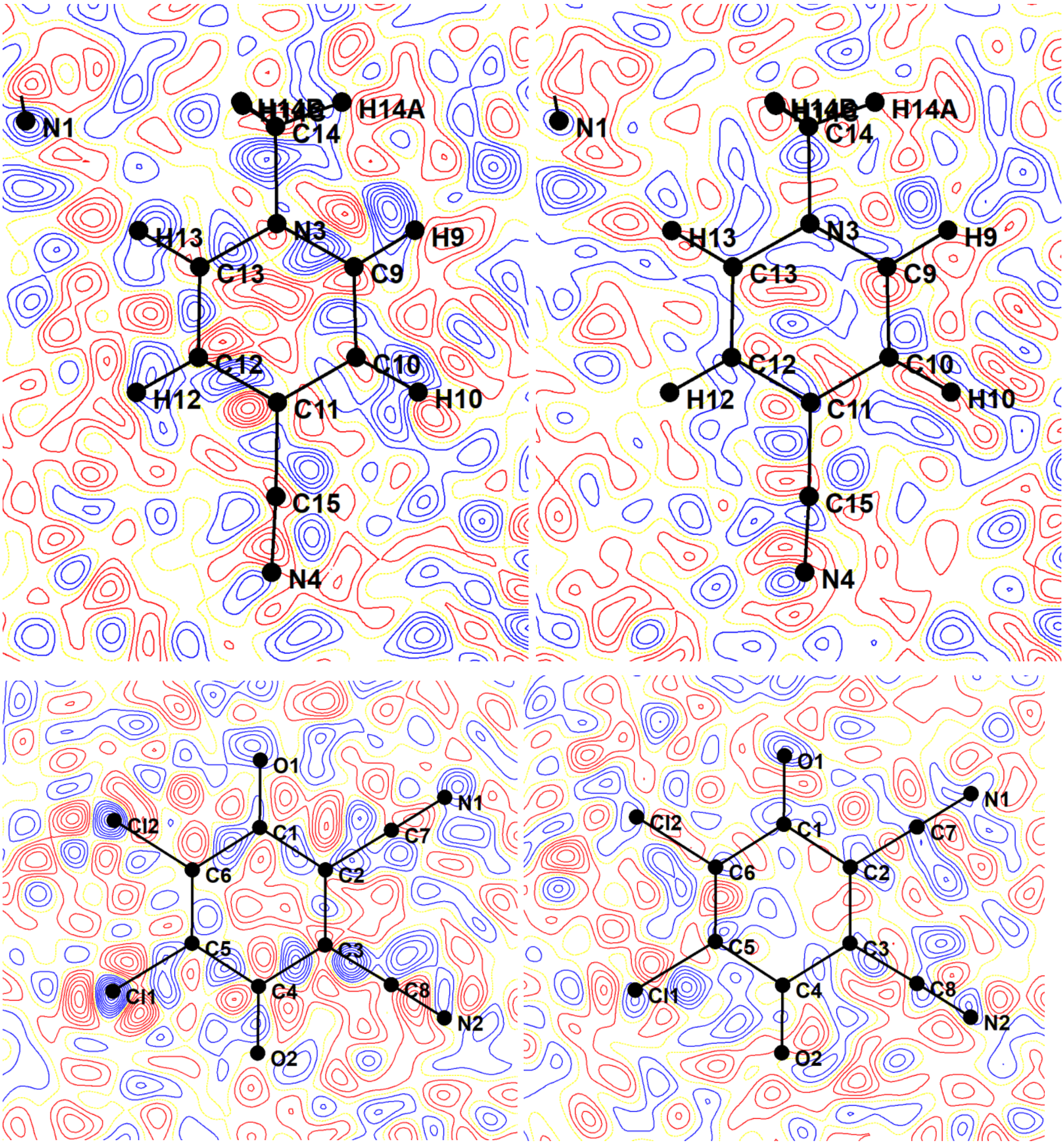

Figure S11 Residual density in the mean plane of 4-cyano-*N*-methylpyridinium cation (top row) and DDQ radical anion (bottom row) at 270 K. Left: spherical refinement, right: transferred-multipole refinement. Positive density is shown in blue and negative in red; yellow dotted lines represent zero density. Contours are drawn for 0.05 eÅ^{-1}.

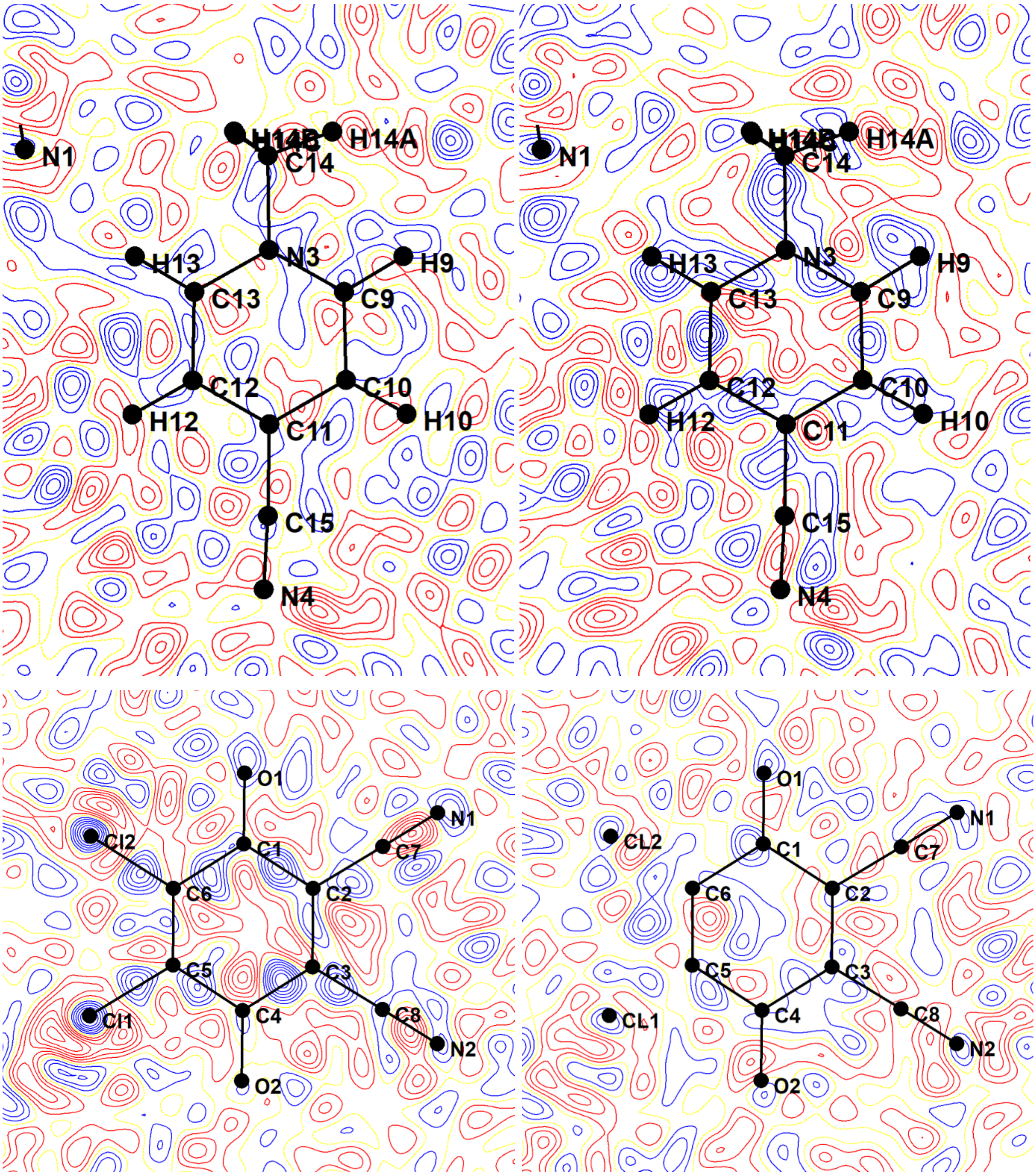

Figure S12 Residual density in the mean plane of 4-cyano-*N*-methylpyridinium cation (top row) and DDQ radical anion (bottom row) at 273 K. Left: spherical refinement, right: transferred-multipole refinement. Positive density is shown in blue and negative in red; yellow dotted lines represent zero density. Contours are drawn for 0.05 eÅ^{-1}.

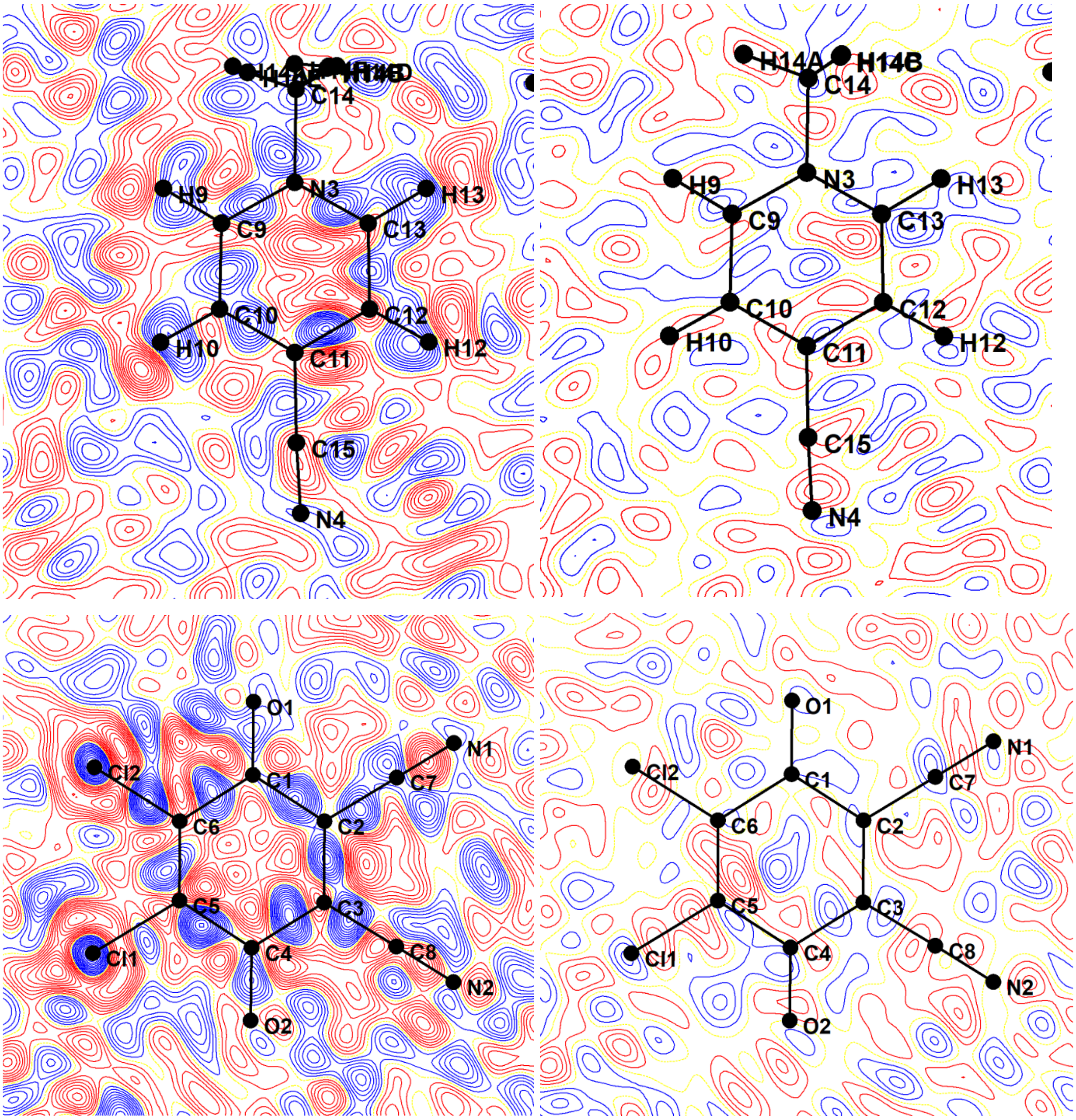

Figure S13 Residual density in the mean plane of 4-cyano-*N*-methylpyridinium cation (top row) and DDQ radical anion (bottom row) at 310 K. Left: spherical refinement, right: transferred-multipole refinement. Positive density is shown in blue and negative in red; yellow dotted lines represent zero density. Contours are drawn for 0.05 eÅ^{-1}.

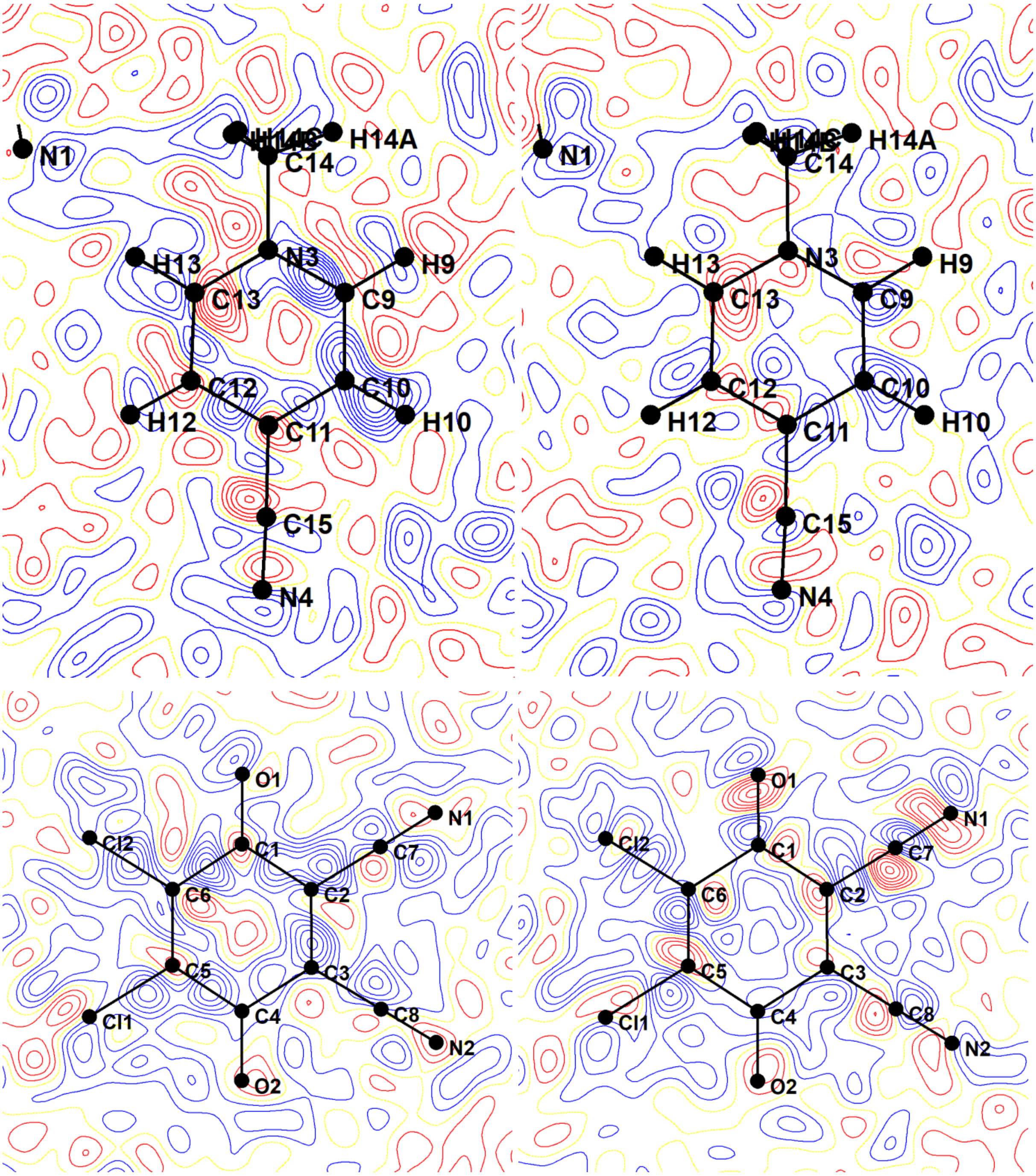

Figure S14 Residual density in the mean plane of 4-cyano-*N*-methylpyridinium cation (top row) and DDQ radical anion (bottom row) at 340 K. Left: spherical refinement, right: transferred-multipole refinement. Positive density is shown in blue and negative in red; yellow dotted lines represent zero density. Contours are drawn for 0.05 eÅ^{-1}.

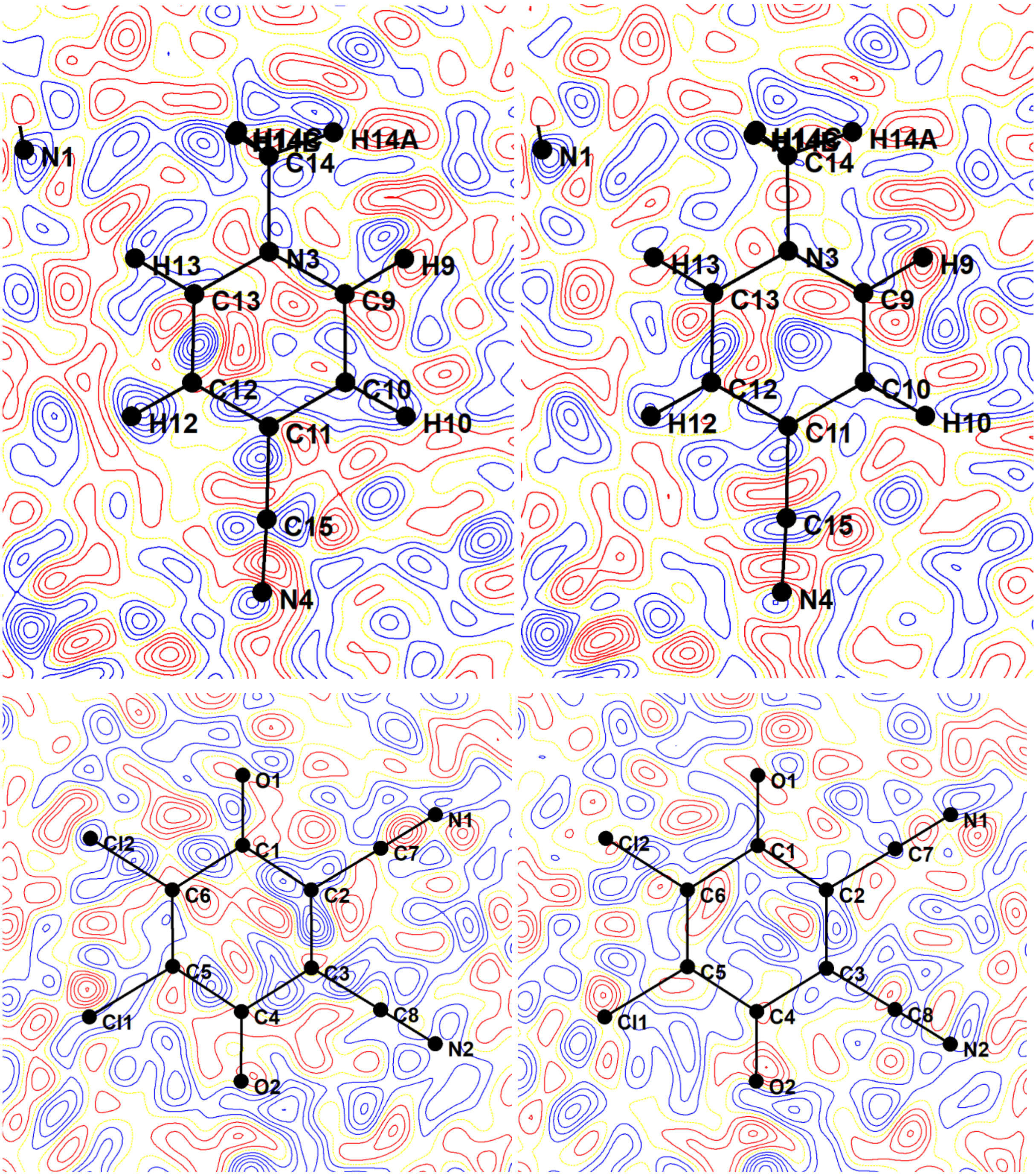

Figure S15 Residual density in the mean plane of 4-cyano-*N*-methylpyridinium cation (top row) and DDQ radical anion (bottom row) at 370 K. Left: spherical refinement, right: transferred-multipole refinement. Positive density is shown in blue and negative in red; yellow dotted lines represent zero density. Contours are drawn for 0.05 eÅ^{-1}.

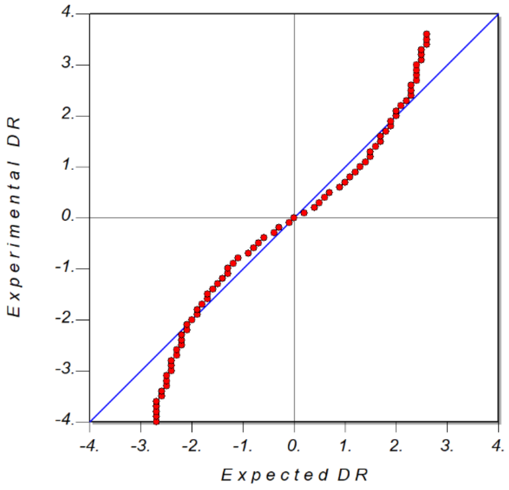

90 K

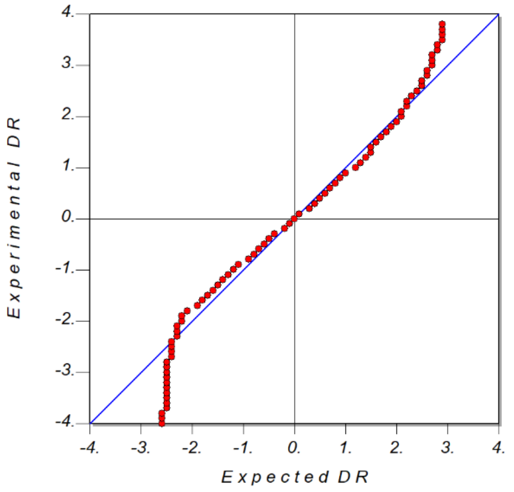

120 K

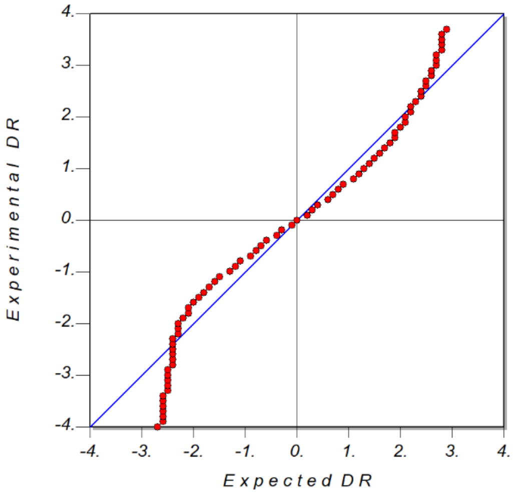

150 K

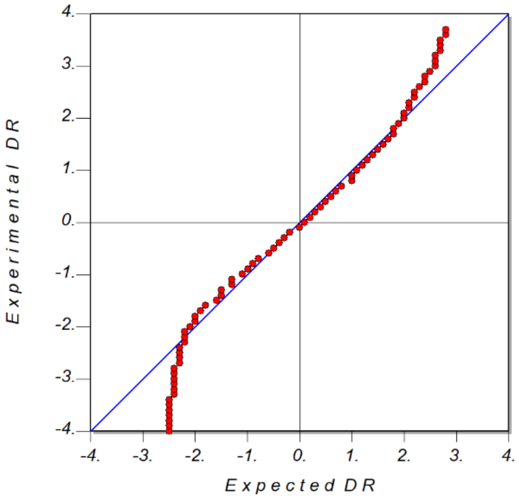

180 K

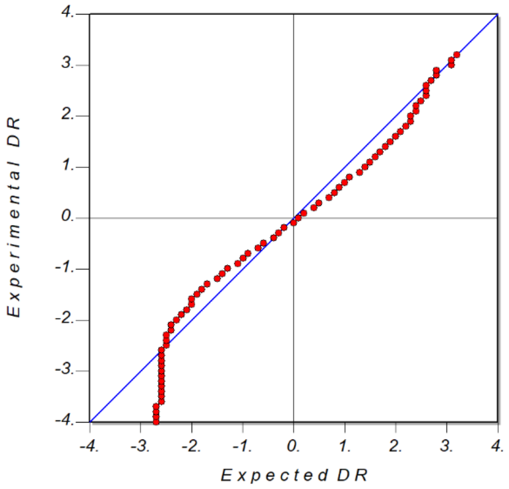

210 K

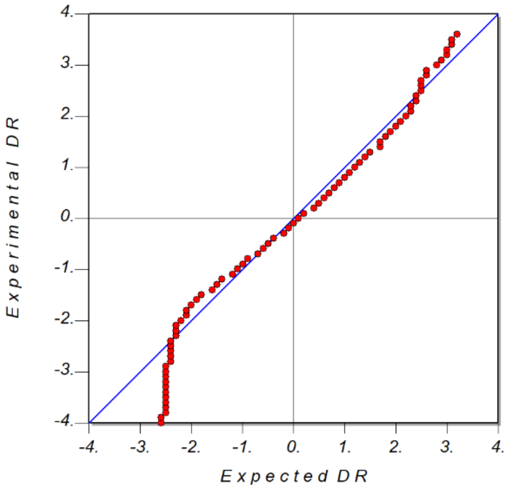

240 K

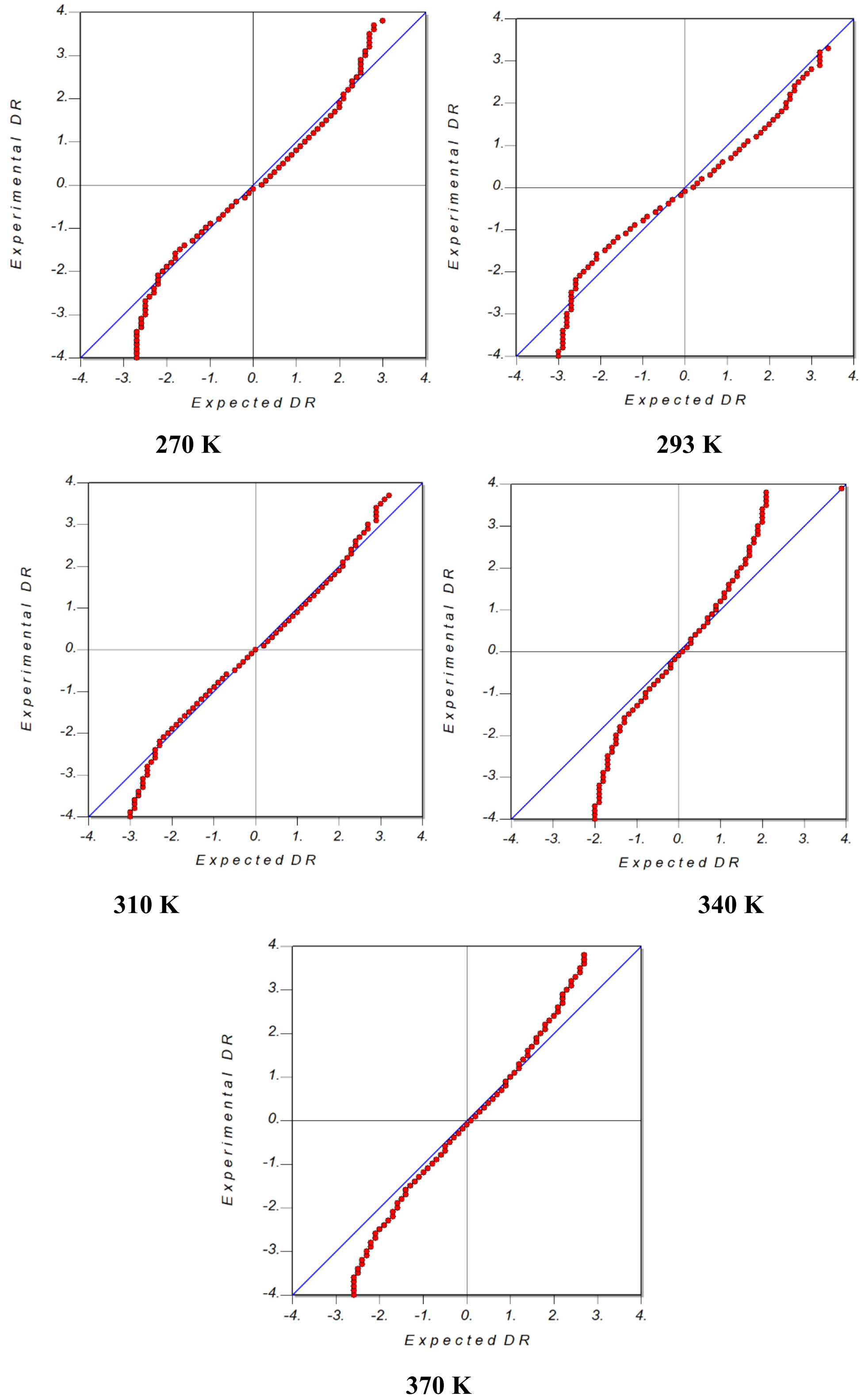

Figure S16 XDRK plot showing the expected and experimental Y_{obs}- Y_{calc} data profile for variable-temperature data.

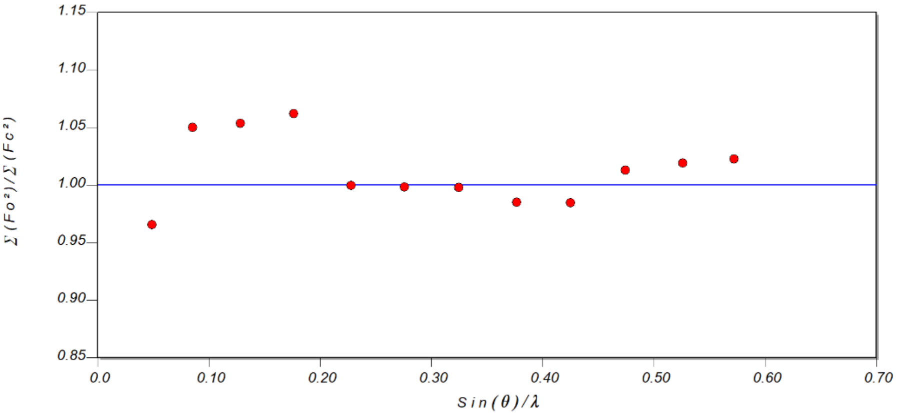

90 K

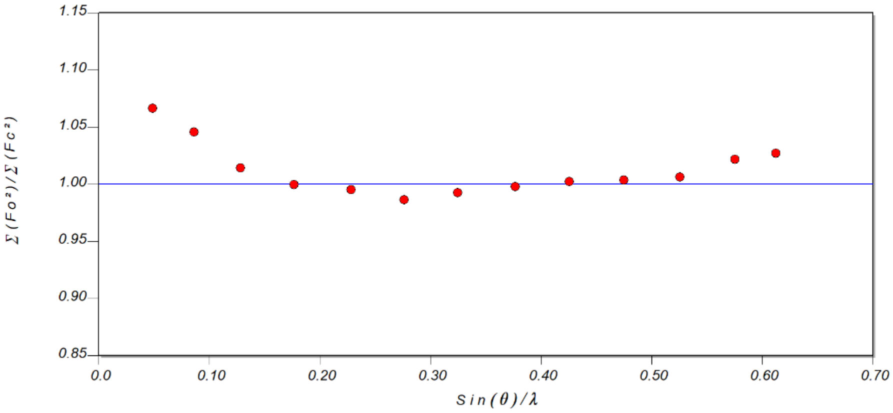

120 K

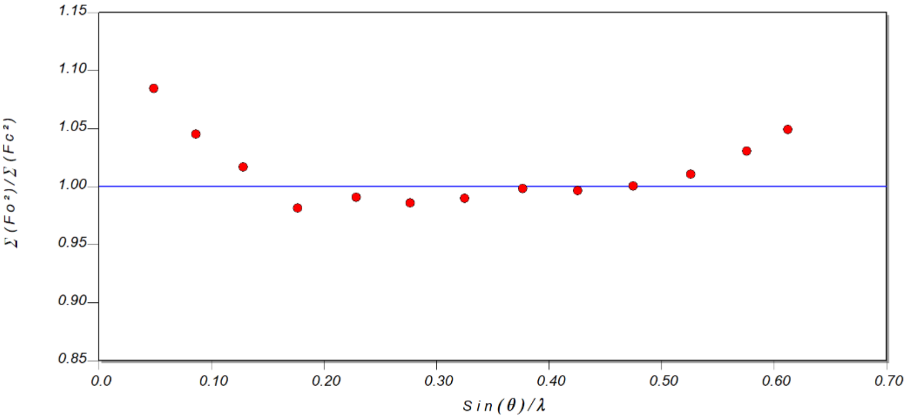

150 K

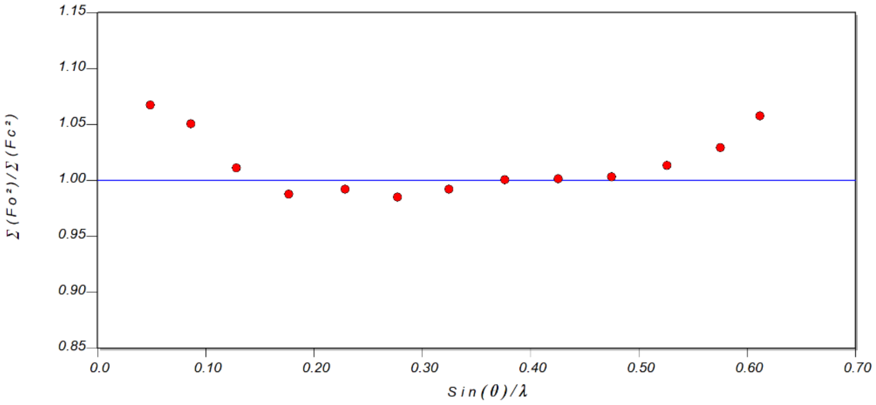

180 K

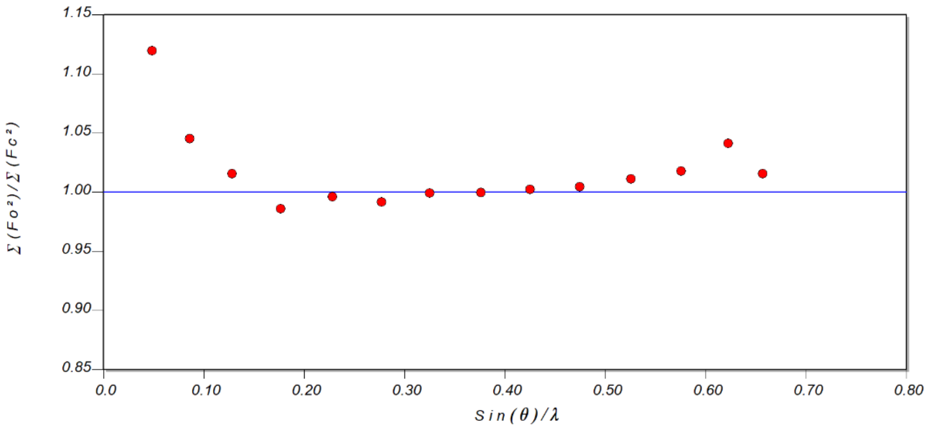

210 K

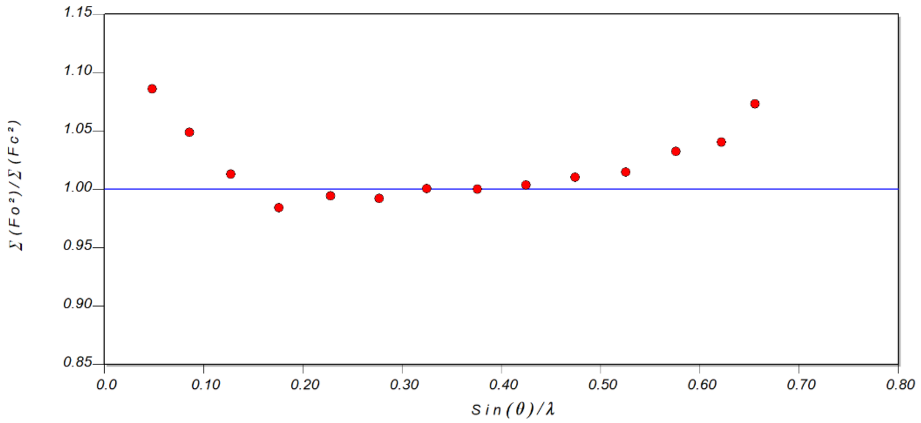

240 K

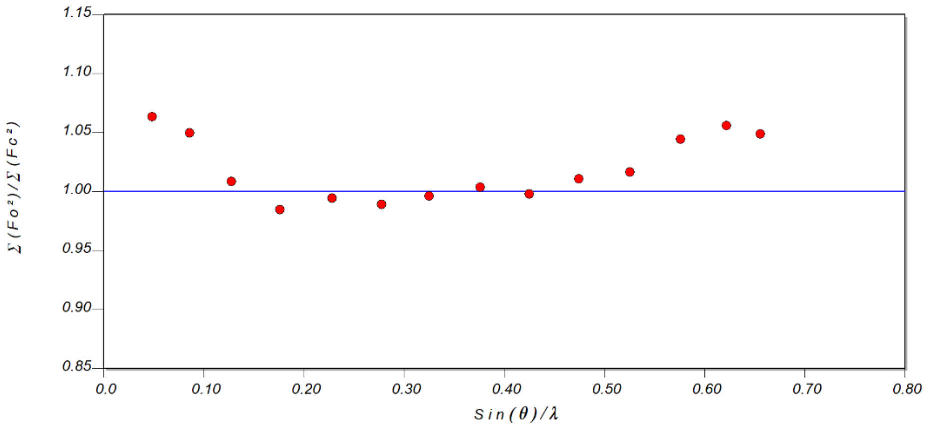

270 K

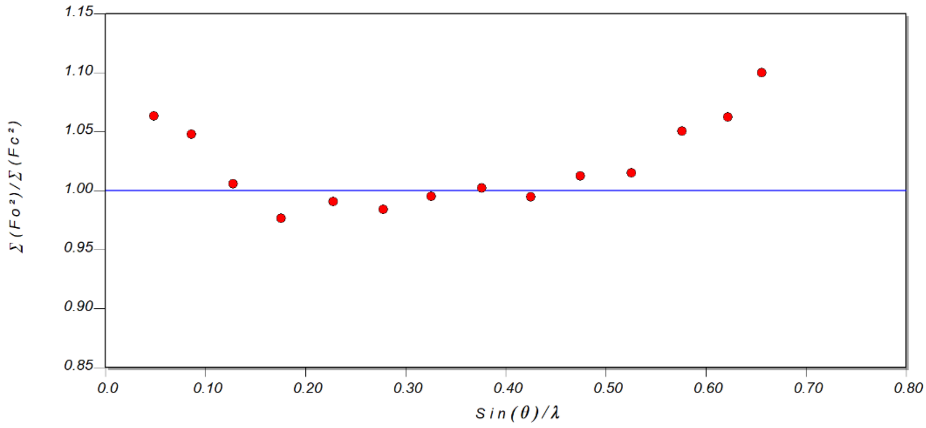

293 K

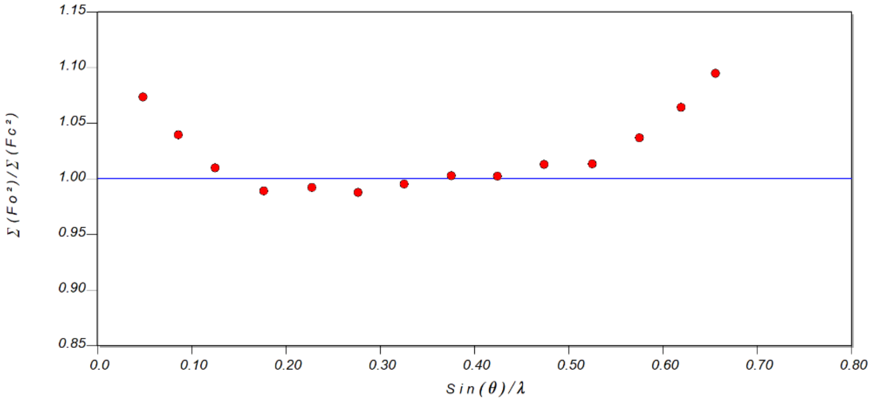

310 K

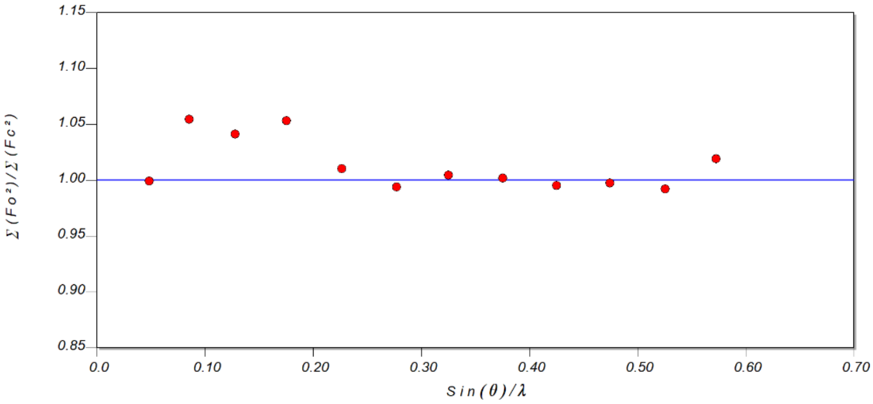

340 K

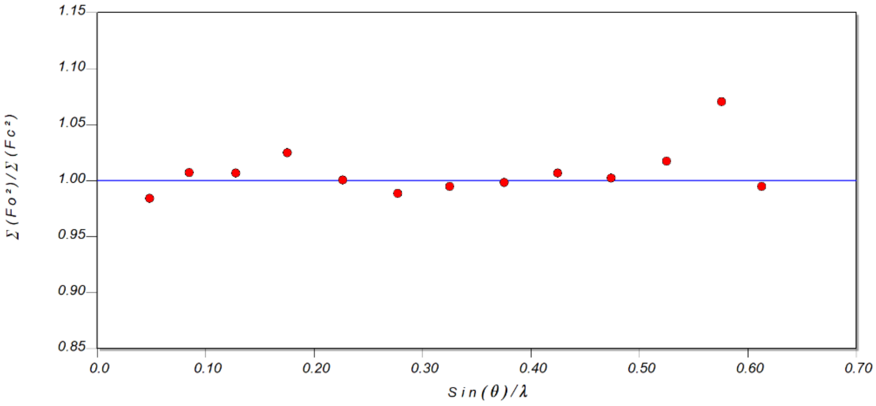

370 K

Figure S17 XDRK plot showing the fit of $<Y_{obs}>$ *vs* $<Y_{calc}>$ as a function of resolution for variable-temperature data.

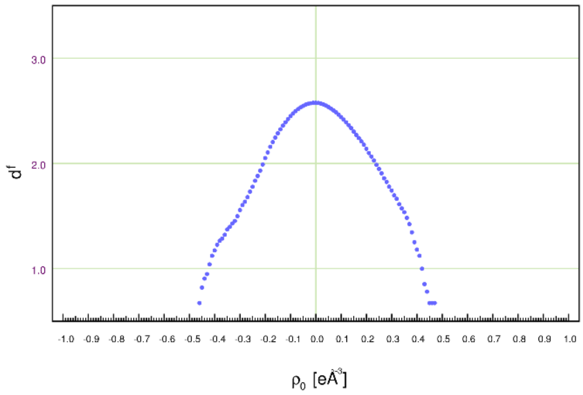

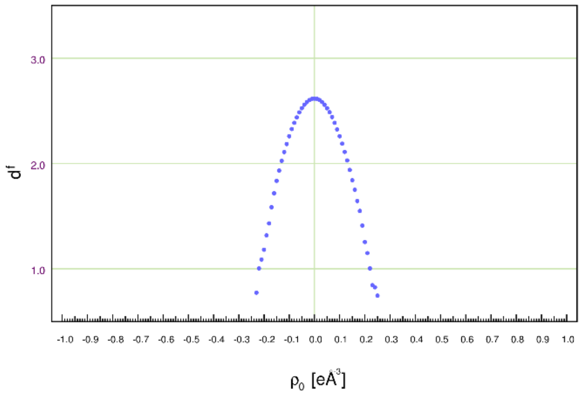

90 K

120K

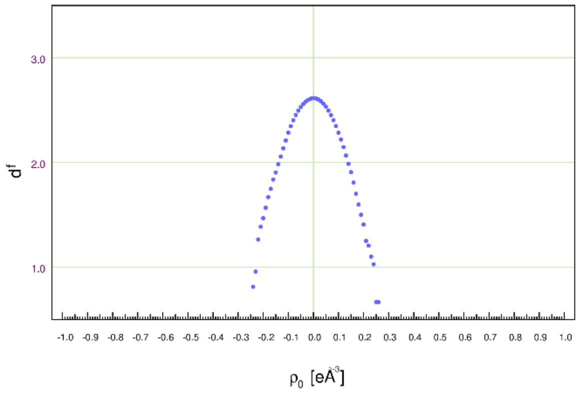

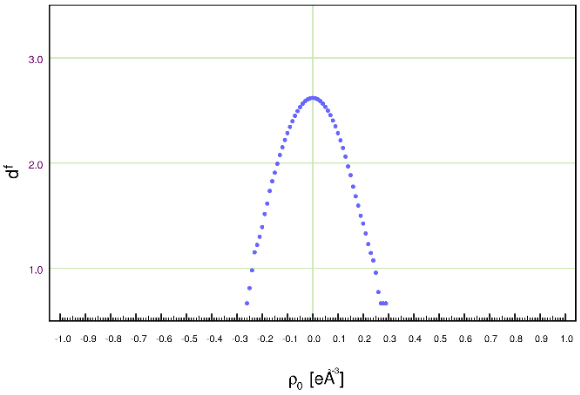

150 K

180 K

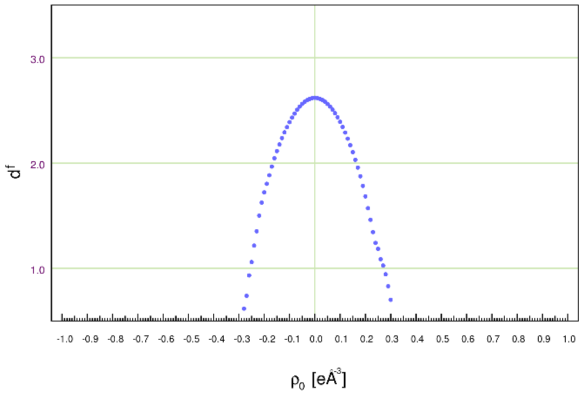

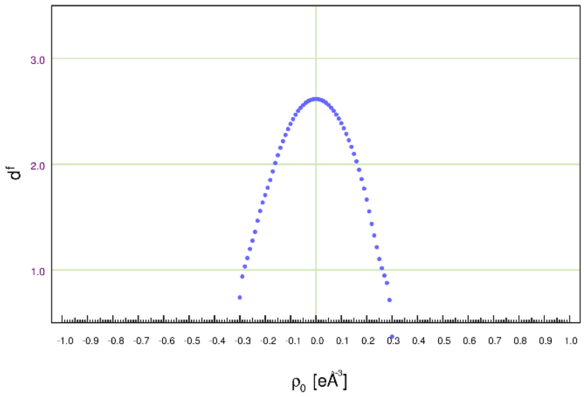

210 K

240 K

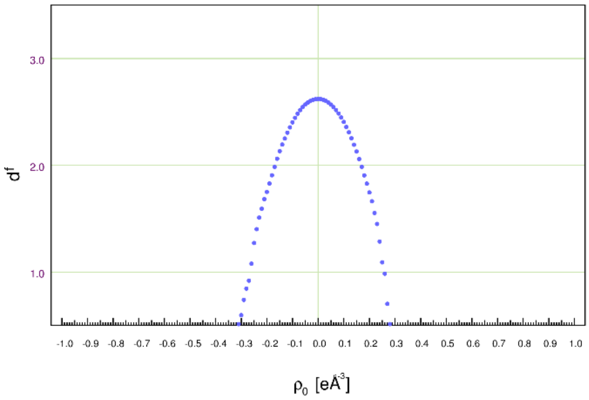

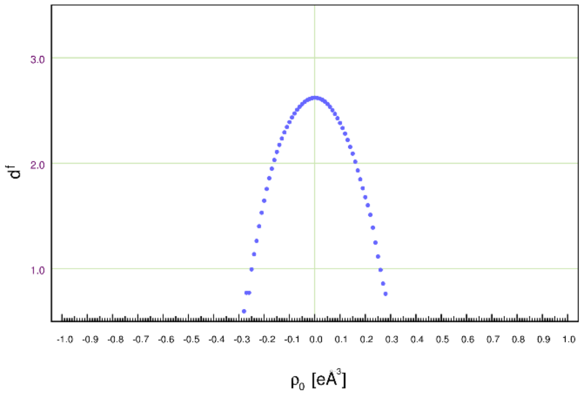

270 K **293 K**

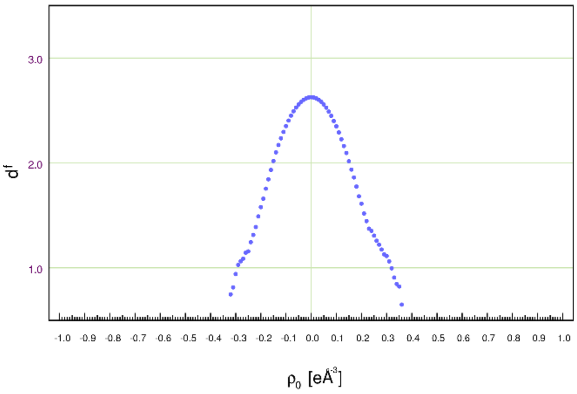

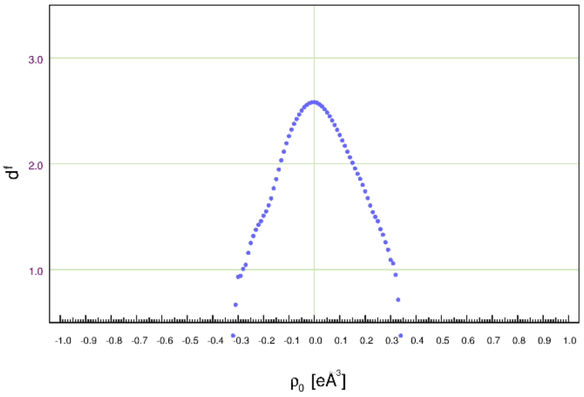

310 K **340 K**

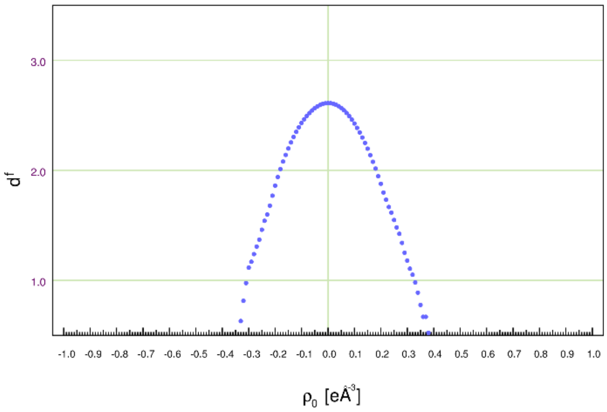

370 K

Figure S18 Fractal dimension plots of residual density [$(Y_{obs}$-$Y_{calc})/\sigma_{Yobs}$] for variable-temperature data. The plot was prepared by the program *jnk2rda* [4].

S5 Residual densities at high pressures (spherical and multipolar)

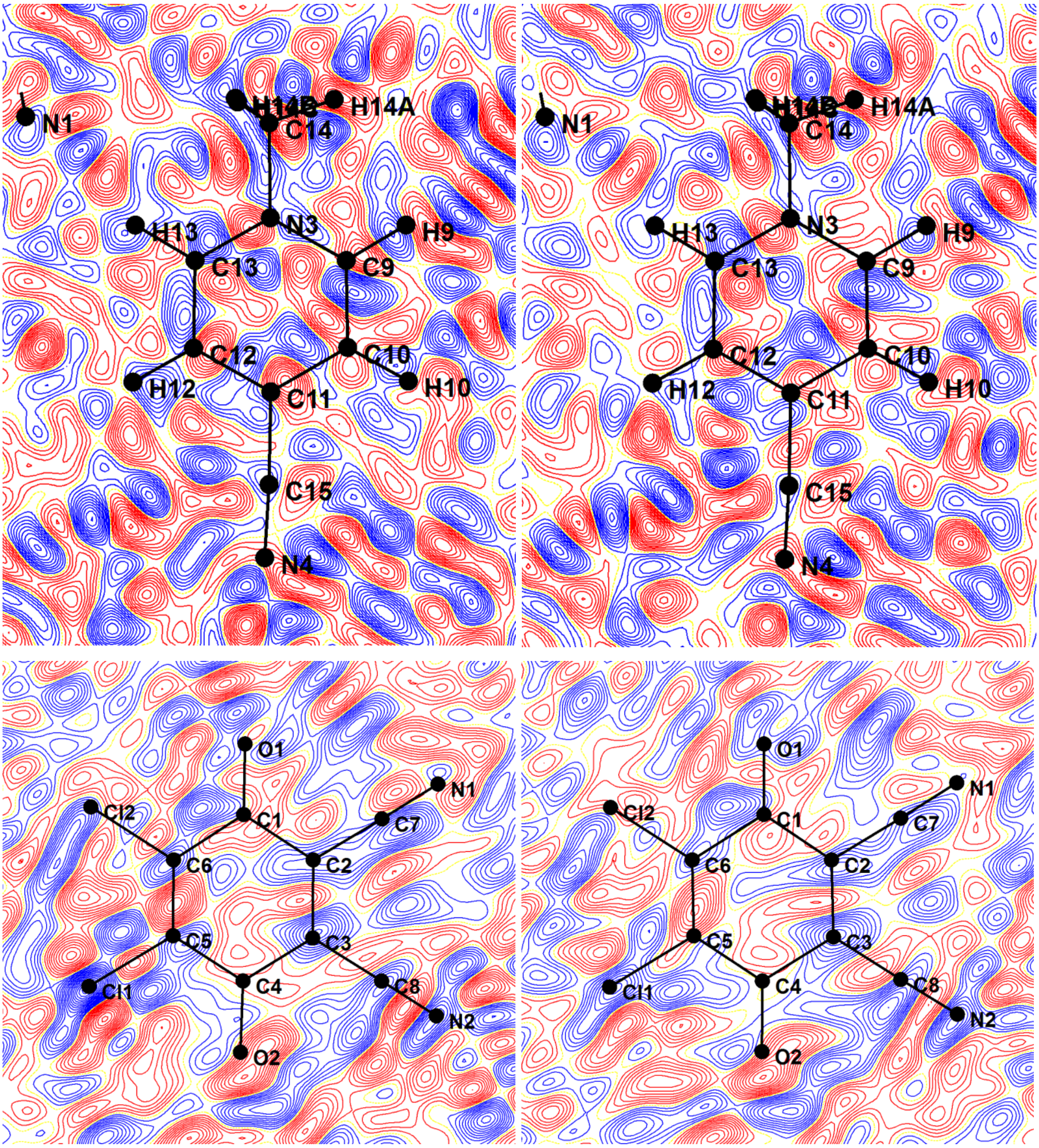

Figure S19 Residual density in the mean plane of 4-cyano-*N*-methylpyridinium cation (top row) and DDQ radical anion (bottom row) at 0.25 GPa. Left: spherical refinement, right: transferred-multipole refinement. Positive density is shown in blue and negative in red; yellow dotted lines represent zero density. Contours are drawn for 0.05 eÅ^{-1}.

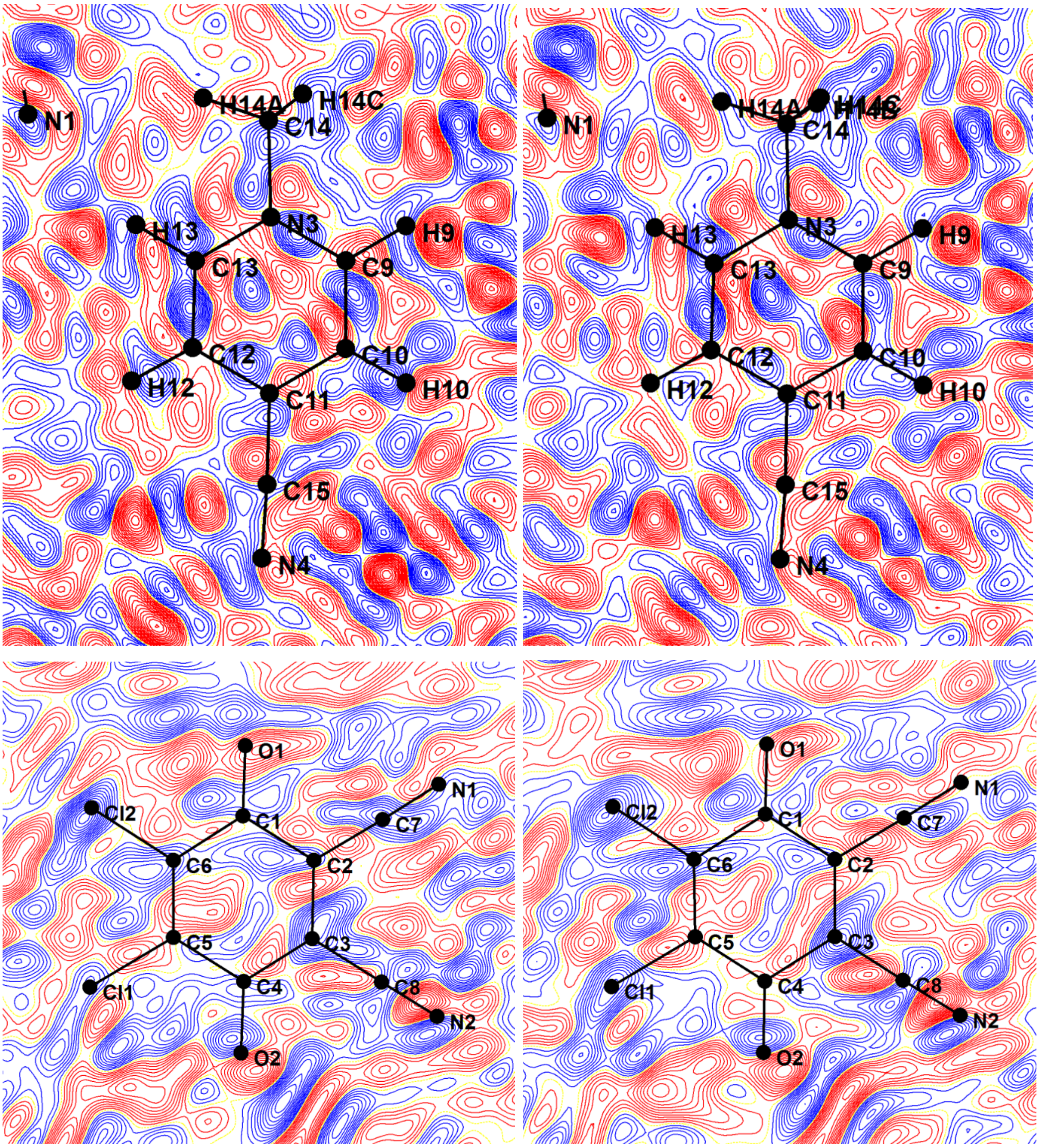

Figure S20 Residual density in the mean plane of 4-cyano-*N*-methylpyridinium cation (top row) and DDQ radical anion (bottom row) at 0.49 GPa. Left: spherical refinement, right: transferred-multipole refinement. Positive density is shown in blue and negative in red; yellow dotted lines represent zero density. Contours are drawn for 0.05 eÅ^{-1}.

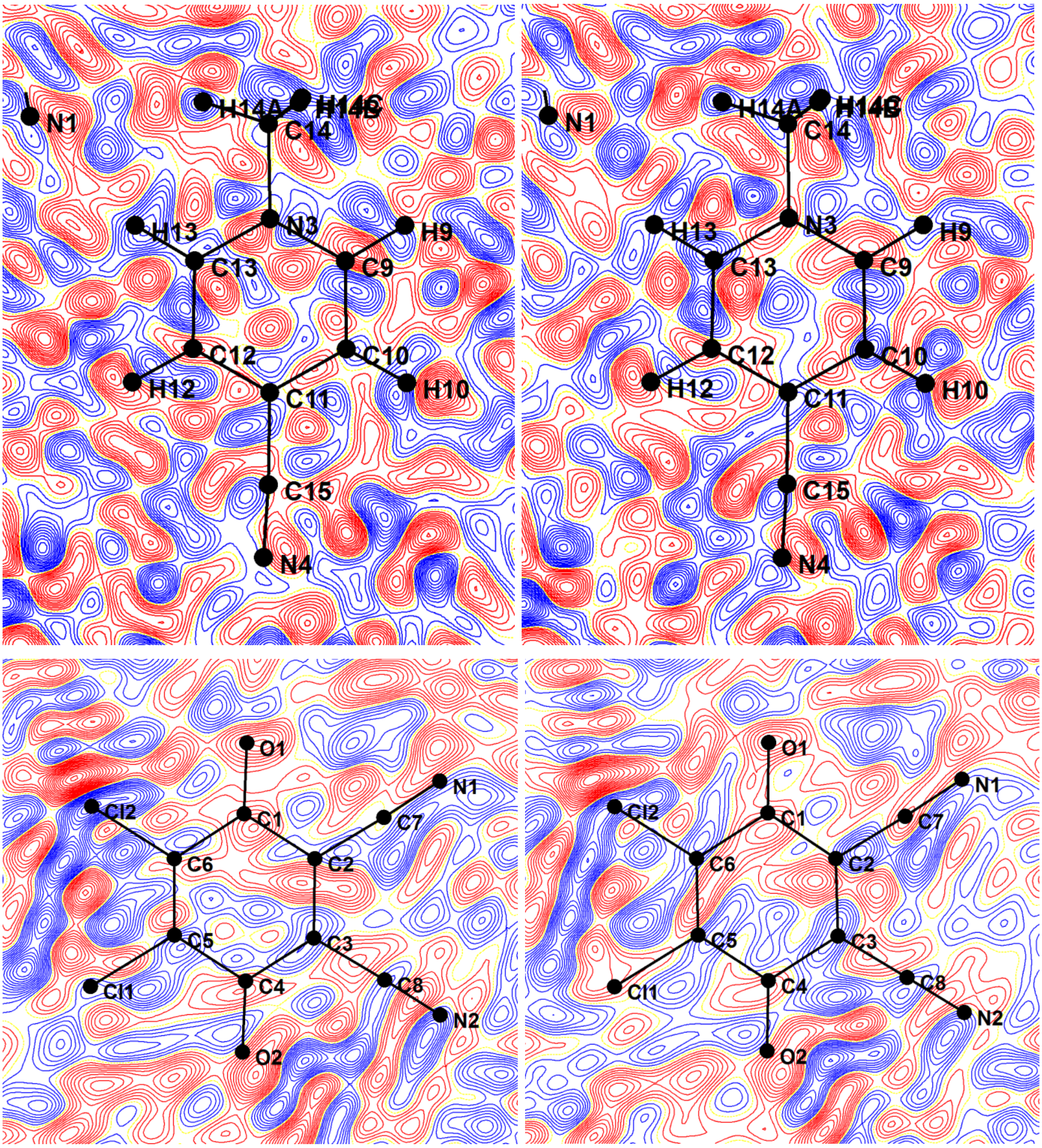

Figure S21 Residual density in the mean plane of 4-cyano-*N*-methylpyridinium cation (top row) and DDQ radical anion (bottom row) at 0.86 GPa. Left: spherical refinement, right: transferred-multipole refinement. Positive density is shown in blue and negative in red; yellow dotted lines represent zero density. Contours are drawn for 0.05 eÅ^{-1}.

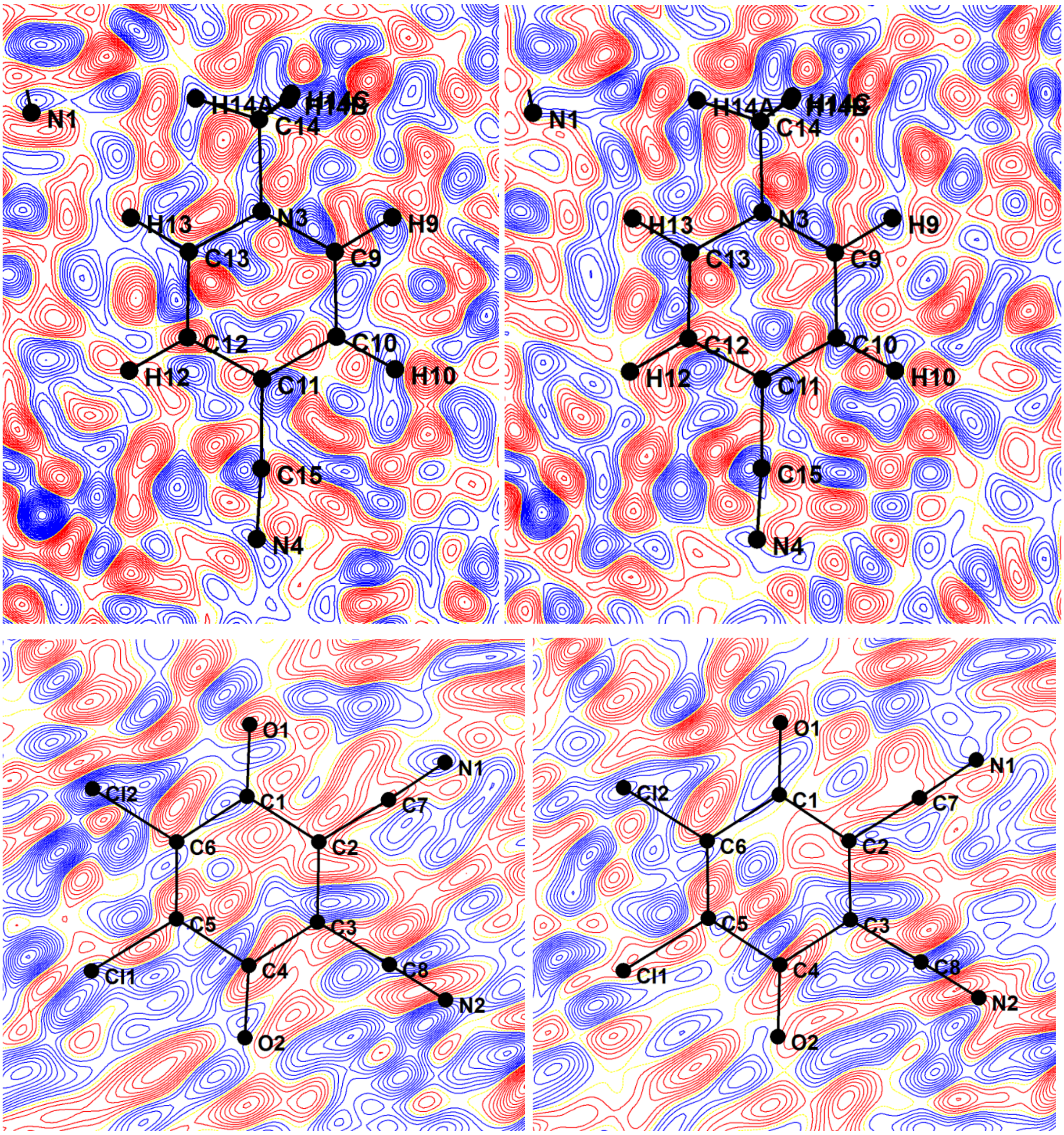

Figure S22 Residual density in the mean plane of 4-cyano-*N*-methylpyridinium cation (top row) and DDQ radical anion (bottom row) at 1.42 GPa. Left: spherical refinement, right: transferred-multipole refinement. Positive density is shown in blue and negative in red; yellow dotted lines represent zero density. Contours are drawn for 0.05 eÅ^{-1}.

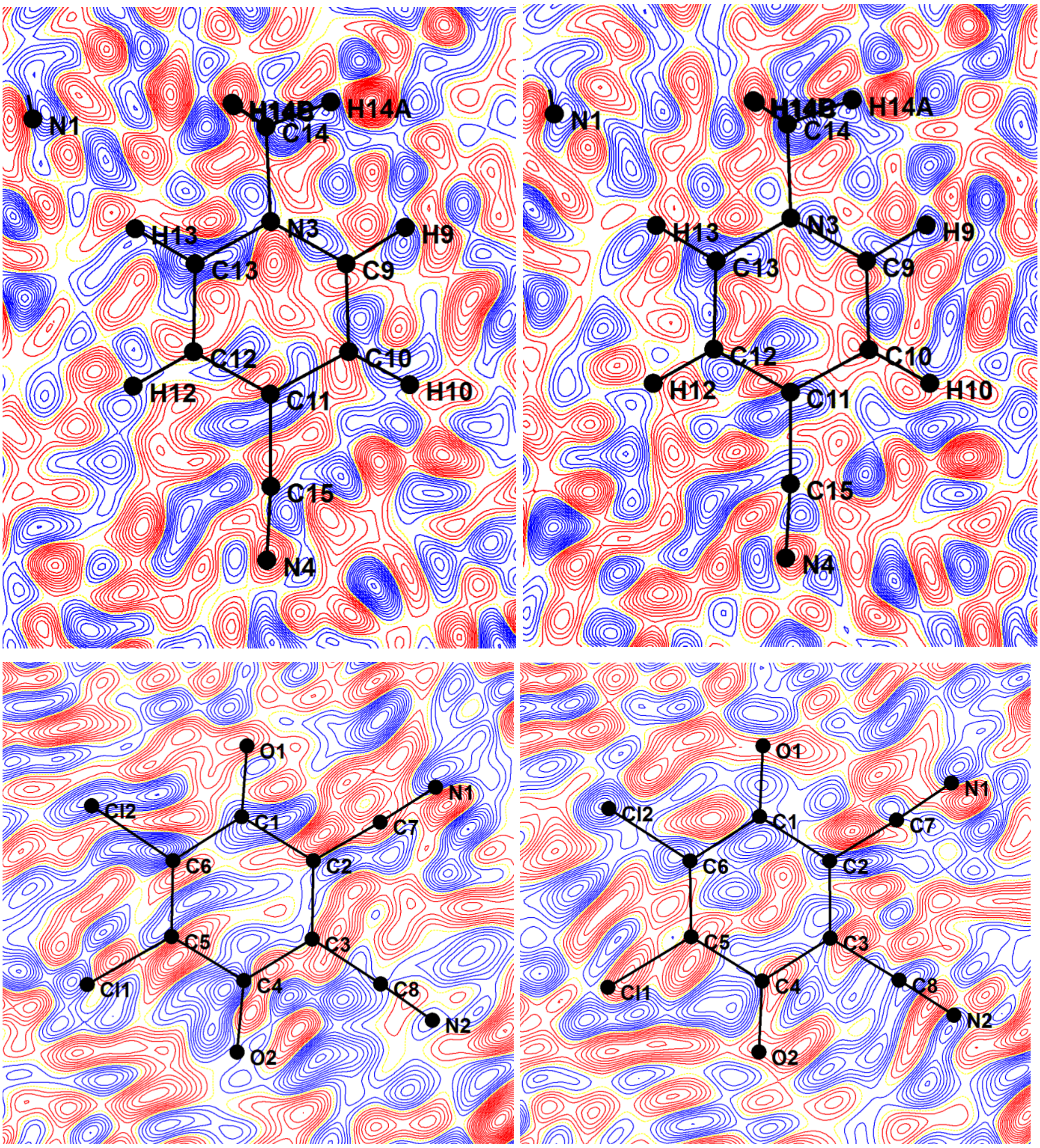

Figure S23 Residual density in the mean plane of 4-cyano-*N*-methylpyridinium cation (top row) and DDQ radical anion (bottom row) at 1.85 GPa. Left: spherical refinement, right: transferred-multipole refinement. Positive density is shown in blue and negative in red; yellow dotted lines represent zero density. Contours are drawn for 0.05 eÅ^{-1}.

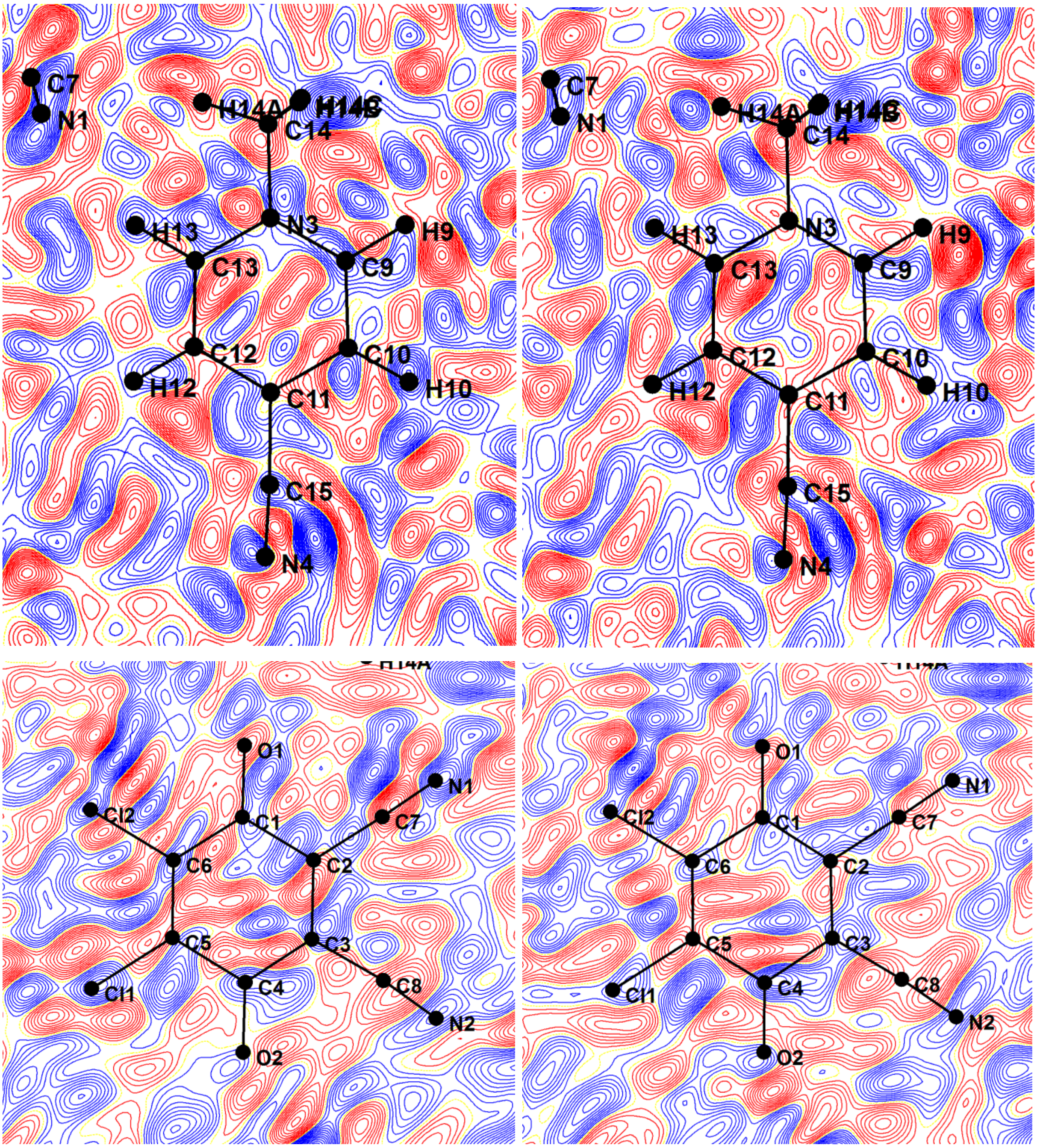

Figure S24 Residual density in the mean plane of 4-cyano-*N*-methylpyridinium cation (top row) and DDQ radical anion (bottom row) at 3.09 GPa. Left: spherical refinement, right: transferred-multipole refinement. Positive density is shown in blue and negative in red; yellow dotted lines represent zero density. Contours are drawn for 0.05 eÅ^{-1}.

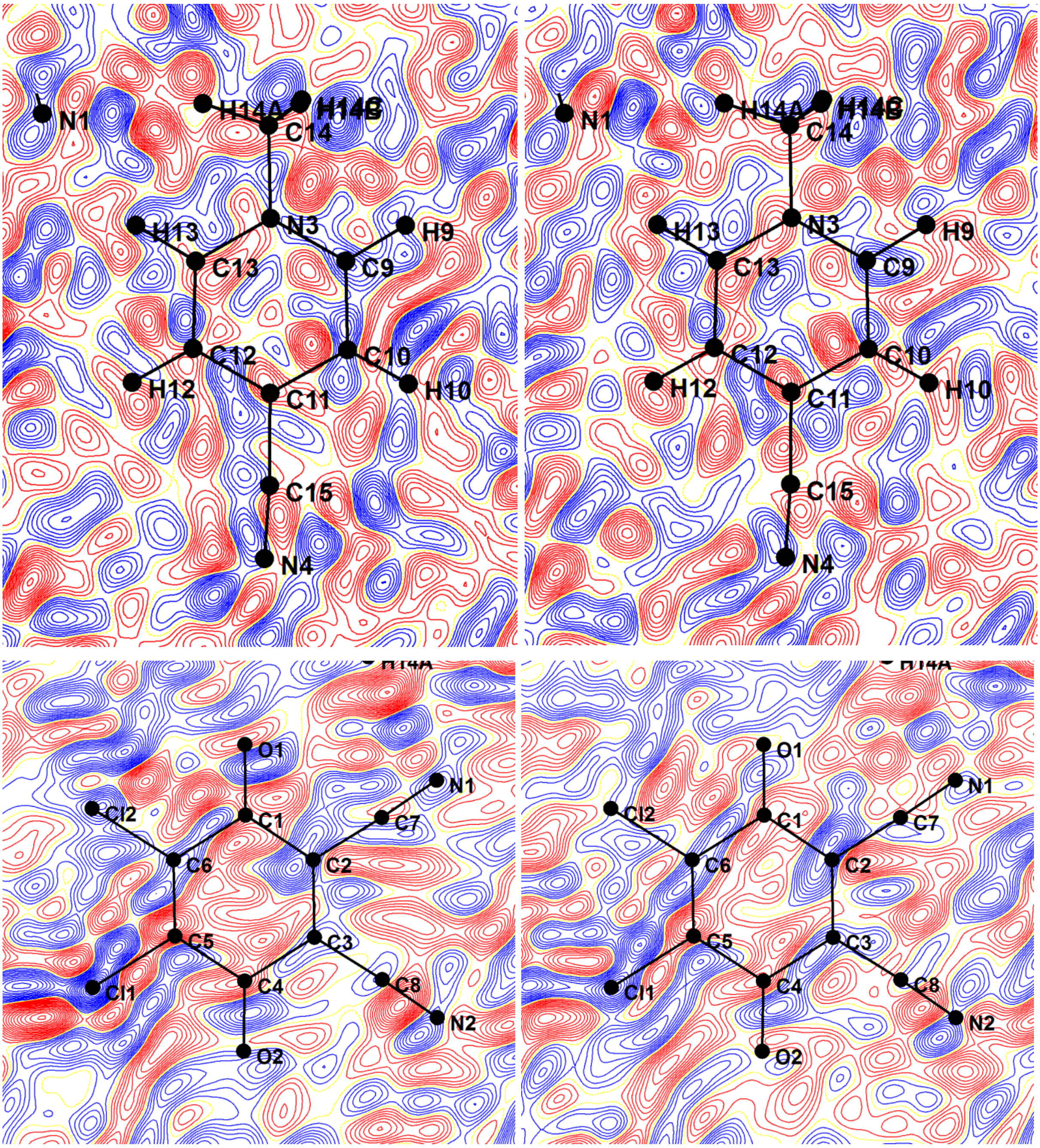

Figure S25 Residual density in the mean plane of 4-cyano-*N*-methylpyridinium cation (top row) and DDQ radical anion (bottom row) at 3.95 GPa. Left: spherical refinement, right: transferred-multipole refinement. Positive density is shown in blue and negative in red; yellow dotted lines represent zero density. Contours are drawn for 0.05 eÅ^{-1}.

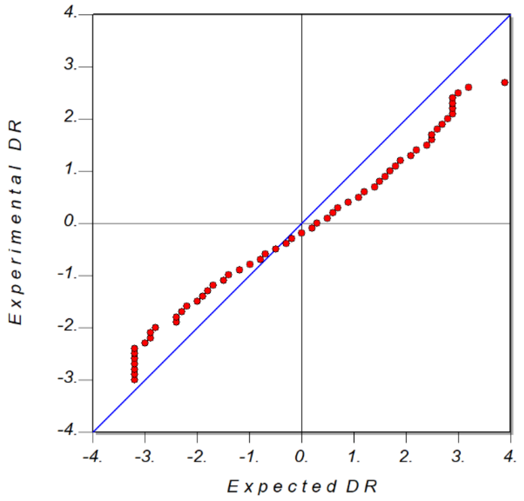

0.25 GPa

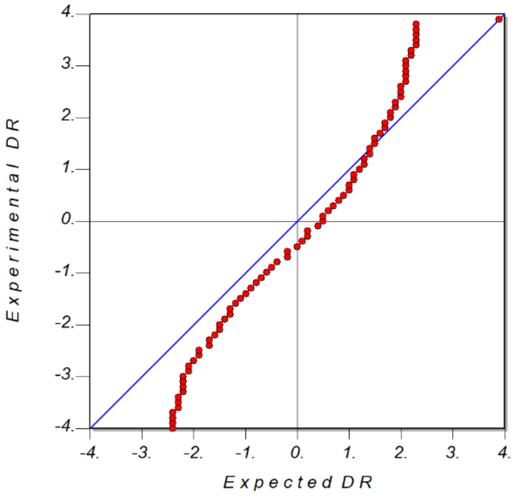

0.49 GPa

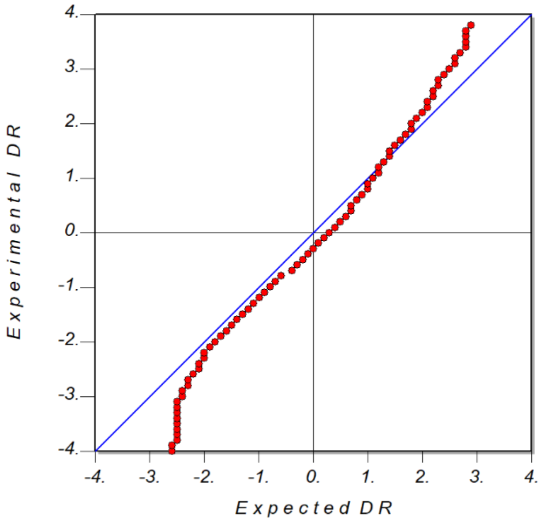

0.86 GPa

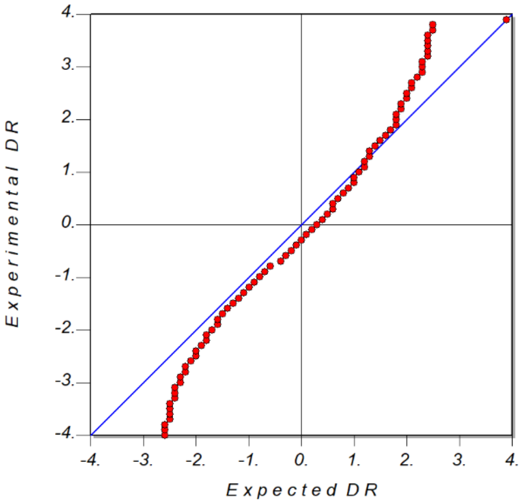

1.42 GPa

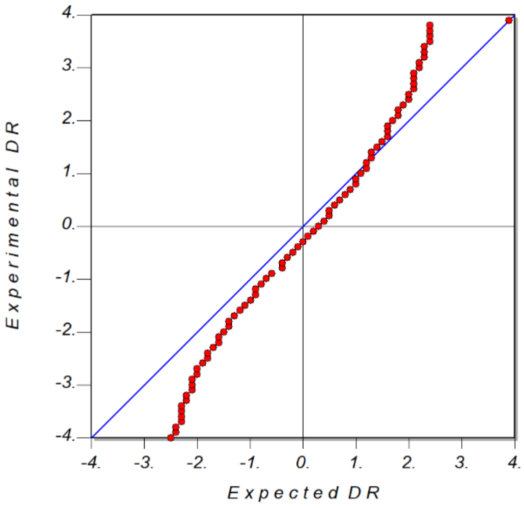

1.85 GPa

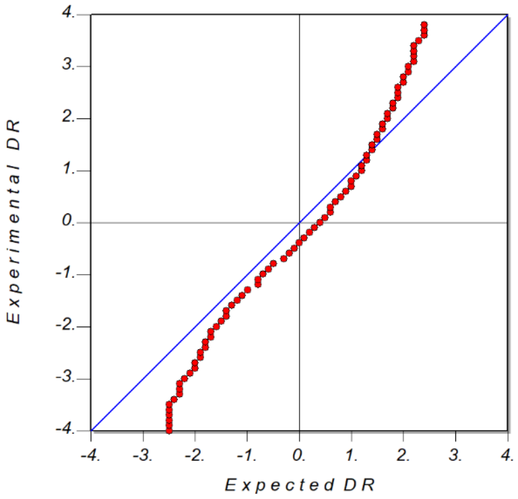

3.09 GPa

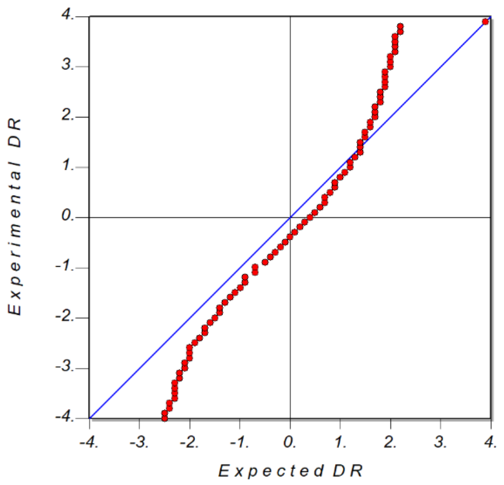

3.95 GPa

Figure S26 XDRK plot showing the expected and experimental Y_{obs}- Y_{calc} data profile for high-pressure data.

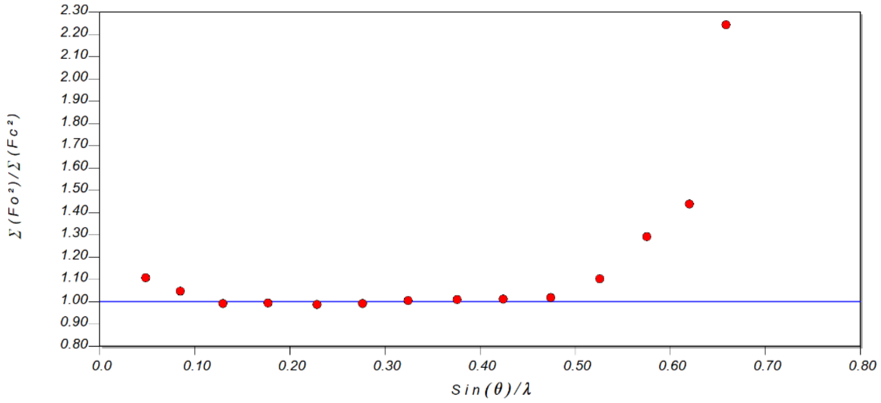

0.25 GPa

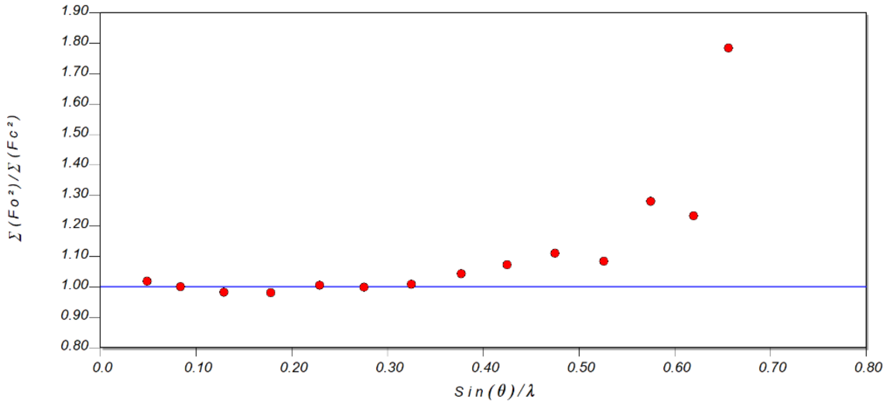

0.49 GPa

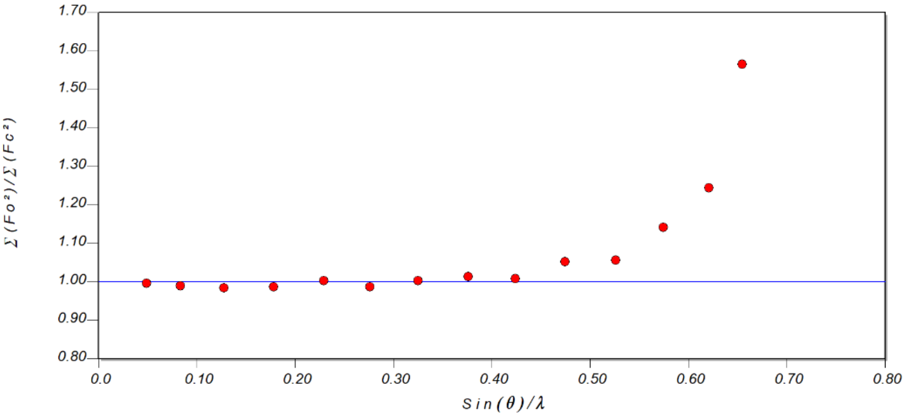

0.86 GPa

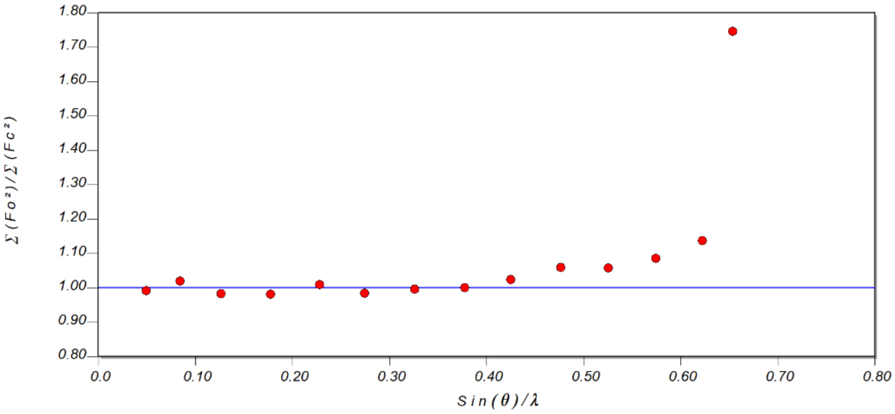

1.42 GPa

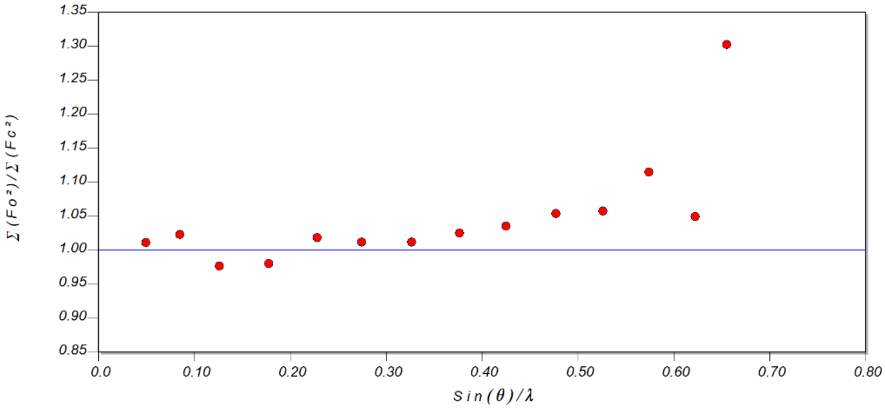

1.85 GPa

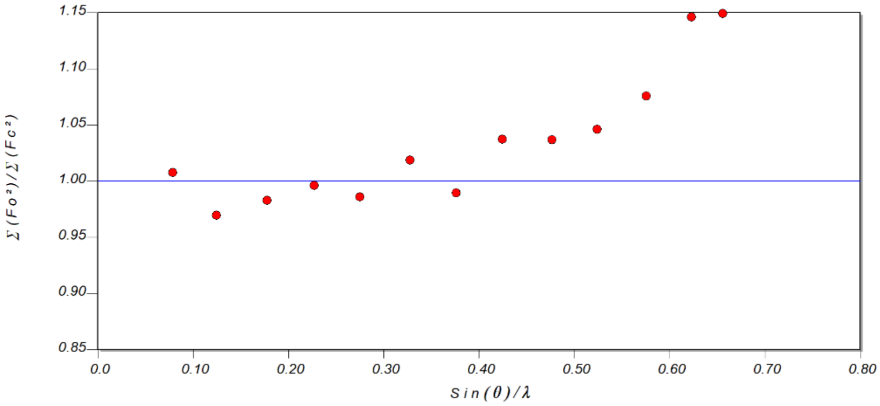

3.09 GPa

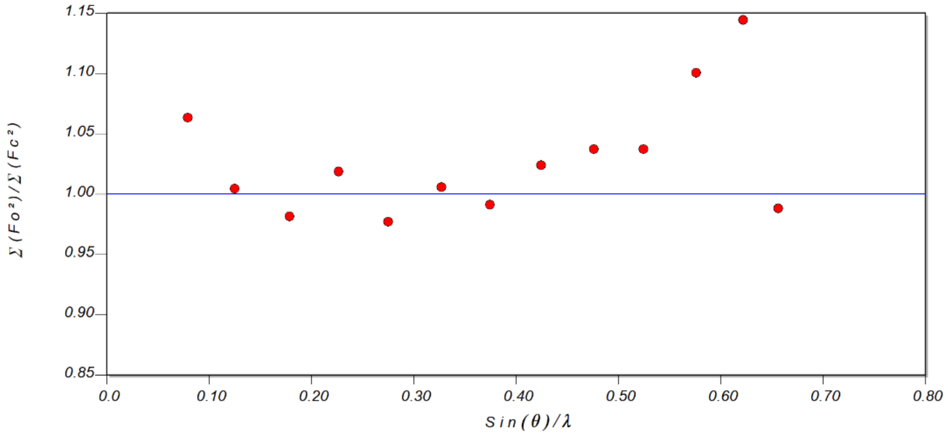

3.95 GPa

Figure S27 XDRK plot showing the fit of <Y_{obs}> *vs* <Y_{calc}> as a function of resolution for high-pressure data.

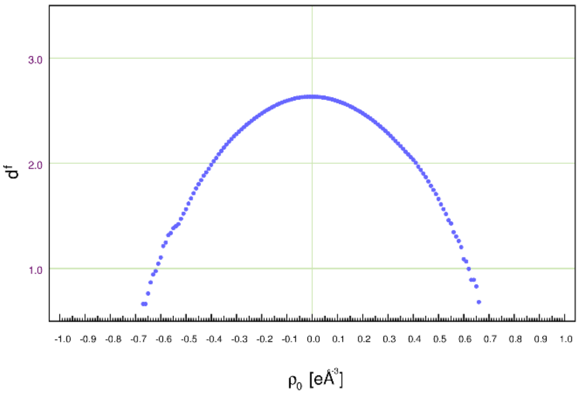

0.25 GPa

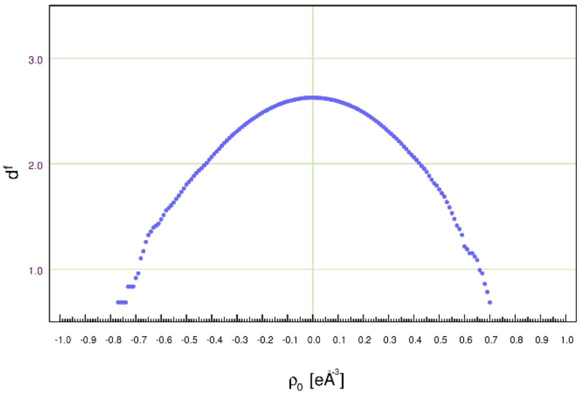

0.49 GPa

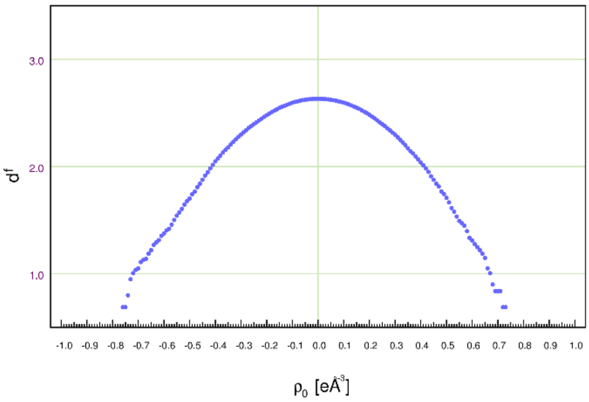

0.86 GPa

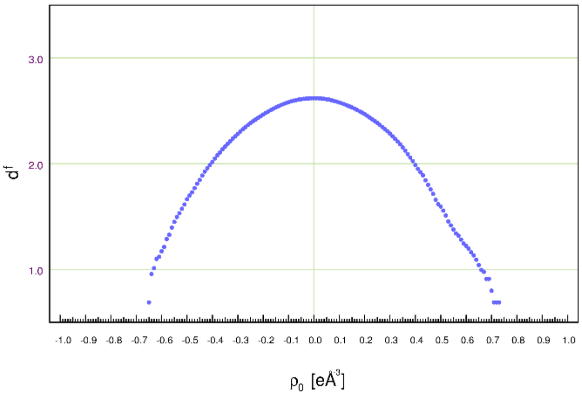

1.42 GPa

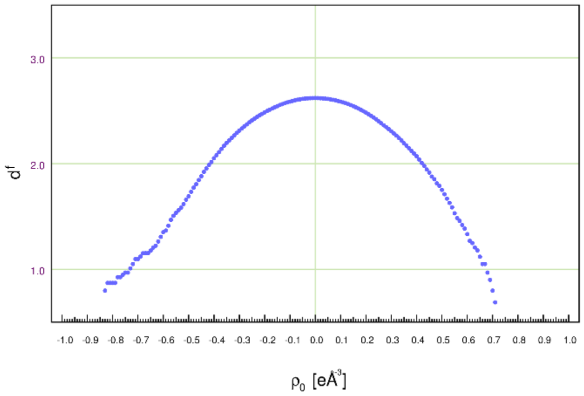

1.85 GPa

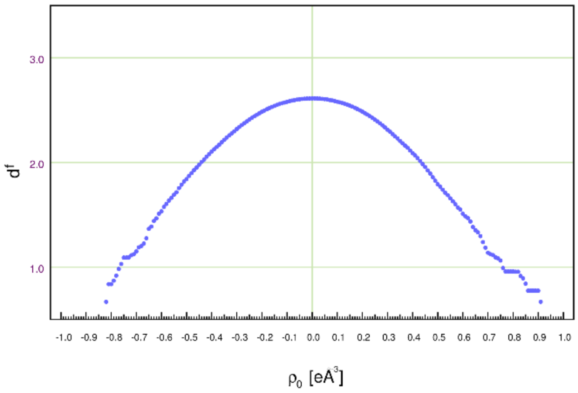

3.09 GPa

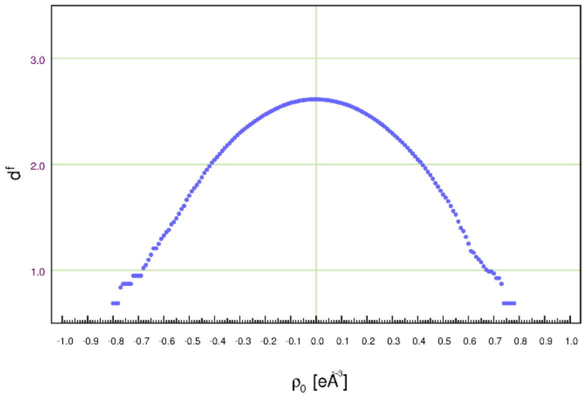

3.95 GPa

Figure S28 Fractal dimension plots of residual density $[(Y_{obs}- Y_{calc})/\sigma_{Yobs}]$ for high-pressure data. The plot was prepared by the program *jnk2rda* [4].

S6 Maps of deformation density and Laplacian at variable temperatures

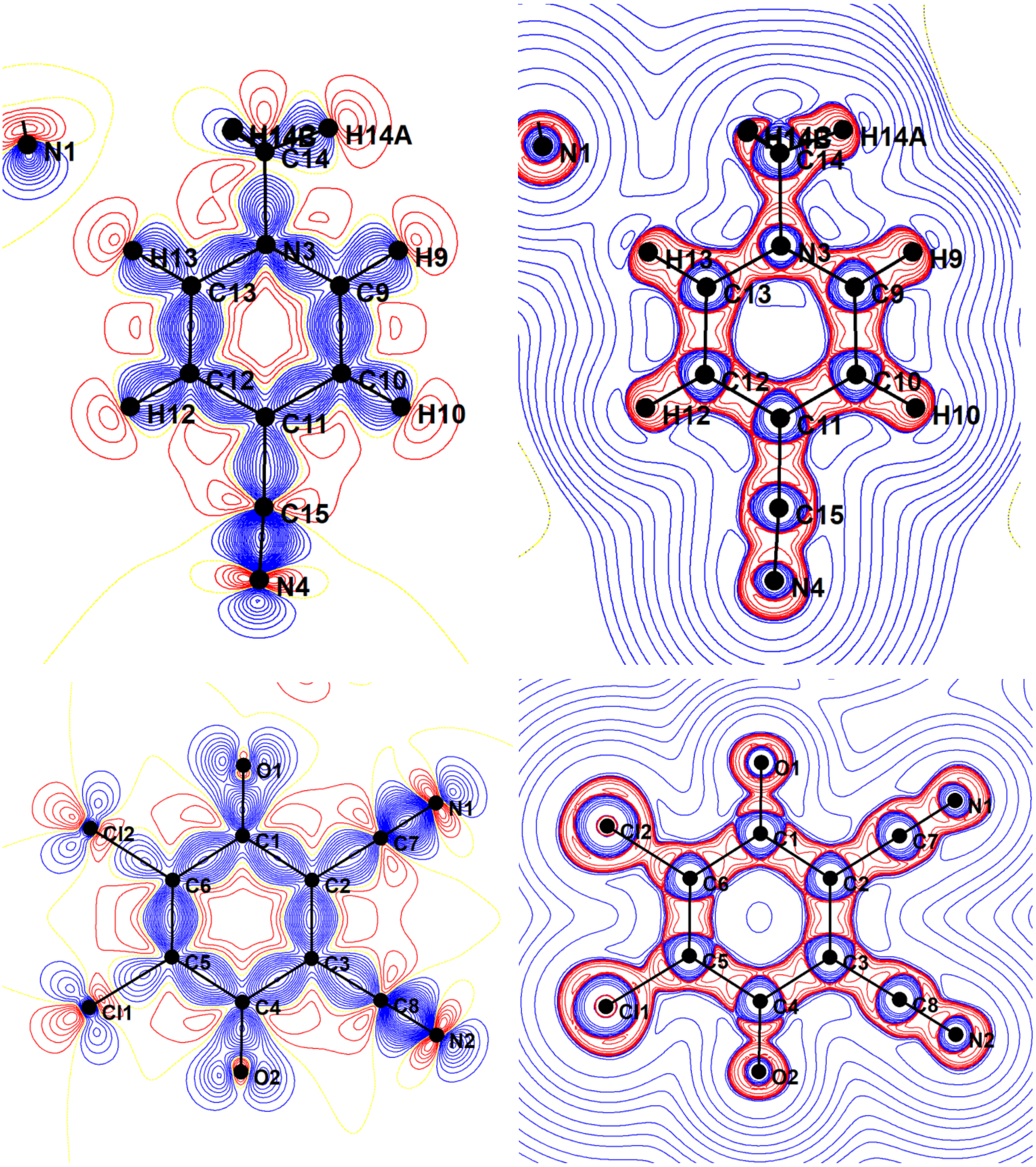

Figure S29 Maps of deformation density (left; contours at 0.05 e Å^{-3}) and Laplacian (right; contours drawn for 2, 4, $8\cdot10^{n}$ e Å^{-5}, $n=-3\ldots2$) in the mean plane of the 4-cyano-*N*-methylpyridinium cation (top row) and DDQ radical anion (bottom row) at 90 K.

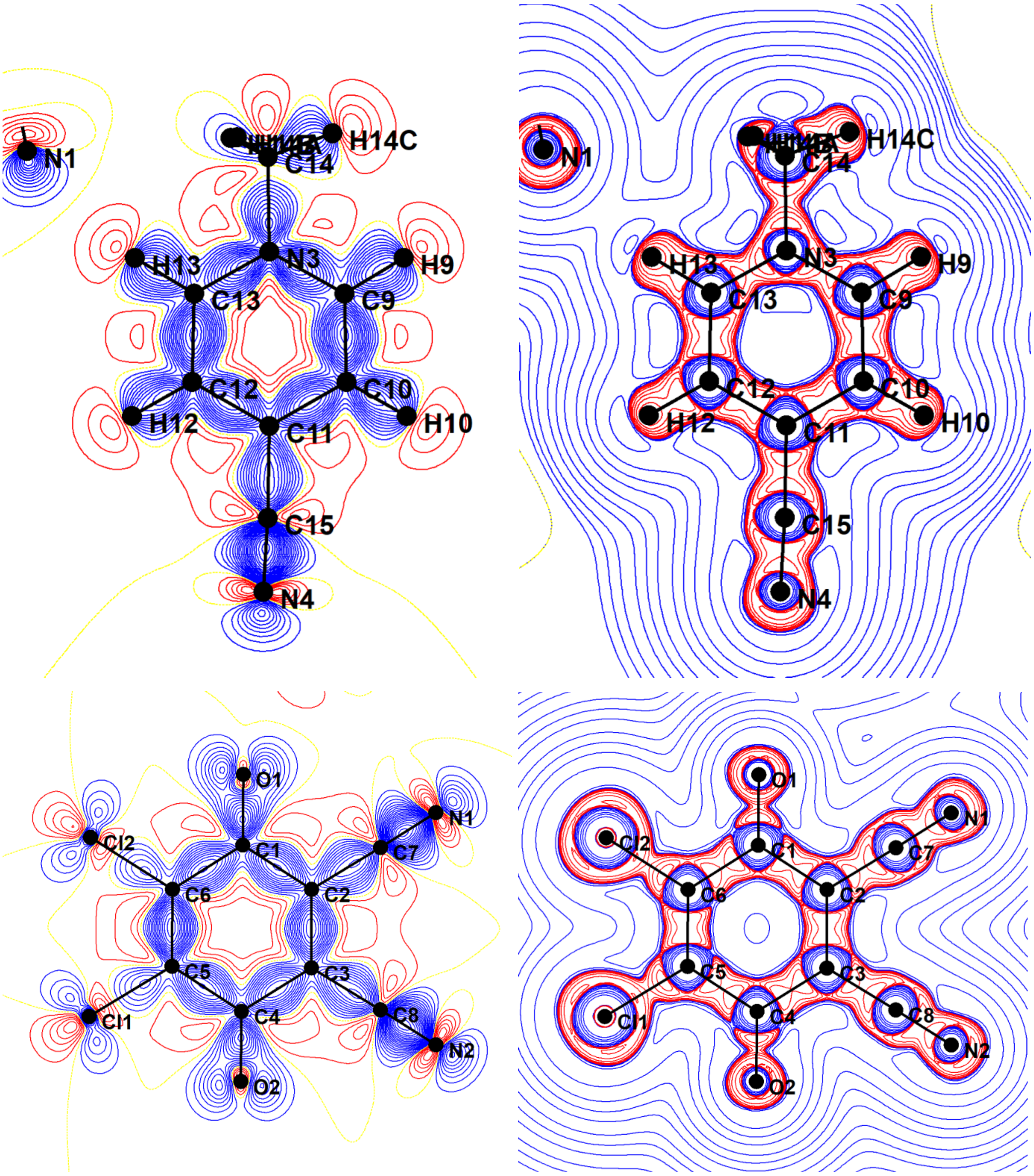

Figure S30 Maps of deformation density (left; contours at 0.05 e Å^{-3}) and Laplacian (right; contours drawn for 2, 4, 8·10^{n} e Å^{-5}, n=−3…2) in the mean plane of the 4-cyano-*N*-methylpyridinium cation (top row) and DDQ radical anion (bottom row) at 120 K.

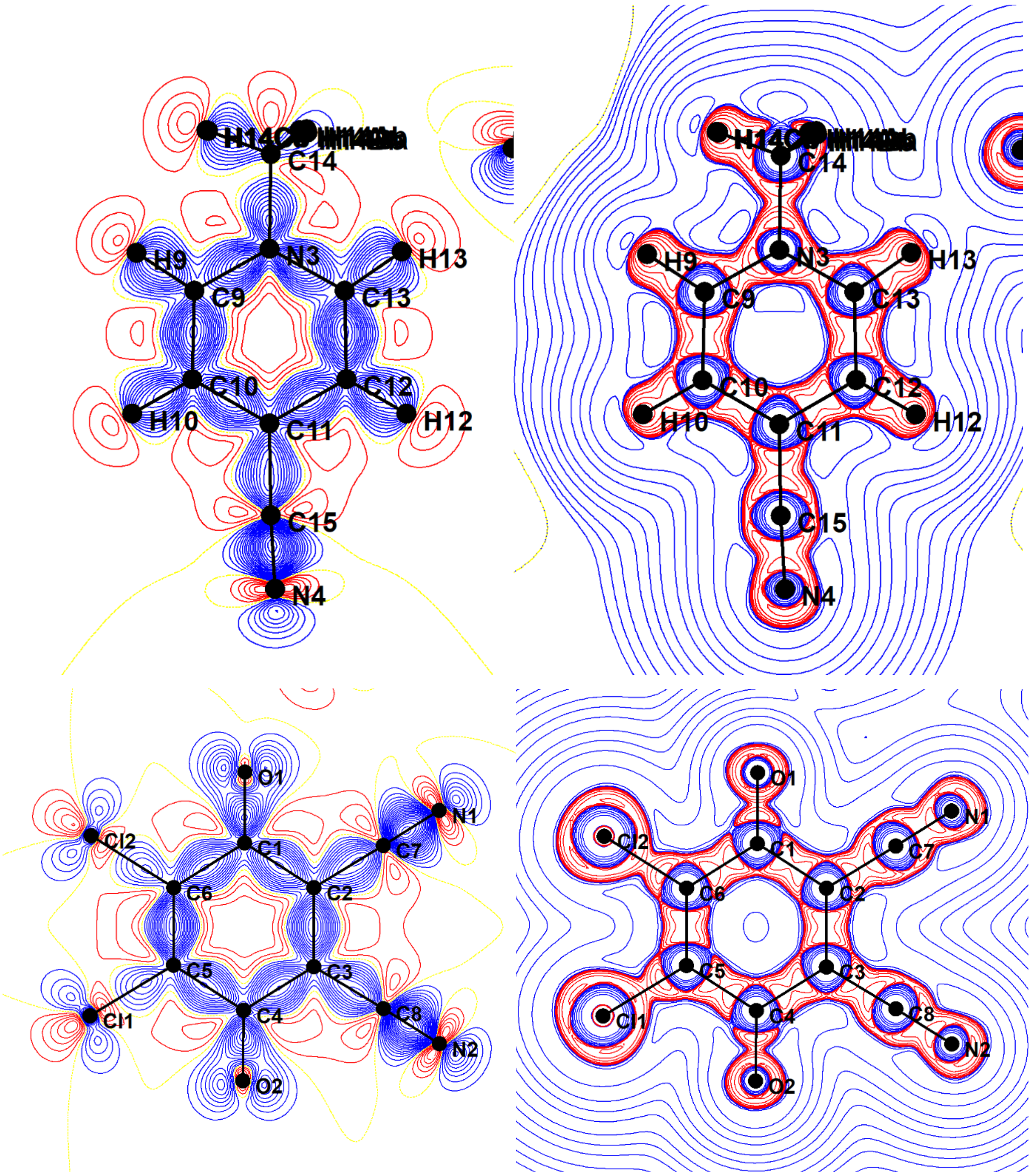

Figure S31 Maps of deformation density (left; contours at 0.05 e Å^{-3}) and Laplacian (right; contours drawn for 2, 4, 8·10^{n} e Å^{-5}, n=—3…2) in the mean plane of the 4-cyano-*N*-methylpyridinium cation (top row) and DDQ radical anion (bottom row) at 150 K.

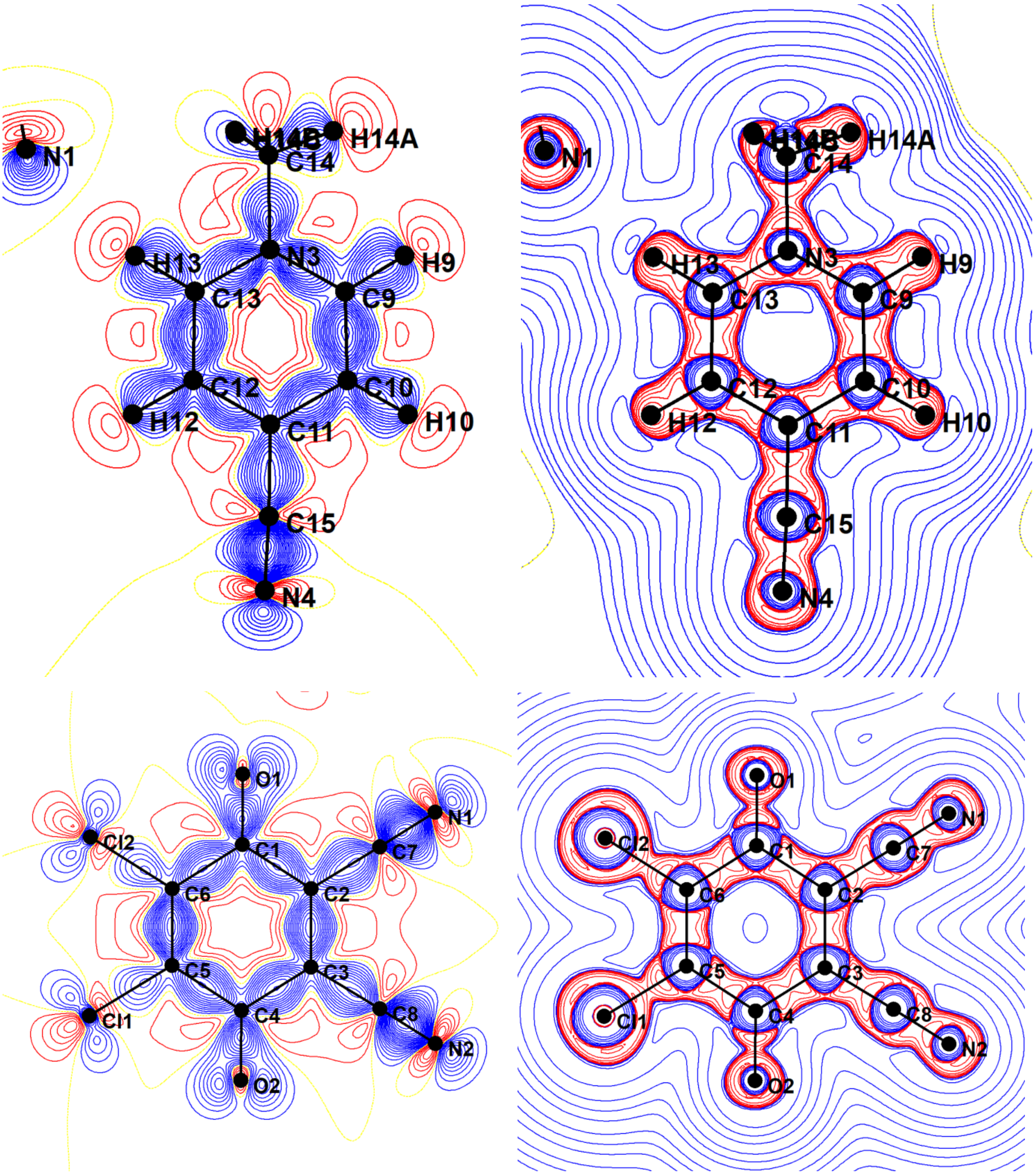

Figure S32 Maps of deformation density (left; contours at 0.05 e Å^{-3}) and Laplacian (right; contours drawn for 2, 4, 8·10^{n} e Å^{-5}, n=−3…2) in the mean plane of the 4-cyano-*N*-methylpyridinium cation (top row) and DDQ radical anion (bottom row) at 180 K.

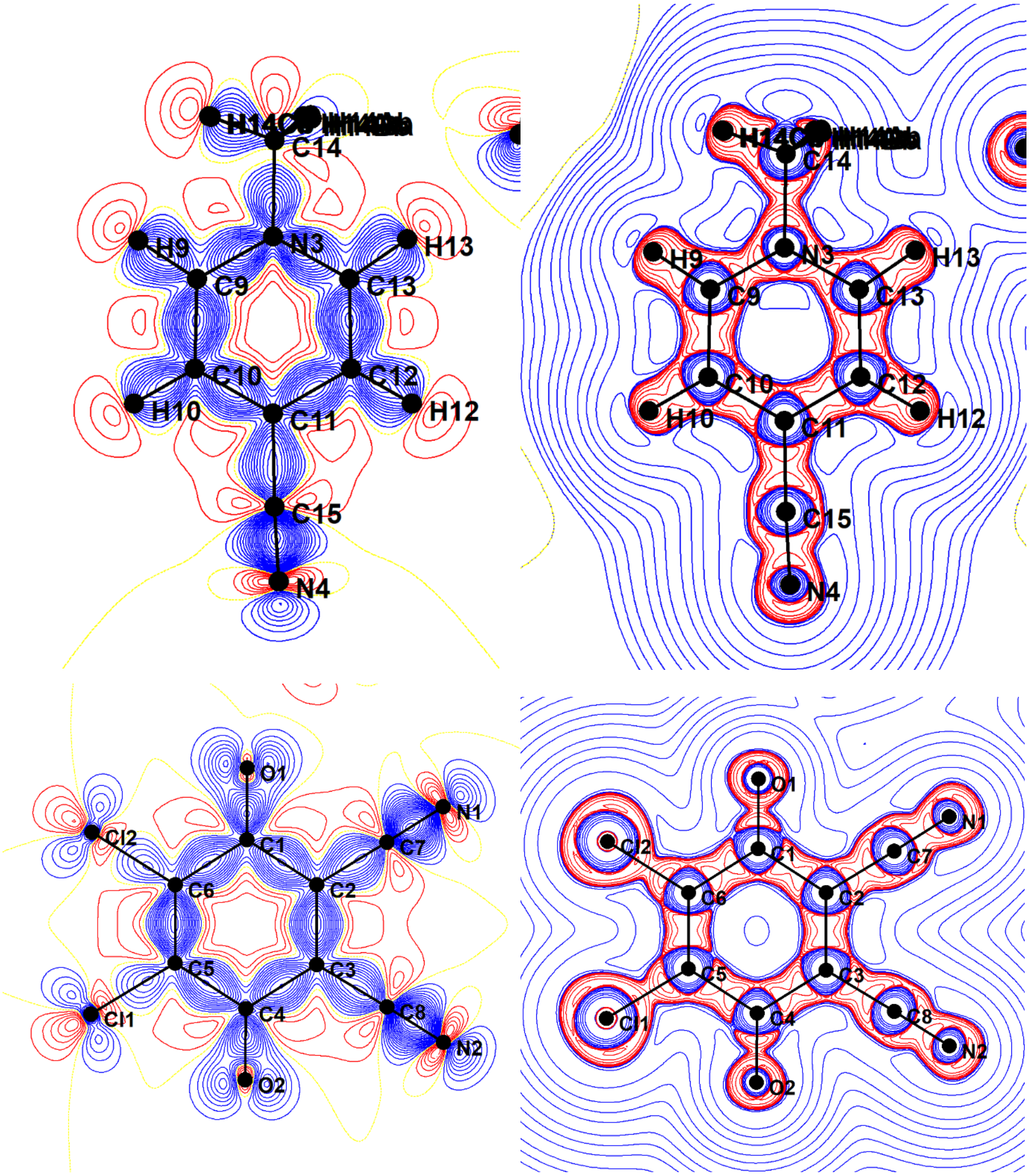

Figure S33 Maps of deformation density (left; contours at 0.05 e Å^{-3}) and Laplacian (right; contours drawn for 2, 4, 8·10^{n} e Å^{-5}, n=−3…2) in the mean plane of the 4-cyano-*N*-methylpyridinium cation (top row) and DDQ radical anion (bottom row) at 210 K.

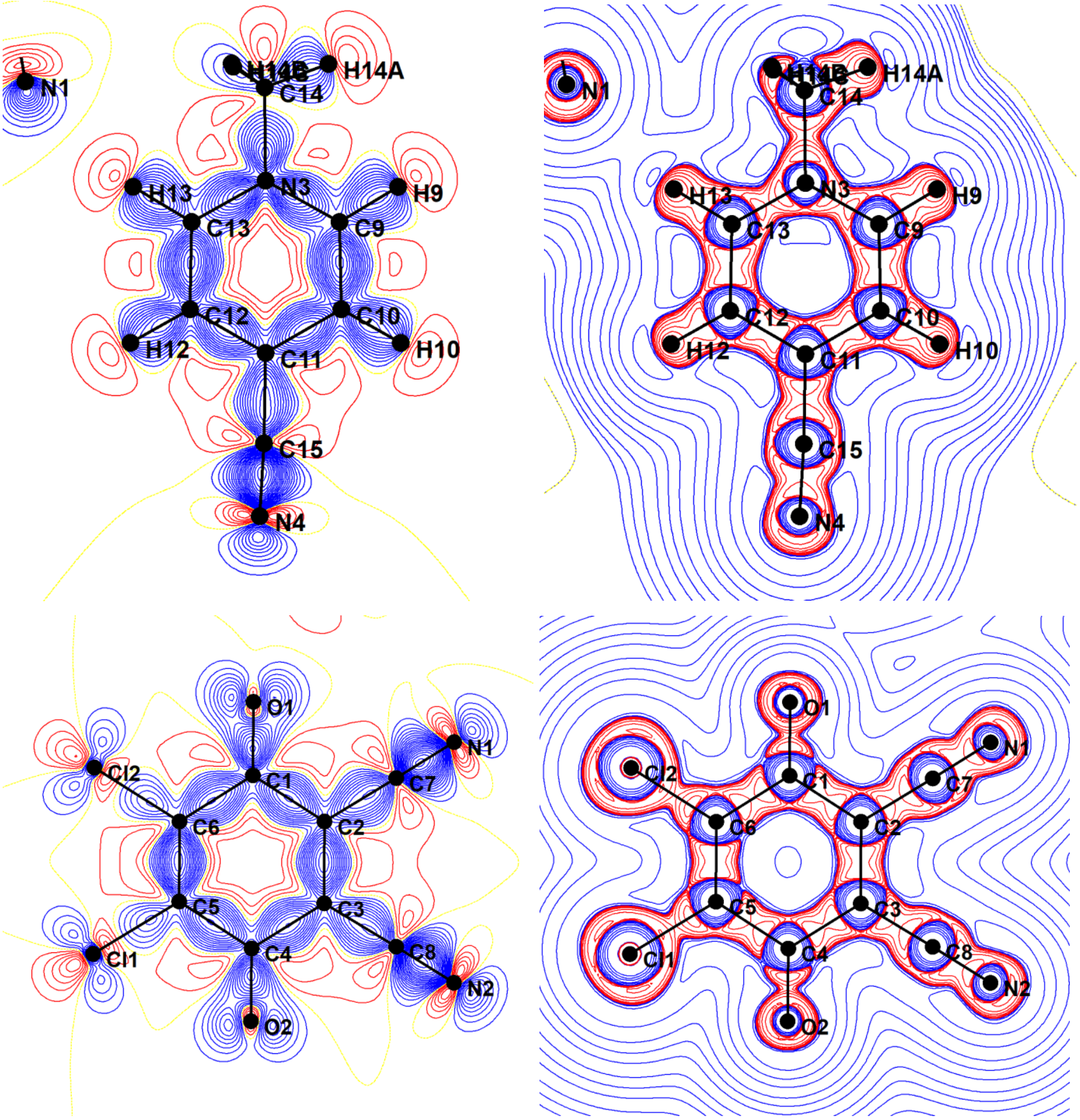

Figure S34 Maps of deformation density (left; contours at 0.05 e Å^{-3}) and Laplacian (right; contours drawn for 2, 4, 8·10^{n} e Å^{-5}, n=–3…2) in the mean plane of the 4-cyano-*N*-methylpyridinium cation (top row) and DDQ radical anion (bottom row) at 240 K.

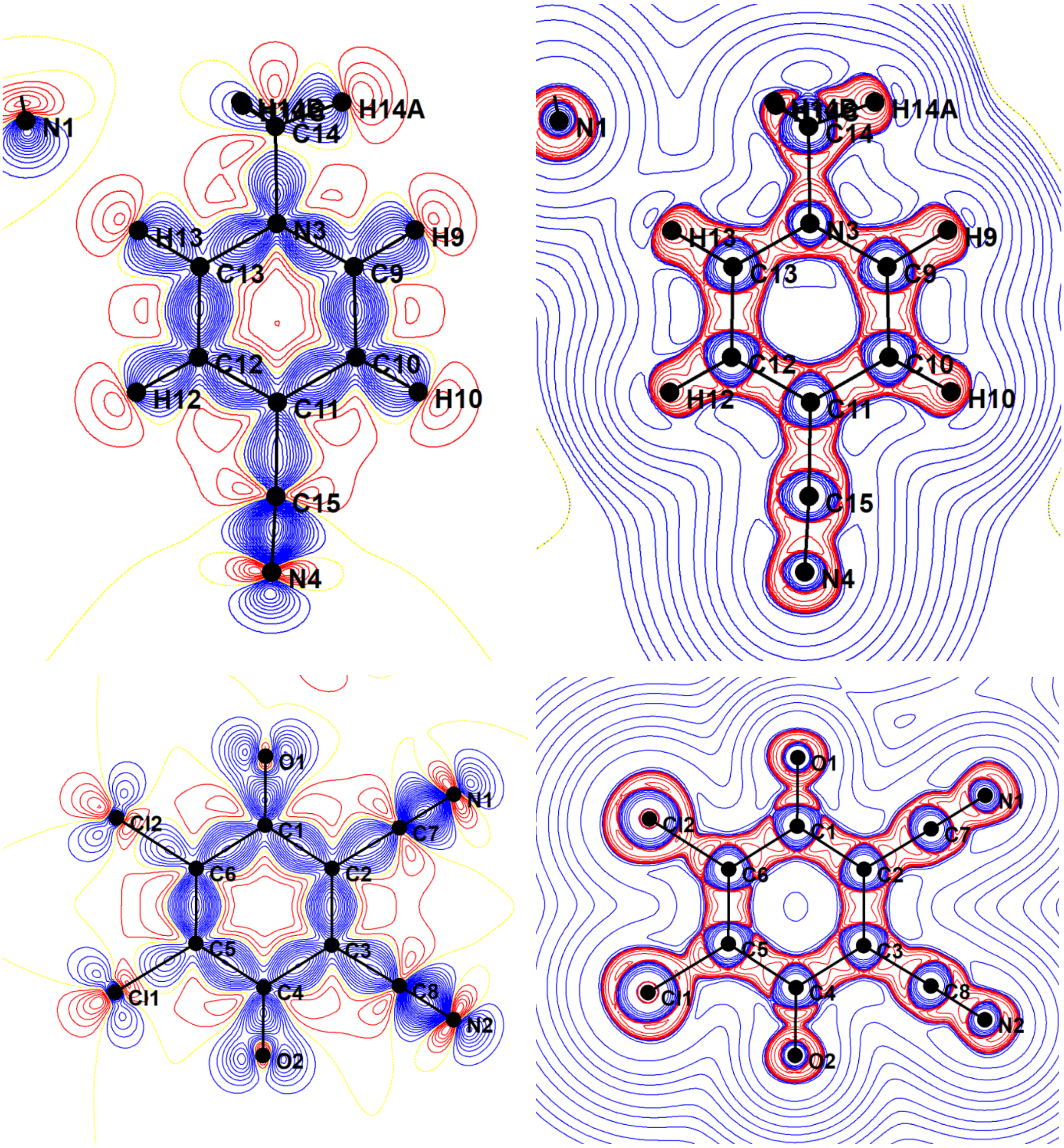

Figure S35 Maps of deformation density (left; contours at 0.05 e Å^{-3}) and Laplacian (right; contours drawn for 2, 4, 8·10^{n} e Å^{-5}, n=−3…2) in the mean plane of the 4-cyano-*N*-methylpyridinium cation (top row) and DDQ radical anion (bottom row) at 270 K.

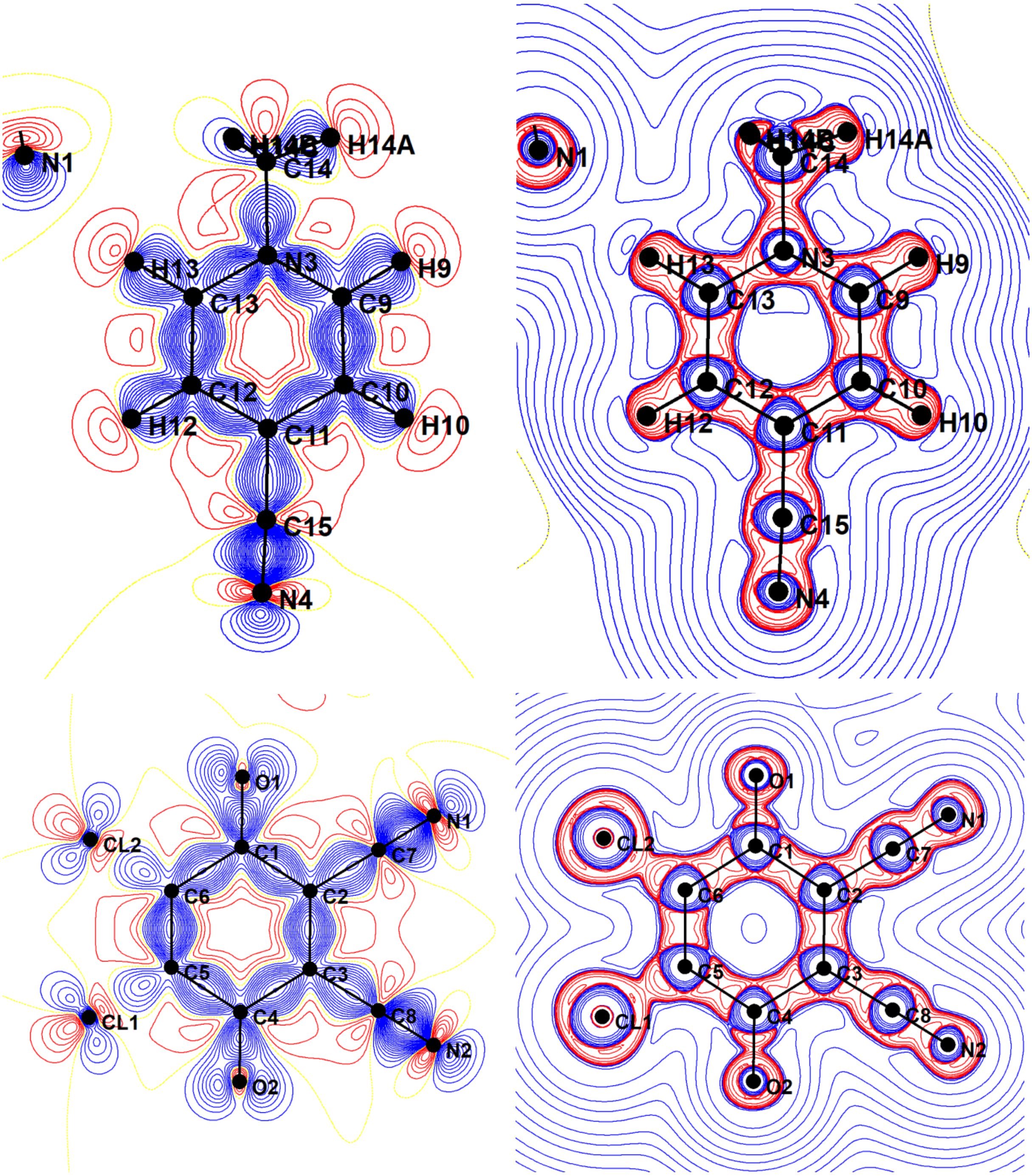

Figure S36 Maps of deformation density (left; contours at 0.05 e Å^{-3}) and Laplacian (right; contours drawn for 2, 4, 8·10^{n} e Å^{-5}, n=—3…2) in the mean plane of the 4-cyano-*N*-methylpyridinium cation (top row) and DDQ radical anion (bottom row) at 293 K.

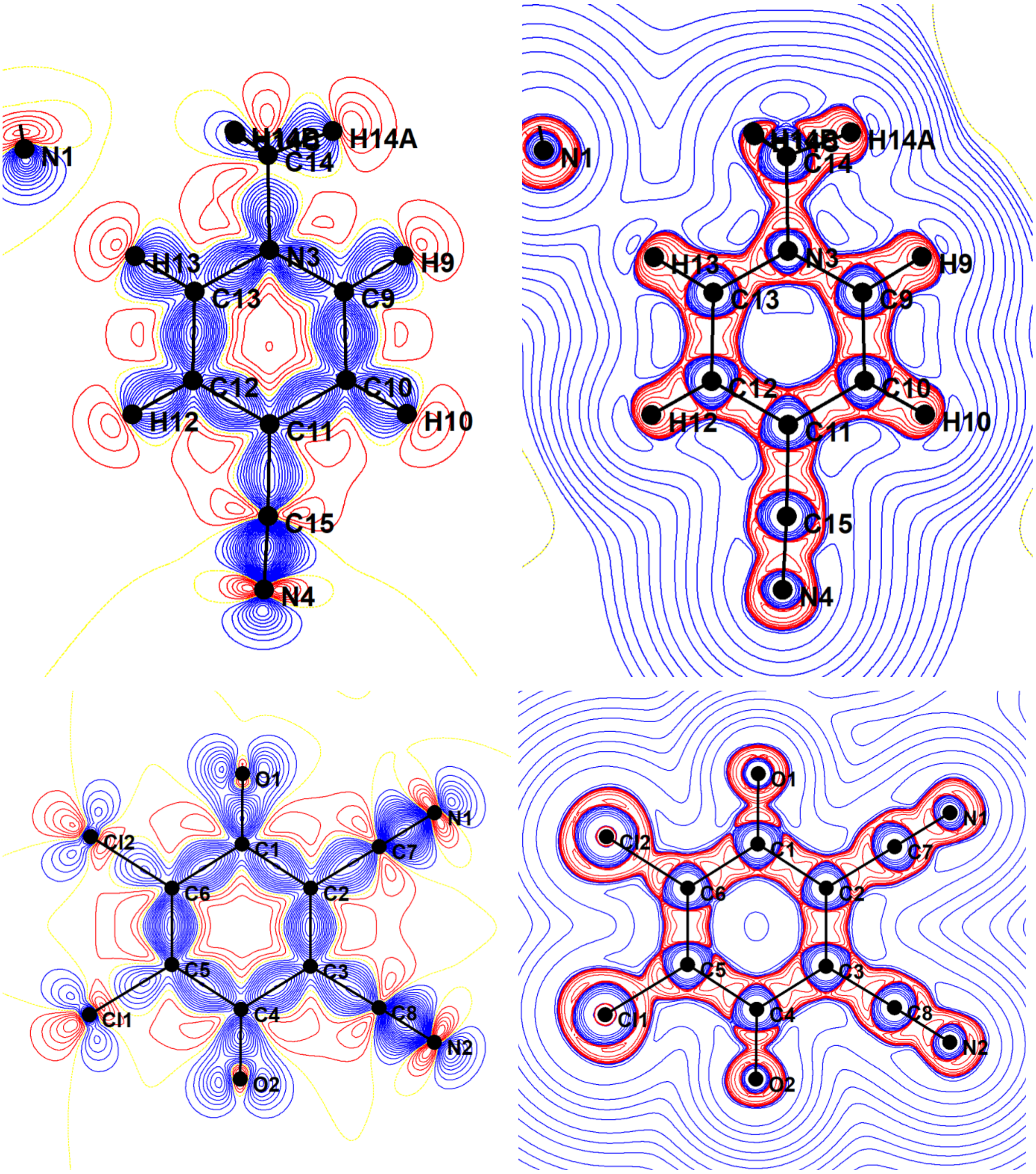

Figure S37 Maps of deformation density (left; contours at 0.05 e Å^{-3}) and Laplacian (right; contours drawn for 2, 4, 8·10^{n} e Å^{-5}, n=–3…2) in the mean plane of the 4-cyano-*N*-methylpyridinium cation (top row) and DDQ radical anion (bottom row) at 310 K.

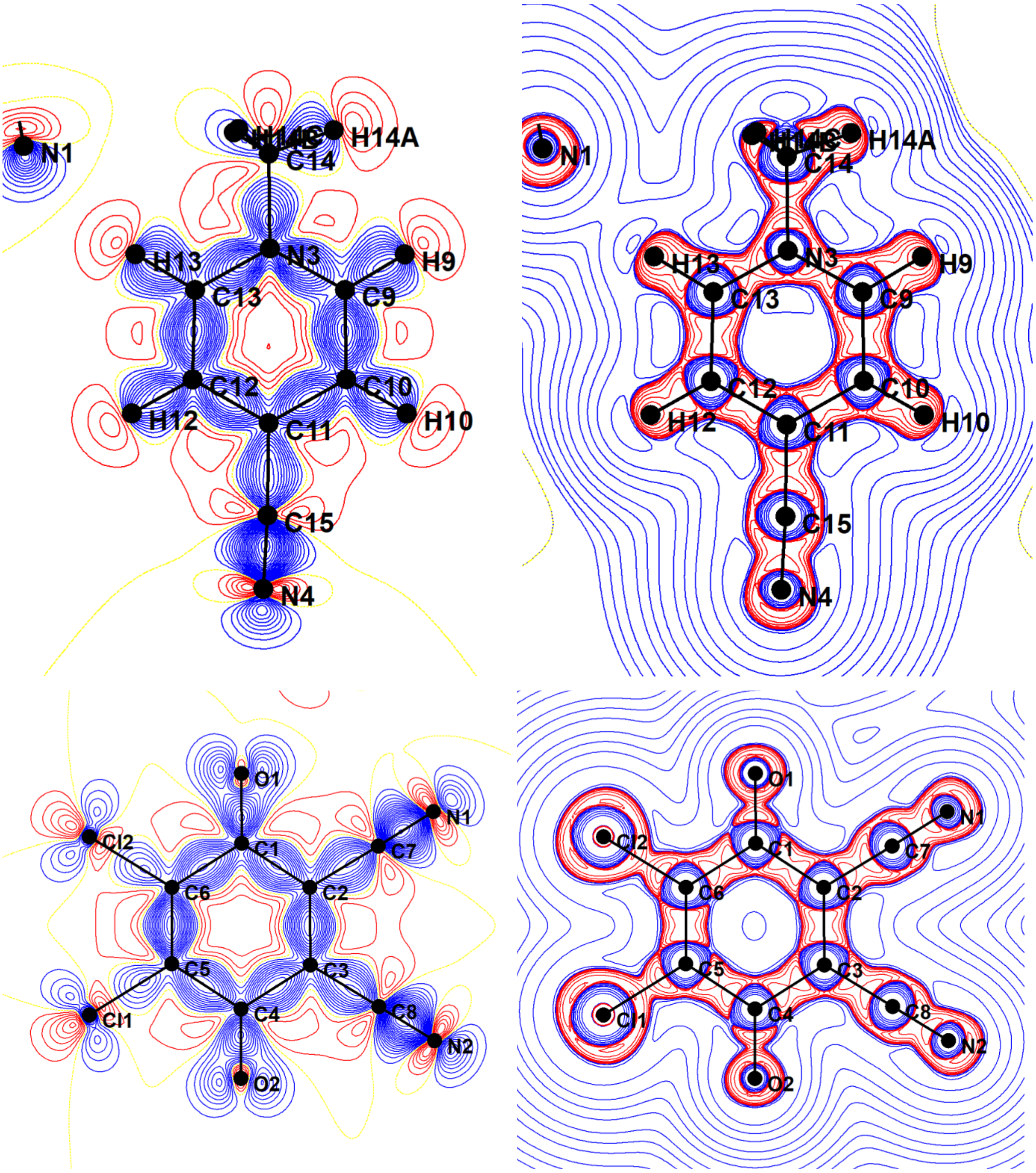

Figure S38 Maps of deformation density (left; contours at 0.05 e Å^{-3}) and Laplacian (right; contours drawn for 2, 4, 8·10^n e Å^{-5}, n=−3…2) in the mean plane of the 4-cyano-*N*-methylpyridinium cation (top row) and DDQ radical anion (bottom row) at 340 K.

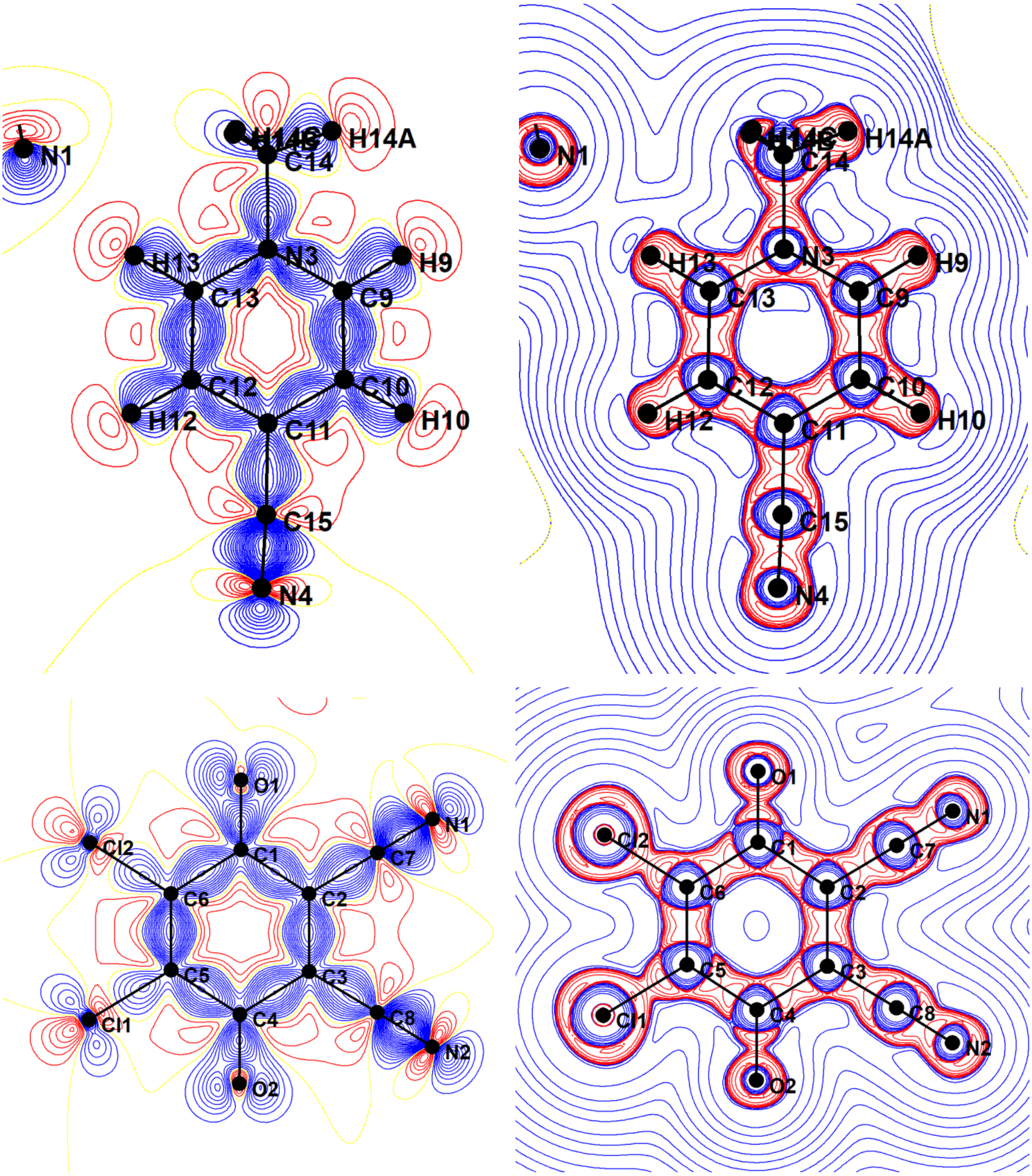

Figure S39 Maps of deformation density (left; contours at 0.05 e Å^{-3}) and Laplacian (right; contours drawn for 2, 4, $8\cdot10^{n}$ e Å^{-5}, n=—3…2) in the mean plane of the 4-cyano-*N*-methylpyridinium cation (top row) and DDQ radical anion (bottom row) at 370 K.

S7 Maps of deformation density and Laplacian at high pressures

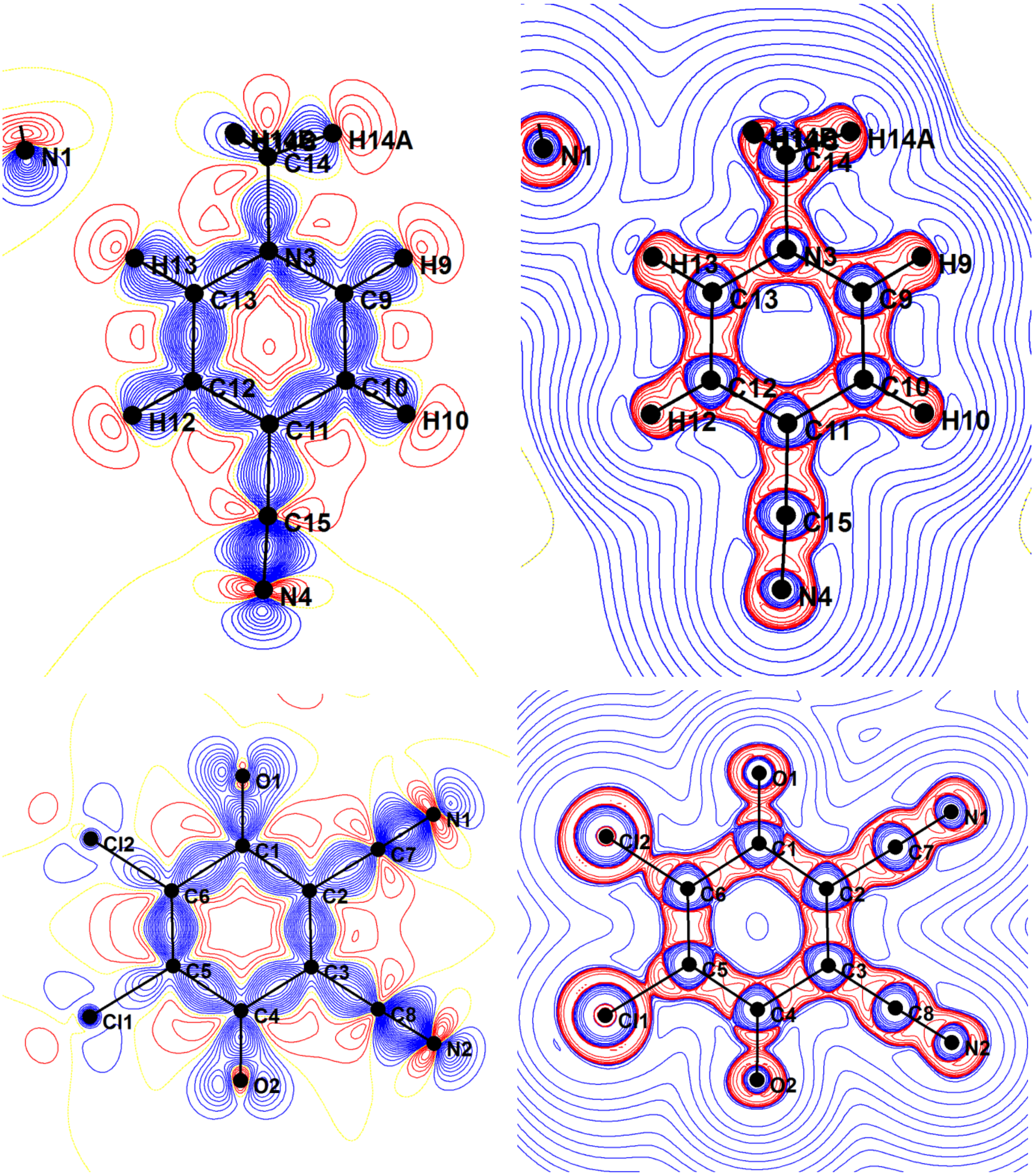

Figure S40 Maps of deformation density (left; contours at 0.05 e Å^{-3}) and Laplacian (right; contours drawn for 2, 4, 8·10^{n} e Å^{-5}, n=—3…2) in the mean plane of the 4-cyano-*N*-methylpyridinium cation (top row) and DDQ radical anion (bottom row) at 0.25 GPa.

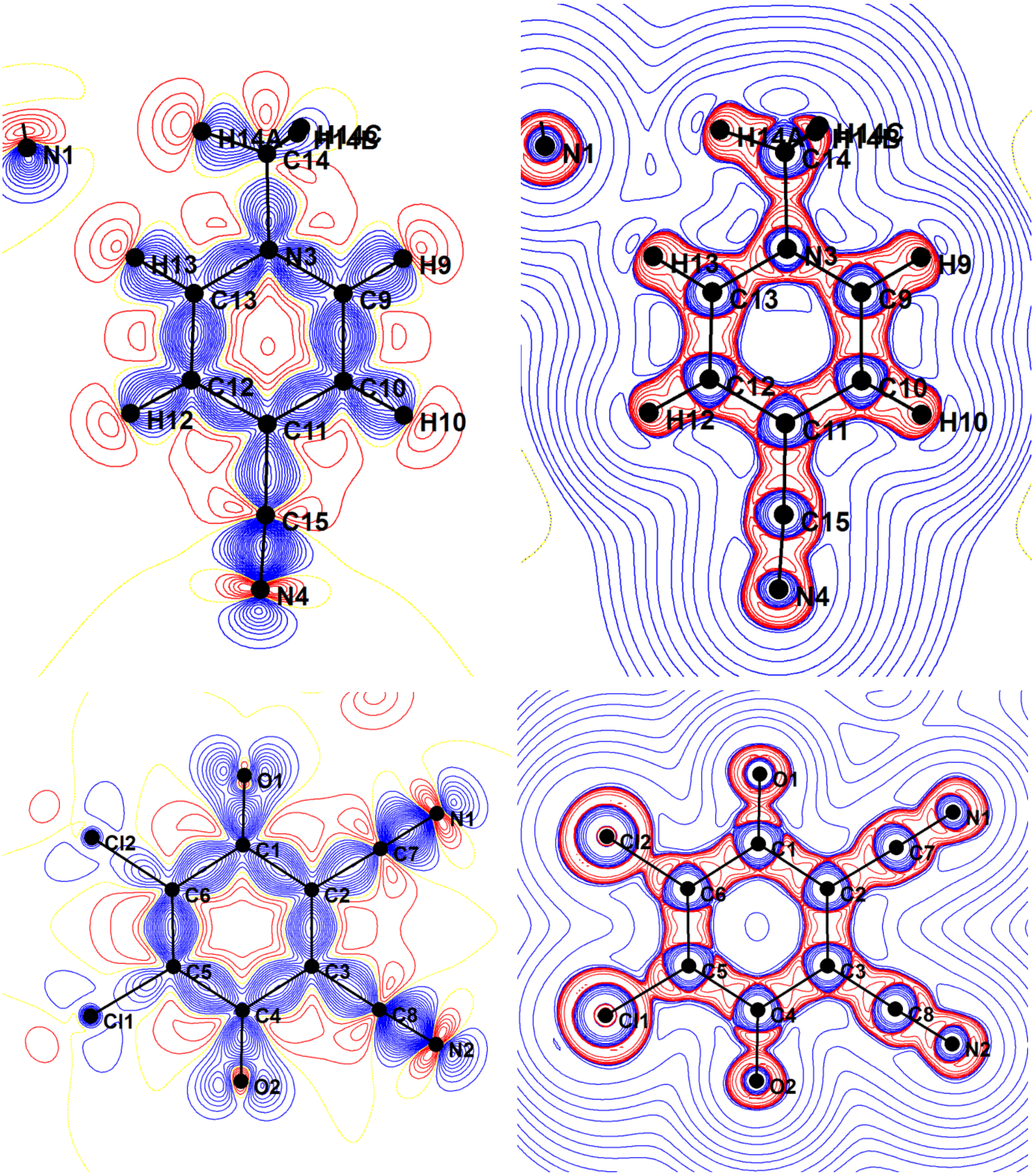

Figure S41 Maps of deformation density (left; contours at 0.05 e Å^{-3}) and Laplacian (right; contours drawn for 2, 4, 8·10^{n} e Å^{-5}, n=—3…2) in the mean plane of the 4-cyano-*N*-methylpyridinium cation (top row) and DDQ radical anion (bottom row) at 0.49 GPa.

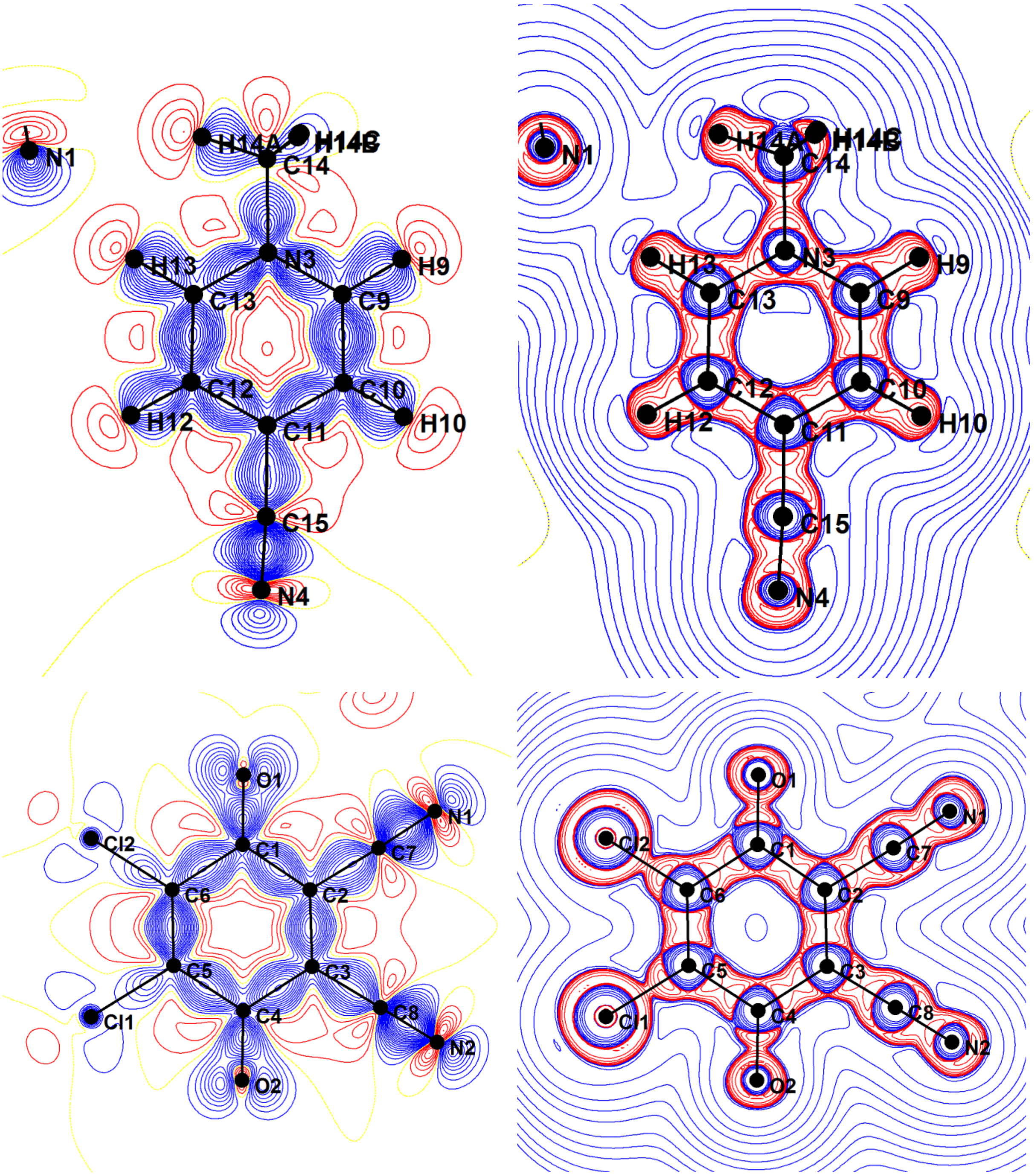

Figure S42 Maps of deformation density (left; contours at 0.05 e Å^{-3}) and Laplacian (right; contours drawn for 2, 4, 8·10^{n} e Å^{-5}, n=—3…2) in the mean plane of the 4-cyano-*N*-methylpyridinium cation (top row) and DDQ radical anion (bottom row) at 0.86 GPa.

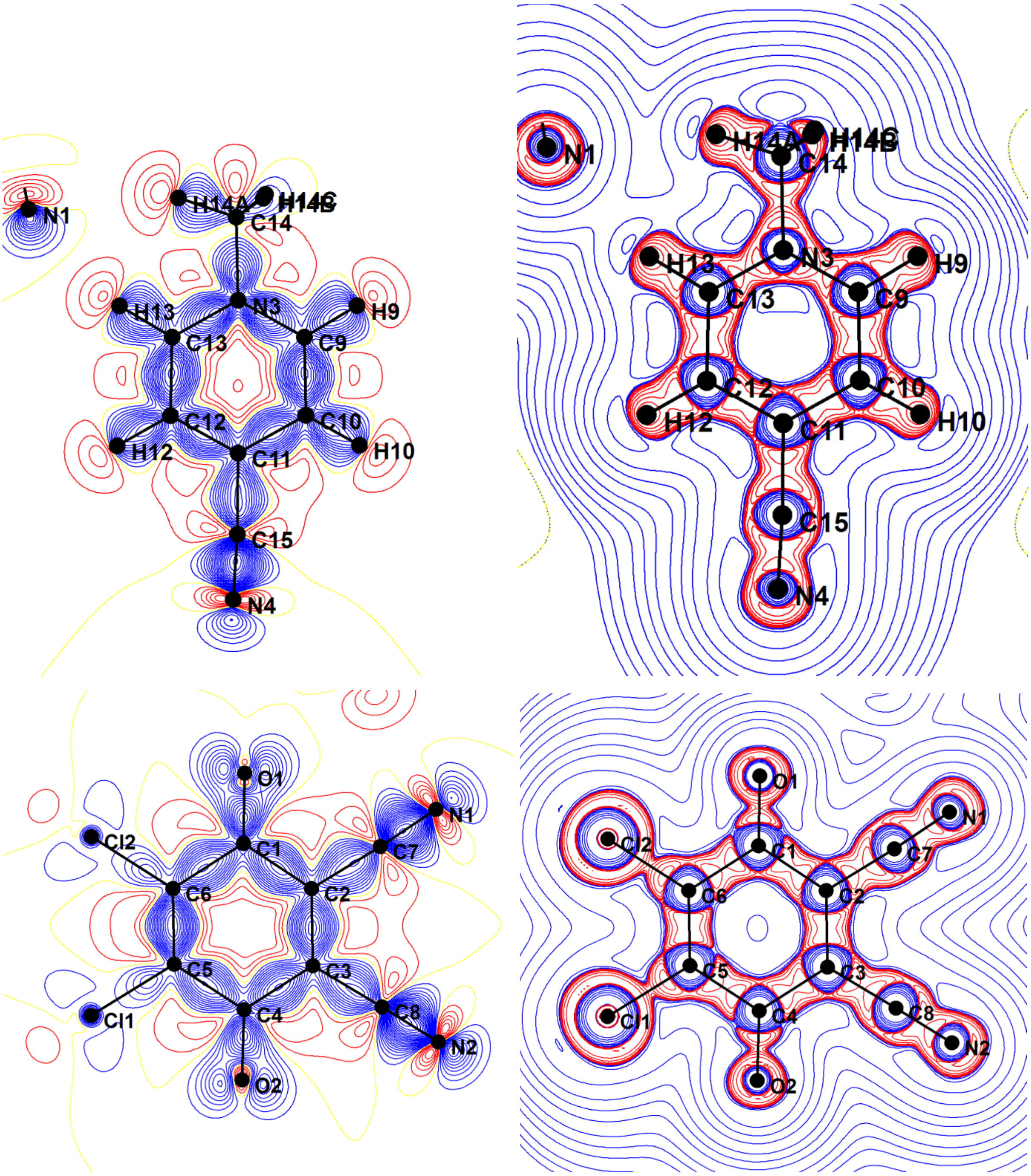

Figure S43 Maps of deformation density (left; contours at 0.05 e Å^{-3}) and Laplacian (right; contours drawn for 2, 4, 8·10^{n} e Å^{-5}, n=—3…2) in the mean plane of the 4-cyano-*N*-methylpyridinium cation (top row) and DDQ radical anion (bottom row) at 1.42 GPa.

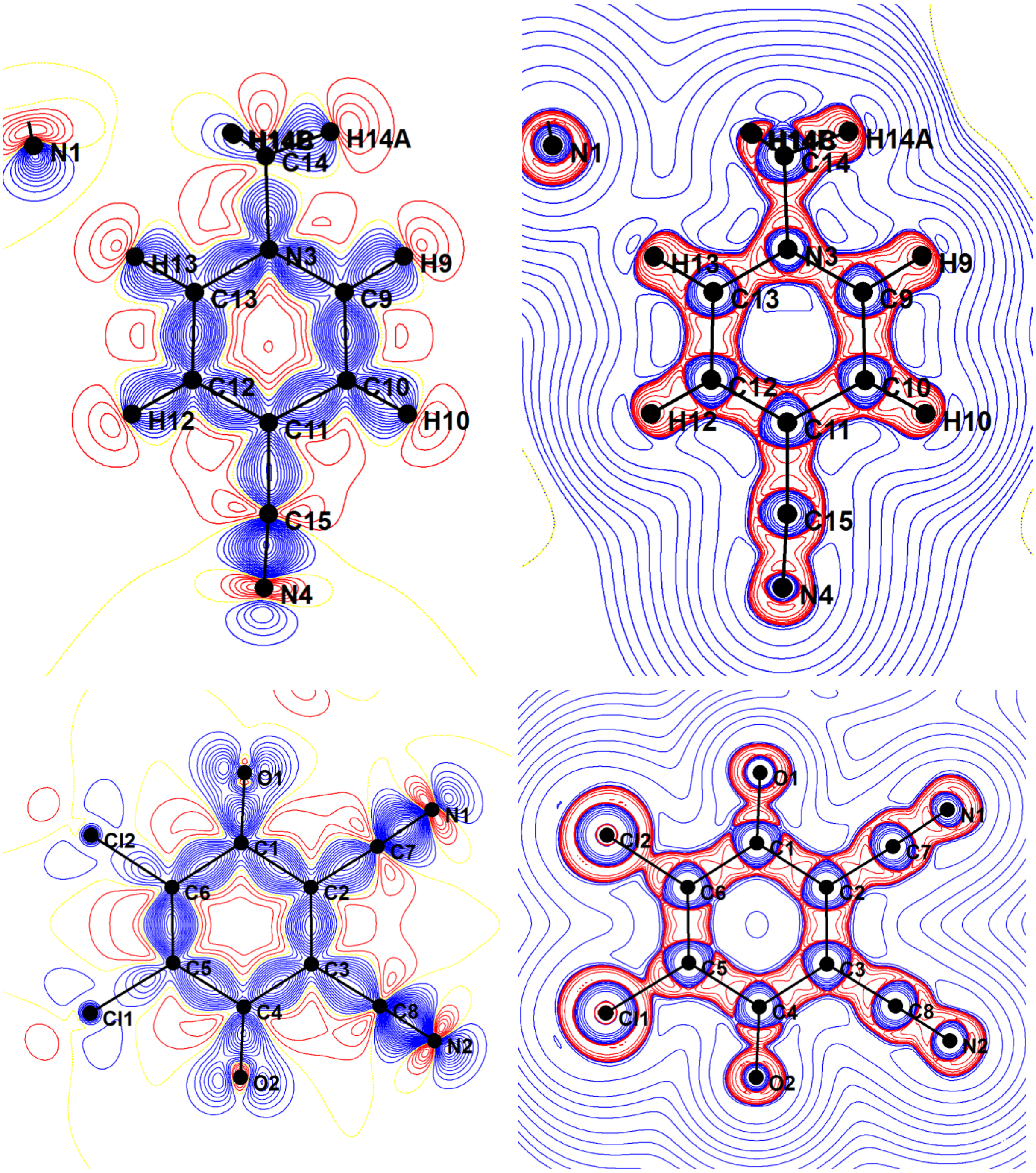

Figure S44 Maps of deformation density (left; contours at 0.05 e Å^{-3}) and Laplacian (right; contours drawn for 2, 4, 8·10^{n} e Å^{-5}, n=—3…2) in the mean plane of the 4-cyano-*N*-methylpyridinium cation (top row) and DDQ radical anion (bottom row) at 1.85 GPa.

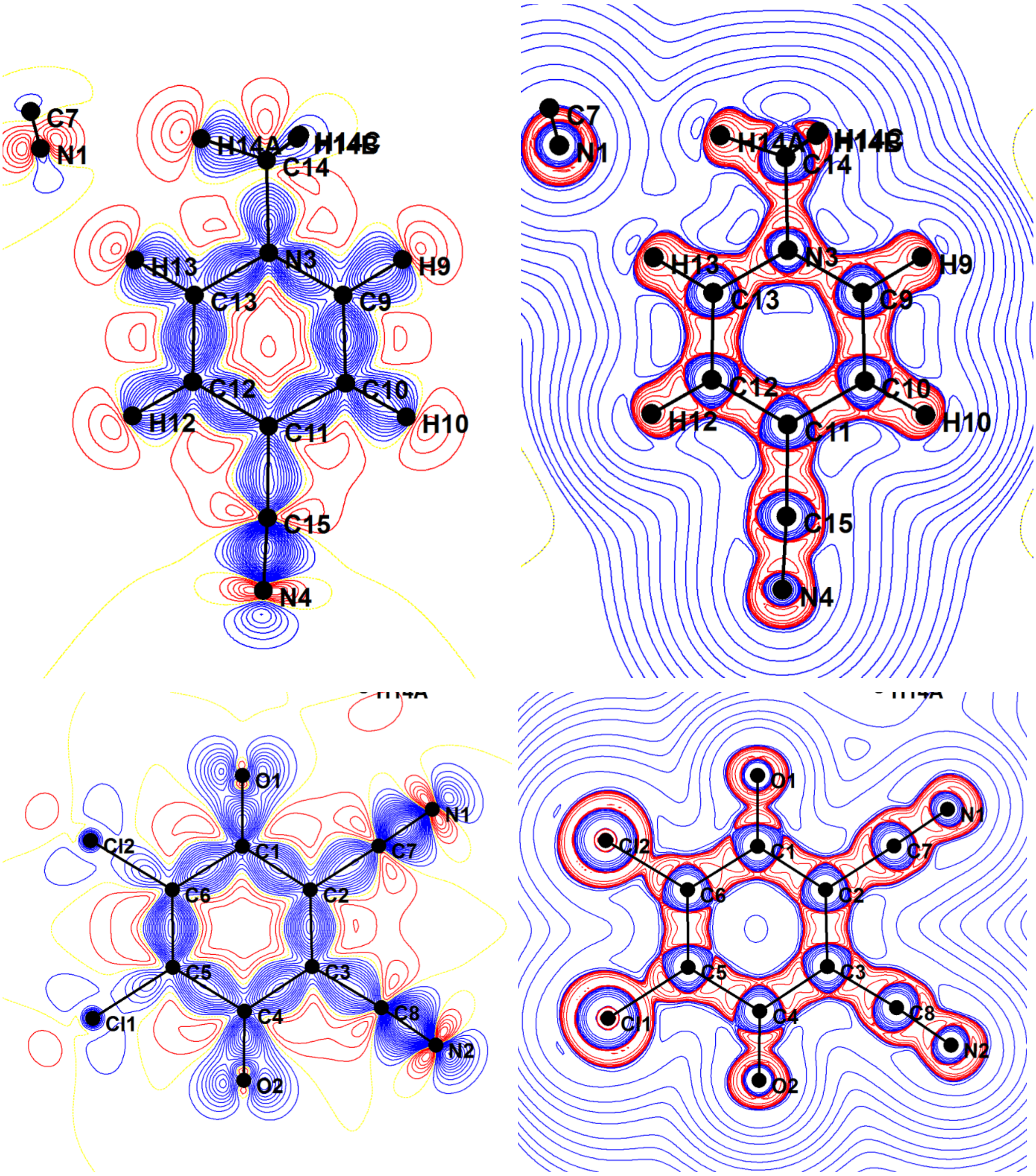

Figure S45 Maps of deformation density (left; contours at 0.05 e Å^{-3}) and Laplacian (right; contours drawn for 2, 4, 8·10^{n} e Å^{-5}, n=–3…2) in the mean plane of the 4-cyano-*N*-methylpyridinium cation (top row) and DDQ radical anion (bottom row) at 3.09 GPa.

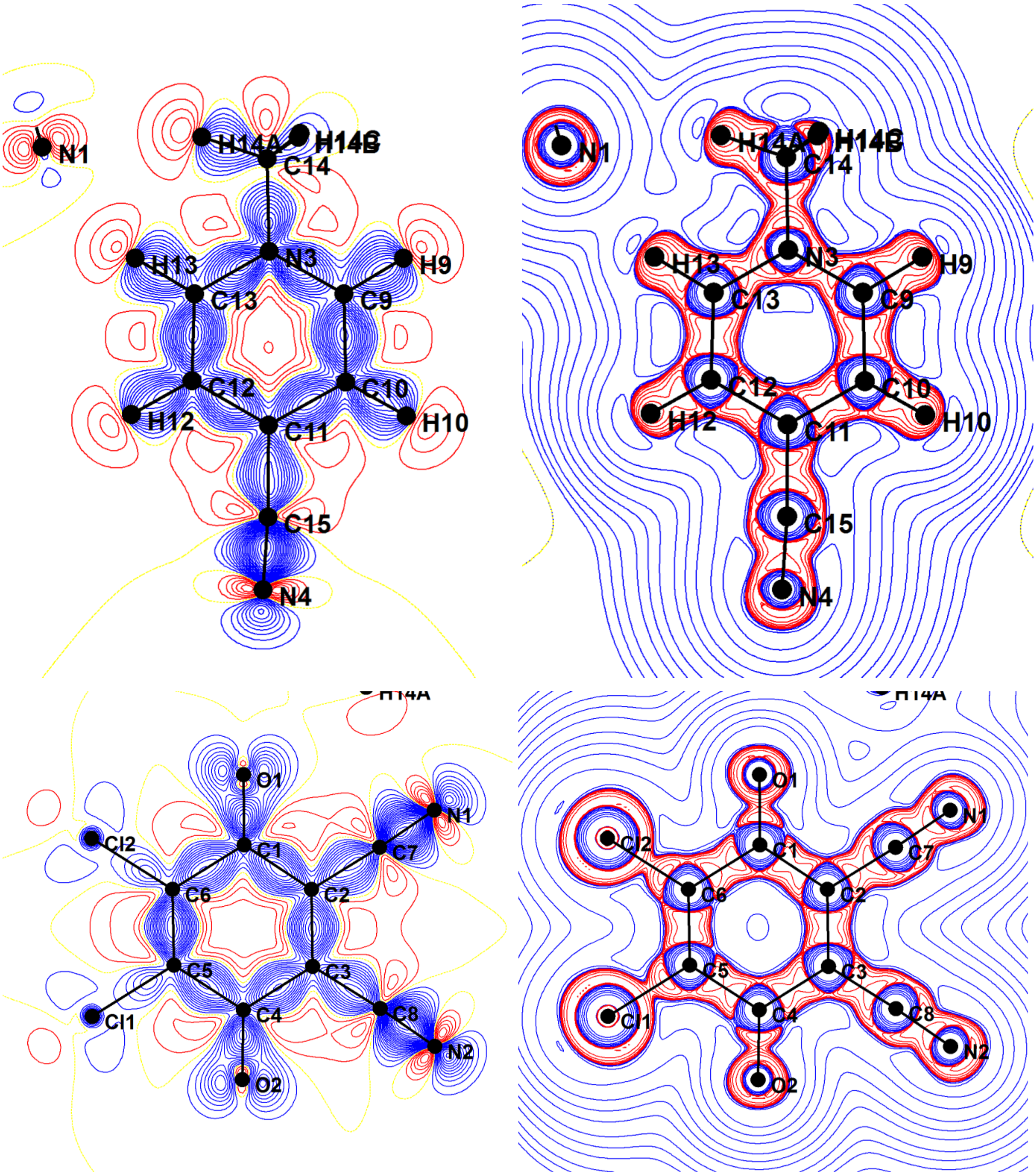

Figure S46 Maps of deformation density (left; contours at 0.05 e Å^{-3}) and Laplacian (right; contours drawn for 2, 4, 8·10^{n} e Å^{-5}, n=—3…2) in the mean plane of the 4-cyano-*N*-methylpyridinium cation (top row) and DDQ radical anion (bottom row) at 3.95 GPa.

S8 Intramolecular critical points at variable temperatures

Table S6 Topology of electron density of the 5,6-dichloro-2,3-dicyanosemiquinone radical anion, derived from electron-density after multipole refinement at 90 K and after periodic calculations (given in italic).

Bond	Length (Å)	Electron Density (eÅ^{-3}) ρ_{cp}	Laplacian (eÅ^{-5})	Ellipticity
C1–O1	1.248(3)	2.722	-29.576	0.12
		2.511	*-7.471*	*0.06*
C4–O2	1.238(3)	2.754	-28.001	0.11
		2.577	*-6.989*	*0.04*
C1–C2	1.453(4)	1.907	-16.102	0.21
		1.865	*-15.423*	*0.21*
C2–C3	1.386(4)	2.146	-20.402	0.29
		2.038	*-16.869*	*0.32*
C3–C4	1.446(4)	1.925	-16.543	0.21
		1.891	*-15.905*	*0.19*
C4–C5	1.478(4)	1.841	-14.250	0.22
		1.800	*-14.700*	*0.18*
C5–C6	1.360(4)	2.292	-23.030	0.32
		2.147	*-18.315*	*0.41*
C6–C1	1.464(4)	1.878	-15.072	0.22
		1.841	*-15.182*	*0.19*
C5–Cl1	1.712(3)	1.435	-3.807	0.12
		1.367	*-6.266*	*0.08*
C6–Cl2	1.720(3)	1.418	-3.563	0.12
		1.346	*-6.025*	*0.08*
C2–C7	1.426(4)	1.905	-13.779	0.12
		1.861	*-15.905*	*0.1*
C7–N1	1.158(4)	3.402	-20.362	0.00
		3.093	*-4.820*	*0.02*
C3–C8	1.431(4)	1.892	-13.492	0.12
		1.841	*-15.664*	*0.1*
C8–N2	1.164(4)	3.377	-21.967	0.0
		3.057	*-6.025*	*0.03*

Table S7 Topology of electron density of the 5,6-dichloro-2,3-dicyanosemiquinone radical anion, derived from electron-density after multipole refinement at 100 K and after periodic calculations (given in italic). Data are from our previous high-resolution work [2], given for comparison.

Bond	Length (Å)	Electron Density ($eÅ^{-3}$) ρ_{cp}	Laplacian ($eÅ^{-5}$)	Ellipticity	Bond order n_{topo}
C1–O1	1.2459(5)	2.710	-29.145	0.12	1.47
	1.246	*2.522*	*-7.230*	*0.06*	
C4–O2	1.2399(5)	2.761	-29.459	0.10	1.47
	1.240	*2.567*	*-7.471*	*0.04*	
C1–C2	1.4500(5)	1.912	-15.714	0.23	1.16
	1.450	*1.875*	*-15.423*	*0.21*	
C2–C3	1.3888(5)	2.133	-20.056	0.30	1.48
	1.389	*2.030*	*-16.628*	*0.32*	
C3–C4	1.4581(4)	1.906	-15.734	0.22	1.12
	1.458	*1.851*	*-15.423*	*0.19*	
C4–C5	1.4682(5)	1.855	-14.428	0.21	1.08
	1.468	*1.829*	*-15.182*	*0.18*	
C5–C6	1.3629(5)	2.273	-22.778	0.31	1.66
	1.363	*2.139*	*-18.074*	*0.41*	
C6–C1	1.4694(4)	1.876	-14.883	0.20	1.06
	1.469	*1.824*	*-14.941*	*0.19*	
C5–Cl1	1.7110(4)	1.441	-3.965	0.11	
	1.711	*1.369*	*-6.266*	*0.08*	
C6–Cl2	1.7121(4)	1.441	-4.210	0.12	
	1.712	*1.365*	*-6.266*	*0.08*	
C2–C7	1.4244(5)	1.900	-14.185	0.11	1.07
	1.424	*1.867*	*-16.146*	*0.10*	
C7–N1	1.1579(6)	3.381	-16.417	0.00	2.26
	1.158	*3.099*	*-4.820*	*0.02*	
C3–C8	1.4253(5)	1.877	-13.716	0.13	1.04
	1.425	*1.860*	*-15.905*	*0.10*	
C8–N2	1.1602(5)	3.369	-19.300	0.01	2.21
	1.160	*3.083*	*-5.302*	*0.03*	

Table S8 Topology of electron density of the 5,6-dichloro-2,3-dicyanosemiquinone radical anion, derived from electron-density after multipole refinement at 120 K and after periodic calculations (given in italic).

Bond	Length (Å)	Electron Density ($eÅ^{-3}$) ρ_{cp}	Laplacian ($eÅ^{-5}$)	Ellipticity
C1–O1	1.2502(14)	2.715	-29.900	0.12
		2.500	*-7.712*	*0.06*
C4–O2	1.2412(14)	2.743	-28.588	0.12
		2.560	*-7.712*	*0.04*
C1–C2	1.4469(16)	1.923	-16.494	0.21
		1.885	*-15.664*	*0.21*
C2–C3	1.3892(16)	2.137	-20.181	0.29
		2.029	*-16.628*	*0.32*
C3–C4	1.4563(17)	1.890	-15.933	0.21
		1.858	*-15.423*	*0.19*
C4–C5	1.4651(16)	1.875	-14.992	0.22
		1.840	*-15.182*	*0.18*
C5–C6	1.3595(16)	2.296	-23.115	0.33
		2.152	*-18.315*	*0.41*
C6–C1	1.4678(17)	1.868	-14.831	0.22
		1.829	*-15.182*	*0.19*
C5–Cl1	1.7128(13)	1.433	-3.774	0.11
		1.364	*-6.266*	*0.08*
C6–Cl2	1.7147(12)	1.429	-3.712	0.11
		1.358	*-6.025*	*0.08*
C2–C7	1.4271(17)	1.906	-13.733	0.12
		1.858	*-15.905*	*0.10*
C7–N1	1.1589(16)	3.400	-20.477	0.00
		3.093	*-4.820*	*0.02*
C3–C8	1.4281(17)	1.900	-13.687	0.13
		1.850	*-15.664*	*0.10*
C8–N2	1.1621(15)	3.387	-21.339	0.0
		3.072	*-5.543*	*0.03*

Table S9 Topology of electron density of the 5,6-dichloro-2,3-dicyanosemiquinone radical anion, derived from electron-density after multipole refinement at 150 K and after periodic calculations (given in italic).

Bond	Length (Å)	Electron Density ($eÅ^{-3}$) ρ_{cp}	Laplacian ($eÅ^{-5}$)	Ellipticity
C1–O1	1.2493(15)	2.718	-29.793	0.12
		2.505	*-7.712*	*0.06*
C4–O2	1.2408(15)	2.745	-28.529	0.13
		2.563	*-7.471*	*0.04*
C1–C2	1.4446(18)	1.930	-16.636	0.21
		1.893	*-15.905*	*0.21*
C2–C3	1.3887(18)	2.139	-20.219	0.29
		2.031	*-16.628*	*0.32*
C3–C4	1.4520(19)	1.910	-16.190	0.21
		1.871	*-15.664*	*0.19*
C4–C5	1.4641(18)	1.877	-15.050	0.22
		1.842	*-15.423*	*0.18*
C5–C6	1.3618(18)	2.288	-22.906	0.32
		2.143	*-18.074*	*0.41*
C6–C1	1.4695(19)	1.863	-14.727	0.22
		1.823	*-14.941*	*0.19*
C5–Cl1	1.7142(14)	1.430	-3.731	0.12
		1.360	*-6.025*	*0.08*
C6–Cl2	1.7127(13)	1.433	-3.781	0.12
		1.364	*-6.266*	*0.08*
C2–C7	1.4277(19)	1.902	-13.695	0.12
		1.856	*-15.905*	*0.10*
C7–N1	1.1605(17)	3.394	-20.909	0.00
		3.084	*-5.302*	*0.02*
C3–C8	1.4264(18)	1.905	-13.794	0.13
		1.856	*-15.905*	*0.10*
C8–N2	1.1627(16)	3.384	-21.527	0.00
		3.069	*-5.784*	*0.03*

Table S10 Topology of electron density of the 5,6-dichloro-2,3-dicyanosemiquinone radical anion, derived from electron-density after multipole refinement at 180 K and after periodic calculations (given in italic).

Bond	Length (Å)	Electron Density ($eÅ^{-3}$) ρ_{cp}	Laplacian ($eÅ^{-5}$)	Ellipticity
C1–O1	1.2502(16)	2.715	-29.899	0.12
		2.500	*-7.712*	*0.06*
C4–O2	1.2385(17)	2.753	-28.109	0.11
		2.574	*-7.230*	*0.04*
C1–C2	1.4456(19)	1.927	-16.574	0.21
		1.890	*-15.664*	*0.21*
C2–C3	1.3871(19)	2.144	-20.331	0.29
		2.036	*-16.869*	*0.32*
C3–C4	1.451(2)	1.914	-16.271	0.21
		1.877	*-15.664*	*0.19*
C4–C5	1.465(2)	1.876	-15.012	0.22
		1.841	*-15.182*	*0.18*
C5–C6	1.3581(19)	2.300	-23.235	0.32
		2.159	*-18.315*	*0.41*
C6–C1	1.469(2)	1.870	-14.887	0.22
		1.833	*-15.182*	*0.19*
C5–Cl1	1.7130(15)	1.433	-3.765	0.11
		1.363	*-6.266*	*0.08*
C6–Cl2	1.7121(14)	1.435	-3.796	0.11
		1.365	*-6.266*	*0.08*
C2–C7	1.425(2)	1.909	-13.846	0.12
		1.864	*-16.146*	*0.10*
C7–N1	1.1595(18)	3.398	-20.661	0.00
		3.089	*-5.061*	*0.02*
C3–C8	1.427(2)	1.903	-13.746	0.13
		1.854	*-15.664*	*0.10*
C8–N2	1.1610(18)	3.392	-21.044	0.0
		3.080	*-5.302*	*0.03*

Table S11 Topology of electron density of the 5,6-dichloro-2,3-dicyanosemiquinone radical anion, derived from electron-density after multipole refinement at 240 K and after periodic calculations (given in italic).

Bond	Length (Å)	Electron Density ($eÅ^{-3}$) ρ_{cp}	Laplacian ($eÅ^{-5}$)	Ellipticity
C1–O1	1.2502(15)	2.715	-29.900	0.12
		2.500	*-7.953*	*0.06*
C4–O2	1.2374(15)	2.756	-27.902	0.11
		2.579	*-6.989*	*0.04*
C1–C2	1.4443(18)	1.931	-16.658	0.21
		1.894	*-15.905*	*0.21*
C2–C3	1.3884(17)	2.140	-20.238	0.29
		2.031	*-16.869*	*0.32*
C3–C4	1.4553(18)	1.902	-15.993	0.21
		1.861	*-15.423*	*0.19*
C4–C5	1.4647(18)	1.876	-15.012	0.22
		1.840	*-15.182*	*0.18*
C5–C6	1.3619(18)	2.288	-22.904	0.32
		2.143	*-18.074*	*0.41*
C6–C1	1.4659(18)	1.873	-14.946	0.22
		1.836	*-15.182*	*0.19*
C5–Cl1	1.7114(13)	1.437	-3.820	0.12
		1.367	*-6.266*	*0.08*
C6–Cl2	1.7134(13)	1.432	-3.759	0.12
		1.362	*-6.025*	*0.08*
C2–C7	1.4255(19)	1.908	-13.834	0.12
		1.863	*-15.905*	*0.10*
C7–N1	1.1606(16)	3.393	-20.956	0.00
		3.083	*-5.302*	*0.02*
C3–C8	1.4260(18)	1.906	-13.818	0.13
		1.858	*-15.905*	*0.1*
C8–N2	1.1618(16)	3.388	-21.285	0.00
		3.074	*-5.543*	*0.03*

Table S12 Topology of electron density of the 5,6-dichloro-2,3-dicyanosemiquinone radical anion, derived from electron-density after multipole refinement at 240 K and after periodic calculations (given in italic).

Bond	Length (Å)	Electron Density ($eÅ^{-3}$) ρ_{cp}	Laplacian ($eÅ^{-5}$)	Ellipticity
C1–O1	1.2496(15)	2.717	-29.826	0.12
		2.503	*-7.712*	*0.06*
C4–O2	1.2381(15)	2.754	-28.040	0.11
		2.577	*-6.989*	*0.04*
C1–C2	1.4456(18)	1.927	-16.580	0.21
		1.890	*-15.664*	*0.21*
C2–C3	1.3890(18)	2.138	-20.194	0.29
		2.030	*-16.628*	*0.32*
C3–C4	1.4519(19)	1.911	-16.198	0.21
		1.873	*-15.664*	*0.19*
C4–C5	1.4649(18)	1.875	-14.999	0.22
		1.840	*-15.182*	*0.18*
C5–C6	1.3584(18)	2.299	-23.210	0.32
		2.157	*-18.315*	*0.41*
C6–C1	1.4712(18)	1.858	-14.627	0.22
		1.818	*-14.941*	*0.19*
C5–Cl1	1.7115(14)	1.437	-3.813	0.11
		1.366	*-6.266*	*0.08*
C6–Cl2	1.7120(14)	1.435	-3.800	0.11
		1.365	*-6.266*	*0.08*
C2–C7	1.426(2)	1.907	-13.811	0.12
		1.860	*-15.905*	*0.10*
C7–N1	1.1610(17)	3.391	-21.066	0.00
		3.081	*-5.543*	*0.02*
C3–C8	1.4270(19)	1.903	-13.753	0.13
		1.855	*-15.905*	*0.10*
C8–N2	1.1597(17)	3.397	-20.715	0.0
		3.086	*-5.061*	*0.03*

Table S13 Topology of electron density of the 5,6-dichloro-2,3-dicyanosemiquinone radical anion, derived from electron-density after multipole refinement at 270 K and after periodic calculations (given in italic).

Bond	Length (Å)	Electron Density ($eÅ^{-3}$) ρ_{cp}	Laplacian ($eÅ^{-5}$)	Ellipticity
C1–O1	1.2491(15)	2.718	-29.762	0.12
		2.506	*-7.712*	*0.06*
C4–O2	1.2357(15)	2.762	-27.582	0.11
		2.588	*-6.748*	*0.04*
C1–C2	1.4446(18)	1.930	-16.638	0.21
		1.894	*-15.905*	*0.21*
C2–C3	1.3883(17)	2.140	-20.241	0.29
		2.032	*-16.869*	*0.32*
C3–C4	1.4510(18)	1.913	-16.251	0.21
		1.875	*-15.664*	*0.19*
C4–C5	1.4650(18)	1.875	-14.991	0.22
		1.840	*-15.182*	*0.18*
C5–C6	1.3572(18)	2.303	-23.311	0.32
		2.161	*-18.556*	*0.41*
C6–C1	1.4705(17)	1.861	-14.669	0.22
		1.821	*-14.941*	*0.19*
C5–Cl1	1.7100(14)	1.440	-3.860	0.11
		1.370	*-6.266*	*0.08*
C6–Cl2	1.7089(13)	1.443	-3.900	0.11
		1.373	*-6.266*	*0.08*
C2–C7	1.4253(19)	1.909	-13.846	0.12
		1.863	*-15.905*	*0.10*
C7–N1	1.1585(17)	3.402	-20.389	0.00
		3.095	*-5.061*	*0.02*
C3–C8	1.4263(18)	1.906	-13.797	0.13
		1.856	*-15.905*	*0.10*
C8–N2	1.1593(16)	3.398	-20.603	0.00
		3.089	*-5.061*	*0.03*

Table S14 Topology of electron density of the 5,6-dichloro-2,3-dicyanosemiquinone radical anion, derived from electron-density after multipole refinement at 293 K and after periodic calculations (given in italic).

Bond	Length (Å)	Electron Density ($eÅ^{-3}$) ρ_{cp}	Laplacian ($eÅ^{-3}$)	Ellipticity
C1–O1	1.2497(15)	2.717	-29.846	0.12
		2.502	*-7.712*	*0.06*
C4–O2	1.2386(16)	2.752	-28.134	0.11
		2.573	*-7.230*	*0.04*
C1–C2	1.4440(18)	1.932	-16.683	0.21
		1.894	*-15.905*	*0.21*
C2–C3	1.3880(18)	2.141	-20.258	0.29
		2.034	*-16.869*	*0.32*
C3–C4	1.452(2)	1.910	-16.185	0.21
		1.871	*-15.664*	*0.19*
C4–C5	1.4633(19)	1.880	-15.093	0.22
		1.845	*-15.423*	*0.18*
C5–C6	1.3576(18)	2.302	-23.276	0.32
		2.160	*-18.556*	*0.41*
C6–C1	1.471(2)	1.859	-14.643	0.22
		1.819	*-14.941*	*0.19*
C5–Cl1	1.7116(14)	1.437	-3.810	0.11
		1.366	*-6.266*	*0.08*
C6–Cl2	1.7087(14)	1.443	-3.906	0.11
		1.374	*-6.266*	*0.08*
C2–C7	1.422(2)	1.917	-14.024	0.12
		1.874	*-16.146*	*0.10*
C7–N1	1.1554(18)	3.415	-19.540	0.00
		3.114	*-4.338*	*0.02*
C3–C8	1.4250(19)	1.909	-13.875	0.13
		1.862	*-15.905*	*0.10*
C8–N2	1.1577(17)	3.405	-20.165	0.00
		3.097	*-4.820*	*0.03*

Table S15 Topology of electron density of the 5,6-dichloro-2,3-dicyanosemiquinone radical anion, derived from electron-density after multipole refinement at 310 K and after periodic calculations (given in italic).

Bond	Length (Å)	Electron Density ($eÅ^{-3}$) ρ_{cp}	Laplacian ($eÅ^{-5}$)	Ellipticity
C1–O1	1.2461(16)	2.728	-29.358	0.12
		2.521	*-7.230*	*0.06*
C4–O2	1.2373(17)	2.757	-27.907	0.11
		2.580	*-6.989*	*0.04*
C1–C2	1.438(2)	1.948	-17.010	0.21
		1.894	*-15.905*	*0.21*
C2–C3	1.3764(19)	2.175	-21.118	0.29
		2.036	*-16.869*	*0.32*
C3–C4	1.458 (2)	1.894	-15.790	0.21
		1.881	*-15.664*	*0.19*
C4–C5	1.467(2)	1.868	-14.839	0.22
		1.846	*-15.423*	*0.18*
C5–C6	1.3564(19)	2.305	-23.387	0.32
		2.159	*-18.315*	*0.41*
C6–C1	1.463(2)	1.882	-15.117	0.22
		1.823	*-14.941*	*0.19*
C5–Cl1	1.7098(15)	1.441	-3.866	0.11
		1.371	*-6.266*	*0.08*
C6–Cl2	1.7052(14)	1.451	-4.021	0.11
		1.383	*-6.266*	*0.08*
C2–C7	1.421(2)	1.924	-14.115	0.12
		1.872	*-16.146*	*0.10*
C7–N1	1.1564(19)	3.411	-19.828	0.00
		3.126	*-4.097*	*0.02*
C3–C8	1.419(2)	1.926	-14.241	0.13
		1.877	*-16.146*	*0.10*
C8–N2	1.1639(18)	3.380	-21.873	0.00
		3.095	*-5.061*	*0.03*

Table S16 Topology of electron density of the 5,6-dichloro-2,3-dicyanosemiquinone radical anion, derived from electron-density after multipole refinement at 340 K and after periodic calculations (given in italic).

Bond	Length (Å)	Electron Density ($eÅ^{-3}$) ρ_{cp}	Laplacian ($eÅ^{-5}$)	Ellipticity
C1–O1	1.242(2)	2.743	-28.713	0.12
		2.542	*-6.748*	*0.05*
C4–O2	1.2355(16)	2.761	-27.509	0.11
		2.588	*-6.507*	*0.04*
C1–C2	1.438(2)	1.948	-17.010	0.21
		1.912	*-16.146*	*0.21*
C2–C3	1.376(2)	2.175	-21.118	0.29
		2.077	*-17.592*	*0.31*
C3–C4	1.458(2)	1.894	-15.790	0.21
		1.851	*-15.182*	*0.18*
C4–C5	1.467(2)	1.868	-14.839	0.22
		1.833	*-15.182*	*0.18*
C5–C6	1.356(2)	2.305	-23.387	0.32
		2.165	*-18.556*	*0.41*
C6–C1	1.463(2)	1.882	-15.117	0.22
		1.846	*-15.423*	*0.19*
C5–Cl1	1.718(2)	1.422	-3.613	0.11
		1.349	*-6.025*	*0.08*
C6–Cl2	1.7162(15)	1.425	-3.663	0.11
		1.354	*-6.025*	*0.08*
C2–C7	1.421(2)	1.924	-14.115	0.12
		1.880	*-16.387*	*0.10*
C7–N1	1.156(3)	3.411	-19.828	0.00
		3.108	*-4.820*	*0.02*
C3–C8	1.419(3)	1.926	-14.241	0.13
		1.882	*-16.146*	*0.10*
C8–N2	1.164(2)	3.380	-21.873	0.00
		3.064	*-6.025*	*0.03*

Table S17 Topology of electron density of the 5,6-dichloro-2,3-dicyanosemiquinone radical anion, derived from electron-density after multipole refinement at 370 K and after periodic calculations (given in italic).

Bond	Length (Å)	Electron Density ($eÅ^{-3}$) ρ_{cp}	Laplacian ($eÅ^{-5}$)	Ellipticity
C1–O1	1.246(2)	2.727	-29.416	0.12
		2.523	*-7.230*	*0.05*
C4–O2	1.237(2)	2.759	-27.762	0.11
		2.583	*-6.748*	*0.04*
C1–C2	1.444(3)	1.931	-16.656	0.21
		1.899	*-15.905*	*0.21*
C2–C3	1.387(3)	2.144	-20.324	0.29
		2.031	*-16.869*	*0.31*
C3–C4	1.459(3)	1.919	-16.364	0.21
		1.863	*-15.423*	*0.19*
C4–C5	1.463(3)	1.890	-15.109	0.22
		1.860	*-15.664*	*0.18*
C5–C6	1.358(3)	2.301	-23.255	0.32
		2.148	*-18.315*	*0.41*
C6–C1	1.470(3)	1.862	-14.696	0.22
		1.813	*-14.941*	*0.19*
C5–Cl1	1.715(2)	1.427	-3.688	0.11
		1.354	*-6.025*	*0.08*
C6–Cl2	1.709(2)	1.441	-3.894	0.11
		1.374	*-6.266*	*0.08*
C2–C7	1.423(3)	1.916	-13.991	0.12
		1.871	*-16.146*	*0.10*
C7–N1	1.153(3)	3.424	-18.973	0.00
		3.120	*-4.338*	*0.02*
C3–C8	1.420(3)	1.922	-14.155	0.13
		1.898	*-16.387*	*0.10*
C8–N2	1.158(3)	3.403	-20.339	0.00
		3.103	*-4.820*	*0.03*

Table S18 Topology of electron density of the 4-cyano-*N*-methylpyridinium cation, derived from electron-density after multipole refinement at 90 K and after periodic calculations (given in italic).

Bond	Length (Å)	Electron Density (eÅ^{-3}) ρ_{cp}	Laplacian (eÅ^{-5})	Ellipticity
N3–C9	1.345(4)	2.252	-22.285	0.08
		2.131	*-15.182*	*0.05*
N3–C13	1.354(4)	2.227	-21.676	0.09
		2.099	*-15.664*	*0.05*
N3–C14	1.475(4)	1.701	-9.565	0.05
		1.602	*-10.844*	*0.03*
C9–C10	1.388(4)	2.213	-21.282	0.18
		2.065	*-17.833*	*0.23*
C10–C11	1.398(4)	2.068	-19.067	0.22
		2.060	*-17.592*	*0.23*
C11–C12	1.385(4)	2.104	-20.008	0.22
		2.070	*-17.833*	*0.21*
C12–C13	1.390(5)	2.208	-21.150	0.18
		2.060	*-17.592*	*0.23*
C11–C15	1.429(4)	1.918	-13.647	0.15
		1.874	*-16.628*	*0.05*
C15–N4	1.162(4)	3.388	-21.300	0.00
		3.075	*-4.579*	*0.03*
C9–H9	1.08(17)	1.806	-19.656	0.06
		1.903	*-22.412*	*0.02*
C10–H10	1.08(19)	1.769	-18.932	0.05
		1.898	*-21.930*	*0.01*
C12–H12	1.08(17)	1.770	-18.935	0.05
		1.865	*-20.966*	*0.01*
C13–H13	1.08(19)	1.805	-19.648	0.06
		1.930	*-23.135*	*0.02*
C14–H14A	1.08(16)	1.686	-16.483	0.09
		1.861	*-20.243*	*0.05*
C14–H14B	1.08(19)	1.686	-16.476	0.09
		1.887	*-21.207*	*0.04*
C14–H14C	1.08(10)	1.687	-16.479	0.09
		1.883	*-20.725*	*0.05*

Table S19 Topology of electron density of the 4-cyano-*N*-methylpyridinium cation, derived from electron-density after multipole refinement at 100 K and after periodic calculations (given in italic). Data are from our previous high-resolution work [2], given for comparison.

Bond	Length (Å)	Electron Density ($eÅ^{-3}$) ρ_{cp}	Laplacian ($eÅ^{-5}$)	Ellipticity	Bond order n_{topo}
N3–C9	1.3469(6)	2.254	-22.897	0.10	1.29
	1.347	*2.126*	*-15.182*	*0.05*	
N3–C13	1.3484(6)	2.275	-22.444	0.10	1.32
	1.348	*2.124*	*-15.664*	*0.05*	
N3–C14	1.4754(7)	1.721	-10.633	0.04	1.02
	1.476	*1.601*	*-10.603*	*0.03*	
C9–C10	1.3810(6)	2.218	-21.328	0.16	1.57
	1.381	*2.091*	*-18.315*	*0.23*	
C10–C11	1.3928(6)	2.120	-18.638	0.18	1.38
	1.393	*2.039*	*-17.351*	*0.20*	
C11–C12	1.3937(6)	2.144	-19.323	0.18	1.41
	1.394	*2.036*	*-17.351*	*0.21*	
C12–C13	1.3800(6)	2.235	-21.310	0.20	1.57
	1.380	*2.093*	*-18.315*	*0.24*	
C11–C15	1.4366(6)	1.872	-13.385	0.06	1.01
	1.437	*1.848*	*-16.146*	*0.05*	
C15–N4	1.1559(7)	3.303	-14.668	0.01	2.23
	1.156	*3.108*	*-3.133*	*0.03*	
C9–H9	1.081(7)	1.870	-18.868	0.06	1.00
	1.081	*1.907*	*-22.412*	*0.02*	
C10–H10	1.081(5)	1.828	-17.202	0.04	0.94
	1.081	*1.882*	*-21.689*	*0.01*	
C12–H12	1.081(7)	1.807	-17.986	0.03	0.89
	1.081	*1.874*	*-21.207*	*0.01*	
C13–H13	1.081(5)	1.854	-19.317	0.05	0,98
	1.081	*1.907*	*-22.653*	*0.02*	
C14–H14A	1.077(7)	1.761	-15.975	0.08	0.97
	1.077	*1.891*	*-20.966*	*0.04*	
C14–H14B	1.076(7)	1.699	-15.388	0.10	0.95
	1.076	*1.886*	*-20.725*	*0.05*	
C14–H14C	1.075(6)	1.776	-16.492	0.08	0.97
	1.075	*1.900*	*-21.448*	*0.04*	

Table S20 Topology of electron density of the 4-cyano-*N*-methylpyridinium cation, derived from electron-density after multipole refinement at 120 K and after periodic calculations (given in italic).

Bond	Length (Å)	Electron Density ($eÅ^{-3}$) ρ_{cp}	Laplacian ($eÅ^{-5}$)	Ellipticity
N3–C9	1.3473(16)	2.246	-22.133	0.08
		2.123	*-15.423*	*0.05*
N3–C13	1.3469(16)	2.248	-22.158	0.08
		2.126	*-15.423*	*0.05*
N3–C14	1.4794(17)	1.692	-9.256	0.05
		1.586	*-10.362*	*0.04*
C9–C10	1.3803(18)	2.237	-21.872	0.18
		2.097	*-18.315*	*0.23*
C10–C11	1.3891(17)	2.094	-19.716	0.22
		2.054	*-17.833*	*0.20*
C11–C12	1.3890(17)	2.094	-19.720	0.22
		2.057	*-17.833*	*0.21*
C12–C13	1.3797(18)	2.239	-21.920	0.18
		2.097	*-18.315*	*0.23*
C11–C15	1.4393(18)	1.889	-13.002	0.15
		1.838	*-16.146*	*0.04*
C15–N4	1.1579(16)	3.403	-20.170	0.00
		3.095	*-3.615*	*0.03*
C9–H9	1.08(16)	1.806	-19.656	0.06
		1.908	*-22.653*	*0.02*
C10–H10	1.08(19)	1.770	-18.937	0.05
		1.887	*-21.689*	*0.01*
C12–H12	1.08(16)	1.770	-18.937	0.05
		1.869	*-20.966*	*0.01*
C13–H13	1.08(19)	1.806	-19.651	0.06
		1.919	*-22.894*	*0.02*
C14–H14A	1.08(10)	1.686	-16.448	0.08
		1.902	*-21.448*	*0.04*
C14–H14B	1.08(19)	1.680	-16.310	0.07
		1.878	*-20.484*	*0.04*
C14–H14C	1.08(17)	1.684	-16.457	0.09
		1.908	*-21.448*	*0.05*

Table S21 Topology of electron density of the 4-cyano-*N*-methylpyridinium cation, derived from electron-density after multipole refinement at 150 K and after periodic calculations (given in italic).

Bond	Length (Å)	Electron Density ($eÅ^{-3}$) ρ_{cp}	Laplacian ($eÅ^{-5}$)	Ellipticity
N3–C9	1.3461(17)	2.250	-22.202	0.08
		2.129	*-15.423*	*0.05*
N3–C13	1.3470(17)	2.249	-22.132	0.08
		2.128	*-15.423*	*0.05*
N3–C14	1.4794(19)	1.690	-9.293	0.05
		1.589	*-10.844*	*0.03*
C9–C10	1.3831(19)	2.228	-21.666	0.18
		2.084	*-18.074*	*0.23*
C10–C11	1.3831(18)	2.093	-19.688	0.22
		2.054	*-17.592*	*0.2*
C11–C12	1.3895(18)	2.093	-19.685	0.22
		2.054	*-17.592*	*0.21*
C12–C13	1.3802(19)	2.237	-21.900	0.18
		2.092	*-18.074*	*0.24*
C11–C15	1.435(2)	1.901	-13.259	0.15
		1.853	*-16.387*	*0.05*
C15–N4	1.1575(15)	3.406	-20.057	0.00
		3.098	*-3.615*	*0.03*
C9–H9	1.083(2)	1.808	-19.702	0.06
		1.900	*-22.412*	*0.02*
C10–H10	1.083(2)	1.767	-18.856	0.05
		1.871	*-21.448*	*0.01*
C12–H12	1.083(2)	1.771	-18.961	0.05
		1.867	*-20.966*	*0.01*
C13–H13	1.083(2)	1.807	-19.665	0.06
		1.899	*-22.412*	*0.02*
C14–H14A	1.0770(14)	1.619	-15.025	0.12
		1.879	*-20.484*	*0.05*
C14–H14B	1.0770(14)	1.700	-17.009	0.12
		1.893	*-21.207*	*0.04*
C14–H14C	1.0770(14)	1.718	-17.580	0.12
		1.888	*-20.725*	*0.05*

Table S22 Topology of electron density of the 4-cyano-*N*-methylpyridinium cation, derived from electron-density after multipole refinement at 180 K and after periodic calculations (given in italic).

Bond	Length (Å)	Electron Density ($eÅ^{-3}$) ρ_{cp}	Laplacian ($eÅ^{-5}$)	Ellipticity
N3–C9	1.3457(19)	2.251	-22.235	0.08
		2.128	*-15.182*	*0.05*
N3–C13	1.344(2)	2.257	-22.337	0.08
		2.140	*-15.423*	*0.05*
N3–C14	1.474(2)	1.704	-9.632	0.05
		1.607	*-10.844*	*0.03*
C9–C10	1.382(2)	2.233	-21.759	0.18
		2.091	*-18.315*	*0.23*
C10–C11	1.385(2)	2.106	-20.008	0.22
		2.069	*-18.074*	*0.2*
C11–C12	1.387(2)	2.101	-19.886	0.22
		2.064	*-17.833*	*0.21*
C12–C13	1.379(2)	2.241	-21.981	0.18
		2.101	*-18.315*	*0.23*
C11–C15	1.432(2)	1.911	-13.458	0.15
		1.865	*-16.628*	*0.05*
C15–N4	1.1598(19)	3.396	-20.704	0.00
		3.085	*-4.097*	*0.03*
C9–H9	1.08(19)	1.806	-19.657	0.06
		1.917	*-22.894*	*0.02*
C10–H10	1.08(19)	1.770	-18.936	0.05
		1.875	*-21.448*	*0.01*
C12–H12	1.08(19)	1.771	-18.938	0.05
		1.855	*-20.725*	*0.01*
C13–H13	1.08(19)	1.806	-19.655	0.06
		1.902	*-22.653*	*0.02*
C14–H14A	1.08(12)	1.686	-16.478	0.09
		1.859	*-20.243*	*0.05*
C14–H14B	1.08(19)	1.687	-16.479	0.09
		1.852	*-20.002*	*0.05*
C14–H14C	1.08(19)	1.687	-16.480	0.09
		1.894	*-21.207*	*0.04*

Table S23 Topology of electron density of the 4-cyano-*N*-methylpyridinium cation, derived from electron-density after multipole refinement at 210 K and after periodic calculations (given in italic).

Bond	Length (Å)	Electron Density ($eÅ^{-3}$) ρ_{cp}	Laplacian ($eÅ^{-5}$)	Ellipticity
N3–C9	1.3463(17)	2.251	-22.178	0.08
		2.128	*-15.182*	*0.05*
N3–C13	1.3453(18)	2.254	-22.236	0.08
		2.136	*-15.423*	*0.05*
N3–C14	1.475(2)	1.705	-9.471	0.05
		1.602	*-10.844*	*0.03*
C9–C10	1.3806(19)	2.236	-21.857	0.18
		2.092	*-18.315*	*0.23*
C10–C11	1.3847(18)	2.107	-20.034	0.22
		2.071	*-18.074*	*0.20*
C11–C12	1.3863(19)	2.103	-19.914	0.22
		2.066	*-17.833*	*0.21*
C12–C13	1.376(2)	2.249	-22.207	0.18
		2.108	*-18.556*	*0.23*
C11–C15	1.433(2)	1.908	-13.392	0.15
		1.863	*-16.628*	*0.05*
C15–N4	1.1555(18)	3.414	-19.541	0.00
		3.108	*-3.374*	*0.03*
C9–H9	1.083(2)	1.806	-19.643	0.06
		1.899	*-22.412*	*0.02*
C10–H10	1.083(2)	1.769	-18.902	0.05
		1.869	*-21.448*	*0.01*
C12–H12	1.082(2)	1.770	-18.923	0.05
		1.865	*-20.966*	*0.01*
C13–H13	1.083(2)	1.808	-19.694	0.06
		1.901	*-22.412*	*0.02*
C14–H14A	1.0770(14)	1.615	-15.138	0.20
		1.881	*-20.725*	*0.05*
C14–H14B	1.0770(14)	1.882	-24.975	0.17
		1.892	*-21.207*	*0.04*
C14–H14C	1.0770(14)	1.670	-16.496	0.20
		1.888	*-20.725*	*0.05*

Table S24 Topology of electron density of the 4-cyano-*N*-methylpyridinium cation, derived from electron-density after multipole refinement at 240 K and after periodic calculations (given in italic).

Bond	Length (Å)	Electron Density ($eÅ^{-3}$) ρ_{cp}	Laplacian ($eÅ^{-5}$)	Ellipticity
N3–C9	1.3438(18)	2.259	-22.342	0.08
		2.134	*-15.182*	*0.05*
N3–C13	1.3432(19)	2.259	-22.376	0.08
		2.137	*-15.423*	*0.05*
N3–C14	1.477(2)	1.695	-9.433	0.05
		1.595	*-10.844*	*0.03*
C9–C10	1.381(2)	2.234	-21.782	0.18
		2.091	*-18.315*	*0.23*
C10–C11	1.3859(19)	2.104	-19.948	0.22
		2.063	*-17.833*	*0.2*
C11–C12	1.384(2)	2.110	-20.102	0.22
		2.074	*-18.074*	*0.21*
C12–C13	1.382(2)	2.233	-21.757	0.18
		2.090	*-18.074*	*0.23*
C11–C15	1.432(2)	1.911	-13.462	0.15
		1.866	*-16.628*	*0.05*
C15–N4	1.1599(19)	3.396	-20.755	0.00
		3.085	*-4.097*	*0.03*
C9–H9	1.08(17)	1.806	-19.653	0.06
		1.904	*-22.412*	*0.02*
C10–H10	1.08(19)	1.770	-18.937	0.05
		1.849	*-20.966*	*0.01*
C12–H12	1.08(18)	1.770	-18.938	0.05
		1.861	*-20.966*	*0.01*
C13–H13	1.08(19)	1.806	-19.655	0.06
		1.882	*-22.171*	*0.02*
C14–H14A	1.08(11)	1.686	-16.483	0.09
		1.883	*-20.725*	*0.05*
C14–H14B	1.08(17)	1.686	-16.478	0.09
		1.881	*-20.725*	*0.05*
C14–H14C	1.08(19)	1.686	-16.477	0.09
		1.888	*-21.207*	*0.04*

Table S25 Topology of electron density of the 4-cyano-*N*-methylpyridinium cation, derived from electron-density after multipole refinement at 270 K and after periodic calculations (given in italic).

Bond	Length (Å)	Electron Density ($eÅ^{-3}$) ρ_{cp}	Laplacian ($eÅ^{-5}$)	Ellipticity
N3–C9	1.3448(18)	2.254	-22.273	0.08
		2.132	*-15.182*	*0.05*
N3–C13	1.3447(19)	2.255	-22.281	0.08
		2.138	*-15.423*	*0.05*
N3–C14	1.479(2)	1.690	-9.305	0.05
		1.589	*-10.603*	*0.03*
C9–C10	1.374(2)	2.256	-22.348	0.18
		2.120	*-18.797*	*0.23*
C10–C11	1.3798(16)	2.122	-20.395	0.22
		2.091	*-18.315*	*0.20*
C11–C12	1.382(2)	2.114	-20.199	0.22
		2.082	*-18.074*	*0.21*
C12–C13	1.386(2)	2.250	-22.204	0.18
		2.112	*-18.556*	*0.23*
C11–C15	1.433(2)	1.909	-13.399	0.15
		1.862	*-16.628*	*0.04*
C15–N4	1.1576(18)	3.405	-20.104	0.00
		3.099	*-3.615*	*0.03*
C9–H9	1.08(16)	1.807	-19.659	0.06
		1.921	*-22.894*	*0.02*
C10–H10	1.08(19)	1.771	-18.941	0.05
		1.838	*-20.725*	*0.01*
C12–H12	1.08(19)	1.771	-18.939	0.05
		1.861	*-20.966*	*0.01*
C13–H13	1.08(19)	1.806	-19.654	0.06
		1.865	*-21.689*	*0.02*
C14–H14A	1.08(10)	1.686	-16.480	0.09
		1.904	*-21.207*	*0.05*
C14–H14B	1.08(16)	1.686	-16.480	0.09
		1.890	*-20.725*	*0.05*
C14–H14C	1.08(19)	1.686	-16.477	0.09
		1.892	*-21.207*	*0.05*

Table S26 Topology of electron density of the 4-cyano-*N*-methylpyridinium cation, derived from electron-density after multipole refinement at 293 K and after periodic calculations (given in italic).

Bond	Length (Å)	Electron Density ($eÅ^{-3}$) ρ_{cp}	Laplacian ($eÅ^{-5}$)	Ellipticity
N3–C9	1.343(2)	2.258	-22.351	0.08
		2.137	*-14.941*	*0.05*
N3–C13	1.340(3)	2.268	-22.521	0.08
		2.154	*-15.182*	*0.05*
N3–C14	1.474(3)	1.704	-9.631	0.05
		1.606	*-10.844*	*0.03*
C9–C10	1.376(3)	2.249	-22.165	0.18
		2.110	*-18.556*	*0.23*
C10–C11	1.384(3)	2.110	-20.086	0.22
		2.075	*-18.074*	*0.20*
C11–C12	1.380(3)	2.120	-20.365	0.22
		2.091	*-18.315*	*0.21*
C12–C13	1.384(3)	2.227	-21.585	0.18
		2.083	*-18.074*	*0.23*
C11–C15	1.434(3)	1.905	-13.315	0.15
		3.127	*-2.651*	*0.03*
C15–N4	1.152(3)	3.427	-18.656	0.00
		3.127	*-2.651*	*0.03*
C9–H9	1.08(17)	1.807	-19.656	0.06
		1.910	*-22.653*	*0.02*
C10–H10	1.08(19)	1.771	-18.939	0.05
		1.904	*-22.171*	*0.01*
C12–H12	1.08(18)	1.770	-18.937	0.05
		1.856	*-20.725*	*0.01*
C13–H13	1.08(19)	1.806	-19.651	0.06
		1.921	*-22.894*	*0.02*
C14–H14A	1.08(11)	1.686	-16.483	0.09
		1.898	*-20.966*	*0.05*
C14–H14B	1.08(19)	1.686	-16.479	0.09
		1.871	*-20.484*	*0.05*
C14–H14C	1.08(17)	1.686	-16.475	0.09
		1.894	*-21.207*	*0.05*

Table S27 Topology of electron density of the 4-cyano-*N*-methylpyridinium cation, derived from electron-density after multipole refinement at 310 K and after periodic calculations (given in italic).

Bond	Length (Å)	Electron Density ($eÅ^{-3}$) ρ_{cp}	Laplacian ($eÅ^{-5}$)	Ellipticity
N3–C9	1.337(2)	2.277	-22.610	0.07
		2.164	*-14.700*	*0.05*
N3–C13	1.338(2)	2.274	-22.581	0.08
		2.163	*-14.941*	*0.05*
N3–C14	1.476(3)	1.698	-9.490	0.05
		1.599	*-10.844*	*0.03*
C9–C10	1.378(2)	2.246	-22.074	0.18
		2.105	*-18.556*	*0.23*
C10–C11	1.380(2)	2.120	-20.343	0.22
		2.089	*-18.315*	*0.20*
C11–C12	1.381(2)	2.119	-20.317	0.22
		2.088	*-18.315*	*0.21*
C12–C13	1.381(2)	2.236	-21.822	0.18
		2.094	*-18.315*	*0.23*
C11–C15	1.431(2)	1.913	-13.487	0.15
		1.865	*-16.628*	*0.04*
C15–N4	1.151(2)	3.435	-18.161	0.00
		3.139	*-2.169*	*0.03*
C9–H9	1.08(13)	1.807	-19.655	0.06
		1.904	*-22.412*	*0.02*
C10–H10	1.08(18)	1.771	-18.939	0.05
		1.905	*-22.171*	*0.01*
C12–H12	1.08(14)	1.771	-18.937	0.05
		1.871	*-21.207*	*0.01*
C13–H13	1.08(17)	1.806	-19.649	0.06
		1.919	*-22.894*	*0.02*
C14–H14A	1.08(13)	1.686	-16.479	0.09
		1.910	*-21.207*	*0.05*
C14–H14B	1.08(8)	1.686	-16.479	0.09
		1.866	*-20.243*	*0.05*
C14–H14C	1.08(15)	1.686	-16.479	0.09
		1.890	*-21.207*	*0.05*

Table S28 Topology of electron density of the 4-cyano-*N*-methylpyridinium cation, derived from electron-density after multipole refinement at 340 K and after periodic calculations (given in italic).

Bond	Length (Å)	Electron Density ($eÅ^{-3}$) ρ_{cp}	Laplacian ($eÅ^{-5}$)	Ellipticity
N3–C9	1.338(2)	2.275	-22.599	0.07
		2.159	*-14.459*	*0.05*
N3–C13	1.333(4)	2.287	-22.681	0.07
		2.183	*-14.700*	*0.05*
N3–C14	1.473(5)	1.707	-9.688	0.05
		1.609	*-10.844*	*0.03*
C9–C10	1.386(4)	2.221	-21.434	0.18
		2.077	*-17.833*	*0.23*
C10–C11	1.389(4)	2.096	-19.744	0.22
		2.056	*-17.833*	*0.20*
C11–C12	1.366(2)	2.161	-21.465	0.22
		2.146	*-19.279*	*0.22*
C12–C13	1.391(5)	2.206	-21.019	0.18
		2.057	*-17.592*	*0.23*
C11–C15	1.435(4)	1.903	-13.255	0.15
		1.854	*-16.387*	*0.04*
C15–N4	1.149(4)	3.442	-17.640	0.00
		3.147	*-1.687*	*0.03*
C9–H9	1.08(6)	1.806	-19.649	0.06
		1.902	*-22.412*	*0.02*
C10–H10	1.08(7)	1.770	-18.934	0.05
		1.865	*-21.207*	*0.01*
C12–H12	1.08(6)	1.770	-18.937	0.05
		1.844	*-20.484*	*0.01*
C13–H13	1.08(7)	1.806	-19.645	0.06
		1.881	*-22.171*	*0.02*
C14–H14A	1.08(7)	1.685	-16.477	0.09
		1.912	*-21.448*	*0.05*
C14–H14B	1.08(4)	1.686	-16.479	0.09
		1.910	*-21.689*	*0.04*
C14–H14C	1.08(6)	1.686	-16.481	0.09
		1.853	*-20.002*	*0.05*

Table S29 Topology of electron density of the 4-cyano-*N*-methylpyridinium cation, derived from electron-density after multipole refinement at 370 K and after periodic calculations (given in italic).

Bond	Length (Å)	Electron Density ($eÅ^{-3}$) ρ_{cp}	Laplacian ($eÅ^{-5}$)	Ellipticity
N3–C9	1.345(3)	2.253	-22.280	0.08
		2.130	*-14.941*	*0.05*
N3–C13	1.340(3)	2.266	-22.529	0.08
		2.166	*-14.941*	*0.05*
N3–C14	1.485(4)	1.674	-8.942	0.05
		1.587	*-10.844*	*0.03*
C9–C10	1.385(4)	2.223	-21.500	0.18
		2.097	*-18.315*	*0.23*
C10–C11	1.393(3)	2.112	-20.170	0.22
		2.092	*-18.556*	*0.20*
C11–C12	1.378(3)	2.125	-20.502	0.22
		2.099	*-18.556*	*0.21*
C12–C13	1.393(4)	2.198	-20.859	0.18
		2.067	*-17.833*	*0.23*
C11–C15	1.433(3)	1.907	-13.362	0.15
		1.877	*-16.869*	*0.04*
C15–N4	1.157(3)	3.408	-19.945	0.00
		3.131	*-2.410*	*0.03*
C9–H9	1.08(19)	1.806	-19.651	0.06
		1.926	*-23.135*	*0.02*
C10–H10	1.1(3)	1.770	-18.936	0.05
		1.877	*-21.448*	*0.01*
C12–H12	1.1(2)	1.770	-19.933	0.05
		1.890	*-21.448*	*0.01*
C13–H13	1.1(3)	1.806	-19.648	0.06
		1.896	*-22.412*	*0.02*
C14–H14A	1.1(2)	1.686	-16.484	0.09
		1.921	*-21.689*	*0.05*
C14–H14B	1.1(2)	1.686	-16.484	0.09
		1.884	*-20.966*	*0.05*
C14–H14C	1.08(13)	1.686	-16.478	0.09
		1.874	*-20.484*	*0.05*

S9 Intermolecular critical points at variable temperatures

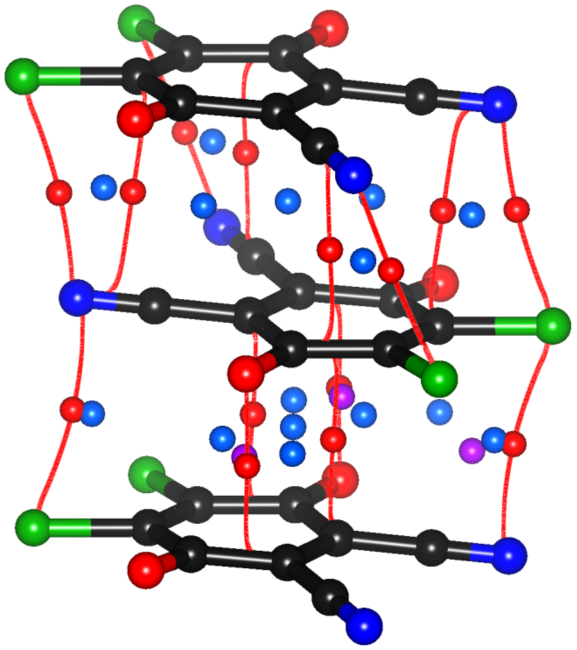

90 K

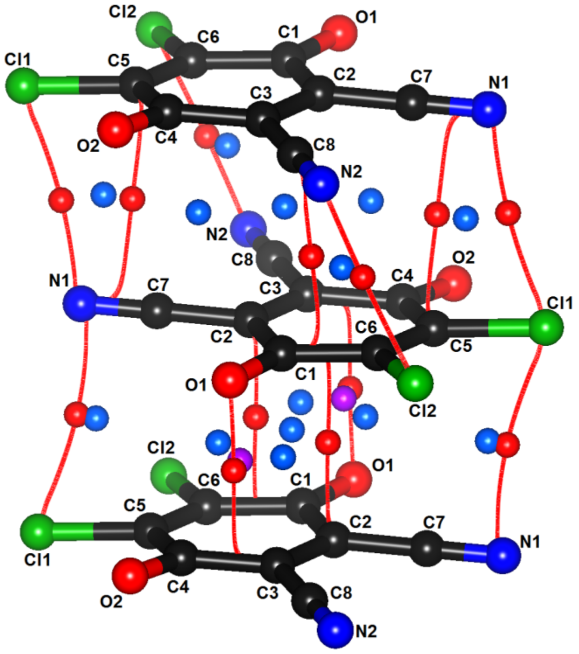

120 K

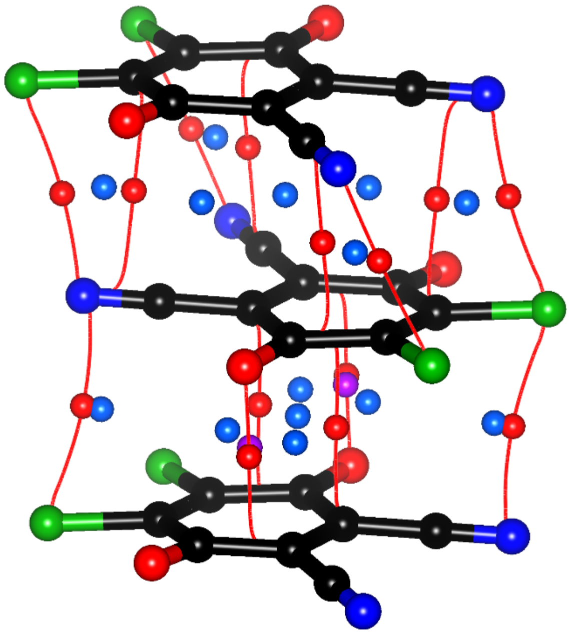

150K

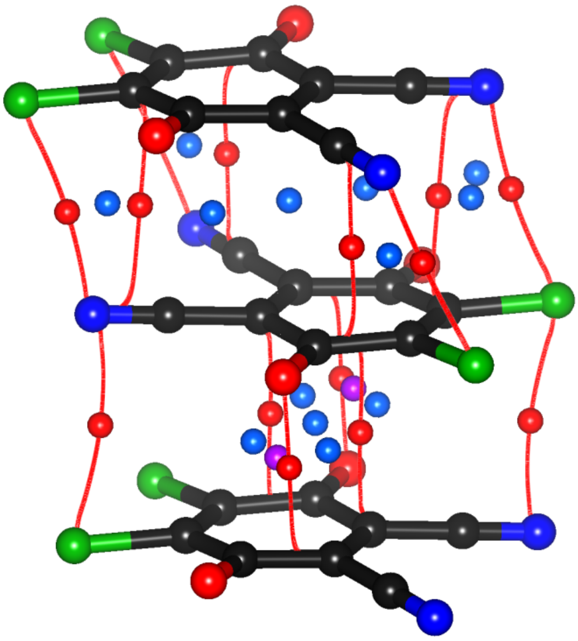

180 K

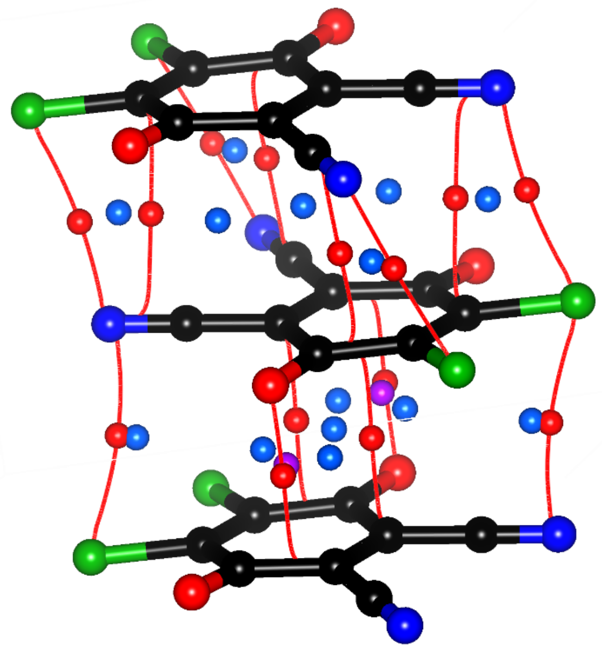

210 K

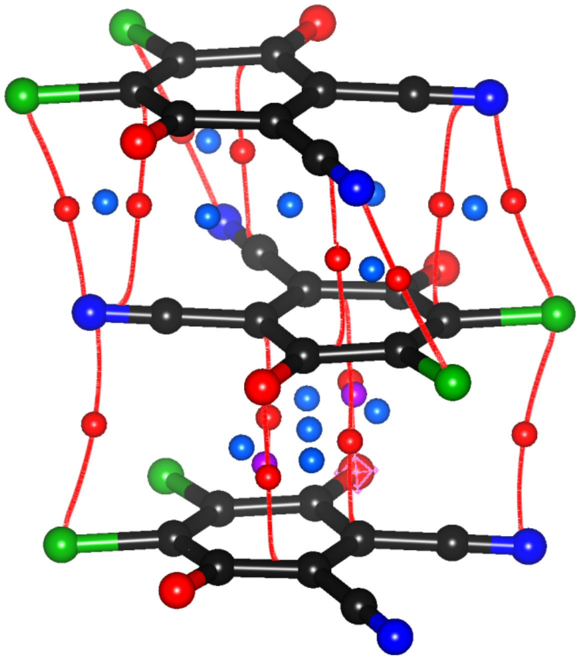

240 K

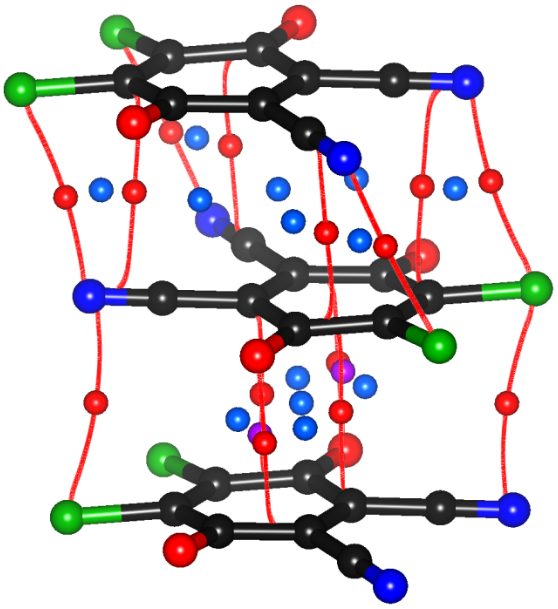

270 K

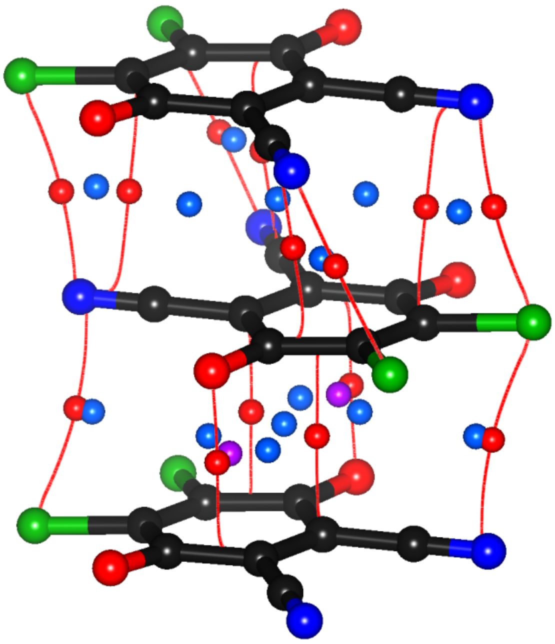

293 K

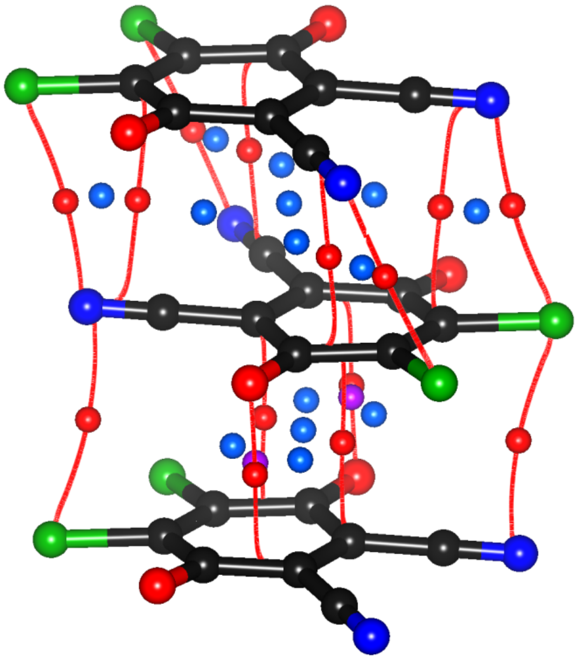

310 K

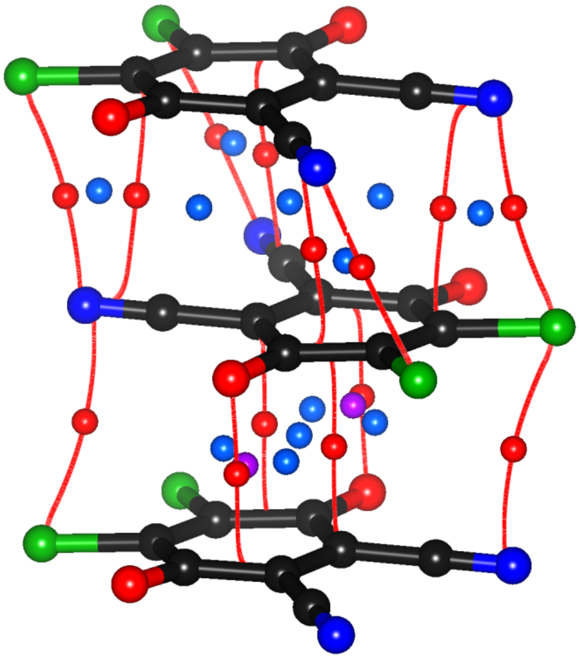

340 K

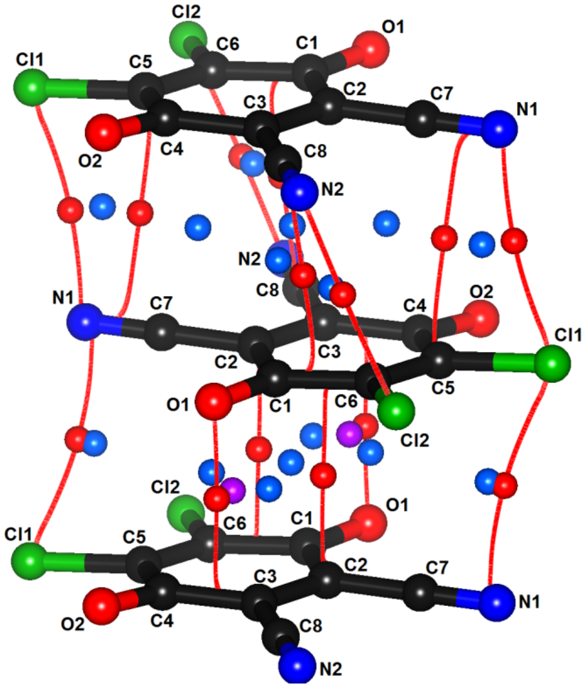

370 K

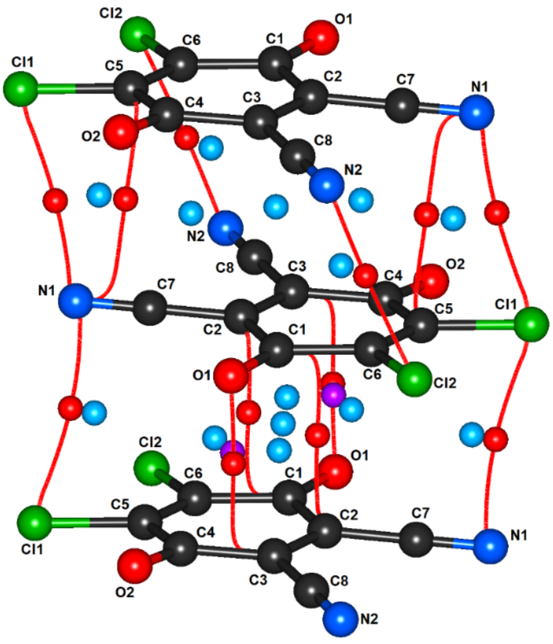

100 K (high-resolution data from [2])

Figure S47 Critical points in a stack of DDQ radical anions at variable temperatures. Weaker inter-dimer contact is above and intra-dimer contact (multicentric bond) is below. (3,-1) critical points are shown as red spheres, (3,+1) as blue spheres and (3,+3) as purple spheres; intermolecular bond paths are shown as red lines.

Table S30 Total electron density ρ_{tot} and Laplacian $\nabla^2\rho$ at the critical points between the DDQ$^-$ radical anions at 90 K. Experimentally determined cp's are printed regular and theoretical ones from periodic calculations are italic. Symmetry operation on B: (*i*) $-x, -y+1, -z$; (*ii*) $-x+1, -y+1, -z$.

A…B	*d* (Å)	ρ_{tot} (e Å^{-3})	Laplacian (e Å^{-5})	CP type	Symm.
close (intra dimer)					
C1···C2	2.9761	0.085	0.920	(3,-1)	(*i*)
		0.080	*0.964*		
O1···C3	2.9282	0.082	1.062	(3,-1)	(*i*)
		0.084	*0.990*		
N1···Cl1	3.5166	0.034	0.437	(3,-1)	(*i*)
		0.027	*0.482*		
O1···O1	4.3618	0.077	0.884	(3,+1)	(*i*)
O1···C2	3.2361	0.072	0.870	(3,+1)	(*i*)
O1···Cl2	4.7852	0.046	0.589	(3,+1)	(*i*)
N1···C5	3.6117	0.034	0.424	(3,+1)	(*i*)
N1···O2	4.9759	0.022	0.318	(3,+1)	(*i*)
C3···C2	4.1024	0.043	0.612	(3,+3)	(*i*)
Cl1···C1	4.7406	0.011	0.134	(3,+3)	(*i*)
Cl1···C7	3.8008	0.034	0.424	(3,+3)	(*i*)
O1···Cl1	4.2841	0.011	0.134	(3,+3)	(*i*)
long (inter dimer)					
Cl1···N1	3.4127	0.039	0.516	(3,-1)	(*ii*)
		0.036	*0.482*		
N1···C5	3.4400	0.037	0.430	(3,-1)	(*ii*)
		0.036	*0.584*		
Cl2···N2	3.4428	0.037	0.482	(3,-1)	(*ii*)
		0.033	*0.482*		
C8···C1	3.4020	0.031	0.370	(3,-1)	(*ii*)
		0.021	*0.482*		
N1···C5	3.4400	0.036	0.443	(3,+1)	(*ii*)
C1···C1	4.8602	0.035	0.377	(3,+1)	(*ii*)
Cl2···C8	3.9144	0.030	0.369	(3,+1)	(*ii*)
N1···C3	4.3226	0.029	0.332	(3,+1)	(*ii*)
N2···C6	3.6045	0.030	0.369	(3,+1)	(*ii*)
O2···C2	4.2702	0.029	0.332	(3,+1)	(*ii*)

Table S31 Total electron density ρ_{tot} and Laplacian $\nabla^2\rho$ at the critical points between the DDQ$^-$ radical anions at 100 K. Experimentally determined cp's are printed regular and theoretical ones from periodic calculations are italic. Symmetry operation on B: (*i*) –*x*, –*y*+1, –*z;* (*ii*) –*x*+1, –*y*+1, –*z*. Data are from our previous high-resolution work [2], given for comparison.

A…B	*d*	ρ_{tot} / e Å^{-3}	Laplacian / e Å^{-5}	*G*cp / e Å^{-3}	*V*cp / e Å^{-3}	|*V*cp| /*G*cp	CP type	Symm.
close (intra dimer)								
C2···C1	2.983	0.085	0.913	0.056	-0.048	0.860	(3,-1)	(*i*)
		0.079	*1.012*	*0.050*	*-0.029*	*0.578*		
O1···C3	2.925	0.084	1.074	0.063	-0.051	0.806	(3,-1)	(*i*)
		0.085	*0.990*	*0.056*	*-0.042*	*0.757*		
N1···Cl1	3.521	0.035	0.434	0.022	-0.016	0.712	(3,-1)	(*i*)
		0.027	*0.482*	*0.060*	*-0.052*	*0.866*		
O1···O1		0.079	0.899				(3,+1)	(*i*)
		0.076	*0.933*					
O1···C2		0.074	0.875				(3,+1)	(*i*)
		0.059	*1.047*					
O1···Cl2		0.048	0.597				(3,+1)	(*i*)
		0.042	*0.617*					
Cl1···C2		0.034	0.419				(3,+1)	(*i*)
		0.022	*0.462*					
N1···C5		0.034	0.419				(3,+1)	(*i*)
		0.025	*0.462*					
N1···O2		0.025	0.327				(3,+1)	(*i*)
		0.022	*0.462*					
N1···Cl2		0.016	0.224				(3,+1)	(*i*)
		0.011	*0.180*					
C3···C2		0.046	0.610				(3,+3)	(*i*)
		0.031	*0.679*					
C6···C5		0.046	0.610				(3,+3)	(*i*)
		0.031	*0.679*					
long (inter dimer)								
Cl1···N1	3.411	0.039	0.518	0.028	-0.019	0.691	(3,-1)	(*ii*)
		0.027	*0.482*	*0.060*	*-0.052*	*0.866*		
C5···N1	3.434	0.037	0.435	0.023	-0.016	0.702	(3,-1)	(*ii*)
		0.025	*0.462*	*0.023*	*-0.014*	*0.592*		
Cl2···N2	3.442	0.037	0.483	0.027	-0.018	0.744	(3,-1)	(*ii*)
		0.033	*0.482*	*0.030*	*-0.021*	*0.693*		
C1···C1		0.037	0.390				(3,+1)	(*ii*)
		0.024	*0.400*					
N1···C5		0.036	0.446				(3,+1)	(*ii*)
		0.022	*0.462*					
Cl1···C7		0.036	0.446				(3,+1)	(*ii*)
		0.025	*0.462*					
Cl2···C8		0.030	0.371				(3,+1)	(*ii*)
		0.033	*0.552*					
N1···C3		0.030	0.343				(3,+1)	(*ii*)
		0.025	*0.462*					
N2···C6		0.030	0.371				(3,+1)	(*ii*)
		0.019	*0.375*					
C3···N1		0.030	0.343				(3,+1)	(*ii*)
		0.025	*0.462*					

Table S32 Total electron density ρ_{tot} and Laplacian $\nabla^2\rho$ at the critical points between the DDQ$^-$ radical anions at 120 K. Experimentally determined cp's are printed regular and theoretical ones from periodic calculations are italic. Symmetry operation on B: (*i*) –*x*, –*y*+1, –*z;* (*ii*) –*x*+1, –*y*+1, –*z*

A…B	d (Å)	ρ_{tot} (e Å^{-3})	Laplacian (e Å^{-5})	CP type	Symm.
close (intra	dimer)				
C1···C2	2.9884	0.084	0.905	(3,-1)	(*i*)
		0.078	*0.964*		
O1···C3	2.9316	0.081	1.053	(3,-1)	(*i*)
		0.084	*0.981*		
N1···Cl1	3.5257	0.034	0.430	(3,-1)	(*i*)
		0.026	*0.482*		
O1···O1	4.3611	0.075	0.870	(3,+1)	(*i*)
O1···C2	3.2406	0.071	0.856	(3,+1)	(*i*)
O1···Cl2	4.7836	0.046	0.587	(3,+1)	(*i*)
N1···C5	3.6275	0.033	0.416	(3,+1)	(*i*)
N1···O2	4.9959	0.017	0.232	(3,+1)	(*i*)
C3···C2	4.1112	0.043	0.607	(3,+3)	(*i*)
O1···Cl1	4.2806	0.010	0.133	(3,+3)	(*i*)
O1···Cl1	4.2806	0.046	0.587	(3,+1)	(*i*)
Cl1···C2	4.5466	0.033	0.416	(3,+1)	(*i*)
N2···Cl2	4.0406	0.010	0.133	(3,+3)	(*i*)
C6···C5	4.5353	0.043	0.607	(3,+3)	(*i*)
long (inter	dimer)				
Cl1···N1	3.4171	0.039	0.512	(3,-1)	(*ii*)
		0.035	*0.579*		
N1···C5	3.4399	0.037	0.431	(3,-1)	(*ii*)
		0.035	*0.579*		
Cl2···N2	3.4456	0.037	0.480	(3,-1)	(*ii*)
		0.033	*0.482*		
C1···C8	3.3964	0.032	0.372	(3,-1)	(*ii*)
		0.021	*0.482*		
N1···C5	3.4399	0.036	0.443	(3,+1)	(*ii*)
C1···C1	4.8626	0.035	0.377	(3,+1)	(*ii*)
Cl2···C8	3.9177	0.030	0.370	(3,+1)	(*ii*)
N1···C3	4.3203	0.029	0.333	(3,+1)	(*ii*)
Cl1···C7	3.9612	0.036	0.443	(3,+1)	(*ii*)
N2···O1	3.6718	0.030	0.370	(3,+1)	(*ii*)

Table S33 Total electron density ρ_{tot} and Laplacian $\nabla^2\rho$ at the critical points between the DDQ$^-$ radical anions at 150 K. Experimentally determined cp's are printed regular and theoretical ones from periodic calculations are italic. Symmetry operation on B: (*i*) $-x, -y+1, -z$; (*ii*) $-x+1, -y+1, -z$.

A…B	*d* (Å)	ρ_{tot} (e Å^{-3})	Laplacian (e Å^{-5})	CP type	Symm.
close (intra	dimer)				
C1···C2	2.9956	0.083	0.894	(3,-1)	(*i*)
		0.077	0.988		
O1···C3	2.9373	0.080	1.040	(3,-1)	(*i*)
		0.083	0.968		
N1···Cl1	3.5362	0.033	0.421	(3,-1)	(*i*)
		0.026	0.474		
C1···C8	3.4041	0.031	0.367	(3,-1)	(*i*)
O1···O1	4.3641	0.075	0.860	(3,+1)	(*i*)
O1···C2	3.2451	0.070	0.847	(3,+1)	(*i*)
O1···Cl2	4.7863	0.046	0.581	(3,+1)	(*i*)
N1···C5	3.6391	0.033	0.409	(3,+1)	(*i*)
Cl1···C2	4.5529	0.033	0.409	(3,+1)	(*i*)
O1···Cl1	4.2845	0.046	0.581	(3,+1)	(*i*)
C3···C2	4.1167	0.043	0.601	(3,+3)	(*i*)
C3···C7	4.6437	0.043	0.601	(3,+3)	(*i*)
long (inter	dimer)				
Cl1···N1	3.4269	0.038	0.502	(3,-1)	(*ii*)
		0.034	*0.568*		
N1···C5	3.4460	0.036	0.424	(3,-1)	(*ii*)
		0.034	*0.568*		
Cl2···N2	3.4566	0.036	0.469	(3,-1)	(*ii*)
		0.032	*0.537*		
C8···C1	3.4041	0.031	0.367	(3,-1)	(*ii*)
		0.021	*0.371*		
N1···C5	3.4460	0.035	0.437	(3,+1)	(*ii*)
Cl1···C7	3.9759	0.035	0.437	(3,+1)	(*ii*)
C1···C1	4.8750	0.035	0.372	(3,+1)	(*ii*)
Cl2···C8	3.9312	0.030	0.365	(3,+1)	(*ii*)
N2···O1	3.6773	0.030	0.365	(3,+1)	(*ii*)
O2···O2	4.2722	0.029	0.329	(3,+1)	(*ii*)
C5···C13	4.4666	0.024	0.320	(3,+1)	(*ii*)
C5···C12	4.6393	0.019	0.313	(3,+3)	(*ii*)

Table S34 Total electron density ρ_{tot} and Laplacian $\nabla^2\rho$ at the critical points between the DDQ$^-$ radical anions at 180 K. Experimentally determined cp's are printed regular and theoretical ones from periodic calculations are italic. Symmetry operation on B: (*i*) –*x*, –*y*+1, –*z;* (*ii*) –*x*+1, –*y*+1, –*z*.

A…B	*d* (Å)	ρ_{tot} (e Å^{-3})	Laplacian (e Å^{-5})	CP type	Symm.
close (intra	dimer)				
C1···C2	3.0021	0.082	0.884	(3,-1)	(*i*)
		0.076	*0.964*		
O1···C3	2.9407	0.079	1.033	(3,-1)	(*i*)
		0.082	*0.960*		
N1···Cl1	3.5410	0.033	0.417	(3,-1)	(*i*)
		0.026	*0.482*		
O1···O1	4.3632	0.074	0.852	(3,+1)	(*i*)
O1···C2	3.2487	0.070	0.839	(3,+1)	(*i*)
O1···Cl2	4.7856	0.046	0.578	(3,+1)	(*i*)
O1···Cl1	4.2831	0.010	0.129	(3,+3)	(*i*)
O1···Cl1	4.2831	0.046	0.578	(3,+1)	(*i*)
C6···C5	4.5390	0.043	0.597	(3,+3)	(*i*)
Cl1···C7	3.8176	0.033	0.406	(3,+1)	(*i*)
C1···Cl1	4.7421	0.010	0.129	(3,+3)	(*i*)
long (inter	dimer)				
Cl1···N1	3.4381	0.037	0.491	(3,-1)	(*ii*)
		0.034	*0.482*		
N1···C5	3.4534	0.036	0.418	(3,-1)	(*ii*)
		0.034	*0.557*		
Cl2···N2	3.4646	0.035	0.462	(3,-1)	(*ii*)
		0.032	*0.482*		
C1···C8	3.4099	0.031	0.363	(3,-1)	(*ii*)
		0.020	*0.482*		
N1···C5	3.4534	0.035	0.431	(3,+1)	(*ii*)
C1···C1	4.8839	0.035	0.369	(3,+1)	(*ii*)
Cl2···C8	3.9424	0.029	0.362	(3,+1)	(*ii*)
N1···C3	4.3176	0.029	0.327	(3,+1)	(*ii*)
Cl1···C7	3.9863	0.035	0.431	(3,+1)	(*ii*)
O2···C2	4.2750	0.029	0.327	(3,+1)	(*ii*)
O2···C7	3.7889	0.015	0.208	(3,+1)	(*ii*)
N1···C6	4.1090	0.033	0.406	(3,+1)	(*ii*)
N2···C6	3.6106	0.029	0.362	(3,+1)	(*ii*)

Table S35 Total electron density ρ_{tot} and Laplacian $\nabla^2\rho$ at the critical points between the DDQ$^-$ radical anions at 210 K. Experimentally determined cp's are printed regular and theoretical ones from periodic calculations are italic. Symmetry operation on B: (*i*) –*x*, –*y*+1, –*z;* (*ii*) –*x*+1, –*y*+1, –*z*.

A…B	Length (Å)	ρ_{tot}	Laplacian	CP type	Symm.
close (intra	dimer)				
C1···C2	3.0086	0.081	0.873	(3,-1)	(*i*)
		0.075	*0.963*		
O1···C3	2.9502	0.078	1.016	(3,-1)	(*i*)
		0.081	*0.944*		
N1···Cl1	3.5467	0.032	0.412	(3,-1)	(*i*)
		0.025	*0.466*		
O1···O1	4.3662	0.073	0.844	(3,+1)	(*i*)
O1···C2	3.2558	0.069	0.828	(3,+1)	(*i*)
O1···Cl2	4.7887	0.045	0.571	(3,+1)	(*i*)
N1···C5	3.6512	0.032	0.401	(3,+1)	(*i*)
Cl1···C7	3.8236	0.032	0.401	(3,+1)	(*i*)
O1···Cl1	4.2864	0.045	0.571	(3,+1)	(*i*)
C3···C2	4.1294	0.042	0.590	(3,+3)	(*i*)
C3···C7	4.6577	0.042	0.590	(3,+3)	(*i*)
long (inter	dimer)				
Cl1···N1	3.4499	0.036	0.480	(3,-1)	(*ii*)
		0.032	*0.545*		
N1···C5	3.4610	0.035	0.412	(3,-1)	(*ii*)
		0.032	*0.545*		
Cl2···N2	3.4708	0.035	0.456	(3,-1)	(*ii*)
		0.031	*0.523*		
C8···C1	3.4161	0.031	0.360	(3,-1)	(*ii*)
		0.020	*0.362*		
N1···C2	4.8786	0.028	0.323	(3,+1)	(*ii*)
N1···C5	3.4610	0.034	0.424	(3,+1)	(*ii*)
Cl1···C7	3.9973	0.034	0.424	(3,+1)	(*ii*)
C1···C1	4.8962	0.034	0.365	(3,+1)	(*ii*)
Cl2···C8	3.9524	0.029	0.359	(3,+1)	(*ii*)
N2···C1	3.6761	0.029	0.359	(3,+1)	(*ii*)
C2···C7	4.1746	0.028	0.323	(3,+1)	(*ii*)

Table S36 Total electron density ρ_{tot} and Laplacian $\nabla^2\rho$ at the critical points between the DDQ$^-$ radical anions at 240 K. Experimentally determined cp's are printed regular and theoretical ones from periodic calculations are italic. Symmetry operation on B: (*i*) –*x*, –*y*+1, –*z;* (*ii*) –*x*+1, –*y*+1, –*z*.

A…B	*d* (Å)	ρ_{tot} (e Å^{-3})	Laplacian (e Å^{-5})	CP type	Symm.
close (intra	dimer)				
C1···C2	3.0203	0.079	0.856	(3,-1)	(*i*)
		0.073	*0.964*		
O1···C3	2.9635	0.076	0.987	(3,-1)	(*i*)
		0.078	*0.917*		
N1···Cl1	3.5623	0.032	0.401	(3,-1)	(*i*)
		0.024	*0.482*		
O1···O1	4.3728	0.072	0.828	(3,+1)	(*i*)
O1···C2	3.2666	0.067	0.810	(3,+1)	(*i*)
O1···Cl2	4.7920	0.045	0.562	(3,+1)	(*i*)
O1···Cl1	4.2917	0.010	0.124	(3,+3)	(*i*)
C6···C5	4.5533	0.042	0.580	(3,+3)	(*i*)
Cl1···C7	3.8322	0.031	0.391	(3,+1)	(*i*)
Cl1···N3	4.9262	0.016	0.196	(3,+1)	(*i*)
N1···C5	3.6675	0.031	0.391	(3,+1)	(*i*)
long (inter	dimer)				
Cl1···N1	3.4669	0.035	0.464	(3,-1)	(*ii*)
		0.031	*0.482*		
N1···C5	3.4756	0.034	0.400	(3,-1)	(*ii*)
		0.031	*0.528*		
Cl2···N2	3.4893	0.034	0.440	(3,-1)	(*ii*)
		0.030	*0.482*		
C1···C8	3.4310	0.030	0.350	(3,-1)	(*ii*)
		0.020	*0.241*		
N1···C5	3.4756	0.034	0.400	(3,+1)	(*ii*)
C1···C1	4.9198	0.033	0.356	(3,+1)	(*ii*)
Cl2···C8	3.9736	0.028	0.350	(3,+1)	(*ii*)
N1···C3	4.3257	0.028	0.315	(3,+1)	(*ii*)
Cl1···C7	4.0184	0.033	0.411	(3,+1)	(*ii*)
O2···C2	4.2893	0.028	0.315	(3,+1)	(*ii*)
O2···C7	3.8026	0.014	0.202	(3,+1)	(*ii*)
O2···N1	3.7627	0.014	0.203	(3,+3)	(*ii*)
Cl1···O2	4.2592	0.019	0.294	(3,+1)	(*ii*)

N2···C1	3.6840	0.028	0.349	(3,+1)	(*ii*)

Table S37 Total electron density ρ_{tot} and Laplacian $\nabla^2\rho$ at the critical points between the DDQ^{-} radical anions at 270 K. Experimentally determined cp's are printed regular and theoretical ones from periodic calculations are italic. Symmetry operation on B: (*i*) –*x*, –*y*+1, –*z;* (*ii*) –*x*+1, –*y*+1, –*z*.

A…B	*d* (Å)	ρ_{tot} (e Å^{-3})	Laplacian (e Å^{-5})	CP type	Symm.
close (intra	dimer)				
C1···C2	3.023	0.079	0.851	(3,-1)	(*i*)
		0.073	*0.964*		
O1···C3	2.967	0.075	0.980	(3,-1)	(*i*)
		0.078	*0.910*		
Cl1···N1	3.563	0.032	0.401	(3,-1)	(*i*)
		0.024	*0.482*		
O1···C2		0.067	0.805	(3,+1)	(*i*)
O1···O1		0.071	0.822	(3,+1)	(*i*)
O1···Cl2		0.044	0.559	(3,+1)	(*i*)
Cl1···C7		0.031	0.391	(3,+1)	(*i*)
N1···C5		0.031	0.391	(3,+1)	(*i*)
C6···C5		0.041	0.575	(3,+3)	(*i*)
C2···C3		0.042	0.576	(3,+3)	(*i*)
long (inter	dimer)				
Cl1···N1	3.474	0.035	0.458	(3,-1)	(*ii*)
		0.030	*0.482*		
C1···C8	3.433	0.030	0.349	(3,-1)	(*ii*)
		0.031	*0.521*		
Cl2···N2	3.495	0.033	0.435	(3,-1)	(*ii*)
		0.029	*0.482*		
N1···C5	3.477	0.034	0.398	(3,-1)	(*ii*)
		0.019	*0.241*		
C1···C1		0.033	0.356	(3,+1)	(*ii*)
C1···C2		0.028	0.324	(3,+1)	(*ii*)
C2···C1		0.016	0.259	(3,+1)	(*ii*)
O2···C2		0.028	0.315	(3,+1)	(*ii*)
O2···N1		0.014	0.201	(3,+1)	(*ii*)
N1···C5		0.033	0.409	(3,+1)	(*ii*)
Cl1···C7		0.033	0.409	(3,+1)	(*ii*)
Cl2···C8		0.028	0.348	(3,+1)	(*ii*)
N1···C3		0.028	0.315	(3,+1)	(*ii*)
N2···C1		0.028	0.348	(3,+1)	(*ii*)

Table S38 Total electron density ρ_{tot} and Laplacian $\nabla^2\rho$ at the critical points between the DDQ$^-$ radical anions at 293 K. Experimentally determined cp's are printed regular and theoretical theoretical ones from periodic calculations are italic. Symmetry operation on B: (*i*) –*x*, –*y*+1, –*z;* (*ii*) –*x*+1, –*y*+1, –*z*.

A…B	*d* (Å)	ρ_{tot} (e Å^{-3})	Laplacian (e Å^{-5})	CP type	Symm.
close (intra	dimer)				
C6···C2	3.046	0.078	0.838	(3,-1)	(*i*)
		0.072	*0.964*		
O1···C3	2.977	0.074	0.963	(3,-1)	(*i*)
		0.076	*0.895*		
N1···Cl1	3.570	0.031	0.395	(3,-1)	(*i*)
		0.024	*0.482*		
Cl1···O1		0.044	0.552	(3,+1)	(*i*)
O1···C2		0.065	0.791	(3,+1)	(*i*)
O1···O1		0.070	0.805	(3,+1)	(*i*)
O1···Cl2		0.044	0.552	(3,+1)	(*i*)
Cl1···C2		0.031	0.386	(3,+1)	(*i*)
N1···C5		0.031	0.386	(3,+1)	(*i*)
N1···C1		0.041	0.567	(3,+3)	(*i*)
long (inter	dimer)				
Cl1···N1	3.485	0.034	0.448	(3,-1)	(*ii*)
		0.030	*0.510*		
C1···C8	3.434	0.029	0.347	(3,-1)	(*ii*)
		0.019	*0.241*		
C5···N1	3.484	0.034	0.394	(3,-1)	(*ii*)
		0.030	*0.510*		
Cl2···N2	3.505	0.032	0.427	(3,-1)	(*ii*)
		0.028	*0.482*		
C1···C1		0.033	0.352	(3,+1)	(*ii*)
O2···C2		0.028	0.312	(3,+1)	(*ii*)
O2···N1		0.014	0.199	(3,+1)	(*ii*)
N1···C5		0.032	0.404	(3,+1)	(*ii*)
Cl1···C7		0.032	0.404	(3,+1)	(*ii*)
Cl2···C8		0.028	0.345	(3,+1)	(*ii*)
N1···C1		0.028	0.344	(3,+1)	(*ii*)
N1···C3		0.028	0.312	(3,+1)	(*ii*)

Table S39 Total electron density ρ_{tot} and Laplacian $\nabla^2\rho$ at the critical points between the DDQ^{-} radical anions at 310 K. Experimentally determined cp's are printed regular and theoretical theoretical ones from periodic calculations are italic. Symmetry operation on B: (*i*) –*x*, –*y*+1, –*z;* (*ii*) –*x*+1, –*y*+1, –*z*.

A…B	*d* (Å)	ρ_{tot} (e Å^{-3})	Laplacian (e Å^{-5})	CP type	Symm.
close (intra	dimer)				
C1···C2	3.034	0.078	0.838	(3,-1)	(*i*)
		0.072	*0.964*		
O1···C3	2.981	0.073	0.955	(3,-1)	(*i*)
		0.075	*0.886*		
Cl1···N1	3.572	0.031	0.394	(3,-1)	(*i*)
		0.023	*0.443*		
O1···C2		0.065	0.790	(3,+1)	(*i*)
O1···O1		0.070	0.808	(3,+1)	(*i*)
O1···Cl2		0.044	0.552	(3,+1)	(*i*)
Cl1···C2		0.031	0.385	(3,+1)	(*i*)
Cl1···C7		0.029	0.362	(3,+1)	(*i*)
N1···C5		0.031	0.385	(3,+1)	(*i*)
C6···C5		0.041	0.568	(3,+3)	(*i*)
C1···C4		0.041	0.568	(3,+3)	(*i*)
Cl2···N1		0.040	0.544	(3,+3)	(*i*)
long (inter	dimer)				
Cl1···N1	3.486	0.034	0.448	(3,-1)	(*ii*)
		0.030	*0.482*		
C1···C8	3.437	0.030	0.347	(3,-1)	(*ii*)
		0.019	*0.241*		
C5···N1	3.485	0.034	0.394	(3,-1)	(*ii*)
		0.030	*0.509*		
Cl2···N2	3.505	0.032	0.427	(3,-1)	(*ii*)
		0.028	*0.482*		
C1···C1		0.033	0.353	(3,+1)	(*ii*)
O2···C2		0.028	0.321	(3,+1)	(*ii*)
O2···N1		0.014	0.200	(3,+1)	(*ii*)
N1···C5		0.032	0.404	(3,+1)	(*ii*)
Cl1···C7		0.034	0.404	(3,+1)	(*ii*)
Cl2···C8		0.028	0.346	(3,+1)	(*ii*)
C3···C8		0.016	0.251	(3,+1)	(*ii*)

C6···C3	0.014	0.200	(3,+1)	(*ii*)
N1···C3	0.028	0.314	(3,+1)	(*ii*)
N2···C6	0.028	0.346	(3,+1)	(*ii*)

Table S40 Total electron density ρ_{tot} and Laplacian $\nabla^2\rho$ at the critical points between the DDQ^{-} radical anions at 340 K. Experimentally determined cp's are printed regular and theoretical theoretical ones from periodic calculations are italic. Symmetry operation on B: (*i*) –*x*, –*y*+1, –*z;* (*ii*) –*x*+1, –*y*+1, –*z*.

A…B	*d* (Å)	ρ_{tot} (e Å^{-3})	Laplacian (e Å^{-5})	CP type	Symm.
close (intra	dimer)				
C1···C2	3.065	0.073	0.793	(3,-1)	(*i*)
		0.066	*0.964*		
O1···C3	3.011	0.069	0.902	(3,-1)	(*i*)
		0.071	*0.834*		
N1···Cl1	3.606	0.029	0.369	(3,-1)	(*i*)
		0.021	*0.416*		
O1···C2		0.062	0.751	(3,+1)	(*i*)
O1···O1		0.067	0.767	(3,+1)	(*i*)
O1···Cl2		0.042	0.531	(3,+1)	(*i*)
Cl1···C7		0.029	0.362	(3,+1)	(*i*)
N1···C5		0.029	0.362	(3,+1)	(*i*)
C6···C5		0.040	0.544	(3,+3)	(*i*)
Cl2···N1		0.040	0.544	(3,+3)	(*i*)
long (inter	dimer)				
Cl1···N1	3.514	0.032	0.423	(3,-1)	(*ii*)
		0.028	*0.482*		
C1···C8	3.455	0.029	0.336	(3,-1)	(*ii*)
		0.018	*0.241*		
Cl2···N2	3.542	0.030	0.396	(3,-1)	(*ii*)
		0.026	*0.482*		
N1···C5	3.505	0.032	0.377	(3,-1)	(*ii*)
		0.021	*0.482*		
C1···C1		0.032	0.344	(3,+1)	(*ii*)
O2···C2		0.027	0.302	(3,+1)	(*ii*)
O2···N1		0.014	0.191	(3,+1)	(*ii*)
N1···C5		0.031	0.386	(3,+1)	(*ii*)
Cl1···C7		0.031	0.386	(3,+1)	(*ii*)
Cl2···C8		0.027	0.332	(3,+1)	(*ii*)
N1···C3		0.027	0.302	(3,+1)	(*ii*)
N2···C1		0.027	0.332	(3,+1)	(*ii*)

Table S41 Total electron density ρ_{tot} and Laplacian $\nabla^2\rho$ at the critical points between the DDQ^{-} radical anions at 370 K. Experimentally determined cp's are printed regular and theoretical theoretical ones from periodic calculations are italic. Symmetry operation on B: (*i*) –*x*, –*y*+1, –*z;* (*ii*) –*x*+1, –*y*+1, –*z*.

A…B	*d* (Å)	ρ_{tot} (e Å^{-3})	Laplacian (e Å^{-5})	CP type	Symm.
close (intra	dimer)				
C6···C2	3.095	0.071	0.768	(3,-1)	(*i*)
		0.066	*0.964*		
O1···C3	3.038	0.066	0.859	(3,-1)	(*i*)
		0.070	*0.821*		
N1···Cl1	3.612	0.029	0.364	(3,-1)	(*i*)
		0.028	*0.482*		
Cl1···O1		0.041	0.514	(3,+1)	(*i*)
O1···C2		0.059	0.718	(3,+1)	(*i*)
O1···O1		0.064	0.740	(3,+1)	(*i*)
O1···Cl2		0.041	0.514	(3,+1)	(*i*)
N1···C5		0.028	0.355	(3,+1)	(*i*)
C3···C2		0.039	0.524	(3,+3)	(*i*)
C6···C5		0.039	0.524	(3,+3)	(*i*)
long (inter	dimer)				
Cl1···N1	3.531	0.031	0.409	(3,-1)	(*ii*)
		0.028	*0.482*		
C1···C8	3.464	0.028	0.331	(3,-1)	(*ii*)
		0.018	*0.241*		
C5···N1	3.521	0.031	0.369	(3,-1)	(*ii*)
		0.028	*0.484*		
Cl2···N2	3.545	0.030	0.395	(3,-1)	(*ii*)
		0.026	*0.482*		
C1···C1		0.032	0.340	(3,+1)	(*ii*)
O1···C1		0.027	0.306	(3,+1)	(*ii*)
O2···C2		0.026	0.300	(3,+1)	(*ii*)
O2···N1		0.014	0.188	(3,+1)	(*ii*)
N1···C5		0.030	0.376	(3,+1)	(*ii*)
Cl2···N2		0.030	0.395	(3,+1)	(*ii*)
N1···C3		0.026	0.300	(3,+1)	(*ii*)
N2···C1		0.027	0.329	(3,+1)	(*ii*)

S10 Intramolecular critical points at high pressures

Table S42 Topology of electron density of the 5,6-dichloro-2,3-dicyanosemiquinone radical anion, derived from electron-density after experimental multipole refinement at 0.25 GPa and from periodic calculations (in italic).

Bond	Length (Å)	Electron Density ($eÅ^{-3}$) ρ_{cp}	Laplacian ($eÅ^{-5}$)	Ellipticity
C1–O1	1.241(7)	2.744	-28.504	0.12
		2.581	*-5.470*	*0.01*
C4–O2	1.245(7)	2.731	-29.169	0.12
		2.507	*-9.253*	*0.04*
C1–C2	1.433(8)	1.960	-17.308	0.21
		1.825	*-14.783*	*0.20*
C2–C3	1.355(8)	2.236	-22.699	0.29
		2.012	*-16.440*	*0.32*
C3–C4	1.473(11)	1.859	-14.907	0.21
		1.961	*-16.990*	*0.20*
C4–C5	1.445(8)	1.930	-16.214	0.21
		1.808	*-14.816*	*0.18*
C5–C6	1.335(11)	2.378	-25.340	0.32
		2.201	*-19.230*	*0.41*
C6–C1	1.492(8)	1.806	-13.416	0.22
		1.853	*-15.467*	*0.19*
C5–Cl1	1.733(9)	1.338	-1.420	0.11
		1.357	*-6.123*	*0.08*
C6–Cl2	1.709(6)	1.392	-2.115	0.11
		1.330	*-5.801*	*0.08*
C2–C7	1.424(12)	1.917	-13.870	0.12
		1.899	*-16.574*	*0.10*
C7–N1	1.167(12)	3.369	-22.582	0.00
		3.157	*-2.935*	*0.02*
C3–C8	1.401(8)	1.977	-15.412	0.13
		1.833	*-15.505*	*0.10*
C8–N2	1.167(6)	3.367	-22.905	0.00
		3.156	*-2.593*	*0.02*

Table S43 Topology of electron density of the 5,6-dichloro-2,3-dicyanosemiquinone radical anion, derived from electron-density after experimental multipole refinement at 0.49 GPa and from periodic calculations (in italic).

Bond	Length (Å)	Electron Density ($eÅ^{-3}$) ρ_{cp}	Laplacian ($eÅ^{-5}$)	Ellipticity
C1–O1	1.234(6)	2.764	-27.286	0.11
		2.505	*-7.700*	*0.06*
C4–O2	1.255(6)	2.699	-30.380	0.12
		2.539	*-8.332*	*0.04*
C1–C2	1.452(7)	1.928	-16.179	0.23
		1.839	*-14.988*	*0.21*
C2–C3	1.358(8)	2.181	-22.239	0.24
		1.972	*-15.770*	*0.32*
C3–C4	1.458(9)	1.896	-15.810	0.21
		1.969	*-17.123*	*0.20*
C4–C5	1.446(7)	1.930	-16.209	0.22
		1.828	*-15.112*	*0.18*
C5–C6	1.362(8)	2.293	-22.932	0.32
		2.191	*-19.051*	*0.41*
C6–C1	1.480(9)	1.839	-14.156	0.22
		1.879	*-15.877*	*0.19*
C5–Cl1	1.708(8)	1.394	-2.121	0.11
		1.386	*-6.444*	*3.73*
C6–Cl2	1.696(5)	1.422	-2.514	0.11
		1.353	*-6.064*	*0.08*
C2–C7	1.423(9)	1.917	-14.009	0.12
		1.908	*-16.745*	*0.10*
C7–N1	1.179(9)	3.320	-25.720	0.00
		3.145	*-3.419*	*0.02*
C3–C8	1.420(7)	1.943	-14.098	0.15
		1.814	*-15.229*	*0.10*
C8–N2	1.178(6)	3.321	-25.691	0.00
		3.168	*-1.730*	*0.02*

Table S44 Topology of electron density of the 5,6-dichloro-2,3-dicyanosemiquinone radical anion, derived from electron-density after experimental multipole refinement at 0.86 GPa and from periodic calculations (in italic).

Bond	Length (Å)	Electron Density ($eÅ^{-3}$) ρ_{cp}	Laplacian ($eÅ^{-5}$)	Ellipticity
C1–O1	1.235(6)	2.762	-27.396	0.12
		2.517	*-7.426*	*0.06*
C4–O2	1.235(6)	2.764	-27.410	0.11
		2.520	*-9.014*	*0.04*
C1–C2	1.434(7)	1.974	-17.180	0.23
		1.815	*-14.648*	*0.20*
C2–C3	1.360(7)	2.178	-22.117	0.24
		2.005	*-16.322*	*0.32*
C3–C4	1.450(10)	1.919	-13.309	0.21
		1.990	*-17.475*	*0.20*
C4–C5	1.466(7)	1.873	-14.884	0.22
		1.792	*-14.587*	*0.17*
C5–C6	1.342(7)	2.355	-24.700	0.32
		2.173	*-18.702*	*0.40*
C6–C1	1.482(9)	1.835	-13.982	0.22
		1.889	*-16.018*	*0.19*
C5–Cl1	1.721(8)	1.365	-1.759	0.11
		1.404	*-6.647*	*0.08*
C6–Cl2	1.700(5)	1.412	-2.368	0.11
		1.357	*-6.090*	*0.08*
C2–C7	1.427(9)	1.906	-13.710	0.12
		1.902	*-16.652*	*0.10*
C7–N1	1.191(9)	3.271	-28.676	0.00
		3.146	*-3.342*	*0.02*
C3–C8	1.411(7)	1.971	-14.741	0.15
		1.828	*-15.445*	*0.10*
C8–N2	1.176(9)	3.332	-25.043	0.00
		3.166	*-1.900*	*0.023*

Table S45 Topology of electron density of the 5,6-dichloro-2,3-dicyanosemiquinone radical anion, derived from electron-density after experimental multipole refinement at 1.42 GPa and from periodic calculations (in italic).

Bond	Length (Å)	Electron Density ($eÅ^{-3}$) ρ_{cp}	Laplacian ($eÅ^{-5}$)	Ellipticity
C1–O1	1.240(6)	2.746	-28.382	0.12
		2.474	*-8.772*	*0.06*
C4–O2	1.241(6)	2.743	-28.670	0.12
		2.534	*-8.564*	*0.04*
C1–C2	1.441(7)	1.956	-16.780	0.23
		1.817	*-14.659*	*0.20*
C2–C3	1.359(7)	2.203	-22.319	0.26
		2.014	*-16.460*	*0.31*
C3–C4	1.441(9)	1.940	-16.820	0.21
		1.968	*-17.105*	*0.19*
C4–C5	1.468(7)	1.870	-14.800	0.22
		1.775	*-14.356*	*0.17*
C5–C6	1.353(7)	2.385	-25.550	0.32
		2.244	*-20.074*	*0.40*
C6–C1	1.473(9)	1.858	-14.494	0.22
		1.927	*-16.621*	*0.19*
C5–Cl1	1.720(8)	1.368	-1.787	0.11
		1.391	*-6.503*	*0.08*
C6–Cl2	1.705(5)	1.401	-2.206	0.11
		1.337	*-5.896*	*0.08*
C2–C7	1.423(7)	1.916	-13.931	0.12
		1.941	*-17.335*	*0.01*
C7–N1	1.178(9)	3.321	-25.665	0.00
		3.199	*-1.648*	*0.02*
C3–C8	1.418(7)	1.931	-14.288	0.12
		1.819	*-15.287*	*0.10*
C8–N2	1.170(5)	3.357	-23.395	0.00
		3.120	*-3.408*	*0.02*

Table S46 Topology of electron density of the 5,6-dichloro-2,3-dicyanosemiquinone radical anion, derived from electron-density after experimental multipole refinement at 1.85 GPa and from periodic calculations (in italic).

Bond	Length (Å)	Electron Density ($eÅ^{-3}$) ρ_{cp}	Laplacian ($eÅ^{-5}$)	Ellipticity
C1–O1	1.250(6)	2.713	-29.836	0.12
		2.503	*-7.712*	*0.06*
C4–O2	1.256(6)	2.697	-30.432	0.12
		2.496	*-10.121*	*0.04*
C1–C2	1.462(7)	1.902	-15.535	0.23
		1.831	*-14.941*	*0.20*
C2–C3	1.363(7)	2.169	-21.796	0.24
		2.130	*-18.556*	*0.31*
C3–C4	1.422(9)	1.992	-18.093	0.21
		1.973	*-17.110*	*0.19*
C4–C5	1.478(6)	1.849	-14.367	0.22
		1.802	*-14.700*	*0.17*
C5–C6	1.341(7)	2.361	-24.817	0.32
		2.231	*-19.761*	*0.40*
C6–C1	1.455(8)	1.905	-15.613	0.22
		1.873	*-15.664*	*0.19*
C5–Cl1	1.718(7)	1.371	-1.862	0.11
		1.357	*-6.025*	*0.08*
C6–Cl2	1.705(5)	1.403	-2.218	0.11
		1.388	*-6.507*	*0.08*
C2–C7	1.386(9)	2.022	-16.436	0.12
		2.005	*-18.315*	*0.10*
C7–N1	1.170(9)	3.360	-23.524	0.00
		3.039	*-7.712*	*0.02*
C3–C8	1.424(7)	1.937	-13.915	0.15
		1.870	*-16.146*	*0.10*
C8–N2	1.147(5)	3.452	-17.141	0.00
		3.160	*-2.651*	*0.02*

Table S47 Topology of electron density of the 5,6-dichloro-2,3-dicyanosemiquinone radical anion, derived from electron-density obtained after periodic calculations with geometry taken from 2.55 GPa experiment.

Bond	Length (Å)	Electron Density (eÅ^{-3}) ρ_{cp}	Laplacian (eÅ^{-5})	Ellipticity
C1–O1	1.528	2.472	-10.121	0.05
C4–O2	1.252	2.510	-9.399	0.04
C1–C2	1.439	2.472	-10.121	0.05
C2–C3	1.399	1.993	-16.146	0.30
C3–C4	1.406	2.030	-18.074	0.19
C4–C5	1.477	1.807	-14.941	0.16
C5–C6	1.368	2.125	-17.833	0.40
C6–C1	1.428	1.964	-17.351	0.19
C5–Cl1	1.716	1.365	-6.266	0.08
C6–Cl2	1.741	1.295	-5.543	0.08
C2–C7	1.374	2.054	-19.279	0.11
C7–N1	1.131	3.264	-5.241	0.02
C3–C8	1.427	1.854	-15.664	0.10
C8–N2	1.146	3.169	-2.169	0.02

Table S48 Topology of electron density of the 5,6-dichloro-2,3-dicyanosemiquinone radical anion, derived from electron-density after experimental multipole refinement at 3.09 GPa and from periodic calculations (in italic).

Bond	Length (Å)	Electron Density ($eÅ^{-3}$) ρ_{cp}	Laplacian ($eÅ^{-5}$)	Ellipticity
C1–O1	1.258(7)	2.691	-30.528	0.12
		2.508	*-9.158*	*0.05*
C4–O2	1.227(7)	2.790	-25.755	0.12
		2.584	*-6.748*	*0.05*
C1–C2	1.425(8)	1.986	-17.893	0.21
		1.871	*-15.423*	*0.20*
C2–C3	1.361(8)	2.196	-22.121	0.26
		1.963	*-15.664*	*0.30*
C3–C4	1.432(10)	1.966	-17.447	0.21
		2.035	*-18.074*	*0.20*
C4–C5	1.48538)	1.824	-13.825	0.22
		1.777	*-14.459*	*0.15*
C5–C6	1.373(8)	2.256	-21.916	0.32
		2.145	*-18.074*	*0.40*
C6–C1	1.457(10)	1.900	-15.488	0.22
		1.953	*-17.110*	*0.17*
C5–Cl1	1.679(8)	1.462	-3.084	0.11
		1.452	*-7.230*	*0.08*
C6–Cl2	1.689(6)	1.439	-2.757	0.11
		1.356	*-6.025*	*0.08*
C2–C7	1.441(11)	1.866	-12.851	0.12
		2.027	*-18.797*	*0.12*
C7–N1	1.150(11)	3.440	-17.763	0.00
		3.096	*-5.061*	*0.02*
C3–C8	1.425(8)	1.929	-13.808	0.12
		1.814	*-15.182*	*0.10*
C8–N2	1.169(7)	3.360	-23.130	0.00
		3.168	*-1.446*	*0.02*

Table S49 Topology of electron density of the 5,6-dichloro-2,3-dicyanosemiquinone radical anion, derived from electron-density after experimental multipole refinement at 3.95 GPa and from periodic calculations (in italic).

Bond	Length (Å)	Electron Density ($eÅ^{-3}$) ρ_{cp}	Laplacian ($eÅ^{-5}$)	Ellipticity
C1–O1	1.244(6)	2.733	-29.086	0.12
		2.541	*-8.194*	*0.05*
C4–O2	1.237(6)	2.758	-27.833	0.12
		2.577	*-7.230*	*0.05*
C1–C2	1.427(8)	1.995	-17.743	0.23
		1.853	*-15.182*	*0.20*
C2–C3	1.363(8)	2.191	-21.957	0.26
		1.979	*-15.905*	*0.31*
C3–C4	1.444(9)	1.933	-16.628	0.21
		2.007	*-17.592*	*0.19*
C4–C5	1.460(7)	1.892	-15.303	0.22
		1.801	*-14.941*	*0.15*
C5–C6	1.340(7)	2.364	-24.892	0.32
		2.155	*-18.315*	*0.40*
C6–C1	1.486(9)	1.824	-13.768	0.22
		1.921	*-16.628*	*0.17*
C5–Cl1	1.704(7)	1.406	-2.246	0.11
		1.452	*-7.230*	*0.08*
C6–Cl2	1.690(5)	1.436	-2.689	0.11
		1.375	*-6.266*	*0.08*
C2–C7	1.417(10)	1.936	-14.349	0.12
		2.009	*-18.556*	*0.11*
C7–N1	1.165(10)	3.375	-22.230	0.00
		3.162	*-2.892*	*0.02*
C3–C8	1.407(7)	1.962	-14.984	0.12
		1.829	*-15.423*	*0.10*
C8–N2	1.173(6)	3.343	-24.421	0.00
		3.150	*-2.410*	*0.02*

Table S50 Topology of electron density of the 5,6-dichloro-2,3-dicyanosemiquinone radical anion, derived from electron-density obtained after periodic calculations with geometry taken from 4.80 GPa experiment.

Bond	Length (Å)	Electron Density ($eÅ^{-3}$) ρ_{cp}	Laplacian ($eÅ^{-5}$)	Ellipticity
C1–O1	1.234	2.595	-6.507	0.04
C4–O2	1.236	2.588	-6.507	0.04
C1–C2	1.402	2.043	-18.315	0.21
C2–C3	1.414	1.939	-15.423	0.30
C3–C4	1.415	2.004	-17.592	0.19
C4–C5	1.477	1.810	-14.941	0.16
C5–C6	1.374	2.102	-17.351	0.41
C6–C1	1.469	1.834	-15.423	0.16
C5–Cl1	1.677	1.473	-7.471	0.08
C6–Cl2	1.684	1.445	-6.989	0.08
C2–C7	1.422	1.874	-16.146	0.11
C7–N1	1.148	3.156	-2.410	0.02
C3–C8	1.392	1.977	-17.833	0.11
C8–N2	1.149	3.154	-3.615	0.02

Table S51 Topology of electron density of the 5,6-dichloro-2,3-dicyanosemiquinone radical anion, derived from electron-density obtained after periodic calculations with geometry taken from 5.48 GPa experiment.

Bond	Length (Å)	Electron Density ($eÅ^{-3}$) ρ_{cp}	Laplacian ($eÅ^{-5}$)	Ellipticity
C1–O1	1.203	2.765	-1.446	0.04
C4–O2	1.248	2.530	-8.676	0.04
C1–C2	1.404	2.035	-18.074	0.20
C2–C3	1.424	1.904	-14.700	0.31
C3–C4	1.422	1.981	-17.351	0.19
C4–C5	1.439	1.934	-16.869	0.17
C5–C6	1.427	1.910	-14.218	0.40
C6–C1	1.458	1.865	-15.664	0.17
C5–Cl1	1.664	1.511	-7.712	0.08
C6–Cl2	1.681	1.454	-7.230	0.08
C2–C7	1.419	1.882	-16.146	0.12
C7–N1	1.174	3.006	-7.953	0.02
C3–C8	1.416	1.894	-16.387	0.11
C8–N2	1.188	2.935	-10.121	0.020

Table S52 Topology of electron density of the 5,6-dichloro-2,3-dicyanosemiquinone radical anion, derived from electron-density obtained after periodic calculations with geometry taken from 6.00 GPa experiment.

Bond	Length (Å)	Electron Density ($eÅ^{-3}$) ρ_{cp}	Laplacian ($eÅ^{-5}$)	Ellipticity
C1–O1	1.224	2.643	-5.061	0.03
C4–O2	1.268	2.433	-11.808	0.04
C1–C2	1.392	2.074	-19.279	0.15
C2–C3	1.447	1.832	-13.736	0.31
C3–C4	1.401	2.047	-18.797	0.15
C4–C5	1.471	1.823	-15.423	0.14
C5–C6	1.362	2.152	-18.315	0.42
C6–C1	1.476	1.792	-14.218	0.12
C5–Cl1	1.684	1.457	-7.230	0.10
C6–Cl2	1.688	1.437	-6.989	0.09
C2–C7	1.466	1.765	-16.869	0.08
C7–N1	1.094	3.498	-16.628	0.02
C3–C8	1.422	1.870	-16.146	0.07
C8–N2	1.152	3.137	-3.133	0.02

Table S53 Topology of electron density of the 4-cyano-*N*-methylpyridinium cation, derived from electron-density after experimental multipole refinement at 0.25 GPa and from periodic calculations (in italic).

Bond	Length (Å)	Electron Density ($eÅ^{-3}$) ρ_{cp}	Laplacian ($eÅ^{-5}$)	Ellipticity
N3–C9	1.355(7)	2.225	-21.505	0.09
		2.134	*-14.941*	*0.01*
N3–C13	1.343(7)	2.258	-22.354	0.08
		2.153	*-15.182*	*0.01*
N3–C14	1.475(10)	1.699	-9.518	0.05
		1.582	*-9.158*	*0.03*
C9–C10	1.363(8)	2.289	-23.201	0.17
		2.215	*-20.243*	*0.22*
C10–C11	1.374(7)	2.138	-20.804	0.22
		2.083	*-18.074*	*0.19*
C11–C12	1.376(8)	2.134	-20.689	0.22
		2.064	*-17.833*	*0.20*
C12–C13	1.376(9)	2.249	-22.150	0.18
		2.189	*-19.761*	*0.21*
C11–C15	1.439(8)	1.890	-12.988	0.15
		1.841	*-16.146*	*0.04*
C15–N4	1.161(7)	3.391	-20.994	0.00
		3.193	*0.000*	*0.03*
C9–H9	1.08(13)	1.807	-19.656	0.06
		2.618	*-43.860*	*0.02*
C10–H10	1.08(17)	1.771	-18.935	0.05
		2.585	*-42.414*	*0.00*
C12–H12	1.08(14)	1.770	-19.930	0.05
		2.579	*-41.932*	*0.00*
C13–H13	1.08(17)	1.807	-19.655	0.06
		2.620	*-44.101*	*0.01*
C14–H14A	1.08(9)	1.686	-16.479	0.09
		2.399	*-34.943*	*0.07*
C14–H14B	1.08(16)	1.686	-16.460	0.08
		2.392	*-34.702*	*0.07*
C14–H14C	1.08(14)	1.686	-16.489	0.09
		2.406	*-35.425*	*0.06*

Table S54 Topology of electron density of the 4-cyano-*N*-methylpyridinium cation, derived from electron-density after experimental multipole refinement at 0.49 Gpa and from periodic calculations (in italic).

Bond	Length (Å)	Electron Density ($eÅ^{-3}$) ρ_{cp}	Laplacian ($eÅ^{-5}$)	Ellipticity
N3–C9	1.350(6)	2.239	-21.913	0.09
		2.189	*-14.700*	*0.01*
N3–C13	1.353(6)	2.230	-21.626	0.09
		2.130	*-15.423*	*0.01*
N3–C14	1.507(9)	1.614	-7.646	0.05
		1.549	*-9.158*	*0.02*
C9–C10	1.375(7)	2.254	-22.286	0.18
		2.191	*-19.761*	*0.21*
C10–C11	1.368(6)	2.157	-21.279	0.22
		2.085	*-18.074*	*0.19*
C11–C12	1.376(7)	2.135	-20.623	0.22
		2.064	*-17.833*	*0.19*
C12–C13	1.358(8)	2.305	-23.651	0.17
		2.222	*-20.484*	*0.22*
C11–C15	1.425(7)	1.931	-13.848	0.15
		1.877	*-16.628*	*0.04*
C15–N4	1.170(6)	3.353	-23.584	0.00
		3.112	*-3.374*	*0.03*
C9–H9	1.08(19)	1.807	-19.668	0.06
		2.619	*-44.101*	*0.01*
C10–H10	1.1(2)	1.771	-18.928	0.05
		2.598	*-42.896*	*0.00*
C12–H12	1.08(19)	1.770	-18.909	0.05
		2.573	*-41.691*	*0.00*
C13–H13	1.1(3)	1.808	-19.665	0.06
		2.631	*-44.583*	*0.01*
C14–H14A	1.1(3)	1.685	-16.489	0.09
		2.415	*-35.425*	*0.06*
C14–H14B	1.08(10)	1.685	-16.459	0.09
		2.399	*-34.943*	*0.07*
C14–H14C	1.08(19)	1.685	-16.485	0.09
		2.389	*-34.461*	*0.07*

Table S55 Topology of electron density of the 4-cyano-*N*-methylpyridinium cation, derived from electron-density after multipole refinement at 0.86 GPa and from periodic calculations (in italic).

Bond	Length (Å)	Electron Density ($eÅ^{-3}$) ρ_{cp}	Laplacian ($eÅ^{-5}$)	Ellipticity
N3–C9	1.340(6)	2.267	-22.527	0.08
		2.178	*-14.700*	*0.01*
N3–C13	1.344(6)	2.257	-22.232	0.08
		2.161	*-14.941*	*0.01*
N3–C14	1.474(9)	1.703	-9.616	0.05
		1.554	*-9.158*	*0.02*
C9–C10	1.388(7)	2.216	-21.290	0.18
		2.176	*-19.520*	*0.21*
C10–C11	1.373(6)	2.142	-20.929	0.22
		2.068	*-17.833*	*0.19*
C11–C12	1.372(7)	2.143	-20.932	0.22
		2.080	*-18.074*	*0.20*
C12–C13	1.374(8)	2.257	-22.333	0.18
		2.225	*-20.484*	*0.21*
C11–C15	1.436(7)	1.900	-13.186	0.15
		1.849	*-16.387*	*0.04*
C15–N4	1.158(6)	3.405	-20.086	0.00
		3.154	*-1.687*	*0.03*
C9–H9	1.08(19)	1.806	-19.660	0.06
		2.626	*-44.342*	*0.01*
C10–H10	1.1(3)	1.770	-18.929	0.05
		2.587	*-42.414*	*0.00*
C12–H12	1.1(2)	1.770	-18.928	0.05
		2.573	*-41.691*	*0.00*
C13–H13	1.1(3)	1.807	-19.649	0.06
		2.628	*-44.342*	*0.01*
C14–H14A	1.08(9)	1.686	-16.473	0.09
		2.418	*-35.666*	*0.06*
C14–H14B	1.1(3)	1.686	-16.488	0.09
		2.400	*-34.943*	*0.07*
C14–H14C	1.08(16)	1.686	-16.472	0.09
		2.388	*-34.461*	*0.07*

Table S56 Topology of electron density of the 4-cyano-*N*-methylpyridinium cation, derived from electron-density after multipole refinement at 1.42 GPa and from periodic calculations (in italic).

Bond	Length (Å)	Electron Density ($eÅ^{-3}$) ρ_{cp}	Laplacian ($eÅ^{-5}$)	Ellipticity
N3–C9	1.342(5)	2.261	-22.415	0.08
		2.145	*-15.182*	*0.01*
N3–C13	1.338(6)	2.274	-22.606	0.08
		2.153	*-15.423*	*0.01*
N3–C14	1.471(8)	1.711	-9.785	0.05
		1.540	*-8.917*	*0.02*
C9–C10	1.383(7)	2.231	-21.661	0.18
		2.189	*-19.761*	*0.21*
C10–C11	1.374(5)	2.137	-20.784	0.22
		2.081	*-18.074*	*0.19*
C11–C12	1.3670(6)	2.159	-21.384	0.22
		2.069	*-17.833*	*0.20*
C12–C13	1.390(7)	2.210	-21.101	0.18
		2.225	*-20.484*	*0.21*
C11–C15	1.439(7)	1.893	-13.016	0.15
		1.823	*-15.905*	*0.04*
C15–N4	1.159(6)	3.397	-20.592	0.00
		3.151	*-1.928*	*0.03*
C9–H9	1.1(2)	1.806	-19.653	0.06
		2.622	*-44.101*	*0.01*
C10–H10	1.1(2)	1.770	-18.931	0.05
		2.591	*-42.655*	*0.00*
C12–H12	1.1(2)	1.770	-18.929	0.05
		2.579	*-41.932*	*0.00*
C13–H13	1.1(3)	1.806	-19.640	0.06
		2.624	*-44.101*	*0.01*
C14–H14A	1.08(9)	1.686	-16.481	0.09
		2.402	*-35.184*	*0.07*
C14–H14B	1.1(3)	1.686	-16.472	0.09
		2.405	*-35.184*	*0.06*
C14–H14C	1.08(16)	1.687	-16.482	0.09
		2.394	*-34.461*	*0.07*

Table S57 Topology of electron density of the 4-cyano-*N*-methylpyridinium cation, derived from electron-density after multipole refinement at 1.86 GPa and from periodic calculations (in italic).

Bond	Length (Å)	Electron Density ($eÅ^{-3}$) ρ_{cp}	Laplacian ($eÅ^{-5}$)	Ellipticity
N3–C9	1.346(6)	2.228	-21.659	0.09
		2.095	*-15.664*	*0.04*
N3–C13	1.342(5)	2.264	-22.427	0.08
		2.159	*-15.905*	*0.05*
N3–C14	1.463(8)	1.733	-10.3339	0.05
		1.635	*-10.603*	*0.03*
C9–C10	1.379(7)	2.241	-21.947	0.18
		2.100	*-18.315*	*0.23*
C10–C11	1.387(5)	2.100	-19.798	0.22
		2.061	*-17.833*	*0.20*
C11–C12	1.364(6)	2.166	-21.548	0.22
		2.147	*-19.279*	*0.21*
C12–C13	1.372(7)	2.265	-22.512	0.18
		2.131	*-18.797*	*0.23*
C11–C15	1.431(7)	1.915	-13.474	0.15
		1.869	*-16.628*	*0.04*
C15–N4	1.163(6)	3.384	-21.526	0.00
		3.071	*-5.061*	*0.03*
C9–H9	1.03(18)	1.807	-19.659	0.06
		1.917	*-22.894*	*0.02*
C10–H10	1.1(2)	1.768	-18.894	0.05
		1.871	*-21.448*	*0.01*
C12–H12	1.1(2)	1.771	-18.934	0.05
		1.892	*-21.448*	*0.01*
C13–H13	1.1(3)	1.807	-19.653	0.06
		1.905	*-22.653*	*0.02*
C14–H14A	1.08(14)	1.686	-16.456	0.09
		1.865	*-20.243*	*0.05*
C14–H14B	1.08(19)	1.687	-16.492	0.09
		1.865	*-20.243*	*0.05*
C14–H14C	1.1(2)	1.687	-16.473	0.09
		1.870	*-20.484*	*0.05*

Table S58 Topology of electron density of the 4-cyano-*N*-methylpyridinium cation, derived from electron-density after periodic calculations with geometry taken from 2.55 GPa experiment.

Bond	Length (Å)	Electron Density (eÅ^{-3}) ρ_{cp}	Laplacian (eÅ^{-5})	Ellipticity
N3–C9	1.354	2.103	-15.664	0.01
N3–C13	1.342	2.149	-15.423	0.01
N3–C14	1.450	1.670	-8.917	0.04
C9–C10	1.341	2.250	-20.966	0.21
C10–C11	1.385	2.070	-17.833	0.19
C11–C12	1.398	2.022	-17.110	0.19
C12–C13	1.323	2.326	-22.653	0.22
C11–C15	1.450	1.807	-15.664	0.04
C15–N4	1.120	1.807	-15.664	0.04
C9–H9	0.93	2.626	-44.342	0.01
C10–H10	0.929	2.591	-42.655	0.00
C12–H12	0.931	2.566	-41.450	0.00
C13–H13	0.93	2.627	-44.101	0.01
C14–H14A	0.96	2.399	-34.702	0.07
C14–H14B	0.96	2.394	-34.461	0.07
C14–H14C	0.96	2.412	-35.666	0.06

Table S59 Topology of electron density of the 4-cyano-*N*-methylpyridinium cation, derived from electron-density after multipole refinement at 3.09 GPa and from periodic calculations (in italic).

Bond	Length (Å)	Electron Density ($eÅ^{-3}$) ρ_{cp}	Laplacian ($eÅ^{-5}$)	Ellipticity
N3–C9	1.340(6)	2.269	-22.536	0.08
		2.148	*-15.182*	*0.00*
N3–C13	1.340(6)	2.272	-22.471	0.08
		2.116	*-15.905*	*0.01*
N3–C14	1.449(9)	1.772	-11.330	0.05
		1.612	*-9.158*	*0.02*
C9–C10	1.379(8)	2.242	-21.956	0.18
		2.182	*-19.761*	*0.21*
C10–C11	1.380(7)	2.121	-20.345	0.22
		2.043	*-17.592*	*0.19*
C11–C12	1.376(7)	2.135	-20.697	0.22
		2.092	*-18.315*	*0.20*
C12–C13	1.355(8)	2.315	-23.895	0.18
		2.205	*-20.002*	*0.22*
C11–C15	1.434(8)	1.903	-13.266	0.15
		1.852	*-16.387*	*0.04*
C15–N4	1.150(7)	3.439	-17.893	0.00
		3.229	*0.482*	*0.03*
C9–H9	1.1(2)	1.807	-19.657	0.06
		2.626	*-44.342*	*0.01*
C10–H10	1.1(3)	1.770	-18.924	0.05
		2.588	*-42.414*	*0.00*
C12–H12	1.1(2)	1.772	-18.947	0.05
		2.573	*-41.691*	*0.00*
C13–H13	1.1(3)	1.808	-19.651	0.06
		2.631	*-44.583*	*0.01*
C14–H14A	1.08(19)	1.687	-16.480	0.09
		2.411	*-35.425*	*0.06*
C14–H14B	1.08(9)	1.687	-16.476	0.09
		2.406	*-35.184*	*0.06*
C14–H14C	1.1(3)	1.687	-16.477	0.09
		2.389	*-34.220*	*0.07*

Table S60 Topology of electron density of the 4-cyano-*N*-methylpyridinium cation, derived from electron-density after multipole refinement at 3.95 GPa and from periodic calculations (in italic).

Bond	Length (Å)	Electron Density ($eÅ^{-3}$) ρ_{cp}	Laplacian ($eÅ^{-5}$)	Ellipticity
N3–C9	1.345(6)	2.254	-22.289	0.08
		2.187	*-14.700*	*0.01*
N3–C13	1.342(6)	2.265	-22.398	0.08
		2.159	*-15.182*	*0.01*
N3–C14	1.447(8)	1.775	-11.467	0.05
		1.605	*-9.399*	*0.02*
C9–C10	1.379(8)	2.240	-21.918	0.18
		2.162	*-19.279*	*0.21*
C10–C11	1.382(6)	2.116	-20.237	0.22
		2.089	*-18.315*	*0.19*
C11–C12	1.384(7)	2.116	-20.105	0.22
		2.034	*-17.351*	*0.20*
C12–C13	1.358(8)	2.303	-23.601	0.18
		2.222	*-20.484*	*0.22*
C11–C15	1.437(7)	1.895	-13.092	0.15
		1.836	*-16.146*	*0.03*
C15–N4	1.146(6)	3.453	-16.918	0.00
		3.282	*2.410*	*0.02*
C9–H9	1.1(2)	1.807	-19.663	0.06
		2.626	*-44.342*	*0.01*
C10–H10	1.1(3)	1.770	-18.929	0.05
		2.599	*-42.896*	*0.00*
C12–H12	1.1(2)	1.771	-18.941	0.05
		2.579	*-41.932*	*0.00*
C13–H13	1.1(3)	1.808	-19.654	0.06
		2.628	*-44.342*	*0.01*
C14–H14A	1.1(3)	1.687	-16.479	0.09
		2.404	*-34.943*	*0.06*
C14–H14B	1.08(19)	1.687	-16.477	0.09
		2.406	*-35.184*	*0.06*
C14–H14C	1.08(9)	1.687	-16.475	0.09
		2.391	*-34.461*	*0.07*

Table S61 Topology of electron density of the 4-cyano-*N*-methylpyridinium cation, derived from electron-density after after periodic calculations with geometry taken from 4.80 GPa experiment.

Bond	Length (Å)	Electron Density (eÅ^{-3}) ρ_{cp}	Laplacian (eÅ^{-5})	Ellipticity
N3–C9	1.323	1.549	-8.917	0.04
N3–C13	1.302	2.319	-13.254	0.00
N3–C14	1.488	1.549	-8.917	0.04
C9–C10	1.378	2.103	-18.315	0.20
C10–C11	1.371	2.126	-18.797	0.20
C11–C12	1.401	2.006	-16.869	0.20
C12–C13	1.352	2.199	-19.761	0.22
C11–C15	1.424	1.896	-17.110	0.04
C15–N4	1.152	3.138	-3.133	0.02
C9–H9	0.929	2.638	-44.583	0.01
C10–H10	0.928	2.601	-42.896	0.00
C12–H12	0.933	2.560	-41.209	0.00
C13–H13	0.931	2.624	-44.101	0.01
C14–H14A	0.961	2.393	-34.461	0.07
C14–H14B	0.962	2.388	-34.461	0.07
C14–H14C	0.958	2.431	-36.389	0.06

Table S62 Topology of electron density of the 4-cyano-*N*-methylpyridinium cation, derived from electron-density after periodic calculations with geometry taken from 5.48 GPa experiment.

Bond	Length (Å)	Electron Density ($eÅ^{-3}$) ρ_{cp}	Laplacian ($eÅ^{-5}$)	Ellipticity
N3–C9	1.309	2.292	-13.977	0.01
N3–C13	1.344	2.142	-15.182	0.01
N3–C14	1.466	1.618	-9.158	0.03
C9–C10	1.394	2.041	-17.110	0.21
C10–C11	1.398	2.025	-17.110	0.20
C11–C12	1.362	2.157	-19.520	0.20
C12–C13	1.361	2.162	-19.279	0.22
C11–C15	1.429	1.877	-16.869	0.03
C15–N4	1.129	3.279	1.446	0.03
C9–H9	0.932	2.618	-44.101	0.01
C10–H10	0.931	2.590	-42.414	0.01
C12–H12	0.93	2.574	-41.691	0.00
C13–H13	0.933	2.618	-43.860	0.01
C14–H14A	0.959	2.415	-35.425	0.06
C14–H14B	0.959	2.418	-35.425	0.06
C14–H14C	0.959	2.396	-34.461	0.07

Table S63 Topology of electron density of the 4-cyano-*N*-methylpyridinium cation, derived from electron-density after periodic calculations with geometry taken from 6.00 GPa experiment.

Bond	Length (Å)	Electron Density ($eÅ^{-3}$) ρ_{cp}	Laplacian ($eÅ^{-5}$)	Ellipticity
N3–C9	1.319	2.251	-14.218	0.02
N3–C13	1.320	2.244	-14.459	0.01
N3–C14	1.457	1.651	-9.158	0.04
C9–C10	1.368	2.142	-18.797	0.21
C10–C11	1.394	2.037	-17.351	0.19
C11–C12	1.364	2.151	-19.279	0.20
C12–C13	1.366	2.144	-18.797	0.21
C11–C15	1.443	1.831	-16.146	0.03
C15–N4	1.112	3.385	6.025	0.02
C9–H9	0.93	2.631	-44.342	0.01
C10–H10	0.929	2.589	-42.414	0.00
C12–H12	0.927	2.583	-42.173	0.00
C13–H13	0.928	2.641	-44.583	0.01
C14–H14A	0.959	2.404	-34.943	0.07
C14–H14B	0.959	2.403	-34.702	0.07
C14–H14C	0.962	2.404	-35.184	0.06

S11 Intermolecular critical points at high pressures

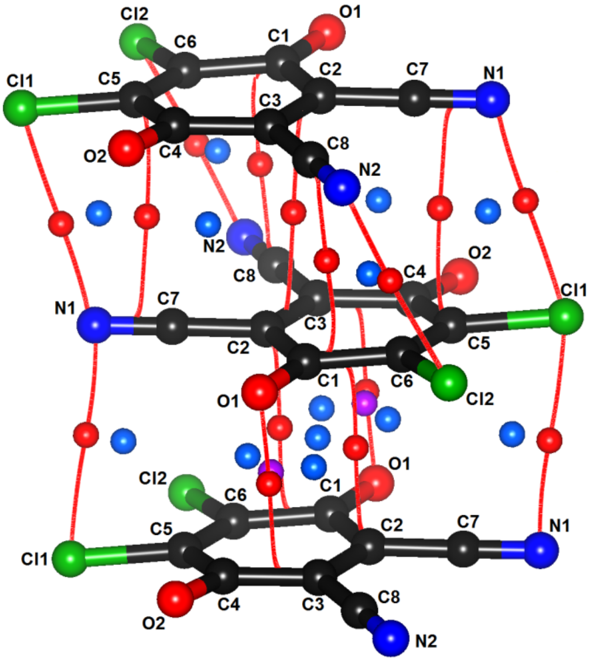

0.25 GPa

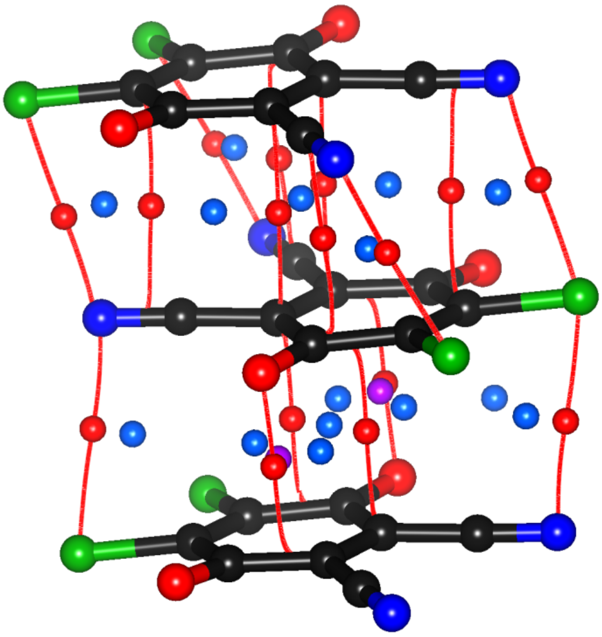

0.49 GPa

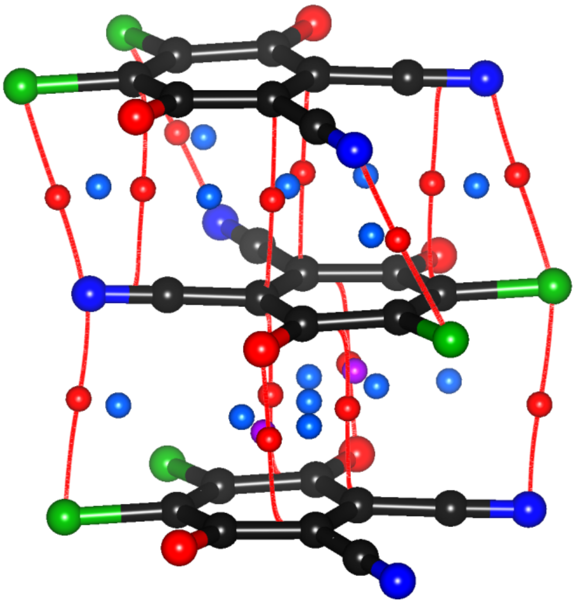

0.86 GPa

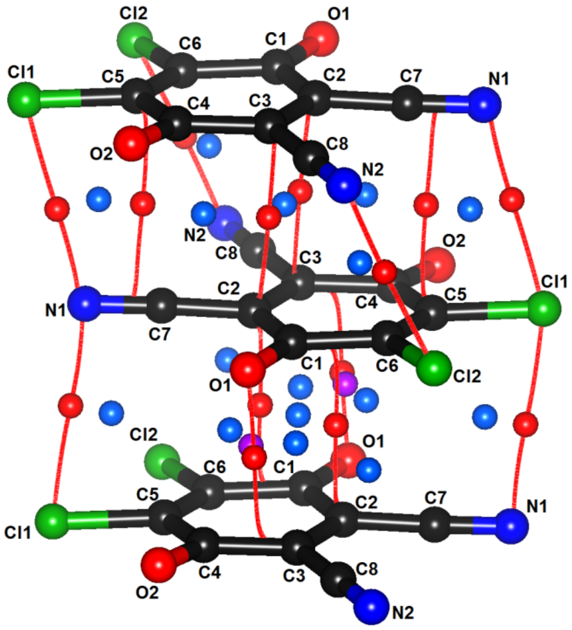

1.42 GPa

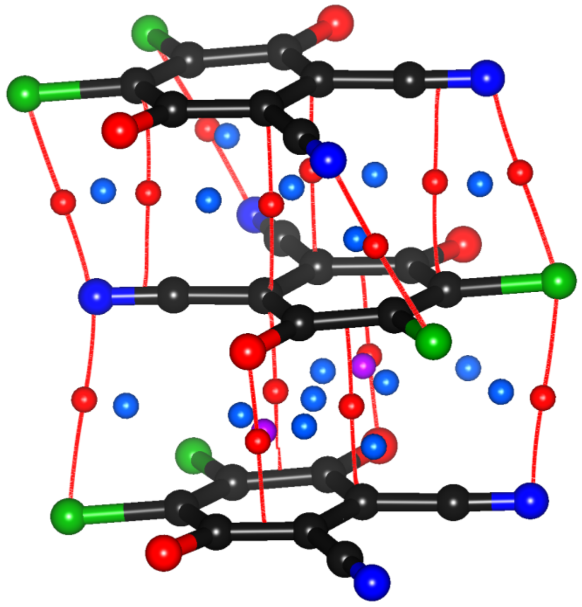

1.85 GPa

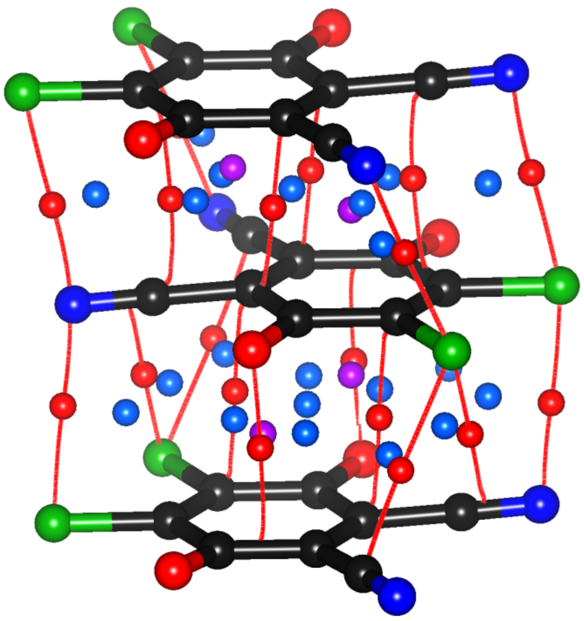

3.09 GPa

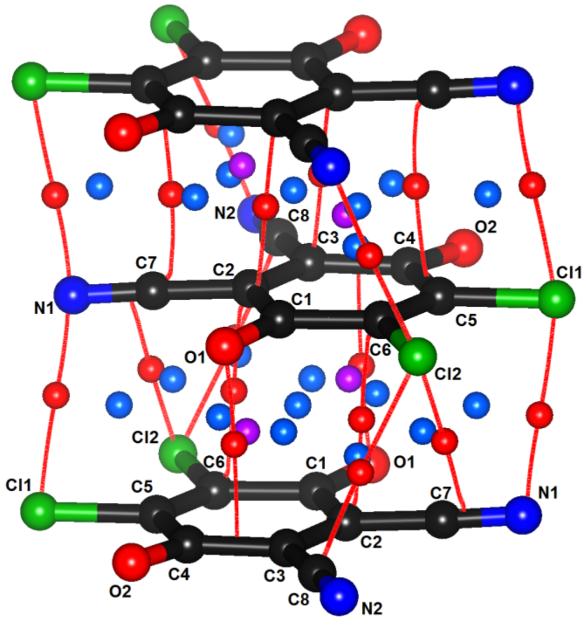

3.95 GPa

Figure S48 Critical points in a stack of DDQ radical anions at high pressures. Weaker inter-dimer contact is above and intra-dimer contact (multicentric bond) is below. (3,-1) critical points are shown as red spheres, (3,+1) as blue spheres and (3,+3) as purple spheres; intermolecular bond paths are shown as red lines.

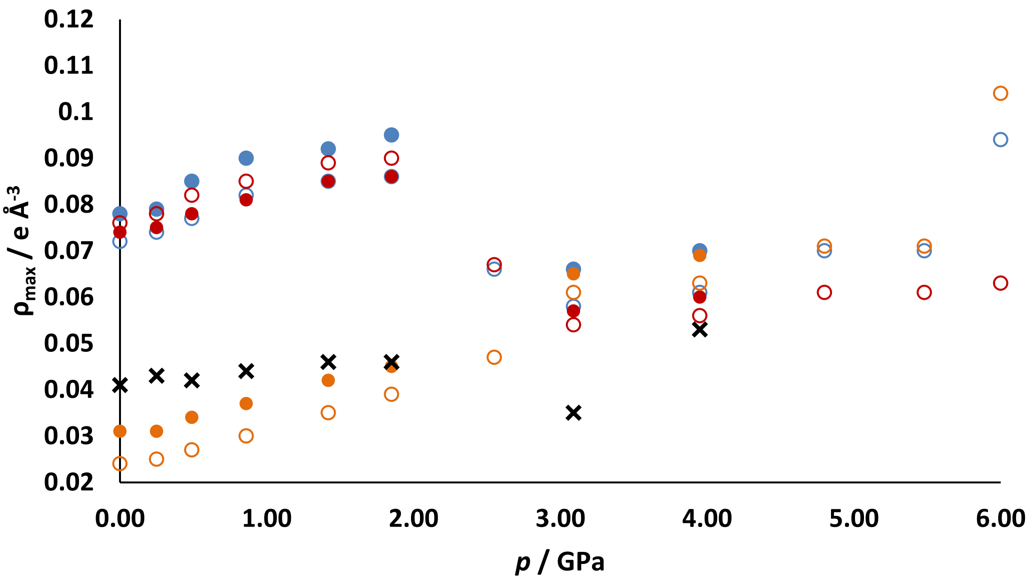

Figure S49 Electron density at (3,-1) critical points between DDQ radicals for HP data sets as a function of pressure for the contact A (symm. op. $-x$, $-y+1$, $-z$). Experimental cp's as full symbols and theoretical as open symbols. Electron density in the intra-dimer (3,+3) cp's are shown as black crosses.

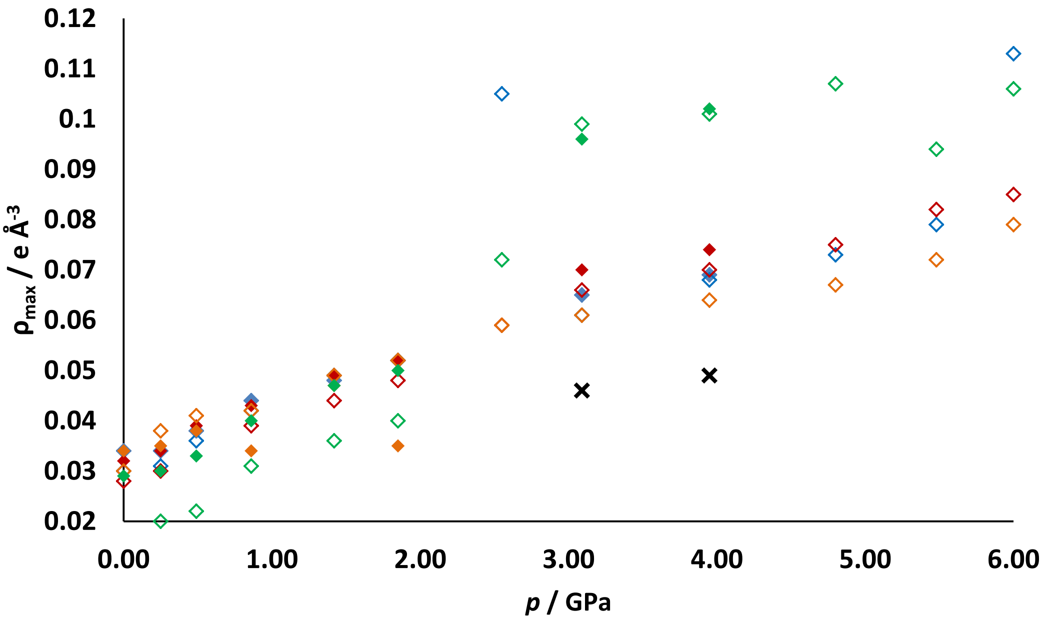

Figure S50 Electron density at (3,-1) critical points between DDQ radicals for HP data sets as a function of pressure for the contact B (symm. op. $-x+1$, $-y+1$, $-z$). Experimental cp's as full symbols and theoretical as open symbols. Electron density in the intra-dimer (3,+3) cp's are shown as black crosses.

Table S64 Total electron density ρ_{tot} and Laplacian $\nabla^2\rho$ at the critical points between the DDQ$^-$ radical anions at 0.25 GPa. Experimentally determined cp's are printed regular and theoretical ones from periodic calculations are italic. Symmetry operation on B: (*i*) $-x$, $-y+1$, $-z$; (*ii*) $-x+1$, $-y+1$, $-z$.

A…B	*d* (Å)	ρ_{tot} (e Å^{-3})	Laplacian (e Å^{-5})	CP type	Symm.
close (intra	dimer)				
C2···C1	3.016	0.079	0.856	(3,-1)	(*i*)
		0.074	*0.964*		
O1···C3	2.980	0.075	0.974	(3,-1)	(*i*)
		0.078	*0.919*		
N1···Cl1	3.552	0.031	0.409	(3,-1)	(*i*)
		0.025	*0.482*		
O1···C2		0.067	0.812	(3,+1)	(*i*)
O1···O1		0.072	0.831	(3,+1)	(*i*)
O1···Cl2		0.046	0.574	(3,+1)	(*i*)
N1···C5		0.029	0.401	(3,+1)	(*i*)
Cl1···C7		0.029	0.401	(3,+3)	(*i*)
C3···C7		0.043	0.589	(3,+3)	(*i*)
C6···C5		0.043	0.589	(3,+3)	(*i*)
long (inter	dimer)				
Cl1···N1	3.466	0.034	0.485	(3,-1)	(*ii*)
		0.031	*0.532*		
C1···C8	3.424	0.030	0.353	(3,-1)	(*ii*)
		0.020	*0.241*		
Cl2···N2	3.479	0.034	0.457	(3,-1)	(*ii*)
		0.030	*0.482*		
N1···C5	3.463	0.035	0.406	(3,-1)	(*ii*)
		0.038	*0.454*		
O2···O2	3.673	0.035	0.377	(3,+1)	(*ii*)
O2···N1		0.014	0.203	(3,+1)	(*ii*)
N1···C5		0.031	0.437	(3,+1)	(*ii*)
Cl1···C7		0.031	0.431	(3,+1)	(*ii*)
Cl2···C8		0.026	0.364	(3,+1)	(*ii*)
N1···C3		0.029	0.327	(3,+1)	(*ii*)
N2···C6		0.026	0.364	(3,+1)	(*ii*)

Table S65 Total electron density ρ_{tot} and Laplacian $\nabla^2\rho$ at the critical points between the DDQ^{-} radical anions at 0.49 GPa. Experimentally determined cp's are printed regular and theoretical ones from periodic calculations are italic. Symmetry operation on B: (*i*) –*x*, –*y*+1, –*z;* (*ii*) –*x*+1, –*y*+1, –*z*.

A…B	*d* (Å)	ρ_{tot} (e Å^{-3})	Laplacian (e Å^{-5})	CP type	Symm.
close (intra	dimer)				
C1···C2	2.993	0.085	0.887	(3,-1)	(*i*)
		0.077	*0.965*		
O1···C3	2.938	0.078	1.016	(3,-1)	(*i*)
		0.082	*0.958*		
N1···Cl1	3.501	0.034	0.441	(3,-1)	(*i*)
		0.027	*0.482*		
O1···C2		0.067	0.843	(3,+1)	(*i*)
O1···O1		0.076	0.857	(3,+1)	(*i*)
O1···Cl2		0.045	0.575	(3,+1)	(*i*)
O1···Cl1		0.045	0.575	(3,+1)	(*i*)
Cl1···C7		0.031	0.428	(3,+1)	(*i*)
N1···C5		0.031	0.428	(3,+1)	(*i*)
C1···O1		0.042	0.595	(3,+3)	(*i*)
C2···C3		0.042	0.595	(3,+3)	(*i*)
long (inter	dimer)				
Cl1···N1	3.413	0.038	0.540	(3,-1)	(*ii*)
		0.036	*0.593*		
C1···C8	3.365	0.033	0.388	(3,-1)	(*ii*)
		0.022	*0.482*		
C2···C3	3.422	0.036	0.401	(3,-1)	(*ii*)
		0.033	*0.555*		
C5···N1	3.429	0.038	0.442	(3,-1)	(*ii*)
		0.041	*0.491*		
Cl2···N2	3.427	0.039	0.504	(3,-1)	(*ii*)
C1···C1		0.035	0.381	(3,+1)	(*ii*)
N1···C5		0.034	0.476	(3,+1)	(*ii*)
N1···C1		0.031	0.354	(3,+1)	(*ii*)
Cl1···C7		0.034	0.476	(3,+1)	(*ii*)
Cl2···C8		0.029	0.392	(3,+1)	(*ii*)
N1···C3		0.031	0.354	(3,+1)	(*ii*)
N2···C6		0.029	0.392	(3,+1)	(*ii*)

Table S66 Total electron density ρ_{tot} and Laplacian $\nabla^2\rho$ at the critical points between the DDQ^{-} radical anions at 0.86 GPa. Experimentally determined cp's are printed regular and theoretical ones from periodic calculations are italic. Symmetry operation on B: (*i*) –*x*, –*y*+1, –*z;* (*ii*) –*x*+1, –*y*+1, –*z*.

A…B	*d* (Å)	ρ_{tot} (e Å^{-3})	Laplacian (e Å^{-5})	CP type	Symm.
close (intra	dimer)				
C1···C2	2.957	0.090	0.942	(3,-1)	(*i*)
		0.082	*1.016*		
O1···C3	2.916	0.081	1.067	(3,-1)	(*i*)
		0.085	*1.003*		
N1···Cl1	3.469	0.037	0.474	(3,-1)	(*i*)
		0.030	*0.482*		
O1···C2		0.071	0.891	(3,+1)	(*i*)
O1···O1		0.082	0.911	(3,+1)	(*i*)
O1···Cl2		0.047	0.597	(3,+1)	(*i*)
Cl1···C2		0.033	0.456	(3,+1)	(*i*)
Cl1···C7		0.033	0.456	(3,+1)	(*i*)
O2···C7		0.018	0.245	(3,+1)	(*i*)
C3···C2		0.044	0.621	(3,+3)	(*i*)
C6···C5		0.044	0.621	(3,+3)	(*i*)
long (inter	dimer)				
Cl1···N1	3.340	0.044	0.621	(3,-1)	(*ii*)
		0.042	*0.668*		
C2···C3	3.372	0.040	0.445	(3,-1)	(*ii*)
		0.031	*0.473*		
Cl2···N2	3.379	0.043	0.556	(3,-1)	(*ii*)
		0.039	*0.622*		
N1···C5	3.353	0.034	0.394	(3,-1)	(*ii*)
		0.042	*0.668*		
C2···C1		0.034	0.386	(3,+1)	(*ii*)
N1···C5		0.039	0.541	(3,+1)	(*ii*)
Cl1···C7		0.039	0.541	(3,+1)	(*ii*)
Cl2···C8		0.031	0.423	(3,+1)	(*ii*)
N1···C3		0.034	0.386	(3,+1)	(*ii*)
N2···C6		0.031	0.423	(3,+1)	(*ii*)
Cl1···Cl1		0.039	0.423	(3,+1)	(*ii*)

Table S67 Total electron density ρ_{tot} and Laplacian $\nabla^2\rho$ at the critical points between the DDQ$^-$ radical anions at 1.42 GPa. Experimentally determined cp's are printed regular and theoretical ones from periodic calculations are italic. Symmetry operation on B: (*i*) –*x*, –*y*+1, –*z;* (*ii*) –*x*+1, –*y*+1, –*z*.

A…B	*d* (Å)	ρ_{tot} (e Å^{-3})	Laplacian (e Å^{-5})	CP type	Symm.
close (intra	dimer)				
C1···C2	2.940	0.092	0.975	(3,-1)	(*i*)
		0.085	*1.072*		
O1···C3	2.904	0.085	1.105	(3,-1)	(*i*)
		0.089	*0.964*		
N1···Cl1	3.403	0.042	0.542	(3,-1)	(*i*)
		0.035	*0.482*		
Cl1···O1		0.049	0.620	(3,+1)	(*i*)
O1···C2		0.075	0.917	(3,+1)	(*i*)
O1···O1		0.084	0.935	(3,+1)	(*i*)
Cl1···C7		0.038	0.510	(3,+1)	(*i*)
Cl2···C3		0.036	0.419	(3,+1)	(*i*)
N2···Cl1		0.036	0.419	(3,+1)	(*i*)
C6···C5		0.046	0.642	(3,+3)	(*i*)
C3···C7		0.046	0.462	(3,+3)	(*i*)
long (inter	dimer)				
Cl1···N1	3.293	0.048	0.687	(3,-1)	(*ii*)
		0.049	*0.744*		
C2···C3	3.300	0.047	0.513	(3,-1)	(*ii*)
		0.036	*0.482*		
Cl2···N2	3.324	0.049	0.620	(3,-1)	(*ii*)
		0.044	*0.723*		
N1···C5	3.324	0.047	0.553	(3,-1)	(*ii*)
		0.049	0.744		
C1···C1		0.046	0.493	(3,+1)	(*ii*)
N1···C5		0.042	0.590	(3,+1)	(*ii*)
Cl1···C7		0.042	0.590	(3,+1)	(*ii*)
Cl2···C8		0.035	0.465	(3,+1)	(*ii*)
N1···C1		0.038	0.438	(3,+1)	(*ii*)
N1···C3		0.038	0.438	(3,+1)	(*ii*)
N2···C6		0.035	0.465	(3,+1)	(*ii*)

Table S68 Total electron density ρ_{tot} and Laplacian $\nabla^2\rho$ at the critical points between the DDQ$^-$ radical anions at 1.85 GPa. Experimentally determined cp's are printed regular and theoretical ones from periodic calculations are italic. Symmetry operation on B: (*i*) –*x*, –*y*+1, –*z;* (*ii*) –*x*+1, –*y*+1, –*z*.

A…B	*d* (Å)	ρ_{tot} (e Å^{-3})	Laplacian (e Å^{-5})	CP type	Symm.
close (intra	dimer)				
C6···C2	2.954	0.095	0.996	(3,-1)	(*i*)
		0.086	*1.100*		
O1···C3	2.899	0.086	1.133	(3,-1)	(*i*)
		0.090	*1.071*		
N1···Cl1	3.370	0.045	0.581	(3,-1)	(*i*)
		0.039	*0.723*		
O1···C2		0.074	0.928	(3,+1)	(*i*)
O1···O1		0.085	0.940	(3,+1)	(*i*)
O1···Cl2		0.049	0.624	(3,+1)	(*i*)
Cl1···C1		0.049	0.624	(3,+1)	(*i*)
Cl1···C2		0.039	0.541	(3,+1)	(*i*)
Cl1···C7		0.046	0.639	(3,+1)	(*i*)
Cl2···C3		0.037	0.443	(3,+1)	(*i*)
N1···C5		0.039	0.541	(3,+1)	(*i*)
N1···O1		0.037	0.443	(3,+1)	(*i*)
O1···C1		0.046	0.647	(3,+3)	(*i*)
C2···C3		0.046	0.647	(3,+3)	(*i*)
long (inter	dimer)				
Cl1···N1	3.261	0.052	0.737	(3,-1)	(*ii*)
		0.052	*0.723*		
C2···C3	3.262	0.050	0.549	(3,-1)	(*ii*)
		0.040	*0.590*		
Cl2···N2	3.261	0.052	0.737	(3,-1)	(*ii*)
		0.048	*0.723*		
N1···C5	3.276	0.052	0.602	(3,-1)	(*ii*)
		0.052	*0.778*		
C1···C1		0.047	0.515	(3,+1)	(*ii*)
O2···C2		0.042	0.475	(3,+1)	(*ii*)
N1···C5		0.046	0.639	(3,+1)	(*ii*)
Cl2···C8		0.037	0.501	(3,+1)	(*ii*)
C1···C5		0.041	0.475	(3,+1)	(*ii*)
N2···C6		0.037	0.501	(3,+1)	(*ii*)

N2···C1	0.027	0.421	(3,+1)	(*ii*)

Table S69 Total electron density ρ_{tot} and Laplacian $\nabla^2\rho$ at the (3,–1) critical points between the DDQ^{-} radical anions at 2.55 GPa obtained from periodic calculations. Symmetry operation on B: (*i*) –*x*, –*y*+1, –*z;* (*ii*) –*x*+1, –*y*+1, –*z*. Units: *d* Å, ρ_{tot} e Å^{-3}, Laplacian e Å^{-5}

A…B	*d* (Å)	ρ_{tot} (e Å^{-3})	Laplacian (e Å^{-5})	CP type	Symm.
intradimer	(*i*)				
C6···C2	3.039	0.066	0.899	(3,-1)	(*i*)
O1···C3	2.912	0.067	0.894	(3,-1)	(*i*)
N1···Cl1	3.300	0.047	0.723	(3,-1)	(*i*)
intradimer	(*ii*)				
Cl1···N1	2.893	0.105	1.205	(3,-1)	(*ii*)
C2···C3	3.054	0.072	0.964	(3,-1)	(*ii*)
Cl2···N2	3.203	0.059	0.964	(3,-1)	(*ii*)
N1···C5	3.249	0.059	0.874	(3,-1)	(*ii*)

Table S70 Total electron density ρ_{tot} and Laplacian $\nabla^2\rho$ at the critical points between the DDQ$^-$ radical anions at 3.09 GPa. Experimentally determined cp's are printed regular and theoretical ones from periodic calculations are italic. Symmetry operation on B: (*i*) $-x$, $-y$+1, $-z$; (*ii*) $-x$+1, $-y$+1, $-z$.

A…B	*d* (Å)	ρ_{tot} (e Å^{-3})	Laplacian (e Å^{-5})	CP type	Symm.
intradimer	(*i*)				
C6···C2	3.109	0.066	0.738	(3,-1)	(*i*)
		0.058	*0.723*		
O1···C4	3.022	0.057	0.742	(3,-1)	(*i*)
		0.054	*0.723*		
N1···Cl1	3.191	0.065	0.807	(3,-1)	(*i*)
		0.061	*0.964*		
Cl2···C7	3.248	0.058	0.773	(3,-1)	(*i*)
		0.053	*0.964*		
Cl2···C8	3.469	0.042	0.452	(3,-1)	(*i*)
		0.029	*0.482*		
O1···C2		0.049	0.605	(3,+1)	(*i*)
O1···O1		0.052	0.593	(3,+1)	(*i*)
O1···Cl2		0.037	0.459	(3,+1)	(*i*)
Cl1···C1		0.037	0.459	(3,+1)	(*i*)
Cl1···C2		0.046	0.616	(3,+1)	(*i*)
N2···C6		0.056	0.752	(3,+1)	(*i*)
Cl2···C2		0.037	0.482	(3,+1)	(*i*)
N1···C5		0.046	0.616	(3,+1)	(*i*)
N1···O2		0.028	0.404	(3,+1)	(*i*)
N1···C6		0.057	0.779	(3,+1)	(*i*)
O1···C1		0.035	0.448	(3,+3)	(*i*)
intradimer	(*ii*)				
Cl1···N1	3.174	0.065	0.883	(3,-1)	(*ii*)
		0.061	*0.964*		
C2···C3	2.913	0.096	1.057	(3,-1)	(*ii*)
		0.099	*1.205*		
C5···C7	3.019	0.073	0.826	(3,-1)	(*ii*)
		0.061	*0.964*		
Cl2···N2	3.153	0.070	0.895	(3,-1)	(*ii*)
		0.066	*0.964*		
C1···C1		0.089	0.960	(3,+1)	(*ii*)
O2···C3		0.072	0.827	(3,+1)	(*ii*)

N1···C1	0.047	0.566	(3,+1)	(*ii*)
Cl1···C7	0.058	0.788	(3,+1)	(*ii*)
Cl2···C2	0.047	0.566	(3,+1)	(*ii*)
Cl2···C3	0.056	0.752	(3,+1)	(*ii*)
N1···C5	0.058	0.788	(3,+1)	(*ii*)
N1···C3	0.072	0.827	(3,+1)	(*ii*)
C1···C6	0.046	0.600	(3,+3)	(*ii*)
C5···C4	0.046	0.600	(3,+3)	(*ii*)

Table S71 Total electron density ρ_{tot} and Laplacian $\nabla^2\rho$ at the critical points between the DDQ$^-$ radical anions at 3.95 GPa. Experimentally determined cp's are printed regular and theoretical ones from periodic calculations are italic. Symmetry operation on B: (*i*) –*x*, –*y*+1, –*z;* (*ii*) –*x*+1, –*y*+1, –*z*.

A…B		*d* (Å)	ρ_{tot} (e Å^{-3})	Laplacian (e Å^{-5})	CP type	Symm.
intradimer	(*i*)					
C6⋯C2		3.092	0.070	0.766	(3,-1)	(*i*)
			0.061	*0.964*		
O1⋯C3		3.097	0.060	0.777	(3,-1)	(*i*)
			0.056	*0.723*		
N1⋯Cl1		3.164	0.069	0.848	(3,-1)	(*i*)
			0.063	*0.964*		
Cl2⋯C8		3.448	0.044	0.471	(3,-1)	(*i*)
			0.031	*0.482*		
Cl1⋯O1			0.012	0.242	(3,+1)	(*i*)
O1⋯C2			0.053	0.640	(3,+1)	(*i*)
O1⋯O1			0.056	0.631	(3,+1)	(*i*)
O1⋯Cl2			0.040	0.488	(3,+1)	(*i*)
Cl1⋯C1			0.040	0.488	(3,+1)	(*i*)
Cl1⋯C2			0.047	0.638	(3,+1)	(*i*)
C6⋯N1			0.058	0.801	(3,+1)	(*i*)
Cl2⋯C2			0.038	0.504	(3,+1)	(*i*)
N1⋯C5			0.047	0.638	(3,+1)	(*i*)
N1⋯C6			0.058	0.801	(3,+1)	(*i*)
O1⋯C1			0.038	0.476	(3,+3)	(*i*)
N1⋯O2			0.021	0.285	(3,+3)	(*i*)
intradimer	(*ii*)					
Cl1⋯N1		3.145	0.069	0.931	(3,-1)	(*ii*)
			0.068	*0.984*		
C2⋯C3		2.905	0.102	1.082	(3,-1)	(*ii*)
			0.101	*1.205*		
C5⋯C7		2.989	0.078	0.880	(3,-1)	(*ii*)
			0.064	*0.964*		
Cl2⋯N2		3.130	0.074	0.932	(3,-1)	(*ii*)
			0.070	*0.964*		
C1⋯C1			0.089	0.979	(3,+1)	(*ii*)
O2⋯C3			0.077	0.879	(3,+1)	(*ii*)
O2⋯C7			0.024	0.247	(3,+1)	(*ii*)

Cl1···C7	0.061	0.822	(3,+1)	(*ii*)
Cl2···C2	0.050	0.604	(3,+1)	(*ii*)
N1···C5	0.061	0.822	(3,+1)	(*ii*)
N1···C3	0.077	0.879	(3,+1)	(*ii*)
Cl2···C8	0.058	0.781	(3,+1)	(*ii*)
N1···C2	0.050	0.604	(3,+1)	(*ii*)
N2···C6	0.058	0.781	(3,+1)	(*ii*)
C1···C6	0.049	0.651	(3,+3)	(*ii*)
C1···N1	0.048	0.651	(3,+3)	(*ii*)

Table S72 Total electron density ρ_{tot} and Laplacian $\nabla^2\rho$ at the (3,–1) critical points between the DDQ$^-$ radical anions at 4.80 GPa obtained from periodic calculations. Symmetry operation on B: (*i*) –*x*, –*y*+1, –*z;* (*ii*) –*x*+1, –*y*+1, –*z*. Units: *d* Å, ρ_{tot} e Å^{-3}, Laplacian e Å^{-5}

A…B	*d* (Å)	ρ_{tot} (e Å^{-3})	Laplacian (e Å^{-5})	CP type	Symm.
intradimer (*i*)					
C6···C2	3.015	0.070	0.964	(3,-1)	(*i*)
O1···C4	3.090	0.061	0.964	(3,-1)	(*i*)
N1···Cl1	3.117	0.071	0.964	(3,-1)	(*i*)
Cl2···C7	3.193	0.061	0.964	(3,-1)	(*i*)
Cl2···C8	3.414	0.034	0.723	(3,-1)	(*i*)
intradimer (*ii*)					
Cl1···N1	3.110	0.073	0.964	(3,-1)	(*ii*)
C2···C3	2.907	0.107	1.205	(3,-1)	(*ii*)
C5···C7	2.985	0.067	0.964	(3,-1)	(*ii*)
Cl2···N2	3.098	0.075	0.964	(3,-1)	(*ii*)

Table S73 Total electron density ρ_{tot} and Laplacian $\nabla^2\rho$ at the (3,–1) critical points between the DDQ$^-$ radical anions at 5.48 GPa obtained from periodic calculations. Symmetry operation on B: (*i*) –*x*, –*y*+1, –*z;* (*ii*) –*x*+1, –*y*+1, –*z*. Units: *d* Å, ρ_{tot} e Å^{-3}, Laplacian e Å^{-5}

A…B	*d* (Å)	ρ_{tot} (e Å^{-3})	Laplacian (e Å^{-5})	CP type	Symm.
intradimer (*i*)					
C6···C2	3.017	0.070	0.964	(3,-1)	(*i*)
O1···C3	3.059	0.061	0.964	(3,-1)	(*i*)
N1···Cl1	3.096	0.075	0.964	(3,-1)	(*i*)
Cl2···C7	3.190	0.061	0.964	(3,-1)	(*i*)
Cl2···C8	3.407	0.035	0.723	(3,-1)	(*i*)
intradimer (*ii*)					
Cl1···N1	3.007	0.079	1.113	(3,-1)	(*ii*)
C2···C3	2.872	0.113	1.205	(3,-1)	(*ii*)
C5···C7	2.968	0.072	0.964	(3,-1)	(*ii*)
Cl2···N2	3.064	0.082	1.205	(3,-1)	(*ii*)

Table S74 Total electron density ρ_{tot} and Laplacian $\nabla^2\rho$ at the (3,–1) critical points between the DDQ$^-$ radical anions at 6.00 GPa obtained from periodic calculations. Symmetry operation on B: (*i*) –*x*, –*y*+1, –*z;* (*ii*) –*x*+1, –*y*+1, –*z*. Units: *d* Å, ρ_{tot} e Å^{-3}, Laplacian e Å^{-5}

A…B		*d*	ρ_{tot}	Laplacian	CP type	Symm.
intradimer	(*i*)					
C6···C2		2.977	0.094	0.723	(3,-1)	(*i*)
O1···C3		3.054	0.063	0.723	(3,-1)	(*i*)
N1···Cl1		3.080	0.104	0.482	(3,-1)	(*i*)
Cl2···C7		3.205	0.065	0.964	(3,-1)	(*i*)
Cl2···C8		3.363	0.040	0.723	(3,-1)	(*i*)
intradimer	(*ii*)					
Cl1···N1		3.057	0.113	0.241	(3,-1)	(*ii*)
C2···C3		2.875	0.106	1.205	(3,-1)	(*ii*)
C5···C7		2.921	0.079	0.964	(3,-1)	(*ii*)
Cl2···N2		3.042	0.085	1.205	(3,-1)	(*ii*)

S12 Calculated HOMO and LUMO orbitals at high pressures

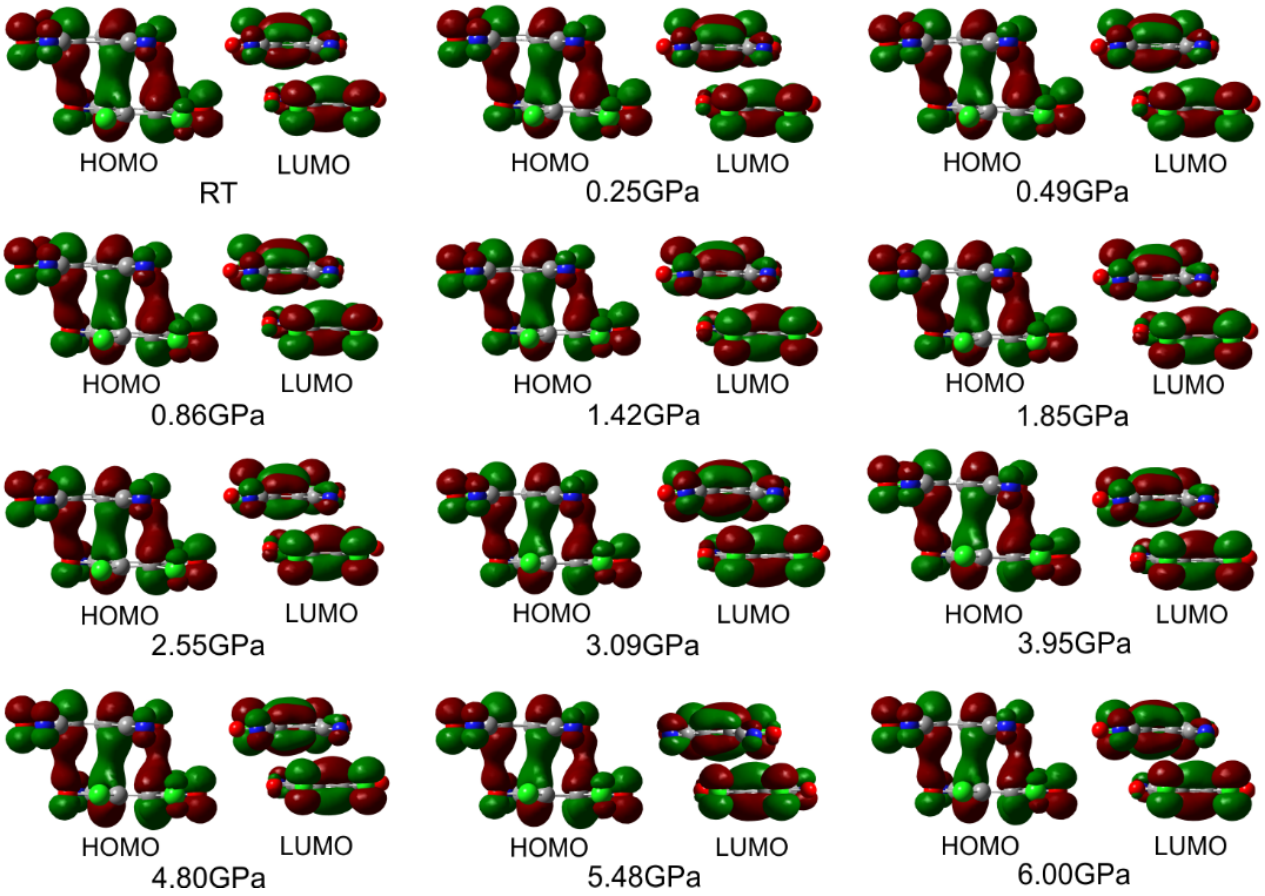

Figure S51 Lowest occupied molecular orbital (LUMO) and highest occupied molecular orbital (HOMO) for a dimer between DDQ radicals for contact A.

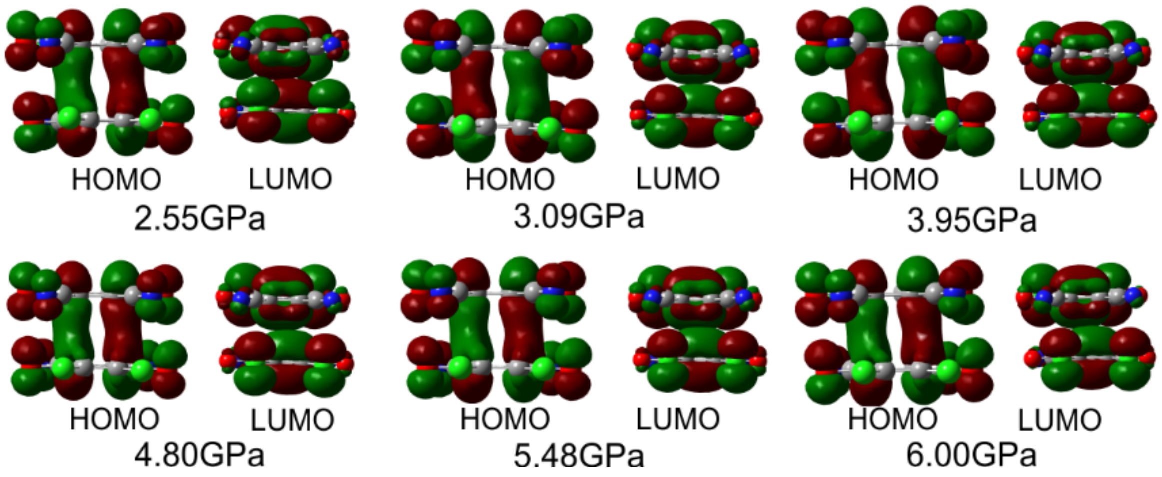

Figure S52 Lowest occupied molecular orbital (LUMO) and highest occupied molecular orbital (HOMO) for a dimer between DDQ radicals for contact B.

S13 Geometric parameters of stacking at variable temperatures

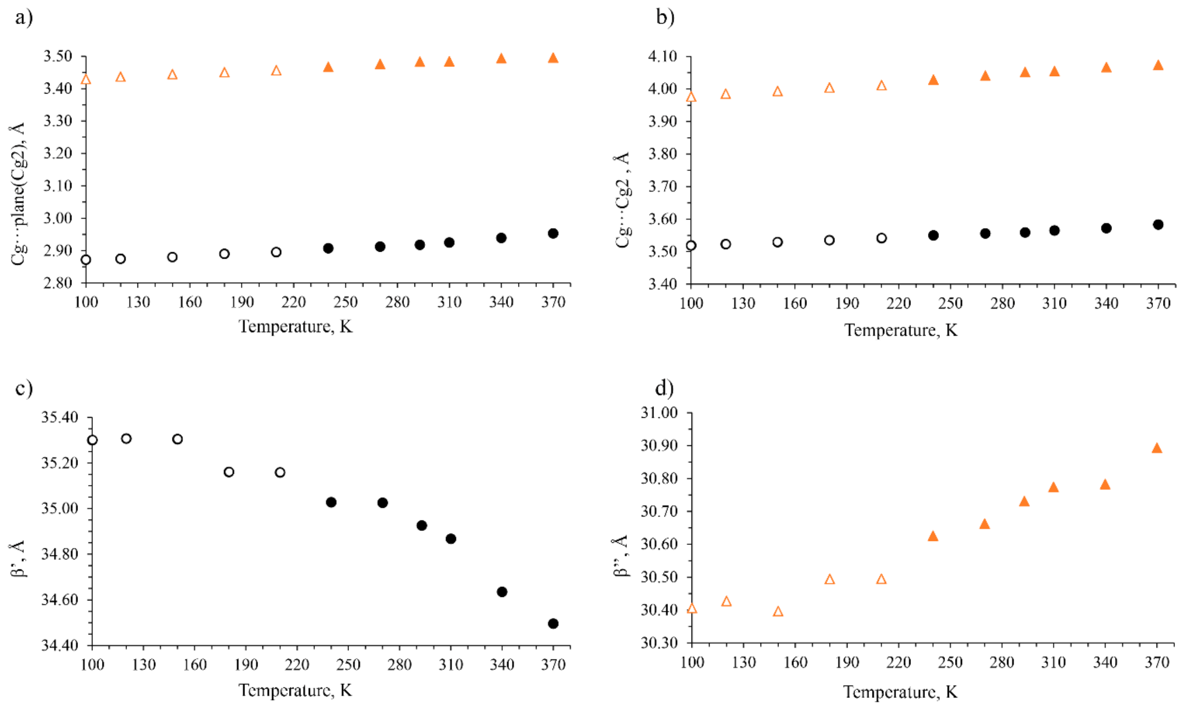

Figure S53 Variation of interplanar distances with temperature plotted at a) and b). Inter-dimer contacts (A) are shown as black dots, and longer inter-dimer contacts (B) are shown as orange triangles. The changes in β' and β'' *vs* T are plotted at c) and d) respectively.

S14 Geometric parameters of stacking at high pressures

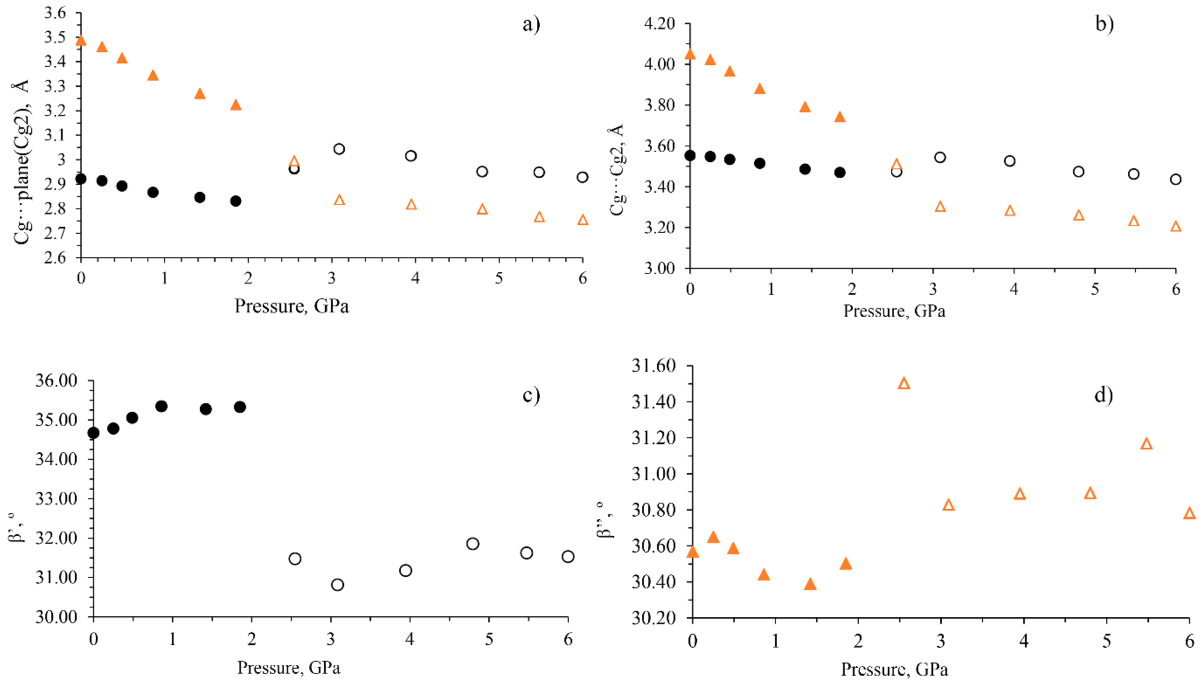

Figure S54 Variation of interplanar distances with pressure. Inter-dimer contacts (A) are shown as black circles, and longer inter-dimer contacts (B) are shown as orange triangles. The changes in β' and β'' *vs* P are plotted at c) and d) respectively. A phase transformation occurs between 1.85 and 2.5 GPa.

S15 References

[1] Farrugia, L. J. (1997). *ORTEP*-3 for Windows - a version of *ORTEP*-III with a Graphical User Interface (GUI), *J. Appl. Crystallogr.*, **30**, 565.

[2] Milašinović, V.; Krawczuk, A.; Molčanov, K. & Kojić-Prodić, B. (2020). *Cryst. Growth Des.*, **20**, 5435-5443.

[3] Domagała, S.; Fournier, B.; Liebschner, D.; Guillot, B. & Jelsch, C. (2012). *Acta Crystallogr. A*, **A68**, 337-351.

[4] Meindl, K. & Henn, J. (2008). *Acta Crystallogr. A*, **A64**, 404-418.
